# Evolution and expression of the duck TRIM gene repertoire

**DOI:** 10.3389/fimmu.2023.1220081

**Published:** 2023-08-09

**Authors:** Lee K. Campbell, Rhiannon M. Peery, Katharine E. Magor

**Affiliations:** ^1^ Department of Biological Sciences, University of Alberta, Edmonton, AB, Canada; ^2^ Li Ka Shing Institute of Virology, University of Alberta, Edmonton, AB, Canada; ^3^ Department of Biology, Carleton University, Ottawa, ON, Canada

**Keywords:** TRIM protein, evolution, B30.2/PRY-SPRY domains, mallard duck, gene duplication

## Abstract

Tripartite motif (TRIM) proteins are involved in development, innate immunity, and viral restriction. TRIM gene repertoires vary between species, likely due to diversification caused by selective pressures from pathogens; however, this has not been explored in birds. We mined a *de novo* assembled transcriptome for the TRIM gene repertoire of the domestic mallard duck (*Anas platyrhynchos*), a reservoir host of influenza A viruses. We found 57 TRIM genes in the duck, which represent all 12 subfamilies based on their C-terminal domains. Members of the C-IV subfamily with C-terminal PRY-SPRY domains are known to augment immune responses in mammals. We compared C-IV TRIM proteins between reptiles, birds, and mammals and show that many C-IV subfamily members have arisen independently in these lineages. A comparison of the MHC-linked C-IV TRIM genes reveals expansions in birds and reptiles. The TRIM25 locus with related innate receptor modifiers is adjacent to the MHC in reptile and marsupial genomes, suggesting the ancestral organization. Within the avian lineage, both the MHC and TRIM25 loci have undergone significant TRIM gene reorganizations and divergence, both hallmarks of pathogen-driven selection. To assess the expression of TRIM genes, we aligned RNA-seq reads from duck tissues. C-IV TRIMs had high relative expression in immune relevant sites such as the lung, spleen, kidney, and intestine, and low expression in immune privileged sites such as in the brain or gonads. Gene loss and gain in the evolution of the TRIM repertoire in birds suggests candidate immune genes and potential targets of viral subversion.

## 1 Introduction

Tripartite motif (TRIM) proteins comprise a large family with important roles in development (1, 2), cell cycle (3), immunity (4–7), autophagy (3, 7), and various other intracellular functions (7, 8). Comparisons of TRIM proteins across species demonstrate a rapidly expanding repertoire during eukaryote evolution. Humans have more than 80 TRIM genes, while mice have approximately 60, zebrafish have approximately 208, worms have more than 20, and flies have more than 10 (5, 9–12). No avian TRIM repertoire has been systematically analyzed. An early estimate identified 37 in the chicken (9), but this has not been updated with improved genomic resources. The ambiguity of TRIM gene number in each species arises from their locations on many different chromosomes, each evolving independently, responding to pressures from pathogens and undergoing species-specific expansions (13–15).

TRIM proteins are characterized by the presence of three domains: the really interesting new gene (RING) domain, one or two B-box domains, and a coiled-coil (CC) domain. These domains together are termed the RBCC motif. TRIM proteins possess E3 ubiquitin ligase activity due to their RING domain, which helps conjugate polyubiquitin to target proteins (16, 17). B-Box domains can also perform E3 ligase activity (18–20), higher order multimerization, and binding substrate proteins (21–24). The CC domain is involved in homo- or heteromeric assemblies (25, 26). The C-terminal domains are often responsible for substrate recognition. Substrates can range from intracellular proteins, pathogen proteins, or nucleic acids (27–30).

TRIM proteins are commonly categorized into 11 subfamilies defined by variable C-terminal domains, termed C-I to C-XI (5, 12, 31, 32). Sardiello and colleagues suggested that the TRIM family broadly separates into two groups (9). Group I is more conserved through evolution and is composed of all 11 subfamilies. Group II contains only TRIM proteins belonging to the C-IV subfamily, which contain C-terminal B30.2/PRY-SPRY domains. The combined B30.2/PRY-SPRY domains arose later in evolution than the SPRY domain and is often associated with immune function (33). Members of group II appear to evolve faster than members in group I (9). Marín and colleagues demonstrated that the evolution of TRIM proteins is more complex and classified TRIM proteins into nine subfamilies, based on when the TRIM proteins arose in eukaryotes (8). The C-IV subfamily dramatically expanded in vertebrates. Members of the C-IV subfamily are regulated by immune responses (6, 9, 34), and many C-IV type TRIMs have direct roles in immunity and viral restriction (4, 5). Several C-IV type TRIM genes are present in the MHC locus of humans (35), chickens (36), ducks (37), and fish (38, 39). This suggests that TRIM genes were part of the ancestral MHC and have undergone duplication events in different species.

Many TRIM proteins are modulators of innate immunity and mediators of direct viral restriction. The repertoire of the mallard duck (*Anas platyrhynchos*) is of interest, as they are the reservoir host of influenza A viruses (40, 41). A comparison of the duck repertoire to chickens (*Gallus gallus*) is of value, since chickens are an important agricultural species and an established model for vertebrate development. Functional studies of individual TRIM proteins of birds demonstrate their importance in immunity. TRIM25 is functionally characterized in chicken (42–45), duck (46, 47), and goose (48). In mammals and ducks, TRIM25 catalyzes the addition of polyubiquitin for the activation of retinoic-acid-inducible gene I (RIG-I) in antiviral signaling (46, 49). Duck TRIM29 was identified as a negative regulator of the RIG-I signaling pathway (50). A gene described as TRIM39 in chicken is predominantly expressed in the spleen, but has not yet been functionally characterized (51). Chicken TRIM62 has been characterized (52) and shown to have antiviral activity against reticuloendotheliosis virus (53) and avian leukosis virus subgroup J (54). TRIM32, known for its antiviral activity in mammals (55–57), can restrict influenza (58) and Tembusu virus in ducks (59).

Here, we characterized the duck TRIM gene repertoire by utilizing NCBI databases and *de novo* transcriptome assembly to identify candidates. We investigated TRIM protein domain architecture and phylogenetic relationships. We compared the duck repertoire to the chicken, to look at species-specific differences. We performed phylogenetic analyses of the C-IV subfamily members of reptiles, birds, and mammals, which allowed us to designate orthologous TRIM genes in ducks. We show that ducks have 57 TRIM genes, compared to 54 in chickens. Ducks and other birds have TRIM genes specific to their respective lineages. Finally, we investigated both abundance and relative expression of these TRIM gene sequences in duck tissues. While some TRIM genes were ubiquitously expressed in duck tissues, other TRIM genes had tissue-specific expression.

## 2 Materials and methods

### 2.1 Data mining

To generate a master transcriptome of duck sequence reads, the NCBI Short Read Archive (SRA) database (https://www.ncbi.nlm.nih.gov/sra) was mined for projects involving domestic mallard ducks (*Anas platyrhynchos*). Wild mallard and Muscovy ducks (*Cairina moschata*) were excluded. Project numbers and individual samples included in this study are listed in **Supplementary File 1**. In addition to assembling a master duck transcriptome, a chicken (*Gallus gallus*) transcriptome was assembled using SRA project numbers listed in **Supplementary File 1**. SRA libraries were uploaded to the Digital Research Alliance of Canada’s research computing environment (formerly Compute Canada) (https://alliancecan.ca/en). SRA files with ambiguous descriptions, questionable content, or failed quality checks (i.e., more than 50% of reads were unpaired, or the files were corrupt) were excluded. To generate a reference dataset of all known avian TRIM sequences, we searched the NCBI protein databases for TRIM sequences. Redundant and misannotated sequences were removed. From the curated avian TRIM list, we made two additional databases, one composed of duck TRIM proteins and one composed of chicken TRIM proteins.

Members of the C-IV TRIM protein subfamily were also mined from NCBI to infer homology among TRIM proteins. We searched for each TRIM protein by name in mammals and generated a library of TRIM proteins from human, an additional placental mammal, and marsupial. For each avian TRIM protein, we ensured that there were at least three representative species. Reptile TRIM proteins were mined for a representative lizard species and a representative turtle species. C-IV protein accession numbers can be found in **Supplementary File 2**.

### 2.2 Transcriptome assembly

SRA libraries were checked for quality and adaptor content using astQC Version 0.11.9 (60). Samples used passed fastQC analysis and were between 100 and 150 bp in length. Reads were trimmed using Trimmomatic version 0.36 (61) with the following parameters: a sliding base window size of 4 (bases removed if phred score is below 20) in a 15-base window and a minimum read length of 33 bp.

A total of 216 duck libraries and 107 chicken libraries were assembled using 7 Kmer values from 25 to 85 using TRANS-ABYSS 2.0.1 (62). Individual libraries were assembled and binned by tissue type, and duplicate contigs within each tissue type were collapsed into a consensus contig using CAP3 (63) with a cutoff value of 95% identity and CD-HIT-EST version 4.8.1 (64) with a cutoff value of 97% identity. Tissue-type assemblies were collapsed into one master assembly using CAP3 assuming 95% similarity. Singlet files were merged separately to reduce loss of genes due to excessive reduction in putative duplicate contigs. Merged singlet contigs were then compared back to the master transcriptome for a final master assembly. Sequences <200 bp were pruned from the master assembly using in-house scripts published at https://github.com/rmpeery/dataProcessing. Two quality control measures were employed to assess the final assembly. We checked the number and average size of contigs using the abyss-fac command in ABYSS v 2.0.1 (62). To ensure that transcriptome collapsing was not impacting gene content, BUSCO version 3.0.2 (65) was used to determine common orthologous gene content. To remove duplicate copies of genes (putative orthologs remaining due to assembly strategy), we used reciprocal blast hits (RBH) and applied a leave-one-out method to remove contigs with ≥ 97% similarity (perl script published at https://github.com/rmpeery/dataProcessing).

### 2.3 Duck TRIM gene identification

The master duck and chicken assemblies were each translated into all six reading frames using EMBOSS 6.6.0 (66). Avian TRIM reference proteins were compiled into a BLAST+ database using the makeblastdb command in BLAST+ version 2.7.1 (67). We ran BLASTp (BLAST+) against the assembled duck transcriptome, and all hits were parsed from the master transcriptome using in-house scripts (published at https://github.com/rmpeery/dataProcessing). Our newly identified, putative avian TRIM proteins were aligned to the reference TRIM proteins, and neighbor-joining (NJ) phylogenetic trees were inferred using CLUSTAL OMEGA (68). We compared full-length transcriptome contigs to the assembled list of annotated duck TRIM proteins to both confirm identity and validate the master assembly. TRIM proteins detected by the BLAST search but were not present in ducks were aligned against the avian TRIM protein database to confirm identity. Other ambiguous hits were submitted to SMART (69) to verify protein domain composition. To identify TRIM genes missing in birds or ducks, TRIM sequences from various species were aligned using COBALT (https://www.ncbi.nlm.nih.gov/tools/cobalt/) and submitted to HMMER (v3.3.2) (HMMER.org) to search for these missing TRIMs in our master assembly. Hits were further analyzed using HMMER (70), UniProt (71), and SMART (69). Domain composition of amino acid sequences were verified using SMART. Duck TRIM genes found in this analysis are summarized in **Supplementary Table S1**, and nucleotide sequences used in downstream analyses can be found in **Supplementary File 3**. Duck TRIM amino acid sequences used in these analyses were derived from translating the nucleotide sequences and can be found in **Supplementary File 4**. All sequences used for the duck TRIM analysis resulted from interrogating the *de novo* transcriptome assembly, unless otherwise noted in **Supplementary Table S1**. Some sequences obtained from NCBI were used because the contigs pulled from the *de novo* transcriptome assembly appeared to be chimeric reads. Genes and proteins currently annotated in the genome and their corresponding accession numbers are compiled into **Supplementary Table S1**.

### 2.4 Comparison between duck and chicken TRIM protein repertoires

The newly identified duck TRIM nucleotide and protein sequences, generated from our *de novo* transcriptome assembly, were compared to chicken TRIM protein sequences from NCBI. Sequences present in the duck but presumed missing in the chicken were submitted to BLASTn and queried against the current chicken genome (version bGalGal1.mat.broilerGRCg7b, unpublished direct release) using the BLAST+ online portal (https://blast.ncbi.nlm.nih.gov/Blast). We used HMMER to further interrogate our chicken transcriptome for missing TRIM genes. TRIM genes found in the chicken transcriptome are available in **Supplementary File 5**. Annotations and identification numbers of these genes were compiled into **Supplementary Table S2**, and amino acid sequences were downloaded or added to (**Supplementary File 5**). Sequences were acquired from the annotations listed in **Supplementary Table S2** unless otherwise noted.

### 2.5 Mapping to chromosomes

To find chromosomal location of all duck TRIM genes, we queried nucleotide sequences against the current NCBI duck genome [assembly ZJU1.0, published (72)] using the online version of BLASTn. Chromosome lengths were taken from the reference duck genome, and locations were assigned from the start of the TRIM gene. Any genes not in the NCBI duck genome were submitted to blast against the Ensembl rapid release domestic *Anas platyrhnchos* genomes (GCA_017639305.1, GCA_015476345.1 and GCA_017639285.1). Genes present around any TRIM hits were compared back to the NCBI Pekin duck assembly to try to infer possible genomic locations. TRIM genes were assigned chromosomal location using karyoploteR (73) in the R studio environment (74). The resulting map was edited using Adobe Illustrator for clarity.

Approximate location and composition of MHC-linked TRIM genes were compared between duck, mouse (*Mus musculus*), human (*Homo sapiens*), Tasmanian devil (*Sarcophilus harrisii*), yellow pond turtle (*Maurenys mutica*), and Eastern fence lizard (*Sceloporus undulatas*) from data on NCBI. Genomic size and location of TRIM genes were extracted from reference genomes ZJU1.0 (Pekin duck), GRCm39 (mouse), GRCh38.p14 [human, published (75)], mSarHar1.11 [Tasmanian devil, published (76)], ASM2049712v1 (yellow pond turtle), and SceUnd_v1.1 [Eastern fence lizard, published (77)]. TRIM genes were assigned chromosomal location using karyoploteR (73) in the Rstudio environment (74). The resulting maps were edited graphically using Adobe Illustrator for clarity and readability.

Sizes and locations of TRIM genes located in the MHC-B locus of mallard ducks, tufted ducks (*Aythya fuligula*), chickens, kākāpō (*Stringops habroptilus*), barn swallow (*Hirundo rustica*), and European golden plover (*Pluvialis apricaria*) were approximated from genomic data on NCBI. Data were extracted from reference genomes ZJU1.0 (Pekin duck), bAytFul2.pri [tufted duck, published (78)], bGalGal1.mat.broiler.GRCg7b (chicken), bStrHab1.2.pri [kākāpō, published (79)], bHirRus1.pri.v2 (barn swallow, https://vertebrategenomesproject.org/), and pPluApr1.pri (European golden plover, https://vertebrategenomesproject.org/). The European golden plover genome is not yet annotated, so TRIM gene approximate locations were found using the NCBI blastn against the genome and coding genes predicted using GENSCAN (http://hollywood.mit.edu/GENSCAN.html). The resulting distances and approximations of gene sizes were edited using Adobe Illustrator.

Sizes and locations of TRIM genes located in the TRIM25 locus of duck, chicken, kākāpō, barn swallow, Adelie penguin (*Pygoscelis adeliae*), and Eastern fence lizard were approximated from genomic data on NCBI. Data were extracted from reference genomes ZJU1.0 (mallard duck), bGalGal1.mat.broiler.GRCg7b (chicken), bStrHab1.2.pri (kākāpō), bHirRus1.pri.v2 (barn swallow), ASM69910v1 (Adelie penguin), and SceUnd_v1.1 (Eastern fence lizard). The resulting distances and estimates of gene sizes were edited using Adobe Illustrator.

### 2.6 Phylogenetic trees and minimum spanning networks

We made two alignments of TRIM proteins, the first aligning all duck TRIM proteins and the second aligning both duck and chicken TRIM protein sequences. A third alignment was made using C-IV subfamily members from representative species. The species used were human, a representative placental mammal, marsupial, turtle, and lizard. Avian TRIM genes not found in chicken or duck were also added to our alignments. We mined two representative reptile species, the Eastern fence lizard and the yellow pond turtle, for MHC-linked TRIM proteins, by manually scanning the chromosomes containing MHC and collecting accession numbers for TRIM genes. Accession numbers for C-IV protein sequences used can be found in **Supplementary File 2**. TRIM proteins were aligned using the online MAFFT alignment program (80). Alignments used the L-INS-I refinement method and were further refined by eye in Unipro UGENE (81). The best protein model was determined for each tree using ModelFinder (82) in the IQTree environment. Maximum-likelihood (ML) phylogenetic trees of these alignments were inferred using IQTree (83) generating a tree for the duck TRIM protein; a tree for the duck and chicken TRIM proteins; a tree for all C-IV TRIM proteins in mammals, reptiles, and birds; and a tree for C-IV TRIM proteins in mammals, reptiles, and birds excluding the non-orthologous expansion of MHC-linked TRIM genes in reptiles. The Ultrafast bootstrap algorithm (84) was run with 10,000 bootstrap replications on the duck only and duck and chicken TRIM ML phylogenies. The Ultrafast bootstrap algorithm was run with 5,000 bootstrap replications on the C-IV TRIM protein subfamily trees. The consensus of these replicates was visualized in FigTree v1.4.3 (http://tree.bio.ed.ac.uk/software/figtree/). Phylogenetic trees were then further edited using Adobe Illustrator for clarity and to add additional information.

To make minimum spanning networks (MSN) with duck TRIM proteins, the duck TRIM protein alignment (as described above) was converted to a distance matrix using msa (85) and seqinr (86) in the Rstudio (v4.0) environment. A MSN was created using the prim algorithm provided by the RGBL r package (87) and visualized using ggplot2 (88).

### 2.7 Assigning names to duck TRIM genes

To resolve ambiguous names and incorrect NCBI annotations, we assigned names to TRIM genes based on homology, determined through phylogenies. As many avian TRIM genes did not have orthologs in mammals, we assigned these TRIM genes numbers starting at 200. The C-IV subfamily of TRIM proteins is expanded in vertebrates, and many of the putative TRIM proteins have redundant descriptions and names on NCBI. Many names of the C-IV TRIM proteins needed to be resolved through phylogenetic analysis comparing these proteins to TRIM proteins from other species. The new names and previously published annotations can be found in **Supplementary Table S1** for ducks and **Supplementary Table S2** for chickens.

### 2.8 Read mapping and differential expression of TRIM genes

Our newly identified TRIM contigs were pruned to CDS regions and were concatenated into a multi-FASTA file and used as the reference for read mapping. We aligned RNA-seq reads to the reference TRIM genes using Bowtie2 v2.3.4.3 (89). We used featureCounts to summarize, count reads, and assign features (90) from the BowTie2 mapped outputs. Reads were normalized to individual library sizes using the EdgeR (91) trimmed mean of M values (TMM) method. Reads were compared using plotMDS.DGElist in the Rstudio environment. Normalized log2 counts per million log2(CPM) were visualized using the cim function in R (92). Raw data of normalized read counts for each sample can be found in **Supplementary File 6**. We analyzed relative patterns of tissue distribution by setting the matrix intercept as the overall mean and using the generalized linear model quasi-likelihood test (glmQL) to determine log2 fold change (FC) values of individual tissues when compared to the mean in EdgeR. All log2(FC) values were visualized using a heatmap using the gplots heatmap.2 function (93) in the Rstudio environment. Dendrograms were added using heatmap.2 function in gplots in the Rstudio environment. All data for these experiments, including log2(FC) and false discovery rate (FDR), can be found in **Supplementary File 7**. To determine the highest and lowest relatively expressed TRIM genes in different duck tissues, we sorted the relative expression results for each tissue by false discovery rate FDR (<0.05) and filtered out any TRIM genes above this threshold. We then sorted these data by log2(FC) and summarized the top 5 highest and top 5 lowest relatively expressed genes.

## 3 Results

### 3.1 Ducks have 57 genes in their TRIM repertoire

To determine how many TRIM genes are present in the domestic mallard duck (*Anas platyrhynchos*), we first mined the NCBI genome and protein databases for all annotated avian TRIM proteins. From this list, we identified TRIM proteins annotated in the duck and chicken. We made a *de novo* transcriptome assembly for ducks using 216 SRA libraries. We used both BLAST+ (67) and HMMER (hmmer.org) searches of our transcriptome to identify 57 TRIM genes in the duck. We used a neighbor-joining tree to cluster putative TRIM contigs with known avian TRIM proteins to verify identity and identify outliers. A flow chart of the workflow can be found in **Supplementary Figure 1**. Genes that were found to be one to one orthologs of human TRIM genes were identified and named to reflect that ancestry. Duck TRIM protein annotations were assembled into a table (**Supplementary Table S1**). All but four of the TRIM sequences that we identified were previously annotated on NCBI. TRIM28 and fibronectin III and SPRY domain containing proteins 1 (FSD1) are annotated in the rapid release Ensembl duck genomes (TRIM28 in GCA_017639305.1 and FSD1 in both GCA_017639305.1 and GCA_017639285.1). TRIM39 and RING finger protein 39 (RNF39) are not yet annotated in any duck genome.

We included 11 genes classified as TRIM-like in this analysis, in that they do not have the traditional “tripartite motif” domain structure that the TRIM family is named for. Some of these, such as RNF207, FSD1 and FSD2, B-box and SPRY domain containing (BSPRY), and NHL repeat containing E3 ubiquitin protein ligase 1 (NHLRC1) are ancestral TRIM genes, which have lost domains during speciation events (8). RNF135, with the protein referred to as RIPLET, contains a RING, CC, and PRY-SPRY domains, and is lacking B-box domains. These TRIM-like genes will be included in our analyses of the duck TRIM repertoire. Sequences discovered to be butyrophilin (BTN) proteins were omitted, although we acknowledge that many BTN proteins are highly similar in C-terminal domain composition to C-IV subfamily TRIM proteins, and thus, TRIM proteins and BTN proteins may share evolutionary ancestry.

Many TRIM genes identified did not appear to be direct orthologs to annotated mammalian TRIM genes and were renamed. Several non-orthologous TRIM genes are located in the MHC region of the duck. Additionally, NCBI annotations suggested ancestry to mammalian TRIM genes that our downstream analysis determined to be inaccurate. We renamed these genes TRIM200–213 to reflect their distinct sequences. For example, two genes (NCBI gene ID 101805457 and 101804875) were both described as TRIM39-Like on NCBI. Gene 101805457 was previously annotated as TRIM39.2 by Blaine and colleagues (37). Gene 101804875 was described as TRIM39-Like in ducks (NCBI) and TRIM39 in chickens (51). However, neither of these genes are an ortholog of TRIM39, so we amended the names to TRIM202 and TRIM212 (for gene 101805457 and 101804875, respectively). Our transcriptome interrogation did find a direct TRIM39 ortholog in the duck that is not present or annotated in Ensembl or NCBI genomes. Likewise, Gene ID 119718713 was annotated as RNF39-like in the NCBI duck genome. This gene does not appear to be orthologous to mammalian RNF39, so we renamed it TRIM200. Ducks do have an ortholog of RNF39; however, this gene is only present in the duck transcriptome and not in genomic resources. **Supplementary Table S1** indicates the duck TRIM genes with direct mammalian orthologs and the genes without clear mammalian orthologs that we renamed, and we will use these assigned names throughout.

### 3.2 The duck TRIM gene repertoire spans 21 chromosomes

**Figure 1:**
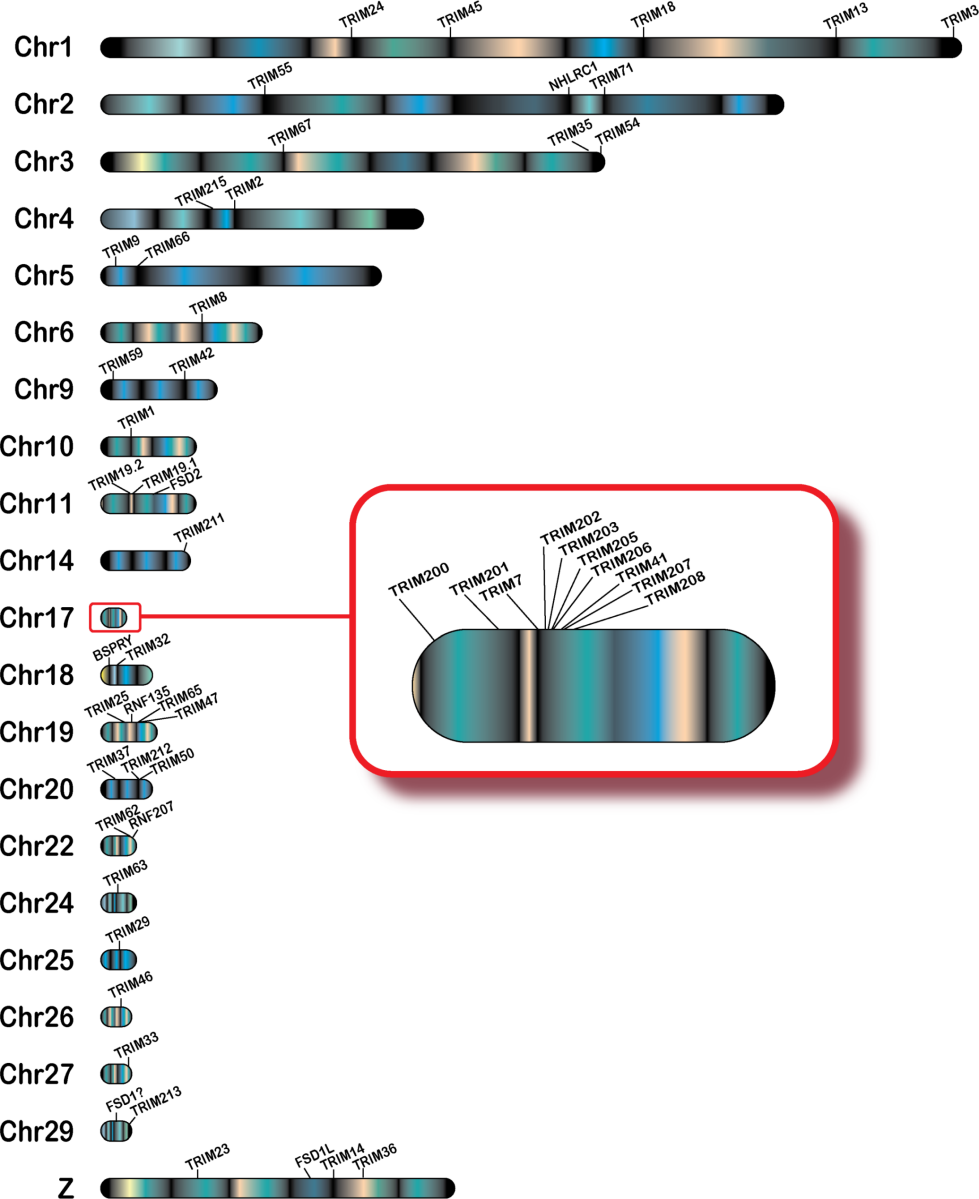
Genomic locations of TRIM or TRIM-like genes in the duck. TRIM and TRIM-like genes were submitted to NCBI blast, and locations in the duck genome were mapped using karyoploteR in the R studio environment. Chromosome 17 is magnified to allow for visualization of the expansion of the TRIM genes in the duck MHC (B) locus. The gene FSD1 is followed by a"?" to reflect a presumed location due to data available in other genomic resources. The cluster of related genes on TRIM25 will be referred to as the "TRIM25 locus.

To determine chromosomal locations of the 57 domestic mallard duck TRIM genes, we interrogated the Pekin duck genome (assembly ZJU1.0) using our newly generated TRIM genes. We assigned chromosomal locations to 54 of 57 TRIM genes, on 21 different duck chromosomes (**Figure 1**). Most duck TRIM genes are present in unique locations throughout the genome. However, two interesting clusters of genes are found on chromosomes 17 and 19. Ducks also have a duplication of promyelocytic leukemia protein (TRIM19/PML), resulting in two similar genes on chromosome 11 in opposite orientation.

A cluster of TRIM genes are found in the duck MHC region on chromosome 17 (**Figure 1**, inset). We placed 10 TRIM or TRIM-like genes on chromosome 17, with nine of these found in the duck MHC region. All MHC-linked TRIM or TRIM-like proteins have C-terminal PRY-SPRY domains (**Supplementary Table S1**). We previously compared this region in ducks to the syntenic region in chickens and turkeys (37). The newest assembly of the Pekin duck genome allows TRIM207, another of the MHC-linked TRIMs, to be placed in this region. This gene was defined as a BTN in chicken (94); however, our analysis suggests that it is a TRIM-like gene. These data support the expansion of a group of related TRIM genes in the MHC region of birds.

A smaller cluster of TRIM genes is located on chromosome 19. The TRIM-like gene RNF135 is located on between TRIM25 and TRIM65. We will refer to this location as the TRIM25 locus. This region also includes a related gene, TRIM47. Another nucleotide sequence distantly related to TRIM25 is found on chromosome 14, which we have named TRIM211. However, the duck TRIM211 gene had premature stop codons in the sequence, suggesting that it may be a pseudogene. Indeed, its annotation on NCBI includes it in the 3′ untranslated region of a separate gene, described as *PPARGC1B* (GeneID: 101790707). Whether this is due to mistakes in genome assembly/sequencing or gene fusion resulting in loss of function is unknown.

We identified TRIM28, TRIM39, RNF39R, and FSD1 within the duck transcriptome data but were unable to map them to chromosomes on the NCBI duck genome. TRIM28 is present in one and FSD1 is present in two of the Ensembl rapid release genomes (GCA_017639305.1 and GCA_017639285.1); however, neither of these genomes are chromosome level assemblies. In the Ensembl rapid release genomes, FSD1 is located in between the genes SH3GL1 and YJU1. Both these genes appear on chromosome 29 in the NCBI duck genome, suggesting that FSD1 is located on chromosome 29, and this chromosome is currently misassembled in the NCBI Pekin duck genome. We were unable to find TRIM39 or RNF39 in either the NCBI or Ensembl rapid release duck genomes. The current NCBI Pekin duck genome assembly has 33 chromosomes in addition to the sex chromosomes (72), and karyotyping shows 2N=40 for ducks (72, 95), suggesting that many chromosomes may still be unassembled.

### 3.3 Chicken and duck TRIM homologues split into two distinct clades

To compare the chicken and duck TRIM gene families, we searched for chicken orthologs for our 57 duck TRIM protein sequences using NCBI BLASTp. This search yielded 52 chicken TRIM or TRIM-like proteins (**Supplementary Table S2**). We created a *de novo* transcriptome assembly for chickens using 107 SRA RNA-seq libraries, and our interrogation of the transcriptome found RNF39R, TRIM39, and TRIM46, which were not present in the chicken genomes on NCBI or Ensembl rapid release. While many chicken TRIM proteins shared high percent identities to duck TRIM proteins (>90% identity), there were some exceptions. The TRIM19 paralogs were divergent at 75% and 55% identity, for TRIM19.1 and TRIM19.2, respectively. TRIM25 (71% identity), TRIM47 (81% identity), and TRIM65 (69% identity) were also divergent. Many of the MHC-linked TRIM proteins have also diverged between ducks and chickens, except TRIM201, which shares 97% amino acid identity. Both TRIM39 and RNF39R are significantly different between duck and chicken with 60.08% and 48.92% amino acid identity, respectively. However, the latter genes are only present in the duck or chicken transcriptomes and not yet confirmed in their genomes, so misassembly could contribute to sequence differences.

**Figure 2:**
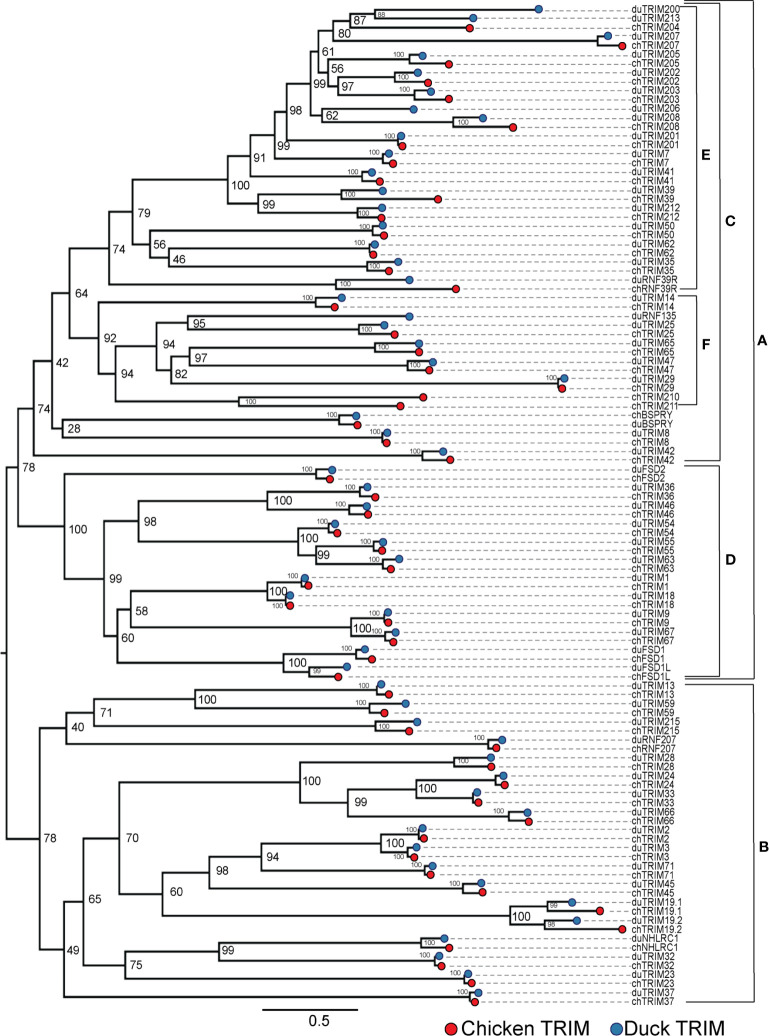
Most duck TRIM proteins have chicken orthologs. Phylogenetic relationships between the duck and chicken TRIM protein sequences were investigated using maximum likelihood (ML) trees with 10,000 Ultrafast bootstrap replications. External nodes were color-coded to indicate species of origin for each TRIM protein. Clades A and B represent the two major subclades. Clade C represents the C-IV subfamily. Clade D represents the C-I and C-II subfamily. Clade E includes the MHC-linked TRIM proteins. Clade F represents genes present in the TRIM25 locus and related genes located elsewhere, referred to as the "TRIM25 cluster". Initial ML tree was referred using. IQTree and visualized using FigTree. The resulting tree was edited for clarity using Adobe Illustrator.

To compare the relationships of TRIM proteins within the duck and chicken repertoires, we aligned all duck and chicken amino acid sequences, then built an ML tree using 10,000 Ultrafast bootstrap replications. The sequences divide into two major clades (clades A and B) (**Figure 2**). In clade A, there are two subclades (clades C and D), clade D containing C-I and C-II TRIM proteins and clade C containing C-IV proteins. C-I subfamily members first arose in animals (8) and are defined as having both COS and FN3 domains and only a SPRY or both PRY and SPRY C-terminal domains. SPRY domains are present in animals, fungi, and plants, while PRY domains arose during vertebrate evolution (33). Some members of the C-I subfamily have both PRY-SPRY domains, while the more ancient members have only SPRY domains. TRIM9 is present in animals, including invertebrates, and has a C-terminal SPRY domain but not PRY domain in shrimp (96, 97), human (98), and ducks (**Supplementary Table S1**). The C-IV TRIM proteins, which expanded in vertebrates (8, 9) and have both PRY and SPRY C-terminal domains, split into two distinct subclades (clades E and F), clade E containing the MHC-linked TRIM proteins and clade F containing genes which cluster with members of the TRIM25 locus, to be referred to as the "TRIM25 cluster". While this suggests that the C-IV subfamily diverged from a common ancestor of the C-I subfamily, the bootstrap values are too low to support this hypothesis. In clade B, most duck proteins have direct orthologs with chicken on short branches. The duck and chicken TRIM19/PML paralogs (TRIM19.1 and TRIM19.2) appear distant from their orthologous counterparts. This could be due to significant divergence in sequences or misassembly of these sequences. Many bootstrap values were too low to resolve distant ancestry of these TRIM proteins, for example the common ancestors of clades A and B, suggesting that further analysis using more taxa is needed.

We found some TRIM proteins only in chicken or only in duck. The TRIM-like gene RNF135 has previously been described as absent in chicken (99), quail (100), penguins, falcons, and petrels (101). We also did not find RNF135 in the chicken transcriptome. Two TRIM proteins found in chicken were without mammalian orthologs, so we named them TRIM210 and TRIM211. Phylogenetic analysis places TRIM210 and TRIM211 within clade F with the TRIM25 cluster, a group including RNF135, TRIM14, 25, and 65, known for RIG-I-like receptors (RLR) pathway modification (102–104). Ducks are missing TRIM210, and TRIM211 is likely a pseudogene, while the genes are intact and adjacent in the chicken genome. A closer inspection of the syntenic region on duck chromosome 14 revealed a deletion in that section. The deletion could be due to genomic misassembly; however, HMMER searches of our duck transcriptome did not find any contigs matching TRIM210. Duck and chicken TRIM29 also clusters within the clade containing the RLR pathway modifiers.

There were four TRIM genes that we did not find in chickens that we found in ducks: TRIM200, RNF135, TRIM213, and TRIM206. We did not find these genes in our chicken transcriptome. We previously noted TRIM206 to be absent in chickens and turkeys (formerly named TRIM27L) (37). We believe that TRIM213 is missing due to deletions in the chicken, from our analysis of the syntenic regions of the chromosome.

### 3.4 Subfamily classification of human, duck, and chicken TRIM proteins

**Figure 3:**
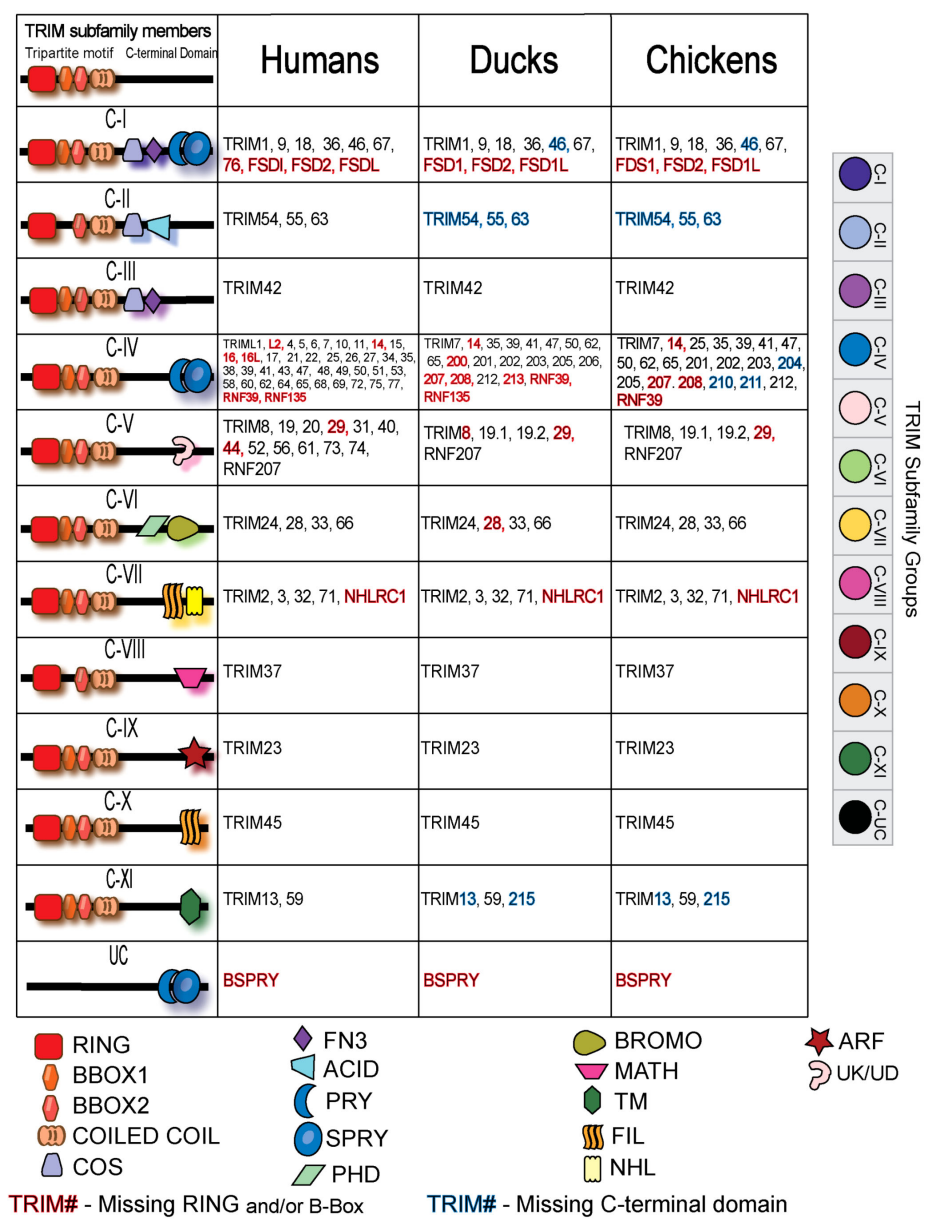
Classification of TRIM proteins by their C-terminal domains in human, duck, and chicken. RING, really interesting new gene; COS, C-terminal subgroup one signature; FN3, fibronectin, type III; SPRY, SPla, and Ryanodine receptor domain; PHD, Plant Homeo Domain; BROMO, Bromodomian; MATH, meprin and TRAF homology domain; TM, transmembrane domain; FIL, filamin domain; NHL, NCL-1, HT2A, and Lin-41 repeats; ARF, ADP ribosylation factor-like; UC, uncharacterized.

To compare the duck TRIM repertoire to human and chicken, we grouped the subfamilies of TRIMs by the standard C-terminal domain nomenclature (5, 31, 32). Many TRIM proteins are shared between humans, chickens, and ducks, but there are some repertoire differences between species (**Figure 3**). All TRIM subfamilies are represented in the duck and chicken TRIM proteins, with most groups containing clear orthologs. Domain composition from duck TRIM proteins was analyzed using SMART, and missing domains are indicated by the color of the protein name. Additional information on duck TRIM domain composition can be found in **Supplementary Table S1**.

The C-IV subfamilies of birds and humans not only share presumed orthologs but also have many unique proteins. Humans have a cluster of MHC-linked TRIMs not present in birds including TRIM31, 40 15, 26, and 38 (105). Human C-IV family TRIM genes, including TRIM5, 6, 22, and 34, appears absent from avian genomes. Humans and ducks have RNF135, which belongs in the C-IV subfamily, and it is missing in chickens. Chickens, however, have additional members of the TRIM25 lineage as they have TRIM210 and TRIM211.

Several members of the C-V family with an uncharacterized C-terminal domain are not present in birds; however, both duck and chickens have two paralogs of TRIM19, co-orthologs to mammalian TRIM19. Ducks and chickens have an additional member of the C-XI family, without mammalian homologues, thus named TRIM215. The C-XI subfamily is classified by a C-terminal transmembrane domain; however, both duck TRIM215 and TRIM13 appear to be missing this domain (**Supplementary Table S1**). TRIM215 appears distantly related to TRIM59 and TRIM13. The group C-II orthologs are missing the C-terminal domains for which they are classified in humans. Duck TRIM54, 55, and 63 appear to be missing the COS and acid domains that their human orthologous counterparts have.

### 3.5 Birds, reptiles, and mammals have independent expansions of MHC-linked TRIM genes

**Figure 4:**
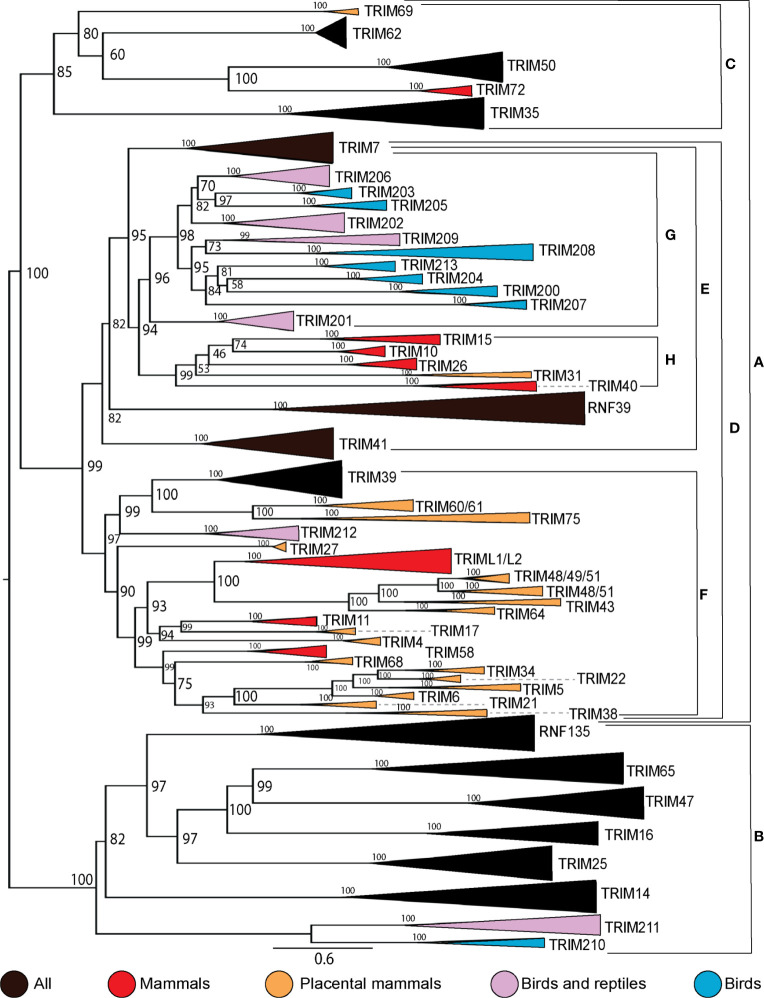
TRIM protein C-IV subfamily has direct orthologs among species and unique expansions within species. Phylogenetic relationships between C-IV subfamily members from human, non-primate placental mammal, marsupial, bird, lizard, and turtle protein sequences were investigated using maximum likelihood (ML) tree with 5,000 Ultrafast bootstrap replications. External nodes were combined when direct homology was inferred between species. Clades A and B represent the two major subclades. Clades C and D represent the two subclades within clade A. Clade E represents the location of MHC-linked TRIM genes, and clades G and H represent the separation of diapsid and mammalian MHC-linked TRIM proteins (respectively). Clade F represents the expansion of mammalian C-IV TRIM proteins. Initial ML tree was inferred using IQTree and visualized using FigTree. The resulting tree was edited for clarity using Adobe Illustrator.

To examine the expansion within the C-IV subfamily in higher-order vertebrates, we assembled protein sequences from human, mouse (or other placental mammal if mouse TRIM protein was non-existent), marsupial, three birds, and two reptiles from the NCBI protein database. We aligned these sequences and inferred a ML phylogenetic tree with 5000 UltraFast bootstrap replications (**Supplementary File 8**). This tree shows both turtles and lizards appear to have independent expansions of C-IV TRIM genes in the MHC region, which were excluded from subsequent analysis. To assess shared and unique TRIM proteins between species, we created a phylogenetic tree of C-IV subtype TRIM proteins from representative mammalian, reptile, and avian species (**Figure 4**). We combined external nodes when all members of the clade were clear orthologs and color-coded these branches by the group of higher-order vertebrates. Many TRIM C-IV subfamily genes are conserved in all vertebrate species, while some are present in only mammals and some unique to birds and/or reptiles. The C-IV subfamily broadly splits into two clades (clades A and B). Clade A splits into two subclades (clades B and C), and clade C contains some highly conserved TRIM proteins, such as TRIM35, 50, and 63. Within clade A is the nested clade D, which contains the MHC-linked TRIM genes from vertebrates; however, these MHC-linked TRIM genes cluster separately between diapsids (clade G) and mammals (clade H). The human MHC-linked TRIM proteins, TRIM10, 15, 26, and 40, cluster closely with orthologs from both mouse and marsupial but not with any TRIM proteins from birds or reptiles.

Our results suggest lineage-specific expansions of TRIM genes in higher-order vertebrates and that the expansions of avian and mammalian MHC-linked genes diverged from an ancestor of TRIM7. The cluster of MHC-linked TRIM genes in mammals (clade H) appears to have expanded from a common ancestor shared with TRIM7. Many of the avian MHC-linked TRIM genes (clade G) expanded from a common ancestor shared with TRIM201, found in diapsids but not mammals. Our analysis also suggests that previous annotations of the avian MHC-linked TRIM genes were incorrect. Genes that we have assigned names TRIM203, 205, and 206 were previously designated as TRIM27.2, 27.1, and 27L, respectively (37). Our results demonstrate that these genes are not orthologous to human TRIM27. TRIM202 and TRIM204 were called TRIM39.2 and TRIM39.1, respectively. TRIM202 and TRIM204 exist in both birds and reptiles and appear to be missing in mammals. TRIM202 has been published under the name TRIM39 in chickens (51); however, we found a gene in the chicken and duck transcriptomes, which clusters with TRIM39 from other species. TRIM209 is in clade G with the avian MHC-linked TRIM genes and appears in both lizards and turtles. A single avian ortholog was found in the Kiwi (*Apteryx rowi*), but we were unable to locate an ortholog of this gene in other birds. TRIM213 is not located in the MHC region of ducks but clusters tightly with the other avian MHC-linked TRIM genes. Our results suggest that the diapsid TRIM genes in clade G are distant paralogs, expanding from a common ancestor shared with TRIM201, while the mammalian TRIM genes in clade H are distant paralogs originating from a common ancestor shared with TRIM7. These results suggest the diapsid and mammalian MHC-linked genes located in clades G and H are not co-orthologs but arose from distinct lineage-specific duplications. Reptiles appear to have lineage-specific duplications of TRIM genes in the MHC without orthologs in other vertebrates. We found 67 TRIM genes adjacent to the MHC class I genes in the eastern fence lizard and 228 in the yellow pond turtle. Many of these turtle and lizard TRIM genes appear not to have orthologous genes in birds or mammals and appear to be inparalogs within their lineages. It appears that independent duplications and diversification of the MHC-linked TRIM genes have happened often in reptilian, avian, and mammalian lineages.

Mammals have a large expansion of TRIM proteins in clade F. Many of these TRIM proteins are known to have expanded in eutherian mammals such as the expansion of the TRIM5/6/22/34 locus (15, 106, 107). Ducks have two TRIM proteins in this minor clade, TRIM39 and TRIM212. TRIM212 appears to be a result of lineage-specific duplication, as it appears present in diapsids only.

Clade B contains the TRIM25 cluster, C-IV TRIM proteins present in all animals surveyed, including TRIM14/16/25/47/65 and RNF135. This clade also includes TRIM210, which we could only find examples of in birds and TRIM211, which is present in birds and reptiles but not in mammals. This clade contains a TRIM protein found only in reptiles, which we have named TRIM214 (**Supplementary File S8**) and shares ancestry with TRIM47 and 65. Notably, RNF135 appears absent in species of bird, which also appear to be missing RIG-I (101) but is found in other birds, reptiles, and mammals. TRIM16 appears absent in chicken and duck but is present in other birds, reptiles, and mammals. This TRIM25 cluster has remained conserved throughout vertebrate evolution, with additional TRIM genes in birds and reptiles.

### 3.6 The MHC-linked TRIM gene repertoire of birds, reptiles, and mammals has dramatically changed over time

**Figure 5:**
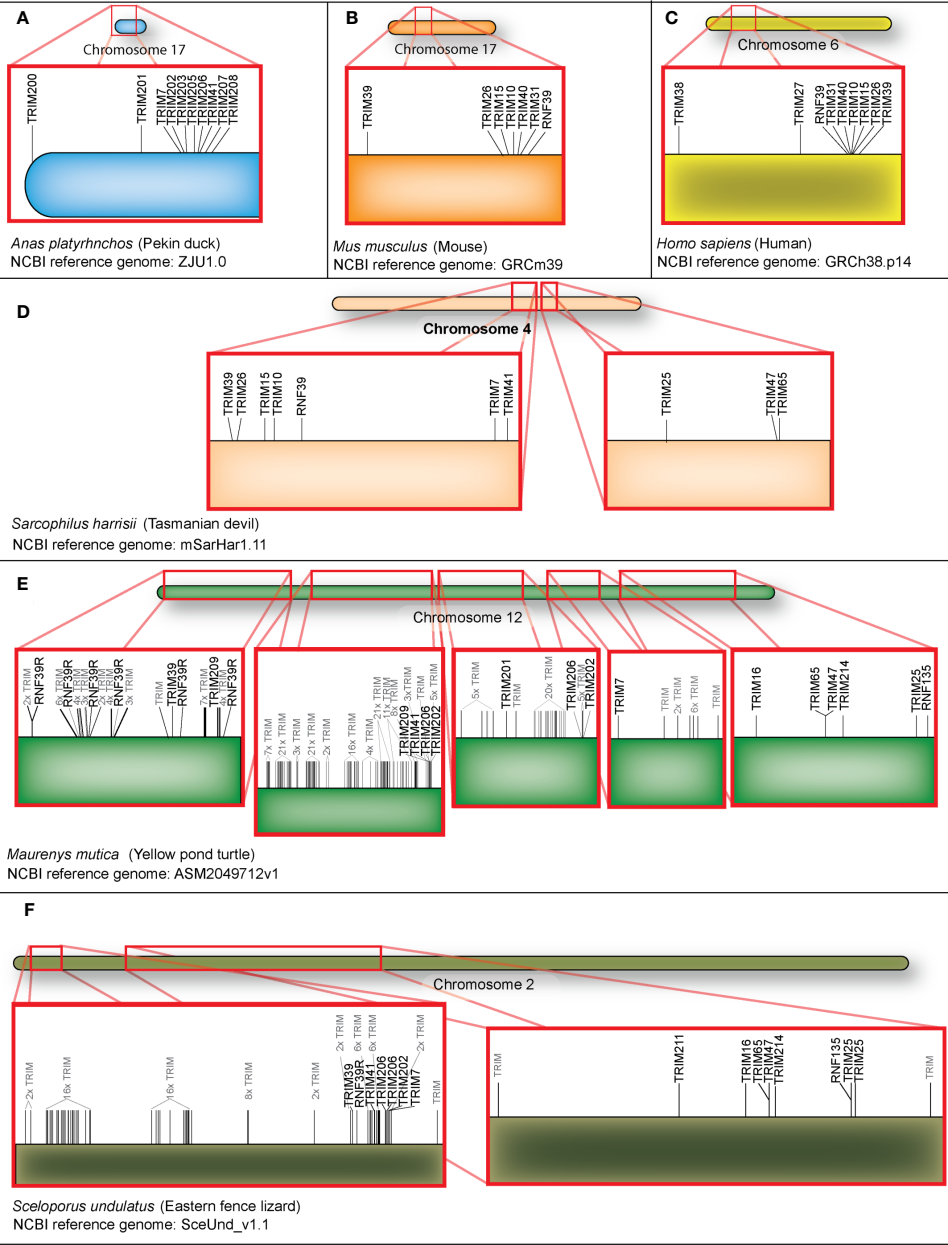
Orthologs of TRIM7, TRIM39, TRIM41 and RNF39 are present in all orders, but have been relocated from the MHC of humans and mice. MHC-linked TRIM genes from duck **(A)**, mouse **(B)**, human **(C)**, Tasmanian devil **(D)**, yellow pond turtle **(E)**, and Eastern fence lizard **(F)** locations were mapped using karyoploteR in the R studio environment. TRIM genes without distinct homologs or names are shaded in gray.

To explore the evolution of TRIM genes in the MHC region, we generated maps of chromosomal locations of MHC-linked TRIM genes for representative vertebrate species (**Figure 5**), including duck, mouse, human, Tasmanian devil, yellow pond turtle, and Eastern fence lizard. The duck MHC-linked TRIM genes include orthologs of mammalian TRIM7 and TRIM41, and the diapsid lineage-specific TRIM200, 201, 202, 203, 205, 206, 207, and 208 (**Figure 5A**). MHC-linked TRIM genes show orthology between humans and mice. Mice have TRIM39, 26, 15, 10, 40, and 31, and RNF39 in the MHC region (**Figure 5B**), which humans share (**Figure 5C**). As previously reported, humans have TRIM38 and 27 telomeric to the TRIM cluster (35). To ascertain which MHC-linked TRIM genes predate eutherian mammal speciation, we examined the MHC region of a marsupial and two reptiles. Marsupials are an ancient mammal, diverging from eutherian mammals approximately 160 million years ago (108). Tasmanian devils have orthologs to MHC-linked TRIM genes present in either placental mammals or duck. Like humans and mice, Tasmanian devils have TRIM39, 26, 15, and 10, and RNF39 (**Figure 5D**). Like ducks, Tasmanian devil MHC-linked TRIM genes also include TRIM7 and 41, suggesting that these genes were lost from the mammalian MHC region during eutherian speciation. Different from birds and placental mammals, TRIM25, 47, and 65 are also located adjacent to the MHC-linked orthologous TRIM genes on Tasmanian devil chromosome 4. To see if this is an ancestral organization, we compared the Tasmanian devil to reptile MHC regions. Recent phylogenetic analysis suggests that turtles share a clade with birds and crocodiles, while lizards form a separate clade (109). We found a large expansion of 226 TRIM genes in the MHC region of the yellow pond turtle (**Figure 5E**) (**Supplementary File S2**). Many of these TRIM genes do not share obvious orthology to mammalian or avian TRIM genes. Like mammalian MHC-linked TRIM genes, the yellow pond turtle has RNF39 and TRIM39 in the MHC locus. Turtles appear to have an expansion of RNF39 co-orthologs, composed of six RNF39R genes. Some genes orthologous to avian MHC-linked TRIM genes also appear in turtles, including two TRIM206 genes, TRIM7, 41, 201, and 202. TRIM209, a TRIM gene that appears in reptiles and kiwi birds, is also in this region. Like the Tasmanian devil, the TRIM25 locus is adjacent to MHC-linked TRIM genes in turtles. We found 77 TRIM genes in the MHC locus and the TRIM25 locus in the Eastern fence lizard (**Figure 5F**). The Eastern fence lizard, more distantly related to birds than the yellow pond turtle, has two paralogs of TRIM206 and an ortholog of TRIM201, suggesting that TRIM201 and 206 are ancestral to the other MHC TRIM genes of birds. Like the yellow pond turtle and Tasmanian devil, the Eastern fence lizard MHC-linked TRIM repertoire also contains RNF39, TRIM7, 39, and 41 orthologs. Similar to the Tasmanian devil and yellow pond turtle, the Eastern fence lizard TRIM25 locus is adjacent to the MHC-linked TRIM genes. TRIM211 and TRIM214, both TRIM proteins that clustered with TRIM25, 47, and 65, are in this region in lizard. There were more TRIM genes downstream of the TRIM25 locus in the Eastern fence lizard; however, we decided to limit this comparison to the MHC and TRIM25 loci. Our results suggest the TRIM25 locus may have evolved alongside the MHC locus in lower vertebrates. As this is conserved between marsupials and reptiles, it suggests that the TRIM25 locus moved away from the MHC independently in avian and placental mammals. Our results suggest that RNF39, TRIM7, 39, and 41 are ancestral MHC-linked TRIM genes with conserved orthologs in reptiles, birds, and mammals.

**Figure 6:**
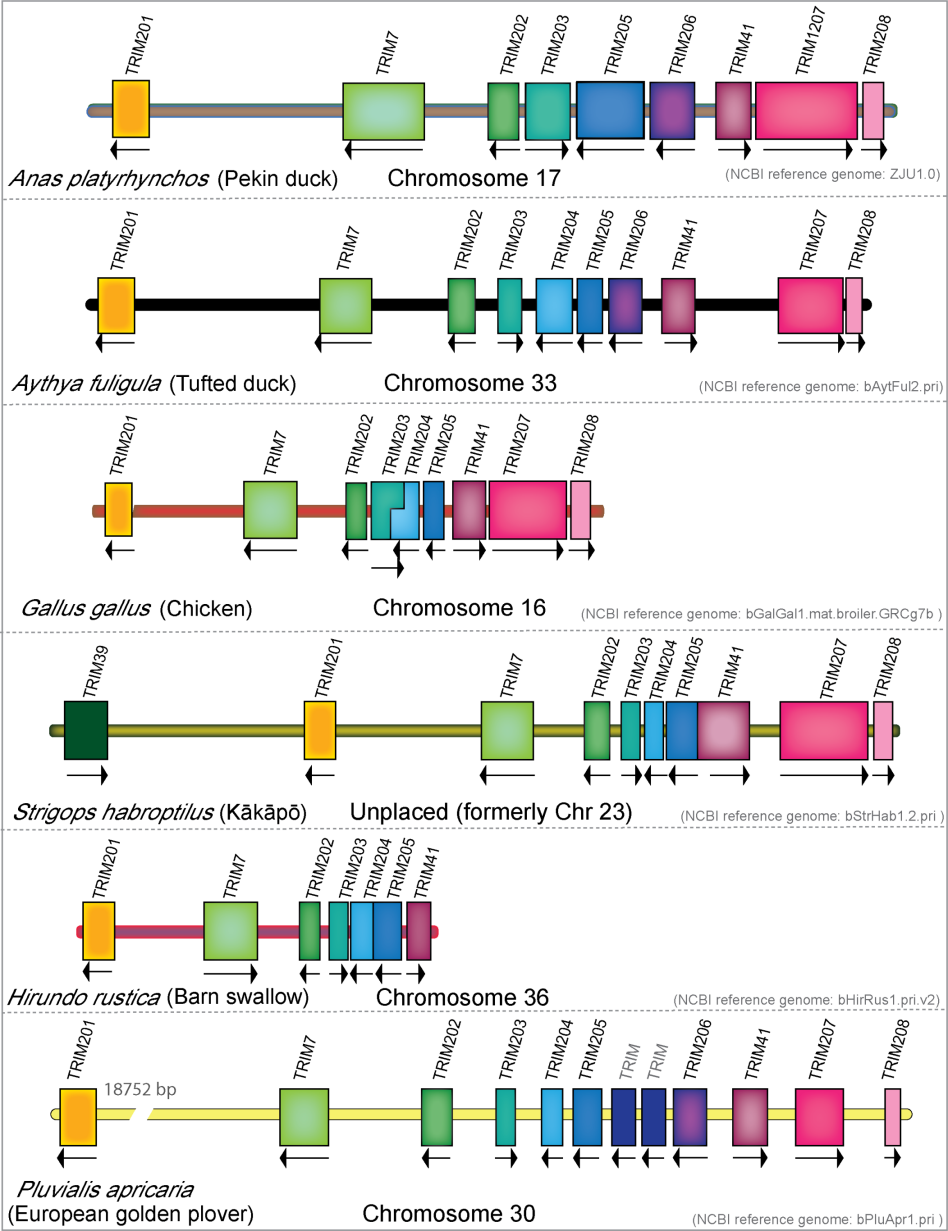
MHC-linked TRIM gene repertoire in birds has expanded and contracted during avian evolution. Genomic locations and direction of transcription of the MHC-linked TRIM genes from mallard duck, tufted duck, chicken, kākāpō, barn swallow, and European golden plover. All sizes of genes and chromosome length were normalized to the size of mallard duck gene TRIM208. Break shows gap of indicated size.

To investigate differences in the MHC-linked TRIM genes within the avian lineage, we compared the genomic arrangement of the MHC-linked TRIM genes of mallard duck, tufted duck, chicken, kākāpō, barn swallow, and European golden plover (**Figure 6**). Tufted duck and chicken both contain TRIM204, while this gene appears to be missing in mallard duck. We can find the corresponding sequence of TRIM204 in the 5′-UTR of TRIM205 in the mallard duck, which suggests a misassembly. However, there are no detectable transcripts of this gene expressed in any mallard tissues, indicating that it may no longer be expressed in mallard ducks. Both species of mallard ducks have TRIM206, which is missing in chicken, kākāpō, and barn swallow. Barn swallows have lost TRIM206 and two TRIM genes downstream of TRIM41. We observe a species-specific expansion of the MHC-linked TRIM genes in the European golden plover between TRIM205 and 206, with two to three additional predicted TRIM-like genes. These TRIM genes are closely related paralogs and do not appear to have direct orthologs in birds or reptiles. Phylogenetic analysis of these paralogs found the golden plover TRIM genes cluster in a clade with TRIM203 and TRIM205 and not with any of the reptile-specific MHC-linked TRIM genes (data not shown), suggesting that this may be a lineage-specific expansion. As the current golden plover genome is not annotated and has no accompanying transcriptome data, it is unknown how many of these genes are expressed. Our analysis of the MHC-linked TRIM genes in birds demonstrates these genes are continuously changing over time.

### 3.7 The TRIM25 locus has undergone gene loss and gain during diapsid evolution

**Figure 7:**
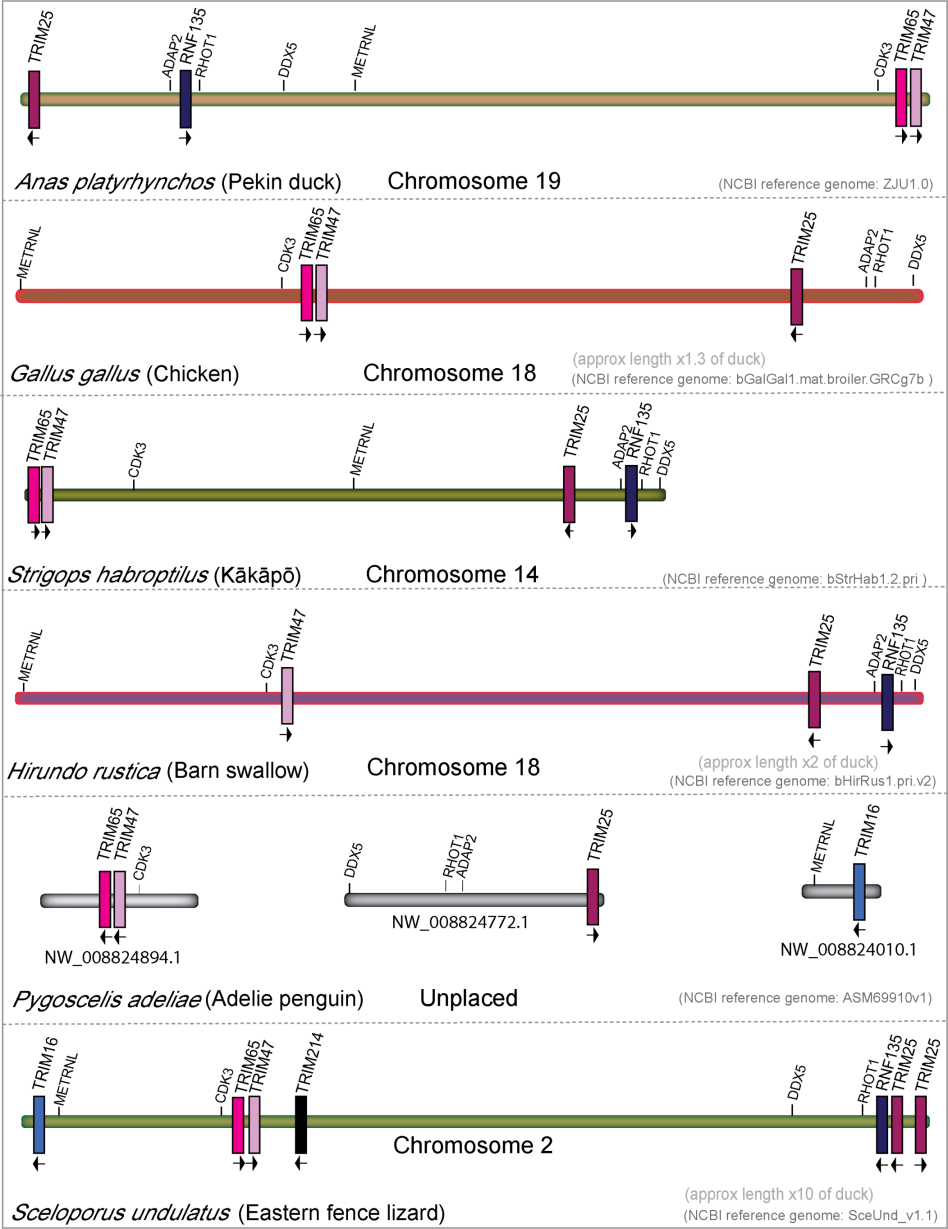
The TRIM25 locus has undergone rearrangement during diapsid evolution. Genomic locations and direction of transcription of TRIM genes in the TRIM25 locus from mallard duck, chicken, kākāpō, barn swallow, Adelie penguin, and Eastern fence lizard. All chromosome lengths were normalized to the size of the total TRIM25 locus length in mallard duck.

The TRIM25 locus has gone through significant rearrangements during avian evolution (**Figure 7**). In the duck, genes are arranged TRIM25/RNF135/TRIM65/TRIM47, while in the chicken, they appear in the gene order of TRIM65/47/25. RNF135 was noted as missing in chickens (99, 110), and we could not find it in our chicken transcriptome interrogation. The inactivation of chicken RNF135 appears to be independent of the chromosomal rearrangement, as only RNF135 is missing, while the surrounding genes ADAP2 and RHOT1 are still present. Like chickens, Adelie penguins appear to be missing RNF135; however, RHOT1 and ADAP2 are still present and near each other. Interestingly, Adelie penguins have an ortholog of TRIM16, which only appears in penguin, ostrich, kiwi, and hoatzin genomes (NCBI). A TRIM16 ortholog is also present in lizard and turtle genomes, suggesting that many birds, including both chickens and ducks, may have lost TRIM16. Barn swallows appear to be missing TRIM65, which is present in all other birds that we surveyed in this study, and in reptiles. Reptiles have a novel TRIM gene in the TRIM25 locus, which we have named TRIM214, as it has no clear ortholog in mammals. In addition, Eastern fence lizards appear to have a duplication of TRIM25. The TRIM25 locus appears to have undergone significant rearrangement in both repertoire and gene placement throughout avian evolution.

### 3.8 Duck TRIM proteins cluster within TRIM subfamilies

**Figure 8:**
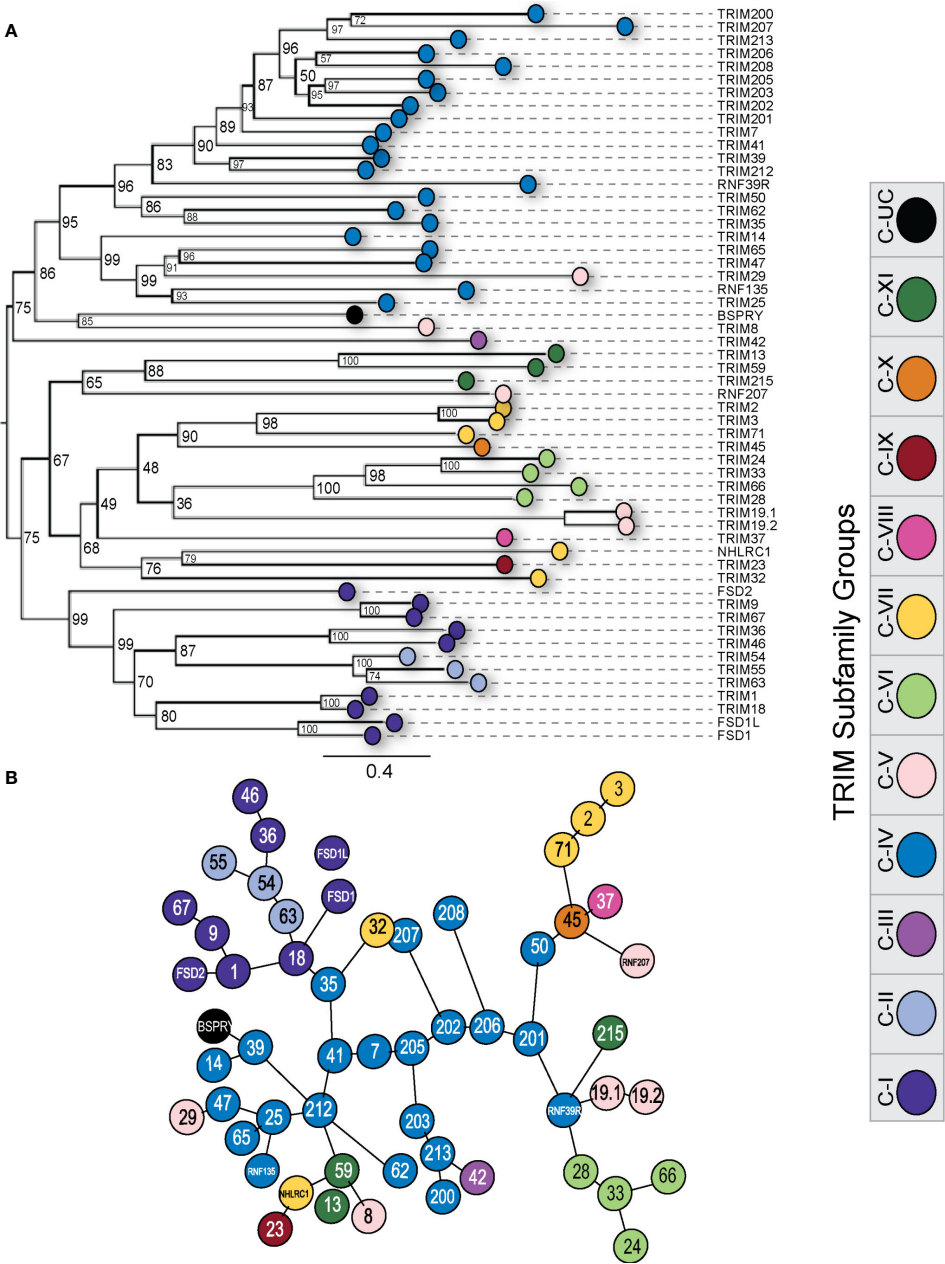
Duck TRIM and TRIM-like proteins cluster within subfamilies. Phylogenetic relationships between the duck TRIM protein sequences were investigated using maximum likelihood (ML) trees with 10,000 Ultrafast bootstrap replications **(A)**. The distances between protein sequences were also investigated using a minimum spanning network to infer similarity and function **(B)**. Each external node was color-coded according to TRIM subfamily designation. ML tree was made using IQTree, visualized using FigTree, and edited for clarity using Adobe Illustrator. MSN was made using msa and seqinR in RStudio and edited for clarity using Adobe Illustrator.

To infer ancestry in the duck TRIM protein repertoire, we aligned all duck amino acid sequences and generated a ML tree. We color coded the tree based on the subfamily assigned by the C-terminal domain possessed by each TRIM protein or their orthologous TRIM protein in humans (**Figure 8A**). TRIM proteins segregate into two major clades, with one clade largely composed of the C-IV proteins. As previously mentioned, The C-IV subfamily is often associated with immune responses in mammals. TRIM8, 29, and 42 also cluster in this clade. TRIM8 and 29 belong to the C-V subfamily. The C-V subfamily has an unclassified C-terminal domain. TRIM42 is the sole member of the C-III subfamily and has a COS and FN3 C-terminal domain.

Within the second major clade, many of the subfamilies are represented, which supports the hypothesis that these subfamilies originated from a common ancestral TRIM gene. Typically, members of a subfamily group together in a clade. Subfamilies C-I and C-II appear to have descended from the same common ancestor, as they segregate into one large clade. Members of subfamily C-V do not share clades and are distant. This is perhaps not unexpected as the C-V subfamily is classified as RBCC domain containing TRIM proteins with unclassified C-terminal regions. The TRIM-like gene NHLRC1 and TRIM32 do not share a clade with the other C-VII subfamily members, suggesting they could have arisen through exon shuffling events independently from the other C-VII family members TRIM2, 3, and 71. We only included TRIM or TRIM-like genes that coded for full-length proteins in this analysis. TRIM211 had premature stop codons throughout the sequence and, as such, was excluded.

To assess the structural similarity of the TRIM proteins, we generated a minimum spanning network (MSN) (**Figure 8B**), which connects protein sequences (nodes) based on the distance between proteins without inferring ancestry and instead can infer shared function between these proteins (111). Most members of a subfamily cluster closely together, including members of the C-IV and C-VI subfamilies. Many C-V members are quite distant in the MSN, reflecting their disparate C-terminal domains and likely dissimilar functions. Most of the C-IV subfamily of TRIM proteins all cluster together in the center of the MSN. The MHC-linked C-IV TRIM mostly form the inner branches and cluster closely, apart from TRIM200, 207, and 208, which are quite far away from the other MHC-linked TRIM proteins on outer nodes. Proteins from the TRIM25 cluster group tightly together, expanding from the TRIM25 node, with the exception of TRIM14, which remains close but branches off of TRIM39 on a separate node. TRIM29, a C-V subfamily member, branches closely with TRIM25, 47, and 65, and RNF135. TRIM212, present in diapsids but appears missing in mammalian lineages, also clusters closely with the TRIM25 expansion. TRIM200 and TRIM213, both found in ducks but appear to be missing in chickens, share a distinct branch with TRIM203.

### 3.9 While most duck TRIM genes are ubiquitously expressed, some are tissue specific

To visualize the expression of TRIM genes in duck tissues, we mined the NCBI SRA database for RNA-seq reads from various tissues and aligned these reads to our 57 TRIM or TRIM-like sequences. We analyzed pairwise differences in TRIM differential expression (DE) between tissues using an MDS plot and most tissues clustered together (**Supplementary Figure S2**). Muscle and heart cluster very closely together when TRIM gene DE is compared between tissues. While all samples cluster together closely with like tissue samples, some tissues such as brain and testes tissues, for example, have more distinct clustering patterns. These results suggest that some TRIM genes are tissue specific, leading to distinct MDS plot clustering of tissues.

**Figure 9:**
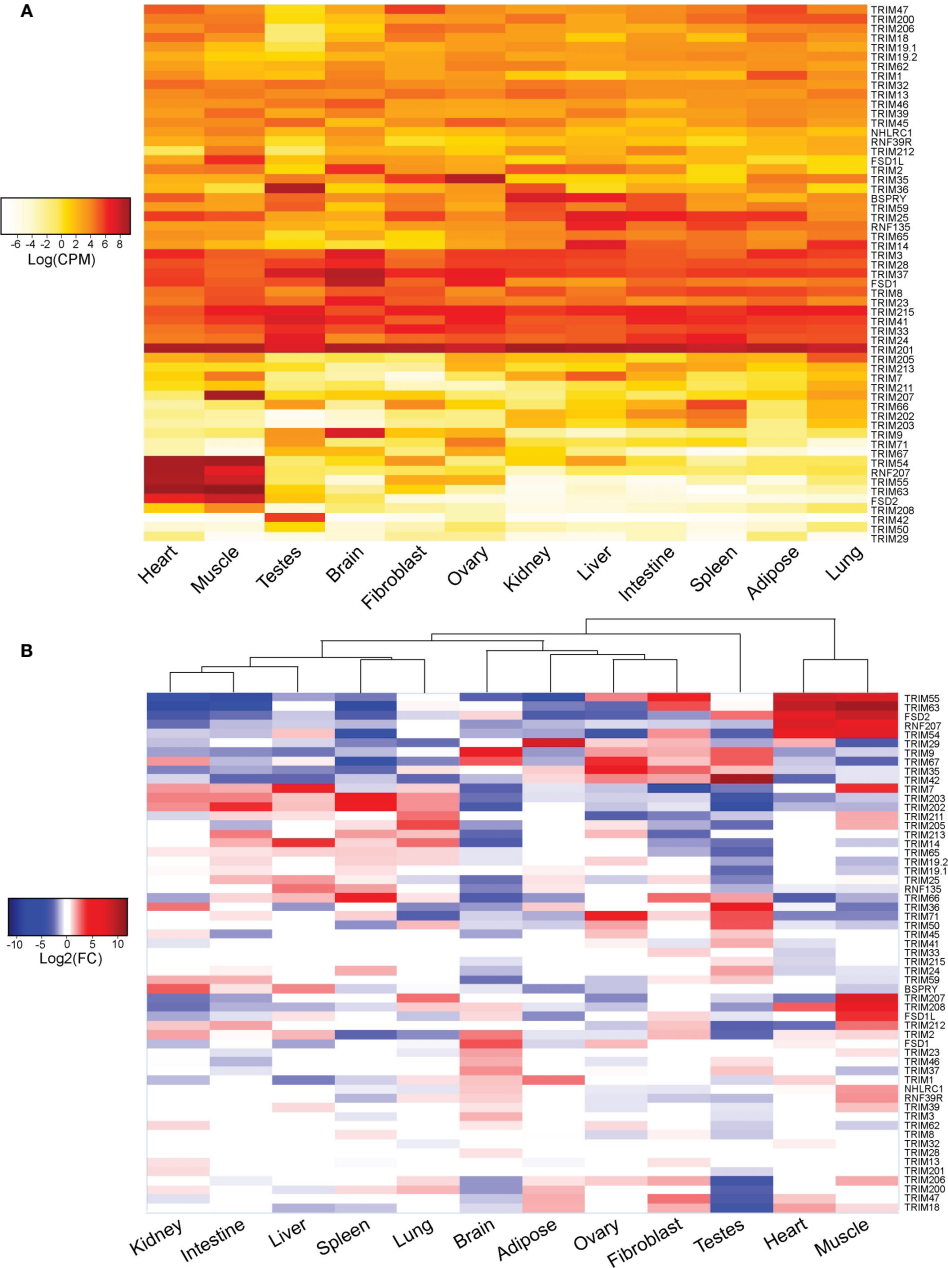
Many duck TRIM genes are ubiquitously expressed and abundant, while some demonstrate tissue-specific expression. The 57 duck TRIM or TRIM-like genes were used as a reference to align RNA-seq reads from the heart, muscle, testes, brain, fibroblast, ovary, kidney, liver, intestine, spleen, adipose, and lung tissues collected from domestic mallard (*Anas platyrhynchos*). Mapped TRIM gene reads were normalized to library size, and averages of log read counts per million (CPM) were visualized in a heatmap **(A)**. Reads mapped to each tissue were then compared to the average read count across all tissues to determine relative tissue expression **(B)**. All analyses were conducted by EdgeR in the Rstudio environment. Heatmaps were created using ggplot2 in Rstudio, and all heatmaps were edited in Adobe Illustrator for clarity.

To visualize abundance and relative expression of TRIM genes in each duck tissue, we generated heatmaps from the analysis of our normalized read counts (**Figure 9**). While many of the 57 duck TRIM genes are ubiquitously expressed in the tissues sampled, with varying levels of read counts per gene, there are some which are more specific (**Figure 9A**). TRIM54, 55, and 63, FSD2, and RNF207 have more reads mapped to muscle and heart than to other tissues. TRIM36 and TRIM42 were abundantly expressed in testis. TRIM9 appears to be highly expressed in brain tissues. TRIM201 and 41, two of the MHC-linked TRIM genes, had high read counts in all tissues sampled. In contrast, TRIM29 had very few averaged reads counted in any tissues sampled. Although TRIM211 encoded a sequence with a premature stop codon, it was still expressed at a low level in all tissues sampled, suggesting that the promoter is still active.

To determine the relative expression of TRIM genes sampled in each tissue, we compared the expression of TRIM genes from each tissue to the average expression in all samples. White or near zero log2 fold change (FC) values denote average expression of the gene in that tissue when compared to all other tissues, while negative (blue) or positive (red) log2(FC) values suggest lower or higher relative expression (respectively) when compared to all other tissues. The resulting heatmap visualizes the relative expression of TRIM genes in each tissue and demonstrates some of the more subtle differences in TRIM gene expression between the tissues (**Figure 9B**). Hierarchical clustering separates the expression pattern of TRIM genes into two major clades. The first major clade contains testis, then branches into two smaller subclades. The first subclade contains the immune tissues: kidney, intestine, liver, spleen, and lung. The MHC-linked TRIM genes TRIM202 and 203 have higher relative expression in these tissues than in any of the other tissues sampled. The second subclade contains brain and adipose tissues, fibroblasts, and ovaries. Muscle and heart form the second major clade, with the pattern of TRIM expression between these two tissues being very similar. Notably, TRIM7, 207, 208, and 212, and FSD1L have high relative expression in the muscle, and only average or low relative expression in the heart.

**Figure 10:**
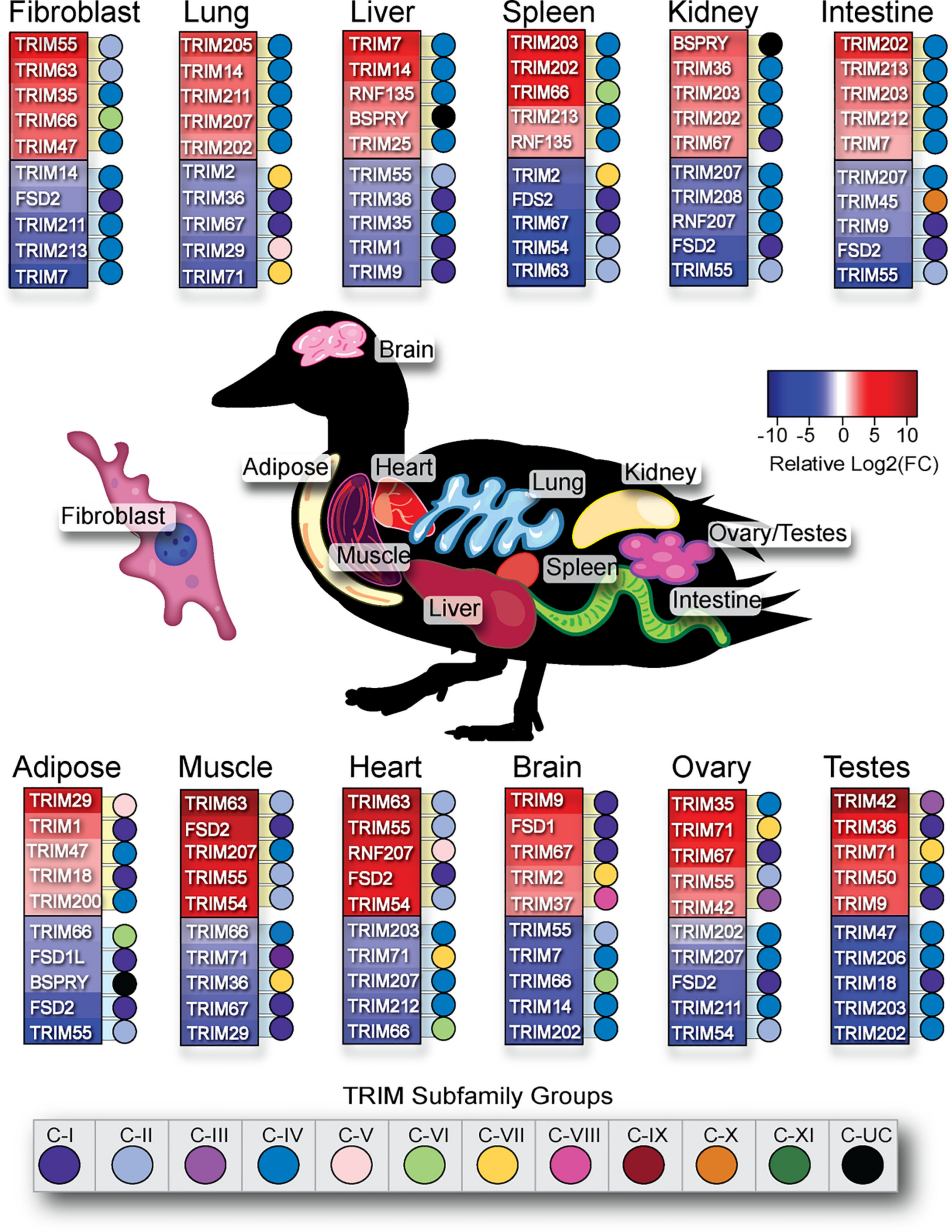
Immune relevant tissues have higher relative expression of C-IV TRIM family members. The relative expression of 57 duck TRIM or TRIM-like genes fibroblast, lung, liver, spleen, kidney, intestine, adipose, muscle, heart, brain, ovary, and testes were sorted by statistical significance (FDR<0.05) and then organized into top 5 highest or lowest relatively expressed TRIM genes per tissue. Dots next to TRIM gene are colour-coded according to TRIM subfamily designation.

We summarized the five highest and lowest statistically significant [false discovery rate (FDR) < 0.05] relatively expressed TRIM genes in each duck tissue analyzed (**Figure 10**). The C-IV subfamily of genes are often associated with inflammation and immune responses. Immune relevant tissues such as the lung, spleen, and intestine had a predominance of C-IV family members expressed at a higher level than in other tissues. Many MHC-linked genes also have higher relative expression in these immune relevant tissues. TRIM205, 207, and 202 have higher relative expression in lung. TRIM203 and 202 have higher relative expression in the spleen. TRIM202, 203, and TRIM7 have higher relative expression in the intestine. Immune privileged tissues such as brain and gonads, the C-IV subfamily genes, are among the least relatively expressed. Fibroblasts had low relative expression of many C-IV family members. TRIM35 and 47 are C-IV subfamily members with higher relative expression in duck fibroblasts, while TRIM55, 63, and 66 have higher relative expression in fibroblasts but are not C-IV subfamily members. TRIM29 was flagged as having higher relative expression in the adipose tissue; however, on closer inspection, two duck adipose samples had an over-representation of reads mapped to TRIM29. As the adipose tissues sampled clustered together when analyzed for TRIM gene expression (**Supplementary Figure S2**), it is likely that two of the birds sampled for this tissue were outliers.

## 4 Discussion

Here, we identify TRIM and TRIM-like genes in the duck by mining the SRA, gene, and protein NCBI databases and generating and interrogating a *de novo* transcriptome assembly. We found 57 duck TRIM genes, classified by their C-terminal domains, and compared them to mammalian and chicken TRIM repertoires. Most duck TRIM proteins are similar to their chicken orthologs. The duck TRIM sequences were aligned, and phylogenetic relationships between the TRIM proteins were inferred. Phylogenetic analyses show expansion of the C-terminal PRY-SPRY containing C-IV TRIM subfamily. Many duck MHC-linked C-IV TRIM genes lack mammalian orthologs, and this repertoire has expanded during diapsid evolution. The TRIM25 locus has also undergone rearrangement during vertebrate evolution, and our analyses suggest that this locus was adjacent to the MHC locus but separated onto different chromosomes in birds and eutherian mammals. Finally, we aligned RNA-seq reads from different duck tissues to the TRIM gene sequences to determine relative expression levels of these TRIMs in each tissue.

To our knowledge, only one study has previously classified the TRIM family genes in birds. Sardiello and colleagues listed 37 chicken TRIM genes during their analysis of the evolution of group I and group II TRIM genes in vertebrates (9). Other reports on avian TRIM genes characterized the expanded B30.2/PRY-SPRY TRIM genes in chickens (36, 94), turkeys (112), and ducks (37). Outside of birds, lineage-specific expansions of B30.2/PRY-SPRY TRIM genes have also been noted in fish (113, 114). Teleost fish had three separate expansions of B30.2/PRY-SPRY TRIM genes, with some specific to teleost fish as they are not found in amphibians, birds, or mammals. Zebrafish have 208 TRIM genes (*Danio rerio*), pufferfish have 67 (*Tetraodon nigroviridis*) (39), and grass carp (*Ctenopharyngodon idella*) have 42 TRIM genes (115). The differences in TRIM gene repertoire numbers in fish are partly due to the whole genome duplication events (116). Here, we document what appears to be a similar expansion of MHC-linked TRIM genes in reptiles, with many of these genes lacking direct orthologous counterparts in birds.

From our transcriptome interrogation, we found 57 TRIM genes in the duck. Of these TRIM genes, 54 could be found on their respective chromosomal locations in the NCBI Pekin duck genome (assembly ZJU1.0), and the location of FSD1 was inferred from synteny of surrounding genes in Ensembl rapid release genomes (GCA_017639305.1 and GCA_017639285.1). The duck TRIM gene repertoire spans 21 chromosomes, suggesting that many of these genes have evolved independently. Similar expansions can also be seen in human (9) and fish (39, 113, 115). As previously described, there is an expansion of PRY-SPRY containing TRIM genes within the MHC-B locus on chromosome 17 in the duck (37). The human MHC-linked TRIM genes are tightly linked and include TRIM10, 15, 26, 31, 39, and 40, and RNF39, while TRIM27 and 38 are telomeric to this region (35). In mice, TRIM27 and 38 are on chromosome 13 in the A3.1 region, while TRIM10, 15, 26, 31, 39, and 40, and RNF39 are located on chromosome 17 in the B1 region (105). In chickens, the MHC-B locus is located on chromosome 16 and contains many of the same TRIM genes found on chromosome 17 in ducks in syntenic organization, including TRIM 7, 201, 202, 203, 205, 207, 208, and 41 (36, 37, 94, 117). These genes were previously named to what was presumed to be orthologous human TRIM genes due to synteny and closest BLAST identity hits. Here, we updated the names of these genes to reflect that phylogenetic analysis suggests that they do not have clear orthologous genes in mammals. Previously, the chicken MHC-linked TRIM genes were named TRIM7.2, 7/7.1,39/39.2, X/27.2, BR/39.1, 27/27.1, 41, B30.2-1/BTN-1, and B30.2-2/BTN-2 (36, 94). Ruby and colleagues presumed that synteny of the TRIM genes in the MHC-B locus of chicken was conserved with human and used exon-based identity to infer ancestry, although their phylogenetic analysis suggested that TRIM27 and TRIM39 in chicken did not share ancestry with their presumed human orthologs. With the inclusion of TRIM genes from genomes of other vertebrates in the phylogenies, we show that many avian MHC-linked TRIM genes arose independently from the human MHC-linked TRIM genes. While mammalian MHC-linked TRIM genes appear to have arisen from a duplication event involving the common ancestral gene shared with TRIM7, avian MHC-linked TRIM genes appear to have arisen from an ancient duplication event involving the common ancestor shared with TRIM201. Both lizards and turtles have TRIM7, 41, and 206, while turtles, presumed to be a closer relation to birds than lizards (109), also have orthologs to duck TRIM201 and 202. While eutherian mammals do not share direct orthologs to duck TRIM genes in their MHC-locus, the Tasmanian devil has TRIM7 and 41 in their MHC locus, suggesting that TRIM7 and 41 are ancestral MHC-linked TRIM orthologs. TRIM206 appears missing in galliform lineages (37) but is present in ducks and many other birds. We document that TRIM204 appears to be missing in mallard duck and is present in chickens. This gene is annotated in tufted ducks and shares synteny with the chicken TRIM204 gene. Thus, it appears that the TRIM genes associated with MHC can change over time (117). Our results support a “birth and death” model of evolution of the MHC-linked TRIM genes (118), with lineage-specific duplication events increasing the TRIM diversity, and previously existing genes being removed by deletion, mutation, or inactivation (as in the case with TRIM204). We demonstrate that some MHC-linked genes have changed significantly between reptiles, birds, and mammals. It appears that TRIM7, 41, and RNF39 are ancestral to the MHC-linked TRIM genes in higher-order vertebrates.

Our phylogenetic trees show that ducks and chickens have direct orthologs for 53 TRIM genes, which segregate into distinct clades. Ducks have four proteins, which appear absent in chickens RNF135, TRIM200, 213, and 206. Chickens have TRIM204, 211, and 210, which are either incomplete or missing in ducks. TRIM211 orthologs are found in both birds and reptiles, while TRIM210 appears to be present only in birds. Duck RNF135, chicken TRIM211, and TRIM210 all fall within the clade containing TRIM14, 25, 47, and 65. No functions have yet been published for TRIM211 and TRIM210; however, due to their phylogenetic location, they may bind to helicase domains similar to the function of members of this clade in mammals (104). Some avian genes have been notoriously difficult to identify due to high GC content and lack of representation of these regions in genomic libraries (119). Indeed, this may be the reason that RNF39R is unannotated and unplaced in the chicken and the duck genomes. The newly identified RNF39R sequences have 72% and 74% GC content in the duck and chicken (respectively), making the contig harder to assemble and place in the genome.

Species-specific differences in TRIM repertoires are seen in other non-avian vertebrates, especially when comparing C-IV TRIM genes. There is a large expansion of C-IV TRIMs on chromosome 11 in primates (120), including TRIM5, 6, 22, and 34. TRIM5 and 22 restrict retroviral replication in primates (13, 14, 121, 122). Humans have a single copy of TRIM5, cattle have five co-orthologs of TRIM5, while TRIM5 is deleted in dogs (15). Primates have additional TRIM duplications on chromosome 11 close to the TRIM5/6/22/34 cluster, including TRIM49 and 64 (120). Bats host many types of viruses (123–125) and have duplications of TRIM5 and 22 (107, 126). There are other species-specific differences of C-IV members in bats, including duplications of TRIM25, 41, 60, and 75 (126). Duplication and deletion events of these genes may be a response to selective pressures from viruses. Large duplication events have occurred in fish C-IV TRIM proteins, and the number of these TRIM gene vary greatly, even within orders of fish. For example, *Perciformes* (perch-like fish) have from 107 to 672 C-IV TRIM genes in their genomes, depending on species (114). It is unknown if the fish C-IV TRIM expansions are duplicating from a similar common ancestor or expanding from different common ancestors in different types of fish. TRIM repertoires in vertebrates are likely rapidly evolving due to selective pressures from pathogens.

Our identification of two TRIM19 co-orthologs in ducks and chickens prompted us to search available avian genomes, which indicate two paralogs of TRIM19. In mammals TRIM19/PML is a key component of PML nuclear bodies (127). PML nuclear bodies regulate many important processes in mammals, such as the DNA damage response, apoptosis, and gene expression (128–130). PML is also involved in immune responses to viruses, by regulating signaling pathways during infection (131, 132), and are targeted by viruses to inhibit this signaling (133, 134). PML has not been annotated in any published fish (39, 113) or amphibian lineages (NCBI). The PML co-orthologs, however, appear in both birds and reptiles, suggesting this duplication happened in the common ancestor of diapsids. It is unknown if either of the avian TRIM19 paralogs form PML nuclear bodies or has antiviral activity.

Our phylogenetic tree of duck TRIM proteins demonstrated that most TRIM subfamilies form separate clades. The C-IV subfamily, the largest and most diverse of the subfamilies, has two major clades, the first containing the MHC-linked TRIM proteins. The avian-specific TRIM213 also clusters in the MHC-clade. This TRIM potentially translocated to or from the MHC region earlier in vertebrate evolution. TRIM39 and RNF39R are unplaced in the duck and chicken genomes, however, are located in the MHC-region in reptiles, mammals, and, in the case of TRIM39, also in the kākāpō. This suggests that RNF39 and TRIM39 may also be located in the MHC region of the duck and chicken. The MHC region has rapidly duplicated and expanded throughout vertebrate evolution, and many genes in this region are involved in adaptive or innate immune responses (135, 136). In chickens, the MHC region was dubbed as the “minimal essential MHC” due to it being much more compact and simpler than MHC regions found in mammals (137, 138), and our comparisons of this region between duck and chicken demonstrate that the chicken MHC region is more condensed than in ducks. Reptiles have large expansions of uncharacterized TRIM genes in the MHC region without any obvious orthologs in birds or mammals.

In humans, the MHC-linked TRIM genes attenuate innate immune signaling pathways (105), thus regulate responses to infection. As the avian MHC-linked TRIM genes are closely linked, they likely have co-evolved and may also be involved in innate immunity. Duck TRIM206 and TRIM205 both modulate signaling downstream of constitutively active RIG-I CARD domains when overexpressed in chicken cells, with TRIM206 increasing IFNβ promoter activity and TRIM205 decreasing it (37). It is not yet clear which components in the innate signaling pathway are targeted by these TRIM proteins. In birds, an ortholog of human TRIM41 was found in the MHC-B locus of chicken, with orthologs later found in the turkey (112) and duck (37) MHC-B locus. We also found TRIM41 in the MHC region of reptiles and marsupials. Human TRIM41 is not found in the MHC and is instead located on chromosome 5. TRIM41 in humans is known to restrict viral replication by selective targeting and ubiquitination of viral proteins (139, 140) and by augmenting antiviral signaling pathways (141). It is unknown if the avian ortholog of TRIM41 can restrict virus; however, TRIM41 is well conserved between birds, reptiles, and mammals.

A second group of duck TRIM genes within C-IV subfamily having PRY-SPRY domains includes the closely related TRIM25, 47, and 65, and RNF135. Our phylogenies support TRIM14, 25, 47, and 65, and RNF135 all originating from the same common ancestral TRIM gene in birds, reptiles, and mammals, which is consistent with what other groups have found when analyzing these TRIM proteins in mammals (104). Our analysis also suggests that TRIM29, a member of the C-V subfamily, belongs in this clade. In mammals, RNF135, TRIM14, 25, and 65 bind helicases involved in immune signaling using their PRY-SPRY domains, while the function of TRIM47 is unknown (104). Duck TRIM29 inhibits signaling pathways downstream of these helicases by catalyzing the addition of K29-linked ubiquitin to the signaling adaptor MAVS, resulting in a decrease in IFN signaling downstream of MAVS (50). RNF135 has previously been excluded from TRIM repertoires because it does not have the classical RBCC motif, as it is missing the B-box domain. Previous studies performed in our lab could not locate a RING domain in RNF135, and we had suggested that this protein would be largely inactive (110). However, the recent duck genome assembly and HMMER searches of the sequence obtained from our *de novo* transcriptome indicate that duck RNF135 does have a RING domain. The minimum spanning network places RNF135, TRIM25, 29, 47, and 65 on the same branch, while TRIM14 is branched separately, but still in proximity. While there is no documented function of TRIM47 as a modifier RLR pathways, the phylogenetic relationships and the placement in the MSN suggest that this protein may be involved in immune function in lower vertebrates. We demonstrated that the TRIM25 locus is adjacent to the MHC locus in reptiles and remains adjacent to the MHC locus in Tasmanian devils, thus likely was the ancestral organization. The TRIM25 locus is no longer adjacent to the MHC of birds or eutherian mammals because of genomic rearrangement during vertebrate evolution. Penguins and reptiles have TRIM16, which appears to be missing in many other birds, including chicken and duck. This suggests that the TRIM25 locus has undergone significant rearrangement throughout evolution. Many viruses target TRIM25 (142–145) and RNF135/RIPLET (45, 146) in order to evade antiviral responses in mammals. It is possible that the changes in this locus are due to selective pressures from pathogens targeting TRIM proteins.

Modifiers of the RIG-I signaling pathway appear missing in avian lineages. RNF135 was reported missing in chicken (110), Japanese quail (*Coturnix japonica*) (100), Procellariiformes (petrel), Sphenisciformes (penguin), and Falconiformes (falcon) genomes (101). RIG-I, the cytoplasmic detector of single-stranded RNA viruses, also appears missing in chickens (147), other Galliformes, petrels, penguins, and falcons (101). RIG-I is stabilized by ubiquitinylation by RNF135/RIPLET in mammals to increase type I interferon signaling during infection (104, 148–150). The loss of RNF135 corresponds with the loss of RIG-I in birds (101). Recently, Krchlíková and colleagues identified remnants of the RNF135 gene with partial exons with frameshifts in chickens. Pseudogenization of RNF135 has happened in many galliform birds, which have lost RIG-I, while the intact gene is present in two basal galliform birds that also have RIG-I. TRIM206 augments the RIG-I signaling pathway when cotransfected with constitutively active RIG-I in chicken cells; however, the mechanism is unknown (37). Kākāpō and barn swallow both also appear to be missing TRIM206; however, both species have RIG-I (NCBI). Penguins appear to be missing RNF135 but have TRIM206, and most species of penguin also appear to be missing RIG-I (101) with the exception of the Adelie penguin, which has RIG-I (NCBI). Falcons also appear to be missing RIG-I (101); however, we found an ortholog of TRIM206 in the Saker Falcon. While the loss of RIG-I and RNF135 appears to be linked in most bird species, the loss of TRIM206 and RIG-I does not seem to be correlated; however, a thorough search of avian genomes should be done to confirm this. It is important to note that while many genes appear missing in avian genomes, our data suggests that even with new long read genomic sequencing technology, some avian genes remain “hidden” in dark DNA (119) and can only be detected in transcriptome data. This should also be taken into consideration when analyzing avian genomic resources.

Many duck TRIM genes are ubiquitously expressed in all tissues sampled; however, some do show tissue-specific expression. Muscle and heart tissues in the duck had the most similar expression of TRIM genes of any of the tissues sampled. TRIM54, 55, and 63 are also known as muscle-specific ring finger (MURF) genes and are primarily expressed in muscle fibers in mammals (151, 152). Duck muscle and heart tissues highly express TRIM54, 55, and 63. TRIM9, 46, and 67 are all associated with neuronal development and brain tissues in humans (1, 152–154), and this pattern of higher relative expression in brain tissue is consistent with what we see in the duck. TRIM42 has the highest relative expression in the testis of the duck, which is consistent with human (152). We reported that duck TRIM71 has higher relative expression in both testis and ovary, compared to other tissues. In human, TRIM71 is highly expressed in testis but not ovary (152), suggesting that even with highly conserved TRIM genes such as TRIM71, tissue specificity can change during evolution.

Immune-relevant tissues had higher relative expression of the C-IV TRIM subfamily members. TRIM14, 202, 205, and 207 had the highest relative expression in lung tissues, while TRIM203, 202, and 213 had the highest relative expression in both the spleen and intestine. TRIM202, 203, and 213 are not yet functionally characterized, making them potential candidates for future immunological studies. TRIM genes which encode immune modulating proteins, had much less relative expression in immune privileged sites such as the brain and gonads. TRIM25, for example, which is known to help increase RIG-I signaling during viral infection in mammals (49) and ducks (46, 49), had much less relative expression in brain. TRIM206, known to increase IFN-β signaling when cotransfected with RIG-I in chicken cells (37), had lower relative expression in the testes. Immune privileged sites also had most C-IV subfamily TRIM genes expressed at a lower relative level than other tissues. It is possible that these transcripts have lower expression in immune-privileged sites to prevent inflammatory responses. Ducks have high relative expression of TRIM14 in both the lung and liver. TRIM14 has not been functionally characterized in ducks but is an antiviral protein in mammals, which can target multiple viruses for degradation such as hepatitis B virus (155) and influenza A virus (30). Ducks act as the natural host and reservoir to influenza A virus (40, 41, 156) and usually have reduced symptoms when infected with low pathogenic strains (40, 157). Highly pathogenic strains of IAV, however, replicate in the lungs of infected ducks and can cause disease (158–160). Young ducks are also susceptible to duck hepatitis virus (DHV), which replicates in the liver and results in liver damage and mortality (161–163). Potentially, the higher expression of duck TRIM14 in the lung and liver is a conserved protective mechanism that allows a quick response to viral infections from RNA viruses such as IAV and DHV.

Some TRIM genes are notably absent in birds, as they were not found in our genome searches or transcriptome. These include the cluster of C-IV TRIM genes located on chromosome 11 in humans, including TRIM6, 5, 22, and 34 (15). Many of these genes have direct antiviral activities, first noted in TRIM5alpha, shown to restrict HIV in non-human primates (23, 121). TRIM22 is known to restrict influenza virus in mammals (164). TRIM6 augments antiviral signaling pathways (165). These genes have undergone expansion and contraction in the mammalian genome, presumably in response to pathogen pressures (15). Our phylogenies suggest that the TRIM5/6/22/34 expansion in mammals happened within the expansion of other mammalian specific TRIM genes including TRIM21, 27, 38, 58, and 68. Birds also appear to be missing TRIM20/PYRIN, a C-V TRIM with a PYRIN domain in the N-terminus. TRIM20 has a proinflammatory role in mammals due it its interactions with the inflammasome component apoptosis-associated speck-like protein containing caspase recruitment domain (ASC) (166, 167). We found no TRIM20 ortholog in our duck transcriptome or in genomes of other birds. We also did not find an ASC protein in our transcriptome or on NCBI, suggesting that the “pyrin-inflammasome” may be absent in ducks.

More TRIM genes may be present in the duck but have greatly diverged and were not detected by our transcriptome interrogation. The incompleteness of the duck genome paired with an incomplete set of tissues to use for *de novo* assembly, leaves the possibility that we have missed some TRIM genes. Our study lacks tissues from the eye, stomach, pancreas, and bursa. If these tissues have tissue-specific expression of TRIM genes, we likely would not be able to find them in our transcriptome. TRIM genes are highly regulated during development, and screening embryonic tissues at various stages of development might help to classify TRIM genes predominantly expressed during development that would otherwise be rare. Additionally, recent duplications or highly similar genes cannot be resolved using *de novo* transcriptome assembly. If highly similar genes are not annotated in the available genomes, we would have missed them.

Throughout our analysis of TRIM genes, the issue of mis- or improper annotations arose. Many of the diapsid TRIM genes are assigned locus numbers and have computer-generated descriptions. Many gene names are used redundantly to describe multiple independent TRIM genes (i.e., TRIM39L, RFPL, etc.). We suggest that pipeline-assigned gene names should not be taken as proper annotations, especially in lower vertebrates, as these gene names are assigned on most similar human hit, which often is a human TRIM gene of low similarity. Naming of TRIM genes in non-human vertebrates has relied on BLAST, identity analysis, and presumed synteny to humans. Our results demonstrate that TRIM gene evolution is too complex to rely on these methods alone, especially when naming the C-IV TRIM subfamily members. To determine orthology, phylogenetic analysis should include characterized TRIM members from multiple taxa. We have implemented new rules in naming TRIM genes in non-human vertebrates and offer some suggestions when naming newly found TRIM genes in non-human species. We assigned diapsid TRIM genes without orthology to human TRIM genes as TRIM2##. The start at TRIM200 was to ensure there was no overlap in names between current TRIM annotations. TRIM genes with close paralogs should be given the same name, with numbers identifying them. For example, we named the TRIM19 paralogs TRIM19.1 and TRIM19.2. This will help inform the ancestry of these genes and preserve numbers for new genes. With the increasing number of non-model organism genomes available on public databases, it has become much easier to compare complex gene families between species.

We found 57 TRIM genes in the duck, with evidence that one of these genes is a TRIM-like pseudogene. We found key differences between the duck and chicken TRIM gene repertoires that highlight the complex and understudied mechanics of TRIM gene evolution. We show evidence that TRIM proteins in the C-IV family are rapidly changing in avian species, with important differences in the organization of MHC-linked genes and TRIM25 locus between species. Remarkably, these two regions are adjacent in a marsupial and reptiles, suggesting that they were linked in the ancestral vertebrate MHC region. This is the first major study in TRIM gene classification in birds, where species of birds were compared. As more complete genomes in other birds and vertebrates are sequenced and become available, we can better trace the expansions and deletions of TRIM genes in the vertebrate lineages.

## Data availability statement

The original contributions presented in the study are included in the article/**Supplementary Materials**. Further inquiries can be directed to the corresponding author.

## Author contributions

LC and KM conceived of the study. LC performed the computational analysis and originally drafted and edited the manuscript. RP completed the RBH analysis and provided scripts for analysis, provided feedback and editing. KM provided funding for the project, feedback on analyses and edited the manuscript. All authors contributed to the article and approved the submitted version.

## Supplementary material

Supplementary Figure 1 Flow chart documenting workflow used to generate *de novo* transcriptome and find *Anas platyrhynchos* TRIM genes.

Supplementary Figure 2 Multidimensional scaling plot (MDS) of duck TRIM and TRIM-like gene expression in tissues sampled. All RNA-seq libraries were accessed from the NCBI short sequence read archive (SRA), normalized by library size, compared using the TMM method in EdgeR in the RStudio environment and edited for clarity in Adobe Illustrator. Individual libraries were compared using dimensions 1, 2, 3 and 4. Individual samples were colour coded by tissue that library was sequenced from.

Supplementary Table 1 TRIM and TRIM-like genes present in the domestic mallard duck (*Anas platyrhynchos*).

Supplementary Table 2 TRIM and TRIM-like genes present in the chicken (*Gallus gallus*).

Supplementary File 1 Project numbers, tissue and data included from the NCBI SRA database.

Supplementary File 2 Accession numbers of C-IV TRIM proteins from representative mammals, reptiles and birds.

Supplementary File 3 Nucleotide sequences of duck TRIM or TRIM-like genes used in this study in fasta format.

Supplementary File 4 Amino acid sequences of duck TRIM or TRIM-like genes used in this study in fasta format.

Supplementary File 5 Nucleotide and amino acid sequences of chicken TRIM or TRIM-like genes used in this study in fasta format. Nucleotide sequences only for genes pulled from *de novo* chicken transcriptome.

Supplementary File 6 Raw read counts for all samples used after library normalization in EdgeR.

Supplementary File 7 Relative expression of TRIM or TRIM like genes in ducks.

Supplementary File 8 Maximum-likelihood tree of C-IV TRIM proteins from birds, reptiles and mammals, including MHC-linked TRIM genes from reptiles with no known orthologs in birds or mammals.
