# Supplementary figures and images for: Evolution and expression of the duck TRIM gene repertoire
Source: Front Immunol. 2023 Aug 9;14:1220081. doi: 10.3389/fimmu.2023.1220081 (PMC10445537; doi:10.3389/fimmu.2023.1220081)

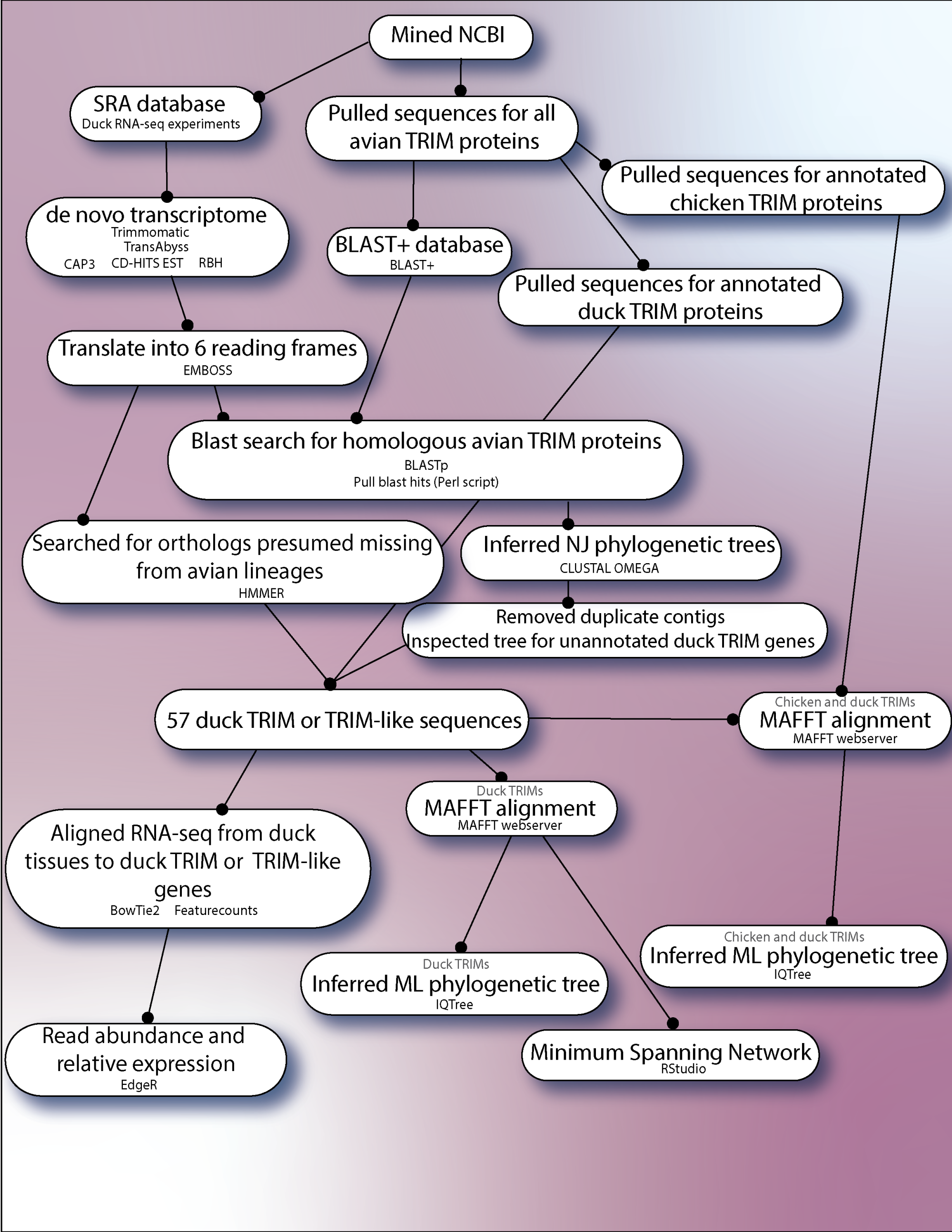

Supplement: Supplementary Figure 1 — Flow chart documenting workflow used to generate de novo transcriptome and find Anas platyrhynchos TRIM genes. [file Image_1.tif]

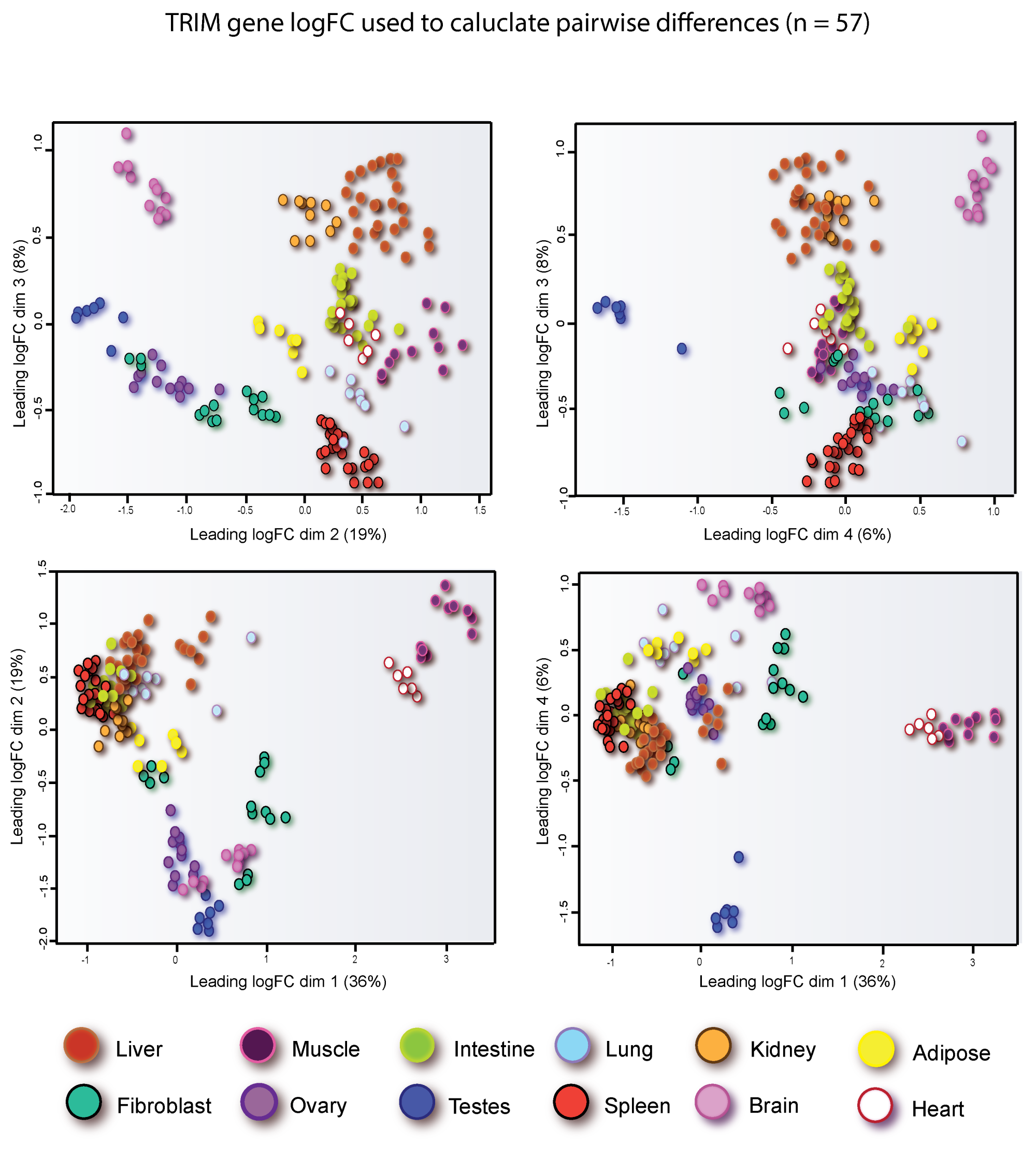

Supplement: Supplementary Figure 2 — Multidimensional scaling plot (MDS) of duck TRIM and TRIM-like gene expression in tissues sampled. All RNA-seq libraries were accessed from the NCBI short sequence read archive (SRA), normalized by library size, compared using the TMM method in EdgeR in the RStudio environment and edited for clarity in Adobe Illustrator. Individual libraries were compared using dimensions 1, 2, 3 and 4. Individual samples were colour coded by tissue that library was sequenced from. [file Image_2.tif]

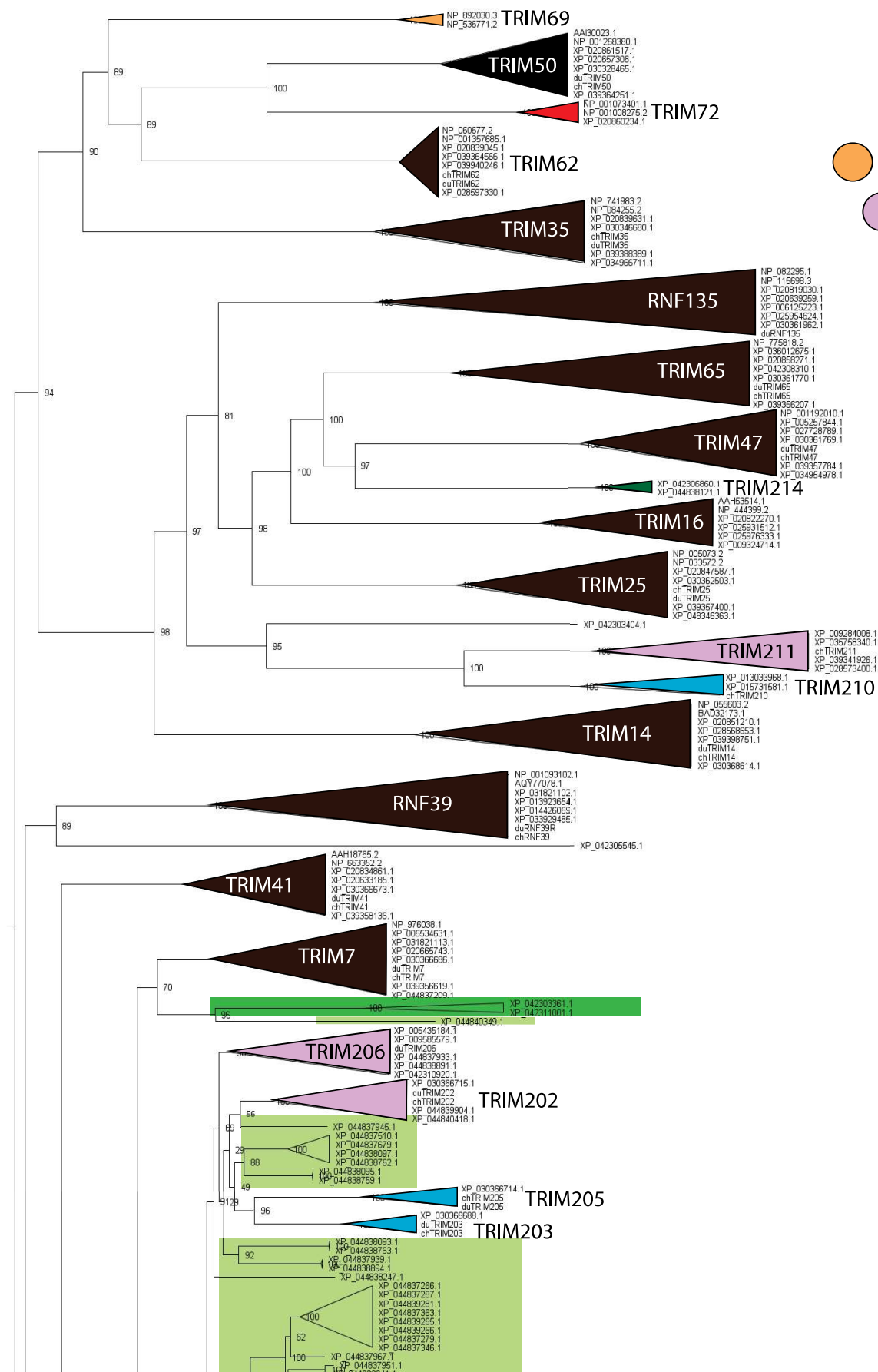

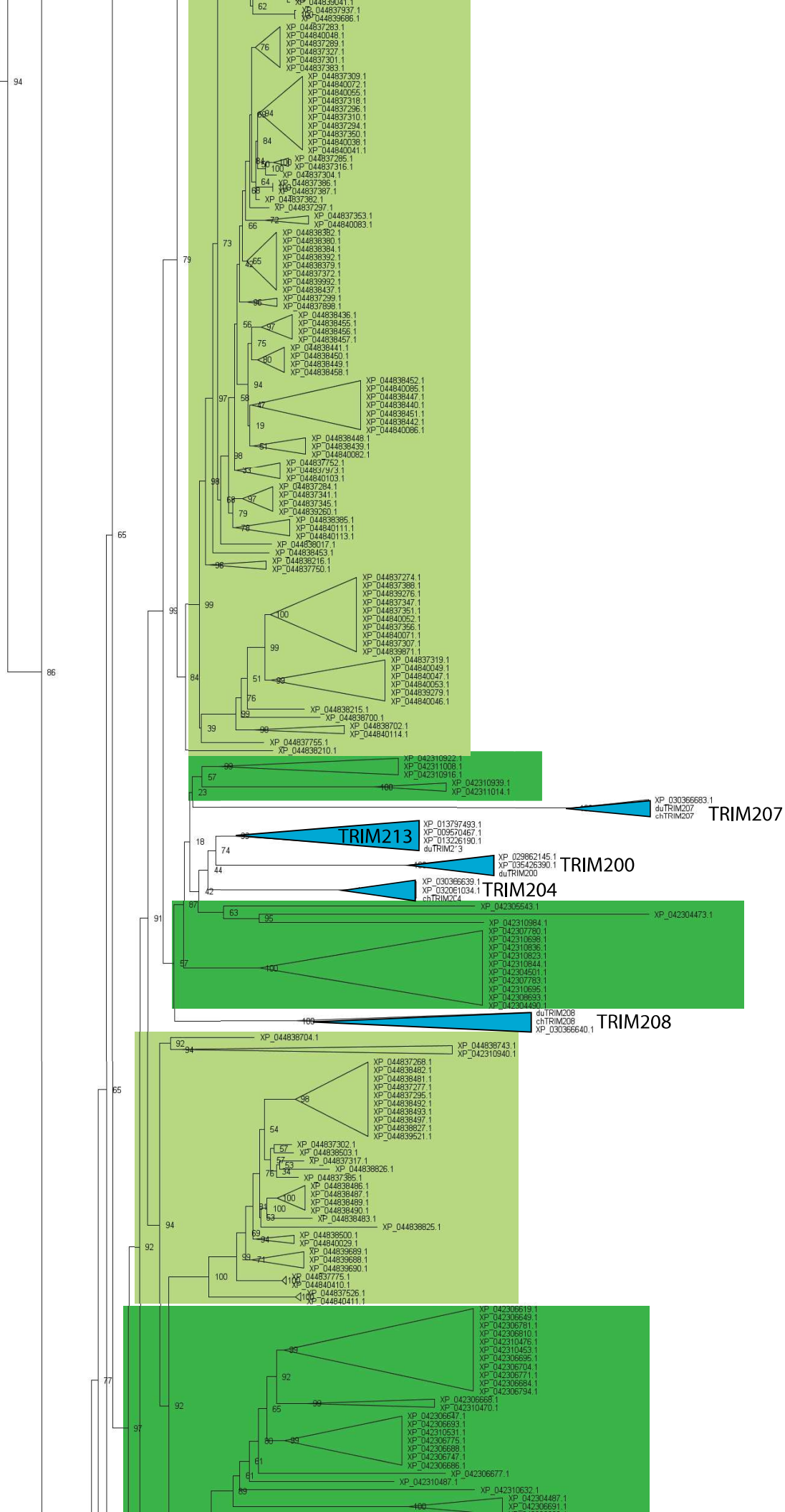

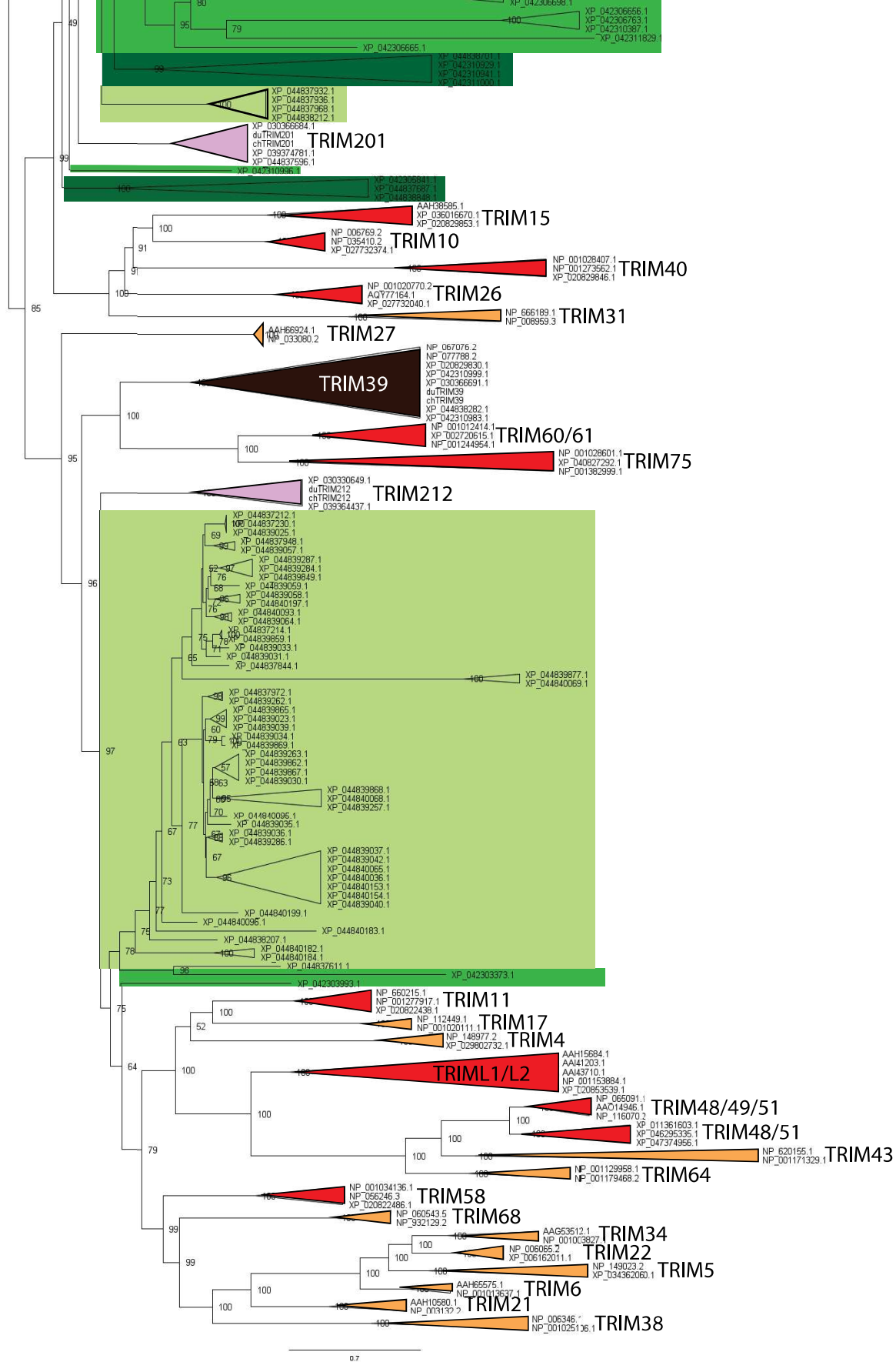

Supplement: Supplementary File 8 — Maximum-likelihood tree of C-IV TRIM proteins from birds, reptiles and mammals, including MHC-linked TRIM genes from reptiles with no known orthologs in birds or mammals. [file DataSheet_8.pdf]
