# Supplementary material for: Evolution and expression of the duck TRIM gene repertoire
Source: Front Immunol. 2023 Aug 9;14:1220081. doi: 10.3389/fimmu.2023.1220081 (PMC10445537; doi:10.3389/fimmu.2023.1220081)
Supplement: Supplementary File 3 — Nucleotide sequences of duck TRIM or TRIM-like genes used in this study in fasta format. [file DataSheet_3.docx]

>TRIM200

ATGGCCCAAGGAAACGCGGTGAAAAACCTCCGGGAGGAAGCCACCTGCGCTGTCTGCTTGGATTTTTTCCGCCGGCCCGTGATGCTCCTGCCTTGCGGCCACAACTTCTGCCGCTCCTGCGTCGCCCGCTGCGCCCAAAAAAGCCAAGACGGGGCCGGATCCTGCCCCCTGTGCCGCCTCCCGTTCCCCCCGGGAGGATTTTGCCCCAACCGGCAGTTGGCCAACGTGGTGGCCGCGGTGCGGGAGCTGGTGGAGGGCGAGGATTTGTGCCTTGGCGGAGACCTCGAGCGTCGCGTCCCCCCTGCCGAGCCCCTCGAGGATCGGGAACCCACCCCATCCTCCTCCAGACCCCAGGAGCACCTCGGACCCCCCCAAAATAAAAGGCAAGAGGACGAGCGAGCGGCCACGCTGCTGACACCCCCAGGGGGGGCCCAGCAGTCGCTGATGGCACGAGTGGAGGCGGAGAGGCAAAAGCTGCTGGCGGTGCTGGGGGGGCTGCGGGGGCTCGTGGGCCAGCAGGAATCGTGGCTACTGGCCCGCCTGGGCCACCTGCGGCGGGGGCTGGAGGAAGCCAAGGGCTGGCAAGGCGGTGGAAATCCATGGGACGCCGGAGTCGCCCCCAGCAGGAGCACGGAGCAGAGGCCGCGGCCGCTGGAGCTCGAAGCGGAGCTCGAGGACTTCTCCCAAAAAAACAACGCCCTGGAAGCGGTGGTGGAGAGGCTCAAAGATGTCCTGGCTTGCTCCCTGGAGGAAGACCTGGGGGGGTACCAGAAAGCGAGCGTCACCCTGGACCCGGCCACCGCTCACCCCCAAATCCTGGTGTCCCCGGACGGCCGCAGCGCCCGGCGGCGCAAAACCCCTCGGGACCCTCCTCCGGGGAGCTCGGAGCGTTTCGAGGCTCTTCGGTGCGTTTTGGGCCGCCAGGGTTTCGCCGCAGGACGGCACCGTTGGGCGGTGGAGGTGTCACCGGGACCCGACTGGGCTTTGGGGGTGGCTCGGGAATTCGTGCCCCGCAAGGGATGTTTCGGGTTGAGCCCCGCGCGGGGCGTTTGGGCTGTGGGGCAGTGGTTGGGGCAATTGAGGGCGCTCACCTGGCCAAGTCCCACCTGCCTGCACCTGGCCCGGGTGCCGAAACGCATCGAGGTGGCCCTGGATTACGGGGGAGGGAGGGTGGCTTTTCGGGACGCCGATAGCGAGGTGGAGATTTTCGCCTTTCCCCCCGCAGCCTTCGCCGGGGAGAGGCTGAGACCCCTGCTGTGGTTGGGAGAAGGGCCGGCCCTGCTCACTTTCTGCCCCTAA

>TRIM39

ATGTCTTCCTCCGACCCTCTGGAGACCCTCCGCGTGGAAGCCAGCTGCTCGGTGTGCCTGGGCTACCTTCGGGACCCCGTCCTCCTGGAGTGCGGCCACAATTTCTGCCGCTCCTGCATCACCCGCTGGTGGGCCGAGCTCTCCCGGGATTTCCCCTGCCCCGTTTGCCGAAAAACCTCGCGCCGCCGCGCCCTGCGCCCCAACCGCCAGCTGGCCAACATGGTCGAGGCCGCCCGGCAGCTCCGCGGCCCCAAAAGGAAAGCCAGCCCCGAAAACCCCGAAATTTGTTGTCAGATCCACGGGAGAGCCCTGGTGAGGTTTTGCCGCGAGGATCAAGCCCCCGCCTGCCGGCTCTGCGGGATTTCCAGCGCCCACCGCGCGCACCAAATGGTGCCCCTGGAGGAGGCGGCCCGGGAGTATAAGGAAAAGCTCCAAAATTTCCTCGAATCCCTGGAGCGCCGCCTCCAAGCCGTCTCCAGCTGCCGCGCGCGGGAGGAGAAAAAACCCAGCGAGCTCAAGAGGAAGGTGGCTTTGAGGCGGGAGAGGATCGCCCGCGAGTTCCAGGAGCTTCATCAGCTGCTGGAGGAAGAGGAGCGGCTGCTTTTGGGGCGGCTGGAGGAAGAGGAACGGGAAATCCTACAAAGGCTCCAGGCCAACCTGGAGCTCCTGGGGGAGCAGCGGAGGGCGCTGGGGGCCCTGGTGGCCGAGCTGGAGGAGAAATGTTTGCAGCCGGGGGCTGAGATGCTCAAGGACATCAAGGACACCCTGGCCAGGTGCGAGGCGGCGGCGGCGGGGGCCGAGCCCACCTCGGTGCCCAGCGAGCTGGAGAAAAATTTCGGCAGCTTCCCCCGGCAGTACTTCGCCCTCCGCAAGATCACCCGGCGCCTCCTCGGCGACGTCACCCTGGACCCCTCCACCGCCCACCCCAACCTCCTCCTCTCCCCGGACCTAAAAAGCGTCCGTTTCGCCCCAAACCCCCCCCGCCGCCTGCCCCACACCCCTCGCCGCTTCACCCTCTACCCCTGCGTTTTGGGGTCGAGGGGCTTCACTTCGGGACGCCACTACTGGGAAGTGGAGGTGGGCGACAAAACCCATTGGGCTTTAGGGGTTTGTAAGGATTCGGTGAACCGCCAGGGCGACCCCCCAAATTTCCCCGAAACGGGATTTTGGAGGGTGAGGCTTTGGAACGGGGAAAATTACGCCGCCACCACCCACCCCTTCACCCCGTTGAGGTTGCGGGTGAAACCTAAAAGGGTCGGGGTCTTCGTGGATTACGAGGAGGGGGAGGTGGCTTTCTACAACGTCACCGACCGCTCGCACATCTTCACCTTCAGCGGCACCTTCACCGAAAAAATTTGGCCCCTTTTCTATCCCGGCCTCAGGGCCGGCAAAAAAAACGCCGCCCCCCTCGTCATCCGCTCGCCGACCGATTGGGAATGA

>FSD1L

ATGGGCGAGCAGAGGGAGGATCTGCAAAGGATTGTCTCTACTCTAGCGAACAAAAAGGATGAAATTGTCAACTTTATTGACATACTGAAACAAACAATAAGAAACGTACAGGTAAACTCTTCTAATGTGTTCCGTGAGTTGGATAAAGAATTTGATGCTCTGCATTCAGTGCTGGATGAGATGAAGGAGAGTATGGCCAACACTATTCAGCAAGAAAGGACTGAAAAAATTCAAGCTCTGAATAATCAGCTTGGCCAGTGTAGCAGTGCACTTGAGAGTTCTGAAGAGCTGCTGGAATGTGCAACAACGATACTGAATATTAAGAACCCCACGGAATTCTCAAAGGCTGCCAGACAGATCAAAGATAGAATCACAGCGTGCCCAGCATTTCGCATCTGTCTGAAACCAACCATCAGTGATAATATGAGCCATTTGATGGTGGACTTCACACTGGAACGGCATGTACTCCAGGCTCTAAAGTTTTTGCCAGTCCCTAAAGTTCCAGAGATAAATACAGCAGCGTGTCTGGTTGCTGATAACTGCGTAACAGTAGCATGGAAAATGCCTGAAGAAGACAGCAGAGTTGATCATTTTGTTCTAGAGTATAAGAAAACCGACTATGATGGGCTTGCTCGAGTTAAGGATGAACAGCCTTGGGAAGTGATAGACTATGTGAAGGATTATGTATATACATTATCAGGTCTGAAATTTGATAAAAAATACATGACTGTTAGAGTACAAGCTTGCAACAAAGCTCTGGCTGGGGACTACTCAGATCCTGTGACTCTGGAAACCAAAGCATTTGTGTTTGGTTTGGACGATACTTCATCTCATCTGAACTTGAAAGTTGAAGAAAACTATGTTGAATGGGATCCTACTGGAGGCAAAGGTCAAGAAAAAGCAAAAGGGAAAGAAAACAAAGGCAGTGGCCAGTATCCTGTCCTGAAGAGCAATAAAAGAAGTGGTGCATCTCCTAAGAGGACTTCTGCCTCAAGGTCCGCAGCGAAGGGCTGCAGAGATCGTTTTACTGGTGAATCATACACAGTGTTAGGAGACACTGCTATTGAAAGTGGAGAACATTACTGGGAGGCAACAGCACAGAAGGACTGCAAAAGCTACAGTGTAGGAGTGACATACAGAAACCTGGGGAAATTTGATCAACTGGGAAAGACTAACTCAAGCTGGTGTATCCACATCAACAACTGGCTGCAAAGCACCCTTTCCGCAAAACATAATAACAAAAGCAAGACCCTAGACTTACCTATTACAGACAGAATAGGAGTATACTGCAACTTTGATGGAGGTCAGCTCATGTTTTATAATGCAAACTCAATGGAACTCCTGTATGCCTTCAAAACAAAGTTCACTCAACCGTTATTACCAGGTTTTATGGTTTGGTGCGGTGGGCTTTCCGTGTCTACCTGTTTGCAGGTGCCAAGTGTAGTGAAAGCATTCCAGAAAAGTGTAAATGGATTGAATTCTTCAACTTGCAGTCTATACAGTGTGCCAGTAATGCCTATTGAAAGTTCCCATTGCTGA

>FSD2

ATGTCAGCCAGATCTGGCAGGGTGAGACAGTATGAAAGTAGTGGCCAGCTGCCTGATCAGTCAGGTTCCCCACCGACTGCAGATTCTAGTGAGACTGAGGCTGAAGGCTTGATATTTTATCACATGGATCTTTACGGATCAAAGGAAAGGTTTGATATCTTTCCCGAGGAGCCATCCGGTCAAGCAGACAGATCTCGAGGGAATAGTAGAAAGGAACCTGCACTGAGCAGTGAAAAAATTCAGCACTCACAAGAAGCTGGATATGACTTGGAAAAGGAGGTTGCGGAGCTGGCCAAGATGTATGGACTTGATGAGGATAAAGAAAAAGAGCTTGAGCTTCTTGGAGGACATCCGCAGACGGTGGAGAGAAGATGGCCACCGGCTCACACACAAAAAGCAGGATCGCAAGGCTCTGTGTACAGTGCATCAAAAAGCAGCTCTCCAGTGAAAGATCAAAGTACAAAGCAACAGGGACTTCCTGACGAGGCTTCTCAAGATGAAGATCAGAGCAAAGCCAGAGCAGAGGATGACACTGAGAGCCACACGTGGTCCAGAGAGGGGCTTAGCAGCGGTGGCATGTCAGATGAACGGAGCAGCCAGGCAGTCAGTGAGGAGGAGGAAACAGCAGATGTTTTTTGCTCTACCTGCAAGATGCCAATCCGGGCCTTTGACAAACTGTTTGGTGAGCACAGGGACCATGAGGTGGCTCAGCTCTCCAATGCTGTGGAAAGCGAGAAGGAGGAGATTCATAAAAACATGTGCAAATTGGAAGAACAGATTGCTCAGATAGAAAACTTTGCCAGTCACTTGGAAGAGATCTTCATCACTGTAGAGGAGAATTTTGGGAGGCAGGAGCAGAACTTCGAGGTGCATTACAACGATGCAGTGCAAGTGCTTGCTCAGAAGTATGAAGAACAGTTGGAAGCTCTGGGGGAAGAGAAGAGGCAGAAGCTGGAAGCATTATATGGGCAGCTCGTCAGCTGTGGGGAACATCTTGACACCTGTAAGGAGCTGACAGACACTACCCAGGAGCTTTACCTGGAAAATGACAAAGCCAATTTTATGAAGGCAGCAGTAACCATGGTTGACAGACTGGAAGAATTCTTAAGGAAAGAAGTGGATTTAGAGCTTTCAACACTGCCAGACTTTGAAGAGCGCAAGATAGATTTCTCAGAAGTTGAACAACTAATGAACTCCATTAATACTATTCCAGCTCCTTGTGCCCCTGTGATCAATCCCCAGGCTCCCAATGCAGCGACTGGCACCTCACTGAGAGTTTGCTGGGGCCTCTTCTCAGATGACACTGTCGAGTGCTACCAGCTGTGCTACAAACCAGTGAGCAATGAGAGGCACGGTGACGAGCAAGCAGAGCATATGCTAAAAGTCAAAGAAACTTACTGCACCATTACTAATCTGTTGCCAAATACACAATACGAGTTTTGGGTCAGTGCTTTAAATGCTTCTGGCATCAGTCCACCAAGTGAAAGAGCTGTTTATGTAACAGCTCCTTCACCACCTATAATTAAGAGTAAAAAGATACGAAGCTGTGAAAATGCAGCACTGGTGTGCTGGGAATCTAGAGATATTAACCCTGTTGATTCCTACATGGTTGAATTGTCCAAGCTGACAGATGAAGAAAATGATGACACTATTACTGAATCTATTGTTGGGATTCCTAACTGTGAAGTCCTAATTCACCTCCAGCCAATACAAAGCTATCGCATCTGTGTTAGAGCCTTAAACCTGGGTGGTTCCAGTGAAAGCAGTGAGCCTGTCCTGATACACACCACAGGCACCTACTTCTGTCTTAATGAGGACACAGCCCATCCTTTATTGGCAATCCTGGATGATGGATTTACAATTGCCTGTGATGAACTGGAAAACCCGGAGTGCGATCTGCCTGTCTATGATAACAGCTTTACAAGGTGCATTGGGATCCTGGGCAGTCTGATCCCATTCCCAGGGAAGCATTACTGGGAGGTGGAAGCTGAGGAAGATACAGAGTACAGAATTGGTGTGGCTTTTGAAAACACTCCGAGGCATGGTTATCTGGGGGCAAACAACTCATCCTGGTGCATGAGGCACATCATCACACCATCAAGGCACAAATATGAATTCCTGCACAGCGGGATGACACCAGACATCAGAATTACCATTCCCCCCACAAGGATTGGCGTCCTGCTGGACTATGAGAACTACAGACTGTCATTTTTCAATGCTGATATTGCCCAGCACCTTTACACGTTCAATTCACACTTCCAGCATTACGTCCACCCCTGCTTTGCTTTGGAAACACCTGGTATCCTACAGATACGTACTGGCATTGCAGCACCCCCATGGACAGCCCTCCCATAA

>FSD1

ATGGGAGAGCAGGAGGCGCTGCGGAAGATCATCACCACGCTGGCCGTGAAGAATGAGGAGATCCAGAACTTCATCTACTCCCTCAAGCAGATGCTGCAGAACGTGGAGGACAACACGGCGCGGGTGCAGGAGGACCTGGAGGGCGAGTTCCAGTCCCTGTACGTGCTGCTGGACGAGCTGAAGGACGGCATGGTGACCAAAATCAAGCAGGAGCGCGCCAGCCGCACCTACGAGCTGCAGACGCAGCTGGCGGCTTGTGCCAAGGCGCTGGAGAGCTCGGAGGAGCTGCTGGAGACGGCCAACCAGACCCTGCAGACCGCCAACAACCACGACTTCATCGAGGCTGCCAAACAGATCAAGGATAGCGTGACCATGGCCCCCGCCTTCCGCCTCTCGCTCAAGGCCAAGGTGAGCGACAACATGAGCCATCTGATGGTGGATTTTGCCCAGGAGCGCCGCCTGCTCCAGGCCCTCGCCTTCCTGCCAGTGCCCAGCACCCCCGAAATCGACCTGGCCGAGTCGCTGGTGGCCGACAACTGCGTGACGCTGGCCTGGAGGATGCCCGACGAGGACAGCAAGATCGACCACTACGTGCTGGAGTACCGCAGGACCAACTTCGAGGGGCCGCCCCGCGCCAAGGAGGACCAGCCCTGGATGGTGGTGGAGGGCATCAAGGGCACCGAGTACACCCTGGCCGGGCTGAAGTTTGACATGAAGTACATGAATTTCCGTGTGCGGGCGTGCAACAAGGCCGTGGCGGGCGAGTTCTCGGAGCCGGTCACTTTGGAGACGAGAGCATTCACCTTCAAGCTGGACGCCAGCACGTGCCACCAGAACCTGCGGGTGGAGGAGCTCAGCGTGGAGTGGGACGCCACGGGTGGCAAGGTGCAGGACGTCAAGGCACGCGAGAAGGACGGCAAGGGCAGGACGGCCTCGCCCGCCAACTCTCCTGCCAGGGTGGTGCAGTCCCCCAAGAGGATGTCCTCAGGGCGTGGGGGCCGAGACCGCTTCACCGCCGAGTCCTACACGGTGCTGGGTGACACGCTGATCGACGGGGACGACCACTACTGGGAGGTGAAATACGACCGGGACAGCAAGGCTTTCGGCGTGGGGGTGGCGTATCGCAGCCTCGGCAAATTCGACCAGCTGGGCAAGACCTCGGCCTCGTGGTGCCTCCACCTCAACAACTGGCTGCAGGTCAGCTTCAGCGCCAAGCACGCCAACAAGGCCAAGGTGCTGGACGTGCCCGTGCCCGACTGCATCGGCGTCTACTGCAACTTCCATGAAGGGTTCCTGTCCTTCTACAACGCCAGGACCAAGCAGCTGCTGCACACCTTCAAGGCCAGGTTCACGCAGCCGGTGCTGCCCGCCTTCATGGTGTGGTGTGGCAGCTTCCACGTCACCGTGGGGCTGCAGGTGCCCAGCGCTGTGAAATGCCTCCAGAAACGCAACAGCACGGCCAGCAGCAGCTCCAGCCTGCCCTAA

>TRIM211

AAAGGCTGCAGGGAGTTTGCGAAGGAATTGGCTGGAGAATCTCTTTGATGAGATGTATAAGCAGCTGGGTAGCAGAAAAGGAGAGCTCCTCGAAACCCTCAGTCATGGTGAGGAGCAACAGCTTTCTCAAATTCAGGCACACATACAAACATACAAAGAGAGGAAGGATGTGACCAGCCATGATATACAGGGGATGAAGGCTCTGAGAGACCAGAAAGACCCACTACTTTTCACTGAGGTACCTAACAGCCACTCCCTTCCCAGTGATACTGCAGCTCTTGAATTGGCCTTTGCTTGCACTATACTAATTCTGCTTTCAAAATATCTTCTGCAGGGTTTGACAGAGATACAAACCAGGATAAGGAGGCCAGTTCCTGAAGAAGCTGCTGTAAAACTGCCAAAGCCACTTATTGTTTTAGATGAATCAACAAAAGAGGCTATTCTAACACTTTTCCAGCAATTCGTCTTGGTCATGAAGCTCTCACTGGAAGCATCACCTGTTCATAAACATCTGACTTTTGCACCATCTTGCATGGGTATATTCTACACAAGTGAATATGAACTCACTGCAAACCTCTAGATGCAGCAACAAATATCAATGTACAGAGTGAGGAGTCTCCTGCCTGTGCACAGCACCCAGAGCTTCTCGGAGGGCTGGCACTTCTGGGAGGCTGACATCAGCTGTGCAAGGCACTGGAAGCTGGGGGTCACTCATCAATGTTTTCATTGCTACCTGCAAATGTATGGCAATAATCTCAGTGTGTTCCTAGATAATACGAAGATCACAGAAAAGGCTACTACTACAGCTATCAGCACAGTCAGGGTAGAGCTGGACTGCAGAAGAAATGCAGTGTCATTTTATAACATCAAGACTAGAGTCCTTGCTAACAGACATCAGCTCATACAGACAGTAAACATCCCTGCGAACTATCCTGTCCATGCTGGCTTCAGCATCTCTAGCGGCTCCCTGAGACTCCAGTAGAGATGTGGGAGCAGGTCTGTCTTACCTTTTAAGCTTTGTTTCTCTATGTATCAGGGCTGCTGCTGTATGGGCTCTCCTACCTTGGCACAACAGAAACTGGGCTTTGGGCTGGGCCTGTGAGTGGCCAGCCTGTCCCCAAGTTATAATCAGGTTCTAGTCTTCAGCAGCAATGGTCTGAATCGAGAGAGAGAATGAGAAAATGAAACCCTACCATGTTTCTCCTCAGATTTTCAGCATGCTGTGGCTTGCTGCAGGAACTCCTATCTTACTTCTTACTTTTGATAATCATGATGAAAATATTGTGAAGTTGTACTTTAGTTCAGCTGTATAGATTGCTGTGGT

>RNF135

ATGGCTGTCCCAGCGGAGCTCGGTCGGCTGCTGGCAGACGTGGAGCTGAGCTGCTCCTGCTGCCTGCAGTACTTTACCGAGCCCGTGCGGCTGGCGAGCTGCAGCCACAGCTTCTGCCGGTCCTGCATCGACACCTACTGCAGGGGGAGGCGGCGCGCCCCCTGCCCGCTCTGCCGGGAGGACTTCGAGCCGAAGGACCTGCGGCCCAACCGGGAGCTGGCCGCCCTGGTCAGCCTGGTGCTGGGCGGGGGAAGGGGCGAGGGGCTGGGGGCGTGGGACCAGTCCACAGCCTCTGGAGATGGTGCCGGCGGTGGATGCAGCTCTGCGTGGCGGCGACCCGGGGAGAAGGAGGAACAGATCCGTGACATCTCTAAGCAACTGGAAATAACTAAAGAGACCATCAACGCCTTGAGGAAGGATCTCAGTAAAACAAAGGAATATACATCTCAGATCCTAAGCCAGATTACTGAAGACTTCTGTTGCATGAAGGAATACATTGAAAGACAAGAGGAAAACACACTGATGTTCATTGAACAGGAGCAAAGAGCAGCTCGACAGAAGATTGTGCAGACTATTCACCAGCTCTGTGTTGAAAAGTACAAACTCATCGACATCAAAGCCCAGATGGAGAAAGGATTAGAAAGTGATGAAATGGAGTGGCAGACTAGTAACTTACTTGAGAGAGGGGGAGGCTCACCTTCAACAATGCATAAATTTACAATTGATGAGAAGTTTAATGTTGTCAGAAGTGCTGTAGGAGATCTTAAGAGAAAGTTGGAAATTTTACTTTTGGAGGAATACCCTCAGCAGTTCCCACCAGCACAATCTCCAGACTTACACCAAGAGACAAGTGTCTGTTCATTATCTTCAGAGTCTGCAGCTAAAAGTCCAGAACCAAGCATTTCGAGCCAGTTTTCTCGGTGGGCAGATAATGTAACTTTTGATCTCACCACAGCATATGACCGCTTAGCAATCACAGCTCAGAACAGGAAAGTAATGGTGTCCAGCAACCCAACTTACTATGAACCATCACTCAAGAGATTCTGCATCAGCCAAGTGTTGTGTTCCCAGGGCTTCTCTACTGGCTGCCACTACTGGGAAGTAATTACCAAGGACAGTGATGGATGGGCTGTTGGAGTTGCTCGTGGAACGATTGGTAGAAGGGACAGATTAGGAAGAACAGAGAGTTCCTGGTGCGTAGAATGGGTAGGTCCCCAAAAGCAGTTGTCAGCATGGCACAGGAATCAAGAAACACTATTACGCAATGATAAACCATTGAAGGTTGGAGTTTTCCTGGAGCTACAGAAGACAGTGTCATTTTATGCCATCACTGACAGAGAAATGCTTTTGCATACATTTGAAATCAATAACTCAAATCCTCTTTATCCTGCTTTCTGGCTATACAGTCTAGATAAAAATGGATCTTTAACTATAAATCACATAAACAGGAAGTAA

>NHLRC1

ATGGCGGCGGCGGGGGAGGCGGGCGAGGCGGAGCTGGGCCTGCTGGAGTGCCGGGTGTGCTTCGAGCCCTACGGCCCCGGCGGGCAGCGGCGGCCCCGCAACCTGCCCTGCGGCCACGTCCTCTGCCGGGGCTGCCTGAGCGCCCTGGGCGGCCGGGGCCGGCTGGAGTGCCCGTTCTGCCGGCGGGTCTGCGGGCCCGCGGACACCAGCGACTGCCTGCCGCTGCTGCAGCTGCTCGAGGTGCTGGGCCCGGCTTGTGGCATCCTCCCGGCTGCCAGCAGCGGGGCCCCGGCGCTGCGGCTGGCCCTGGGCGGCTGGGGAGCTCTGGTCAACCCCACGGGGGTGGCGGCGTGCCCCAAGTCGGGGCGCTTGGCGGTGGCCCACGACGGCAAGAAGAGGATCCACGTCTATGGGCCCAGCGGGTCCTGCCTGCAGCGCTTCGGGGAGCGCGGCGAGGCGGGCTGCGACGTCAAGTACCCGCTGGACGTGGCCGTCACGGCCGACGGGCACGTGGTGGTCAGCGACGGCGGGGACCGGGCGGTGAAGGCTTTCGACTGGGAGGGACGTGGGGTGCTGGCGGTGCGAGAGGGCTTCTGCCTGCCCTGGGGCTTGGACGCCACCCCGGAGAGCGACGTGATCCTGGCGGATGCGGAGGCAGGGGTGCTGTACCGCCTGACGGCCGACTACGGGAAGGGCGAGCTGAAGAAGTGCCAGGTGCTGCGGGCCAAGTTAAGCAGCCCCAGAGCGGTGGCGGTCTGCCGGAGCTCGGGGGCGGTGGTGGTGGTGGAGCACCTGAAGGCCAGGGGGCCCAAGGGCAGCAGCACCCGGATAAAGATATTCAGCGCGGACATGGATCTGGTGGGACAGATGGATAGCTTTGGCCTCAACCTCTTCTTCCCCTCCAAAATATACACCACGGCCGTGGCCTTCGACAAAGAGGGCCGCATCGTAGTGACAGATGTCTGTAGCCAGGCCGTGATCTGCTTAGGGAAGCCTGGGGAGTTTCCCATCTTCAACCCTCTAATTAGCCACGGGCTTTCGTACCCCATAGGACTGACTTACACGGCAGACAATTCCCTTGTGGTGTTAGACAGTGGTGATCACACGGTAAAAATATATAGCTCCACCTGA

>RNF39R

ATGTCCCCCCAATGTCCCCCCCCCGTCCCTCAGCGCCCCCCCCACGTCCCCCCACCCCCCCCAATCCCCACCCCTCCCTCTCCCCTCCCTCTCCCTGCCCCTCCCCACCCCTTTAGGGCCCCACCCCAACCCCAAAACCCTCCGCCACCGCCGCCTCCTCGTCCCCAAACCCCCCAGGACCCCCCCCCAAAAACCCCAAAAATGTCCCCTTCGTCCCCTTTGCCCCCCCCCCAGCGGGGTCCTTTGGCCCTATTGGCTTCGGCCACCCGTTGTGGGGTTTGCGGGGGGGCTTTTCGGGACCCGGTGCTTTTGGGGTGCGAGCACGGCTGCTGTCGCCGCTGCCTCCCCCCCGGGGACCCCGTAAATTGTCCCCAATGTCACCGGGGGTGGCCCCGAAACCGAATTCGGACCCCGGTGGCTTTGGCCGTCGAGGTTCGCATCGCCCGACGCCTGGCGGGGGTCGCCGGGGGGGGCACGGGGGGGGGACAGGAGGAGGATGAAGAGGAGGAAGAGGAGGAGGAGAGGAGGAAGGGGATGAAGAGGAGGAGGAGGAAGGCGCAGAGGTGGAGCCTGGGGGCCCAGCTCCGCGCCGACACCGCCCCCCCCCGCCCCCCGCCGCCGCCCCACGAAGACCCCCGGGATGGGGGGGCCCCGGCAGCCGCCCCCCCGTCCCCCTCCCCAGGATGA

>RNF207

ATGGCCGGGGGCATCTTCTCCCCGCTGGGGAGCTGCTCGGAGCTGGAGAAGGGCGCCTGGCACCCGCTGGTGTGCCTGCTGTGCCACGAGCCCTTCCAGCACCCCTGCCTGCTCGACTGCTACCACAATTTCTGTGCCAGCTGCCTGCGCGGCCGCGCATCCGACGGCCGCCTGCGCTGCCCCCTCTGCGGGCACCCCTCGGTGGTGAGGGGGGGCACGGGGCTGCCCCCCGTGGACCGGCTCCTGCAGTTCCTCGTGGACAGCTCGGCCGACGGCGAGGAGGACGTGCAGTGCGCCAACTGCGACCGGCGCTGTGCCAAGGCGGATCTGGATGCCATGTGCTTCTGCAACACCTGCAGCCAGCCCCTCTGCGCCCCGTGCCGCGAGGAGACGCACCGCGCCAAGGTCTTCGCCCGCCACGAGATCGTTTCCCTCTCCAAGCGCACCAAGGACATCCACAAGAAGTGCCCACTGCACGAGGAGCCCTACATCATGTTCTCCACCGAGAAGAAGTCCATGCTCTGCATCAACTGCTTCAGGGACATGCAGGGGGAGAGCCGGGCGCACTGCATCGACATCGAGACGGCGTACGTGCAGGGCTGCGAGAAGCTGGACCAGGCGGTGCTGGCGGTGAAGGAGCTGCAGACGTCCACGCGCGAGGCCATCGTCCTCCTCAAGGCCATGATCGAGGAGGTGCGCAACAGTGCCAGCCAGGAGGAGACGGCCATCAACGCCCTCTTCAGCGGCATGCAGGAGCAGCTCTCCGAGAGAAAGAAGGCGCTCCTGAAAGCCGTGCAGAGCCAGCACGAGGAGAAGGAGAAGGCGTTCAGGGAGCAGCTCGCCCACCTCGCCTCCCTGCTGCCCACCCTGCAGGTCCACCTGGTGATCTGCTCGGCCTTCCTGAGCTCCGCCAACAAAGCCGAGTTCCTGGACCTGGGCTACCAACTGATGGAGAGGCTGCAGAGGATCGTGAAGCTGCCCCACCGCCTGCGGCCGGCACAGACCAGCAAGATCAACACCGAGTACCGGGCAGAGTTCGCCCGCTGCCTGGAGCCCCTGCTGATGCTCACCCCTCGCCGCTCCGTGGTGGGCAGCGCCGGCGGCATCGGGCCTGGCATCGCCGGCACCAACATGATCCCCGTCGGCCAGTCCTCCAAGACCCTGATGGTGCCCGGCTGCCCCCCCAGCGGCGATAAGATGTCCACGGGCTCCATGGTGCGGAAGCCGACGCTGCACCGCTACATCAGCACCAAGGTGCTGCTGGCCGAGGGGCGCGAGACCCCCTTCGCCGAGCACTGCCGCAACTACGAGAACACCTACCGGATGCTGCAGACGGAGATCCAGGGCCTGAAGGACCAGGTGCAGGAGCTGCACCGCGACCTGACCAAGCACCACTCGCTGATCCGCACGGAGATCATGAGCGAGATCCTGCAGAAGTCGCTGCAGATGGACGTGCAGATCGCGGCGCACTACTCCTCGGTGGAGATGATGCGCAGCGTCTTCGAGGAGGTCTGGGAGGAGACGTACCAGAGGGTGGCGAACGAGCAGGAGATCTACGAAGCCCAGCTCCACGACCTGCTGCAGCTGCGGCAGGAGAACAGCTGCCTGACCACCATCACCAAGCAGATCGCACCCTACGTCCGCTCCATCGCCAAGGTGAAGGAGCGGCTGGAGCCCAGGCTGCAGGAGCCCCGGGAGCCCAAGGATGAGCACACGCAGACGCTGCTCAGGATCGAGGACAGCAGCGAGGCAGCACAAAGGGACGGCTCGCCTGGCAGCAAGGAGAGCAGGGAGCGGGCCCTGGGGAGCCGAGGAGGCGGCCGCACGCCCACCCTCGCCCCCGAGGACCCGCTCCTGAAGAACGAGGAGCGCTGCCAAAGCAAGCAGAGAAGCGGGACCGAGGGCACCACTCGGGAAGACCCCACAGCTTCGAGTTGA

>BSPRY

ATGGCCCAGCGGGCCGGGGGGGCGCCGGCCGGGAGGGCGGCGGAGCTGCGGAACAAGCTCGTGGACCAGTGCGAGCGGCTGCAGCTGCAGAGCGCTGCCATCGCCAAGCACATGGCCGAGGTGCTGCCCGCCAAGAGCCAGAGCGTCCTGACTGCAGCCAATGGGGCACGAGAGTTGGTCATCCAGAGGCTGATGTTTGTAGGAAAGGTGTGTGAAAATGAAGAACAGCGGTTGCTGGAGAACGTGCACACAGAGGAGGAGCGGGTGCACCAGAGCATCCTGACTCAGCAGGAGCACTGGACTGAGGCTTTGCAGAAGCTGGATGCCCTCCGGACCTACCTGGTGGACATGATCACCAACAAGGATGACCAGGGCTTGGTGCGTGCAGAAAAAGAGATCTTCGAGAGGACAGAGGTGGCGGAGGGAATCTTGGAGCCGCAGGAGTCTGTGAAGTTAAACTTTAATCAGCAGTGTGTTCAGAGTCCACTGCTGCACCGGCTGTGGGCCTCTGCTGTTCTCTCCTGCATCACAGGCTCTCAAGAAATTCTCATTGACGAGAAAACTGTCAGCCCCCACCTGAGCCTGTCAGAAGACAAGAAAACCCTGACCTTCAGCCCCAAGAAAGCAAAGCTGGACTTGGACTGCCCCGACCGATTTGACCACTGGCCCAACGCTTTGGCTACTGCAGCTTTCCAGACAGGGCTCCACGCGTGGAAGATCAGTGTGGAGAAGAGCTGCGCCTACAAGCTGGGGGTTTGCTATGGCTCGCTGCCACGGAAGGGGTCTGGCAACGAGGTCCGCCTGGGCTTCAACGCTGCCTCCTGGGTCTTCTCGCGCTACGACAAGGAGTTCAGGTTCTTGCACGCAGGTCAGCCCCAGCCTGTGGAGCTGATCAAGTCCCCGGCCGAGATTGGCGTGCTGGTTGACTTTGCAGGAGGGGAGGTGCTCTTCTACGACCCTGACTCCTGTGCCATCCTCTTCTCCCACAGGGAGACCTTTGCGGCACCGCTCTATCCCGTCTTTGCAGTGGCACACCACAGCATCTCGCTCGTCCAGTGA

>TRIM1

ATGGAAACACTGGAGTCGGAACTGACCTGCCCTATCTGTCTGGAGCTGTTTGAAGACCCGCTGCTGCTGCCCTGCGCTCACAGCCTCTGCTTCAACTGCGCGCACCGCATCCTCGTCTCCCACTGCGCCACCAACGAGCCGGTGGAGTCTATCACCGCCTTCCAGTGCCCCACCTGCCGCTATGTCATCTCCCTCAACCACCGGGGCCTGGAGGGCCTCAAGAGGAATGTGACGCTGCAGAACATCATCGACCGCTTCCAGAAGGCGTCGCTGAGCGGCCCCAACTCCCCCAGCGAGAGCCGCCGCGAGAGGACGTACCGCAACAGCCCTACCATGTCCGTGGCCGGCGAGAGGATCGCCTGCCAGTTCTGCGAGCAGGACCCGCCGCGGGACGCCGTCAAGACGTGCATCACCTGCGAGGTGTCCTACTGCGACCGCTGCCTGCGGGCCACCCACCCCAACAAGAAGCCCTTCACCAGCCACCGCCTGGTGGAGCCCGTGCCCGACGCGCACTTCCGAGGACTCACCTGCCTGGAGCACGAGAACGAGAAGGTGAACATGTACTGTGTGGCTGACGACCAGCTCATCTGTGCCTTATGTAAACTGGTGGGCCGCCACCGGGACCACCAAGTGGCGTCCCTCAGCGATCGCTTTGAGAAGCTCAAGCAAACCCTGGAGACGAATCTCACCAACTTGGTGAAACGCAACAGCGAACTGGAAAACCAGATGGCCAAGCTGATACAGATCTGCCAGCAAGTGGAGGTGAACACGGCCATGCACGAGGCCAAGCTGATGGAGGAGTGCGACGAGCTGATGGAGATCATCCGCCAGCGCAAGCAGGTCATCGCCGTCAAGATCAAGGAGACGAAGGTGATGAAGCTGAGGAAGCTGGCCCAGCAGGTCGCCAACTGCCGGCAGTGCCTCGAGCGCTCGACGGTCCTCATCAACCAGGCGGAGCACATCCTGAAGGAGAACGACCACGCGCGGTTCCTGCAGACGGCCAGGAACGTGGCCGAGAGGGTCGCCATGGCAACTGCATCCTCTCAAGTTCTGATACCAGATATCAATTTTAATGACGCCTTTGAAAACTTCGCCTTGGATTTTTCCAGAGAGAAGAAGCTGTTGGAGGGGCTGGATTATCTCACCGCGCCCAACCCGCCGTCTGTCCGCGAGGAGCTCTGCACGGCCTCCCACGACACCATCACGGTGCACTGGATCTCGGAGGACGAGTTCAGCGTCAGCTCCTACGAGCTGCAGTACACCATCTTCACCGGCCAGGCCAACTTCATCAGTCTCTACAACTCCATGGACAGCTGGATGATCGTCCCCAACATCAAGCAGAACCACTACACCGTCCACGGGCTCCAGAGTGGCACCCGCTACATCTTCCTCGTCAAGGCCATCAACCAGGCAGGCAGCAGGAACAGCGAACCCGCCCGCCTCAAAACCAACAGTCAGCCTTTTAAGCTGGACCCCAAGATGGCTCACAAGAAGTTGAAGATCTCCAACGACGGGCTGCAGATGGAGAAGGACGAGAGCTCCTTGAAGAAGAGCCACACGCCCGAGAGGTTCAGCGGGACAGGGTGCTATGGCGCGGCAGGCAATGTCTTCATCGACAGCGGCTGCCACTACTGGGAGGTGGTGGTGGGATCCTCGACCTGGTACGCCATCGGCGTGGCTTACAAGTCAGCCCCCAAGAACGAGTGGATTGGGAAGAACTCCTCCTCCTGGGTCTTCTCCCGCTGCAACAACAACTTCGTGGTGAGGCACAACAACAAGGAGATGCTGGTGGAGGTCCACCCACAGATGAAGCGCCTCGGCGTCCTCCTGGATTACGACAACAACGCGCTCTCCTTCTACGACCCGGCCAACTCCCTCCACCTCCACACCTTCGAAGTCTCCTTCATCCTGCCCGTGTGCCCGACGTTCACCATCTGGAACAAATCCTTGATGATCCTCTCGGGTCTGCCCGCCCCGGACTTCATAGATTACCCAGAGCAGCAGGAGTGTAACTGCAGGCCCCAGGAGTCCCCGTACGTATCAGGGATGAAAGCCTGTCACTAG

>TRIM2

ATGGCCAGTGAAGGCTCTAACATCCCAAGTCCTGTGGTCCGTCAGATTGACAAGCAGTTTCTGATTTGCAGCATATGCCTGGACCGTTACAAGAACCCCAAAGTACTTCCCTGTCTGCACACTTTTTGTGAAAGGTGCTTACAGAATTACATTCCAGCCCATAGCTTAACCCTTTCTTGCCCCGTGTGCCGCCAGACCTCCATTCTGCCAGAGAAAGGGGTTTCTGCACTCCAGAACAACTTCTTTATTACCAACCTGATGGATGTCCTGCAACGAACGCCAGACAACAGCATTGAAGAGTCCTCCATCCTGGAGACTGTCACCGCTGTTGCTGCGGGCAAACCGCTCTCCTGCCCCAATCATGACGGAAATGTGATGGAATTTTACTGCCAGTCCTGCGAGACAGCCATGTGCCGGGAGTGTACAGAGGGAGAACATGCAGAACATCCCACAGTACCACTCAAGGATGTGGTGGAGCAACACAAGGCTTCCCTCCAAGTCCAGCTGGACGCTGTCAACAAACGGCTTCCAGAGATCGACTCAGCACTTCATTTTATATCTGAAATCATACACCAGTTAACCAACCAAAAGGCTAGCATTGTAGATGACATTCATTCCACTTTTGATGAACTCCAGAAGACTTTAAATGTGCGAAAGAGTGTGCTGCTTATGGAACTGGAAGTGAATTATGGCCTTAAACACAAAGTCCTCCAAACGCAGCTGGATACCTTACTGGAAGGACAAGAGAGCATTAAAAGCTGCAGCAACTTTACGGCCCAAGCCCTCAACCATGGCACTGAAACCGAAGTGCTGCTGGTGAAGAAGCAGATGAGCGACAAGCTGAATGAGCTGGCCGAGCGGGACTTCCCATTGCAGCCCCGTGAAAACGACCAGTTGGACTTCATAGTGGAAACAGAAGGCCTGAAGAAGTCAATTCACAATCTGGGTACCATTTTAACCACCAATGCTGTTGCCTCTGAAACAGTCGCCACAGGTGAGGGGTTGAGGCAGACTGTGATCGGACAGCCCATGTCTGTTACCATCACAACGAAGGACAAAGATGGGGAGCTCTGTAAATCTGGGAACGCTTACCTGACTGCTGAACTGAGCACGCCTGACGGGAGCGTAGCAGACGGGGAAATACTTGACAATAAAAACGGCACTTACGAATTTTTGTATACTGTTCAGAAAGAAGGGGACTTCACTTTGTCACTGAGACTTTATGATCAGCATATCAAGGGCAGCCCATTTAAACTTAAAGTGGTCAGGTCAGCGGATGTGTCCCCTACCACTGAAGGGGTTAAGAGGCGTGTTAAATCTCCTGGCAGTGGTCACGTGAAGCAGAAAGCTGTGAAAAGACCTGCGAGCATGTACAGCACTGGAAAAAGAAAAGAAAATCCCATTGAAGATGATTTGATCTTCAGAGTAGGCACCAAAGGAAGAAACAAAGGGGAATTTACAAATCTTCAAGGTGTAGCTGCATCTACAAATGGAAAAATATTAATAGCAGACAGTAACAACCAGTGTGTGCAGATATTTTCCAACGATGGCCAGTTCAAAAGTCGCTTTGGCATAAGAGGAAGGTCTCCTGGCCAGTTGCAACGGCCAACAGGAGTGGCTGTGCATCCCAGTGGTGACATCATTATTGCAGATTATGATAACAAATGGGTTAGCATATTCTCATCAGATGGAAAATTTAAGGCCAAAATTGGGTCTGGAAAGCTCATGGGCCCTAAAGGCGTTTCTGTGGATCGTAATGGACACATCATTGTGGTGGACAATAAAGCTTGCTGCGTCTTCATCTTCCAGCCAAATGGGAAAATAGTCACCCGGTTCGGTAGCCGAGGAAATGGTGACAAGCAGTTTGCAGGTACACTTGATGGTAATATGTAA

>TRIM3

ATGGCGAAGCGGGAGGCCAGCGCCAGCCCGGTGGTGCGGCAGATTGACAAACAGTTCCTGGTGTGCAGTATCTGCCTCGACCGCTACCGCAACCCCAAGGTGCTGCCCTGCCTGCACACCTTCTGCGAGAGGTGTCTCCAGAACTATATCCCGCCCCAGAGCCTGACCCTCTCCTGCCCCGTCTGCCGCCAGACCTCCATCCTGCCCGAGCGGGGCGTCGCGGCCCTGCAGAACAACTTCTTCATCACCAACCTGATGGAGGTGCTGCAGCGCGACCCTGACAGCCGTGGCCCCCACCCTGGCCAGGGGCTGGACCCCGTCAGCGCCGTCACCGGCCAGCCCCTCTCCTGCCCCAACCACGAGGGCAAGGTGATGGAGTTTTACTGCGAGTCCTGCGAGACAGCCATGTGCCACGAGTGCACGGAAGGGGAGCACCGGGAGCACGTCACGGTGCCGCTACGCGACGTGGTGGAGCAGCACAAGGCGGCCCTGCAGCAGCAGCTGGAGGCCATCAGGGGCAGGCTCCCGCAGCTGGCAGCCGCCATTGGGCTGGTGTCAGAAATAAGCCAGCAGCTGGTGGAGCGCAAGAACGAGGCCGTGTCCGAGATCGGCAGCACCTTCGAGGAGCTGGAGAAGGCTCTGCAGCAGCGTAAGGGCTTGCTGGTGCGGGACCTGGAGGCCATCTGCGGGGCCAAGCAGAAGGTGCTGCAGGCACAGCTGGACGCGCTGCGCCAGGGCCAGGAGAACATCCTCAGCAGCTGCGCCTTCACCGAGCAGGCGCTGCACCACGGCACGGCGCCCGAGGTGCTGCTGGTGAAGAAACAGATGAGCGAGCGGCTGAGCGAGCTGGCCAGCCAGGAGTTCCCGGAGCACCCGCACGAGAACGACCAGCTGGACTACGTGGTGGAGACCGACGGCGTCCGCAAGTCCATCCTCAACCTGGGCGTGCTCATCACCACCAGCGCCACGGCGCACAAGACGGTGGCCACCGGCGAGGGGCTGCGGCACGCGGTGGTGGGACAGCCCGCCTCGCTCAGCGTCACCACCAAGGACAAGGACGGGGAGCTGGTGCGCAGCGGCAGCGCCAGCCTGCGCTTCCAGGTGACGGGGCCGGACGGCAGCGTGGCTGAGAGCGAGGTGCTGGACAACAAGAACGGCACCTACGAGCTGCTGTACACACCGCGTGCCGAGGGCGACTTCATGCTCTCCATCCTGCTCTACGGGCAGCCCATCCGCGGCAGCCCCTTCCGCGTCCGCGCCGTCAAGGCCTGCGACGTGCCCCCCTCCCCGGACGACGTCAAGCGCCGCGTGAAGTCTCCCAGCAGCGGACACATCCGGCAGAAAGCCGTGCGGCGCCCCTCCAGCATGTACAGCAGCGGCAAGAAGAAGGAGAACCCCATCGAGGACGAGCTCATCTTCCGTGTCGGGAGCCGCGGCCGGGAGAAGGGCGAGTTCACCAACCTGCAGGGCATCTCCACCTCCAGTGCCGGCCGCATCGTGGTGGCCGACAGCAATAACCAGTGCGTGCAGGTGTTCTCCAACGAGGGGCAGTTCCGGCTGCGCTTCGGCGTGCGGGGCCGCTCCCCGGGCCAGCTGCAGCGCCCCACCGGCGTCACCGTGGACATGAACGGGGACATAATCATCGCCGACTACGACAACCGCTGGGTCAGCATCTTCTCCCCGGAGGGCAAGTTCAAGGTGAGGGGGGGGCCGGGGCGGGGGGCAGTGATTCGGGGCCCCCTCTGA

>TRIM7

ATGGGCTTGGGCTGGTTTCCTCCATCGCAGTGGCCAGCAGTGGTCCCACGCTGCCTCCCGCTGTGCCCCTGGGTGCTGGGACCCCTTGGGCTCCCTGCTAAGACCCTCCCGATGCAAAAGGCTGGGAGATTTGGGAGGACACCCCTTTCCTACACCTCTGCTGCGAGGAGAGAGGCCATGGCGGCGATGTTCCTGCCCGGCAATCTCCAGGACGAAGCCACTTGCTCTGTCTGCCTGGAGTTCTTCAAAGACCCCGTGTCCATCGAGTGCGGGCACAACTTCTGCCGGGCGTGCATCATCAAGAGCTGGAAGGACTTGGAGATGGACTTCCCCTGCCCGCAGTGCCGGGAGGTTTTCCAGCAGAAGAGCTTCAGGCCCAACCGGCAGCTGGCGAACATGGCTGAGATCATCAGCCAGTTCGCCCTCCGGGGGGCCAAGGGGGCCGAGGAGGATGGGCTGTGCGCGAAGCACAGGGAAGCCTTGAAGCTCTACTGCAAGGACGACCGCAGGACCATCTGCGTGGTGTGCGACCGGTCCCGGGAGCACCGGCCTCACGCCGTGGTGCCCGTCGACGAGGCGTCCGAGGAGTACAAGGAGAAAATCCAGGGCCGCTTGGATTTCCTGAAAAAGGAGAGGCAAGAGCTGCTGGAATTTAAAGTGAACGATGATAAGAAAACCCAGGAGCTTTTGAAAACAATAGAGAATGAGCGGCAGAAGCTGCTGGTGGAGTTCGAGGGGCTACGGCAGTTCCTGCACGACCAGGAGCACATCCTCCTGGGGCAGCTGGAGAAGATGGAGAAGAGCATCGCCAAGAGGCAGAACGAGAACATCTCCGACCTCTCCAAGGAGATCACGCTCCTCAACAAGCTCATCACGGAGCTGGAGGAGAAAATCCAGCAGCCCATGCTTGAGTTCCTCAAGGATGTAATGAGCATCATAAGCAGAAGTGACGATGTGAAGAGCCACAAGCCTGTACCCGTGTGCACGGATATGAAAATGCACGTCTGCAATTTCTCTCTCAAGACCGTCGTCCTTGAGAAAGTCGTAAAGAAATTCAAAGAAAACCTGCGGGATGAACTGGGAAGAGGTGAAAAAGAGGACCTGACCCTGGACCCCGAATCGGCGAACCACCTCCTCATCCTCTCCGCGGACCTCAAGAGCGTGAGGATGGGCTGTAGGAAGCAGGAGCTGCCCGACAACCCCAAGAGATTCGACACGAACTCGCGGGTCCTGGCCAGCACGGGCTTCAAGTCGGGAAGACATTACTGGGAGGTGGAGGTGGGGGCCTCAGACGGTTGGGCCTTCGGGGTGGCCAGGGAGTCCGTCAGGAGGAAAGGGCTGACGCAGTTTTCCCCCGAAGAAGGGATCTGGGCGGTGCAGCAAAATGGAGGCCGCTACTGGGCGGTCACCTCGCCCCAACGCACGCCGCTGTGCCTCAACCAGAAACTCAACAAGGTCAGGGTGTACCTGGATTACGAAGGGGAAGAAGTGTCCTTCTACAACGCCGACAACATGCAGCACATCTTCACCTTCAACGTCGCCTTCCAGGAGAAGGTGTTCCCCCTCTTTTCGGTTTGTTCCACGGTTACGTACATCAAACTGTGCCCTTGA

>TRIM201

ATGCGCACTCTCCCTCCCGCCCGCTCCCCCCGCGCACCGCGCATGCGCGGCCCCGCCCCCTCGCCGCACGCATGCGCGGCCCCGCCCCCTCCGGCCGCCAGTCGGGGCGGCGGCGCGGAGGGGTGCGGAGCTCGGGAGCGCTGCGCTGCTCGTGGCGGTGATTTGGGGGGGGGAAGGGGGAAAAAGGGGGAAGGTTCCGACATGGAGGCGCCGCCCGCTCCCCCCGCCGTGCCGCCGGCTCCCTGCAGCGCCGCCCGCAGCCTGCAGGACGAGCTGACGTGCCCGGTGTGCCTGGAGTACTTCACCGACCCGGTGCTGGTGGCCGAGTGCGGCCACAACTTCTGCCGGGCCTGCGTCACCCAGTGCTGGGAGGACTCGGCGAGGCGCCTCTGCTGCCCGCAGTGCCGGGAACCGGTGCCGCAGCGGCTTTTCCGCCCCAACCGCTCCCTCGGCAACATCGTCCACATCGTCCGCCAGCTGGGGCTGCCCCCCGGCCCCGCCGAGCCTCCCCCGGGGCCTCCGGCCCCATCTCCGCCTCTGCCCGCCGCCGCTCCGCCGGGTCCCCCGGGGCCTCCGCGGTGTCCCCGGCACGGGGAACCCTTGAGGCTTTTCTGCGTTCAGGACCGCCGGGCCGTCTGCGTGGTTTGCCACCTTTCCAGGGAGCACCGCACCCACACCGTGCTGCCCGCCGAGGAGGCGGCACACGCCGCCGAGATCTCTTCCTCCTTCCAGGAGGTGCCGCAGGAACATCTGAGCTCCCTGCGGAAAGGACGTGAAGAAGCCAAGGCCGAGAGGGAGAGGCAGAGCGAAGATTTGCTGAAGCAGACAGAAGTGGAGAGGCAGAAGATCGTGGCCGAGTGCAAGGAGCTGCGAGGTTTCCTGGAGGAGAAGGAGCAGCTCCTGCTGTCCCGCCTGGAGGAATTAGACAGAGACATCGTCAAGAGGAGAGATGAGAGCGTCTCCAGGCTCTCCGAGGAGATCGCCCAGCTCGATAAGTTGTTGGGGGAGCAGGGAGGAGAAAACGGCCCCGGGAACCAGTCCGGGCAGGTTGTCACCACGGCTGGAAGCAGCTTTGAGAGCTGGATGTTTTGCAAGCCGGAGGCCGGCTTTGCCGAGCTGGAGAAGAAACTGAAGAGCTTCTCCCAGAAGAGCGCGGTGCTGAAAGAGGTCCTGCTGGAGTTCAAGGAAAACCTTCGGTTTGAGTTGGAGAACGATACAGGCGATCTCTCTCTGGATCCGGACACGGCCAACCCCTACCTGGTGCTGTCGGAGGACAAGAGGAGTGTGAGGCTCCGGAGTGCCCCCCAGGAGCTGCCCGCCAACCCCAAAAGATTCGATTATTCCTTCTGCGTGCTGGCGGCCGAGGGTTTCGTGGCTGGGAGGCATTACTGGGAGGTGGAGGTGGGCGACGGGGAGAGCTGGGTGCTGGGGGCTGCCCGCGAGTCGGTGCGGAGGAAGGAAAAAATCGACTTCGCGCCCGAGGAAGGGATTTGGGCGGTGGGGTTGAACTGGAAGGGGAAAAATTGGGATCAGTACCAGGCTTTCACCTCCCCGGAGACGCCCCTGTCCCTGTGCGAGAGGCCCAGGAAGATCGGGGTGTACCTGGACTACGAAGGCGGCTGGGTGGCGTTTTACAACGCCGATAACATGGCTCCCATCTTCACCTTCACCGCCGCTTTCACCGAAAAGATTTTCCCTTTCTTCTGGCTCTTCTATGTGGGCTCCTCGCTCTCCCTTTGCAATTGA

>TRIM8

ATGGCCGAGAACTGGAAGAACTGTTTCGAAGAGGAGCTCATCTGCCCCATCTGCCTGCACGTCTTCGTGGAGCCCGTCCAGCTGCCCTGCAAGCACAACTTCTGCCGGGGCTGCATCGGGGAGGCGTGGGCCAAGGAGTCGGGCTTGGTGCGGTGTCCGGAGTGCAACCAGGCGTACAACCAGAAACCCAACCTGGAGAAGAACCTGAAGCTCACCAACATCGTGGAGAAGTTCAACTCCCTCAACCTGGAGAAGCCCCCCTCCGTCTTGCACTGCGTCTTCTGCCGCCGGGGCCCTCCGCTGCCCGCCCAGAAGATCTGCTTGAGGTGCGAGGCTCCGTGCTGCCAGTCCCACGTCCAAACCCACCTGCAGCAGCCCTCCACGGCCCGTGGGCACCTCCTGGTGGAGGCGGACGACGTTCGGGCCTGGAGCTGCCCCCAGCACAACGCCTACCGCCTGTACCACTGCGAAGCCGAGCAGGTGGCCGTCTGCCAGTTCTGCTGCTACTACAGCGGGGCCCACCAGGGACACTCGGTCTGCGACGTGGAGATCCGCCGCAATGAGATCAGGAAGATGCTGATGAAGCAGCAGGACCGCCTGGAGGAGCGGGAGCAGGACATCGAGGAGCAGCTCTACAAGCTGGAGTCGGACAAGCGGCTGGTGGAGGAGAAGGTGAGCCAGCTGAAGGATGAGGTGCGCCTGCAGTACGAGAAGATGCACCAGATCTTGGACGAGGACTTGCGGAAGACCATGGAGATCCTGGACAAGGCCCAGGCCAAGTTCTGCAACGAGAACGCCGCCCAGGTCCTGCACCTCAACGAGCGCATGCAGGAGGCCAAGAAGCTCCTCAGCTCCGTCCAGGTCATGTTCGACAAAACCGAGGACATCAACTTCATGAAGAACACCAAGTCTGTGAAAATCTTAATGGACAGGACCCAGAACTGTACAGGTGGCAGCCTGCCTCCACCCAAAATCGGGCACCTCAACTCCAAGCTCTTCCTGAACGAGATCGCCAAGAAGGAGAAGCAGCTCAGGAAGCTGCTGGAAGGTCCCCTGAGCACCCCCGTCCCCTTCCTGCAGAGCATCCCCATGTACCCCTGCACCGTCAGCAACTCCGGCGCGGAGAAGCGCAAGCACTCCACCGCCTTCCCCGAGGGCAGCTTCCTCGAGCCATCGTCCGGGACTGTGGCGAGCCAGTACATGAGCCAGGGCGCTTCGGCCGGCGAGGGGCAGTCCGCACAAGCCATGGTGCCTTGTAGCTCCACGCAGCACATTGTTGGACTTCCCAGCGGCCCGCAGCCGGTGCACTCGGGCTCCGTCTTCAACCCATCTCACTACCCCAACACCACCTCATCTCAGCAGTCCGTGCTCTCCCAGTACGGCGGGCGCAAAATCCTCGTCTGCTCGGTGGACAACTGTTACTGTTCCTCGGTGTCCAACCACGGCGGGCACCAGCCCTACCCGCGCTCGGGGCACTTCCCCTGGACGGTCTCCTCGCAGGAGTACTCGCACCCGCTGCCGCCCGCGCCCGCCGTGCCCCAGTCGCTGCCGGGACTTGCGGTGCGGGAATGGATTGACGCGTCCCAGCAGCACGGGCGGCAGGATTTCTATCGGGTCTACGGCCAGCCGTCGGCCAAACACTACGTCACCAGTTAA

>TRIM9

ATGGAAGAGATGGAAGAGGAGCTGAAATGCCCGGTGTGCGGCTCCTTCTACCGCGAGCCCATCATCCTGCCCTGCTCGCACAACCTGTGCCAGGCGTGCGCCCGCAACATCCTGGTGCAGACGCCGGACTCGGAATCCCCGCAGAGCCGCCGCGCGTCGGGCTCGGCCGTCTCCGACTACGACTACCTGGACCTGGATAAGATGAGCCTGTACAGCGAGGCGGACAGCGGCTACGGCTCCTACGGCGGCTTCGCCAGCGCTCCCACCACCCCGTGCCAGAAGTCCCCCAACGGCGTGCGGGTTTTCCCTCCCGCCGCCCCGCCGCCCCCCGCCGCGTTGGCCCCTCCGCCTCCGCCGCCCCGCAACGCGTGCCTGACGTGCCCGCAGTGCCACCGCAGCCTGGTGCTGGACGAGCGCGGGCTGCGCGGCTTCCCCCGCAACCGGCTGCTGGAGGGCGTCATCGACCGCTACCAGCAGGGCCGGGCGGCGGCGCTGCGCTGCCAGCTGTGCGAGAAGGCGCCCAAAGAGGCGGCCGTCATGTGCGAGCAGTGCGACGTCTTCTACTGCGAGCCGTGCCGCCTGCGCTGCCATCCGCCCCGCGGCCCCCTGGCCAAACACCGCCTGGTGCCGCCCGCGCAGGGCCGCGTCAGCCGCCGCCTGAGCCCCCGCAAGATCTCCACGTGCACGGACCACGAGCTGGAGAACCACAGCATGTACTGCGTGCAGTGCAAGAGCCCCGTGTGCTACCAGTGCCTGGAGGAGGGCAAGCACTCGAGCCACGAGGTGAAGGCGCTGGGGGCCATGTGGAAGCTGCACAAGAGCCAGCTGTCCCAGGCCTTGAACGGGTTGTCAGACAGAGCCAAGGAGGCGAAAGAGTTCCTGGTGCAGCTCCGCAACATGGTGCAGCAGATCCAGGAGAACAGCGTGGAGTTCGAGGCCTGCCTGGTGGCCCAATGTGACGCCCTCATCGACGCCCTCAACAGAAGGAAAGCCCAGCTGCTGTCCCGCGTCAACAAGGAGCACGAGCATAAGCTGAAGGTGGTGAGAGACCAGATTTCCCACTGCACGGTGAAGCTGCGGCAGACCACGGGGCTGATGGAGTACTGCCTGGAGGTCATCAAGGAGAACGACCCCAGCGGCTTCCTGCAGATCTCCGACGCTCTCATCAGGAGGGTGCACTTAACCGAGGACCAGTGGGGAAAAGGGACGCTCACACCCCGCATGACCACGGACTTTGACCTGAACCTTGACAACGGCCCCCTGCTGCAGTCCATCCATCAGCTCGACTTCGTGCAGATGAAAGTTTCCTCCCCAGTGCCGGCCCCCCCAATCCTGCAGCTGGAGGAGTGCTGCACCCACAACAACAGTGCCACGCTGTCCTGGAAGCAGCCCCCCTTGTCGACGGTGCAGGTGGAAGGCTACATCCTGGAACTCGATGATGGCAACGGAGGGCAGTTCAGGGAGGTGTACGTGGGCAAGGAAACCATGTGCACGGTGGACGGGCTTCACTTCAACAGCACTTACAGCGCCCGCGTCAAAGCCTTCAACAAAACAGGAGTCAGCCCCTACAGCAAGACCCTGGTGCTGCAGACATCCGAGGTAGCTTGGTTTTCTTTCGATCCTGCCTCTGCACACGCTGACATCATCTTTTCTAACGACAACCTGACTGTCACCTGCAACAGCTACGATGACAGGGTTGTGCTGGGGAAAACGGGCTTTTCCAAAGGGCTGCACTATTGGGAGCTGTCCATCGATCGCTACGATAACCACCCGGACCCAGCCTTCGGCGTGGCGCGCATCGACGTCCTGAAGGATGCCATGCTGGGCAAGGATGACAAAGCTTGGGCCATGTACGTGGACAACAACCGCAGCTGGTTCATGCACAACAACTCTCACACCAACAGGACTGAGGGTGGGATAACGAAAGGTGCCACGGTGGGAGTGCTGCTGGATTTGACCAGGAGGACCTTGACGTTCTCCATCAATGAGGACCAGCAGGGCCCTGTCGCCTTTGAGAACCTGGAGGGCCTGTTCTTCCCAGCAGTCAGCCTCAACAGGAACGTGCAGGTGACGCTGCACACGGGGCTGCCGGTCCCTGAGTTCTACGCCTCGCGCTCGGCCATGCAGTGAG

>TRIM207

ATGATGGAGTCAGGGAAGAGGAATGCAGATCTGGAAGAATGGGATGCAAAAATCAGGAAGTTAACAGAAGAATTTGAAGAAAGTGACACAGAACTCGAGGAATGTGACACAGAAAATGAAATAGGGGAACTCACAGCTGAGATAGACAATCTGACTGCGGAGCTTGAGGAACGTGATAGAAAAATTAGAAAACTTACTTCAGACCTTGGTGAATGTGATACAAAAATCAAGGAGCGTGATAGAAAAATAACCAAGCTCTCAGCAGAAATACGTAGGCTCACAGCTCTGCTTGGGGACCGAGACAAACAGCTTCGGAAACTAACTGCAGAACTTGCAAAGTGTGATGCAACGATTAGATTATTCACGGTGGAACTTGGAGAGCGTCATACAAAAATAGGGGAACTTACTGCAGAAGTCGGGGACTACGATAGGCAACTTCGGAAACACGCAGCAGAACTTGTGGAACGGGATGAAAAAATCAGGGAACATGAAGCAGAGATAAGGCGTCTTACTGAGCTGCTAGAGAATCGTGATTTGGAAGCCCGAAACCAGGATGCCCTTATCAGAAAATTGACTGAAGAGCTTGAAGAATTAAAAGGTCTGGAAGCTGATGAATCCTCTGAGGACCTTGAGGAGAGTGATCTAGAGAGGGATGAACTCACCGAAGAACTTGATGAAATCACTGAAGAACTTGGGGAACGTGATAAAAAAATAGAGGAACTCACTGAAGAGCTTGCCAAGCAGACTGCCCAGATTGATGAACTCACTACAGAGCTGGGAGAACGTGATGCCAAAATCGATGAACTGTCTGCAGAAATGGAAAAACGCGATAGGAAAATTGACGAACTCACTGCAGAGCTTGAGGAATACAAAGCAAAAATGAGAAAATGCATGGAAGAGCATAGGAAAGAGGAAGAAAAATTGGCAAACGTGACGCTGGACCCCGAGACAGCCCACCCTCGCCTCATCCTGTCCAAGGACCAGAAGAGCGTGCGATGGGAATACATGCTGCAGGAGTCACCCGACAGCCCCGAGCGCTTCGACGCCGATCCCTGCGTGCTGGGTTGCGAAGCCTTCACCTCTGGGCGTCATTACTGGGTGGTGGACCTGGCAGAAGGGCAGTACTGCGCCGTCGGGGTCAGCAGAGAGTCTCTGCCAAGGAAGGGACCCGTTAGTTTTAATCCCGAAGAAGGGATCTGGGCCGTGCAGCAATGGGGATTCAAGAACCGAGCCCTCACCTCCCCTCCGACCCTCCTGAACCTGCCACGGGTCCCCAAAAAGATCCGCATCTCTCTAGACTATGAGTGGGGGGAGGTGGCGTTTTTTGACGTTGAGAACAAGGTCCCCATCTTCACTTTTCCTCCAGCCTCCTTCGCCGGGGAGCGGATCCGGCCTTGGTTCTGGGTGGAGCTGGGCTCCCTCTCGCTGGTCCGATGA

>TRIM208_NCBI_GeneID:110354628

ATGGCTTCAGCTGCTTCTCGAGCAAGGGAAAGGGGAGAGGAAGGCGCCCACCACATCGCCCCGAAGCATCGGCCAGATCCTGAGCACCTGAGCTGCGGAGGTGGCACAGCCAAATCCTGTCCAGAGCGAGGAAGGCTCGGAGATGCCGAGGCCATCAGCCACCGAAACCACCTCAGGAAGGGGAATTTCCAACCAAAACTCCACCTGGAGCATTTAGCAGAGAAGCTGAAGCTCTTGGGGCTGGAAGGAGGTGGAGAGGAGGAGCAGCTCTGCTCGTGGCATAAGAGGACGTTTGCCTTCAGGAGGGATGCGAAAGCATCCTGCTGCGGTGCACCAAGGGCTTGTGGAGAAGCCCACAGAGAGGAACCTGCCCAGGAGGACAGGGAGCAAATTCACAGAGACCTGGAGAGCCTCAAGAAACATAGGGAAGAGCTTTTGGAACTGAAAGCAAGCGGGGAGAGGAGATGCCAGGGGTATTTGACACAAACAGAGGCCGAGAGGCAGAAAATTGTGTCTGAATTTCGGCAGCTGCGCCGGTTTCTGAAGGACAAGGAGGTCGTCCTCCTGGCCCAGCTGGGGGAGCTGGACAGGGAGGTGATGAGGAGGCAGGAGGAAGAGGAGGCCAAGGTGTCGGGGGAGATTTCGCTCCTCGACATCCTCATCTGGGAGGCGGAGAGGAAACTCGAGCAACCCACGAGCGGATTCCTGCAGAGTGCCAGAAGCACCATGGGCAGGTGGGAGACGAGCAGCACCCGGAGGATGATGGAGACCTTCTCGGACCTCGAGCGGAGGCTCCGCGTCATTTCTCAACAAAATGACATCCTCAGAGAGGCGCTGGGGAGATTTCAAGGTATGGTGGAGCACAACTGGGCTGAGCAGGGGTTGTTTTTTCTGCTTGGGGGGAGGCAAATCTGTCTGCTCCTTCCCTACTGTAACATCCTAAAAATATGTAGAAGCAAGCAGCTGGAGATCATGACATCCAACGTTGTTCTTCATCCCCTAGACATTTTACCCTCTGAACTGGAGAAAGAAGTAGAACCATCTCAGGAGGAGAGGGAAAAGCAGCATTTGTCACTCTGGACCCTGATACTGCCCACGCGAGCCTCGTCGTGTCCCGGGACCGCCGGGGGGTGA

>TRIM213

ATGGGGGGGGTCCCAAAGCCAGGAGGGGGTCCCGAAGTCACAGGAGTGTCCCGAAGGCACGGAGGGGAGGTCCCACAGCCGTCGGGATCTCGCAGCTGTGAGGCGCCGCTGCCTCCCCGAGCTCCCCTCCCGGTTCCGAAAGCGAAAGAGCTGCAGAGCCAAGCCGGGAGCCGCACCCTGGGCCCCTCGGCCATGGCAGGCAGCAGCGAGGCGGCGGCGGAGCTGCAGGAGGAGGCGACCTGCGCCATCTGCTTGGATCTGTTCCGCAGCCCGGTGATGCTGGACTGCGGACACAACTTCTGCCAGGCCTGCATCGGCCTGTGCTGGGCGAGATCCGCTGGGGCTCCCTCTTGCCCCCAGTGCCGCCAGGCCCTGCCCAGCCGCAGCCTGAGGCCCAACCGGCAGCTGGGCAACATCGCGGCCAGGCTCAGGCGGCTTGGCCAGACTGTGGAGCCCCGCCAGGAGCAAAACCAGTCTGAGCTGGAGAGCCTCAGGAGAGAGAGAGGAGAGCTGGAGGAACAGCTAAAGAAGGAGCGATGTACCTGCCAGGGCTATCTGGACAAAGTAAAAGCAGAGAGGCAGAAGATTGAGTCTGAATTCAAGCAGCTGCACCAATGCCTGGAGGAACAAGAGTGCCTGCTGATGGCTCGGTTGGGAGAGCTGGAGAGGCAAATTGAAACAAGAGTGAAGGAAAAGGCTGATAAATTGTCTAAGAGGATTTTTCATCTGGATGGCCTGATCAGGGAGAAGGAGGAGTCTCAGCTCTCAGGATGTGAGTTCCCACAGGATACTGGTGACATTCTGGGCAGGTGCGAGAAGGGAAAATTTCAGCAAAAGGAGTGGACGAGCCAAAATCTGGAAAAGGCAGCTGGTGTCTGCTCAAAAAAACCTCCTGAGCTAGAAGAGACAGCAAGGAAACTCCAAGATGCTTCAACAACTGCTCTGAGGGAGGAAGGGGAAGAGTCACAGGGCCTGTACACAAAAGTGAACGTGACTCTGGATCCAGACACAGCTCAGTCACGGCTCATCTTATCAGAAGATGGGAAGAGTGTGATGCAGGGAGCCACGCAGCAACACCGGCCTGACAGCACGAAGCGGTTTGACCCATGGCCATGCGTGCTGGGCTGCGAGGGATTCGACTCAGGGCGACCGTGCTGGGAGGTGGAGGTGGGCAGTGGGTCCTGCTGGGCTGTGGGGGTGGCCCTGGAGTCTGTGAGGAGGAAGGGACCGATTGACATGAACCCCGTGGGGGGGATCTGGGCAGTGGGGCAGTACAAGGAGAAGTTCCAGGCTCTGACTTCCCCCACTCCCACCCCTTTCCTCCCCAGCATGGTCCCCCGCAGGGTACGGGTCTGTCTGAACCATGCAGAGGGGCGGGTGACGTTTGTCAATGCAGACAACGAGGCTACAATTTTCACTTTCCTGCAAGCCACGTTTTCAGGAAAGCGAATCTACCCCTGGTTCTGGGTGGGGAAGGGGTCCCAGCTCAAACTGTGA

>TRIM13

ATGGAGCTCCTGGAGGAAGACCTCACCTGTCCCATTTGCTGCAGCCTGTTTGATGATCCTCGCGTCCTGCCCTGCTCGCACAACTTCTGCAGGAAGTGTCTGGAAGGCATCCTGGAGGGCAACGTGCGGAACGTGTTGTGGAGGCCGGCCCCTTTCAAGTGCCCCACGTGCCGGAAGGAAACTCCGGTGACGGGAGTCACCAGCTTACAGGTCAACTATTCCCTGAAAGGCATCGTGGAGAAGTACAACAAAATCAAGGTGGCTCCCAAAATGCCCGTCTGCAAAGTGCACAGCGGGCAGCCCCTCAACATCTTCTGCCGGACGGACATGCAGCTGATCTGCGGGGTCTGCGCCACCCGCGGCGACCACACGAAGCACGTCTTCTGCTCCATCGAGGAAGCCTACTCCCAGGAGAAGCGGGCTTTCGAAACCCTCTTCCAGGGCTTCGAGACGTGGCGCTGCGGGGACGCGCTCTCCCGCCTGGATACGTTGGAAACCAGTAAGCGCAAAGCCCTGCAGATGCTGACCAAGGATTCCGACAAGGTGAAGGAGTTCTTCGAGAAGCTGCAGCACACGCTGGAGCAGAAGCGCAACGAGATCCTCTCCGACTTCGAGACCATGAAGCTCGCCGTGATGCAGGCCTACGACCCGGAGATCAACAAGCTGAACGCCATCCTGCAGGAGCAGCGGATGGCTTTCAACATCGCCGAGGCCTTCAAGGACGTGTCCGAGCCCATCATTTTCCTGCAGCAGATGCAGGAGTTCAGGGAAAAAATCAAGGTGCTCAAAGAAACCCCGCTACCTTGTTCCAACGTGGACGTCAGCCCTACGATGAAGAGCTTCGACACCAGCCAGTGGAACGGGATCAAACTGGTTGATGTGGACAAACTGGCCTTGCCTCAGGAAAACAGCACCCTGAAACTGAAGATCCCCTCGGTCTTCTCGCGCCGGTTGATAGTAACCACTCTTATTTGCTTGCTGCTTCTTGCTGTCACCAGAATGTCCTTTGTGGAGTCTGTCGTTGACAATCTCCAGGGCTGGAAGTCTCAGCTCTTTACAATTAGCTTATCTTATTTGGCAGATACGGTGGAGATAGCAGATCACGCCGTCTTTTACTGGGAGCAGATGACAGATGGAGCTTCGCTTCTGAGCGAAAAGTGTAAAAACTACACGTTGGTCGTATTGGATAACGTGGCGCAGTTCGTCTGCAAATATAAACTGTTGTGA

>TRIM14

ATGGCGCTGGGGGAGCCGCGGCGGGGCTGCGGGGCACACGCGGGGCGGCCGCTGGAGCTGCTCTGCGAGGACTGCGGCCGCTGTGTCTGCGCCCTCTGCCCCGCGCTGGGGCCGCACCGCGGGCACCGGGCATGCCTGCTGCACCATGCCGCCCGGCACCGGCAGGAACTCCTGACGCTGTGTTTGAAGGATCTAGAAGAGAGAAAGGAACAAGAGGCTGGTAACAGAAGGTGTATAGAGCAAGCTGCTAATGATCTGAAGGCACACGCTGCTATGACCAAAAAGCAGCTGTCAGACAGAATGACTGAGCTCCAGTTACTGCTTCGCGAGGAGGAGAGCCTGGCGAGAAATTTAATTGACGAAAAGACTCAGCAAGCCCTGGAAGCACATGGTCAGCAGATGGAGTCCTGTCAAGAAAAGCTTGCAGCCCTGGACACCTTTTCATATCGAATCAGAGAAATGCAGCAGAACAATGATATCATTCAGTTTCTGGAGAAATCCATAGAAATCGAGAAGGAACTGCAGGAGTCAAAGAGCCAGCTCGAACAGTGTCACCCGATACCTCTCTCATTTGAGCATGTGCTCAACTACTACAAGCATCTCATGACAGGGCTTCAGTCCGTTCTACAGAAACCACTAGAGGTCCGGCTTAAAGAAGACGTTTTCAGCAGCCTGAACGCCACCACAAAGAAGGAGCCTGGAACAATGCTGAAAACCATGTCTCCTGTCGATCGGTTGCTTTTCTTAAAACATGCAAGATCGCCAACCTGGGAATACGACAGCCTTCATCCGAGGCTGAAGTTGTCTGACGACCGTCTAGTGGTAAGCTGCAACTGGAGGAGGATATTTTACCCCTGTGGCCCCCAGCGGTTCGATAAATTGTGGCAGGTGCTAAGCAGGGATGGGTTCCTCTCCGGGAGCCATTACTGGGAAGTCGACCTCCTTCAGGCTGGAACTGGGTGGTGGATCGGCGCAGCCTACCCGTCCATCGGCAGGAAGGGAGACTCTGAAACCTGCCGCCTGGGCTGGAATCGAGCATCGTGGTGCATCAAAAGGTTTGATTTCGAATACTGGGCATTCCACAAGGGGGAGAGGATCCCTCTCACAGTAGAAGATGACCCTGACCGCGTTGGTGTTTTTCTGGATTACGAGGCAGGGATCCTTTCCTTCTACAACGTTACGGATGGCATGGCTCACCTGCACACCTTCCGCTGCAAGTTCACAGAGCCAGTGTACCCAGCCTTGAGGCTCTGGGAGGGGTCCATTGGCACATGCAAGCTGACGTAA

>TRIM18

ATGGAAACACTGGAGTCGGAACTGACCTGCCCTATCTGTCTGGAGCTGTTTGAAGACCCGCTGCTGCTGCCCTGCGCTCACAGCCTCTGCTTCAACTGCGCGCACCGCATCCTCGTCTCCCACTGCGCCACCAACGAGCCGGTGGAGTCTATCACCGCCTTCCAGTGCCCCACCTGCCGCTATGTCATCACCCTCAGCCAGCGTGGCTTAGAGGGGCTCAAGCGCAACGTCACCCTGCAGAACATCATCGACCGGTTTCAGAAAGCCTCGGTGAGCGGGCCCAACTCCCCCAGCGAGACCCGCCGGGAGCGGGCTTTTGACAGCAACAGCATGTCGTCCTGCGAGAAGGTCCTCTGCCAGTTCTGCGACCAGGACCCTGCCCAGGAGGCGGTGAAGACCTGTGTTACCTGCGAGGTCTCCTACTGCGAGGAGTGCCTGAAAGCCACTCACCCCAACAAGAAGCCCTTCACTGGCCACCGGCTGATCGAGCCCATCCCAGACTCTCACATCAGGGGATTAATGTGCTTGGAGCACGAGGACGAGAAGGTTAACATGTACTGCGTGACTGATGACCAGCTGATCTGTGCCTTGTGCAAGCTAGTCGGACGGCACCGTGACCATCAGGTGGCAGCTTTAAGCGAGCGCTATGACAAGTTAAAGCAAAATTTGGAGAGTAACCTCACCAACCTTATTAAGAGGAATACTGAACTGGAAACTCTTTTGGCGAAACTCATTCAGACCTGTCAACACGTAGAAGTAAATGCATCCCGCCAGGAAACCAAGCTGATGGAAGAATGTGACCAGCTCATTGAAATAATCCAGCAAAGACGACAAATAATTGGAACCAAAATCAAGGAAGGAAAGGTGGTAAGGTTGAGAAAACTGGCTCAGCAGATTGCAAACTGCAAACAGTGCATTGAGCGCTCAACATCCCTCATCTCTCAGGCTGAGCAGTCTCTGAAGGAGAATGATCATGCTCGCTTCCTGCAAACTGCTAAAAACATCACTGAAAGGGTTTCCATGGCAACCGCATCCTCCCAGGTTCTAATTCCTGAAATTAATCTCAATGATACTTTTGATACTTTCGCACTAGATTTTACCAGGGAGAAGAAATTATTGGAATGCCTTGATTATCTTACAGCTCCCAACCCTCCCACCATTCGAGAAGAGCTCTGTACAGCTTCTTATGATACTATTACTGTCCACTGGACATCGGATGATGAATTCAGCGTGGTCTCTTATGAGCTGCAGTACACCATCTTCACTGGACAAGCTAATGTTGTTAGTTTATGTAACTCAGCCGACAGCTGGATGATTGTTCCCAATATCAAACAAAACCACTACACCGTGCATGGGTTGCAGAGTGGCACTAAGTACATCTTCATTGTTAAGGCCATTAATCAGGCTGGCAGCAGAAACAGTGAGCCCGGCAAGCTCAAGACAAACAGTCAGCCATTTAAACTGGACCCCAAATCTGCTCACAGAAAATTGAAAGTGTCTCATGATAACTTGACAGTGGAACGTGATGAAACCTCCTCCAAAAAGAGTCATACACCAGAGCGATTCACAAGCCAAGGGAGCTACGGAGTAGCTGGCAATGTGTTCATTGACAGCGGACGGCATTACTGGGAAGTGGTTATTAGTGGCAGTACATGGTATGCCATTGGTATTTCATACAAGTCAGCCCCAAAGCACGAGTGGATTGGGAAGAACTCTGCCTCCTGGGTGCTCTGCCGCTGTAACAACACGTGGGTGGTGCGACACAACAGCAAGGAAATCCCAATAGAGCCCGCACCTCACCTCCGCCGTGTTGGCATTTTGCTGGACTACGACAACGGTTCCCTTGCCTTTTATGATGCCTTGAACTCCCTGCACCTTTACACCTTTGACATTACATTTGGGCAGCCCGTGTGCCCCACATTCACTGTGTGGAATAAGTGTTTGACCATTATAACTGGCTTGCCTATCCCTGACCACTTAGACTCCTCTGAGCAGCTGGCATGA

>TRIM19.1_NCBI_GeneID:113844857

ATGCCTGGCAGCACCGAACCCCCCCGGCCCTCTGGGACCCCTGACGCCGGCCCCGCTGCCTCCACGGAGCCTTCAGTCCCCATGGAGCCGGCACCCCCCCGGCCACAAGAGCAGGAGGAAGAGGAGGATTTCCAGTTCGTCCTGTGCGAGGGCTGCCGGCAGGAATCGCCCAGCCTGAAGCTCCTCACCTGCCTGCACAGCCTGTGCCTGGGCTGCCTGAGCGAGAAGAAGCCGGTGGGGCAGTGCCCCGTGTGCCAGGAGCCCATCCCGCAGCCCAACGGCATCCCCGAGGTGGACAACGTGCTCTTCGCCAGCCTGCAGGCCAGGCTGCGCGTCTACCGCAGGATCGTCAGCGGGGCGCTGAGCTGCAGCCGCTGCCGCAGGGAGCCGGCGGCCGTCTGGTGCTCCGAGTGCGAGGAGTTCCTCTGCCCCGGCTGCTTTGAGGACCACCAGTGGTTCTTCAAGAAGCGGAGCCACGAGGCCAGGAAGGTGGAGGAGCTGCGGGCCGAGTCGGCGCATCGCTTCCTGGAGGGCACCAAGAAGTCCTGCACCCTCTTCTGCTCCAGTCACGGCCACACCGAGCAGGGCCACATCACCAGCATCTACTGCAAGAAGTGCGAGAAGGCGCTGTGCTGCTCGTGCGCCCTGCTGGACGCCCAGCACTCGCCTTTCTACTGCGACATCCGTGCCGAGATCCAGCGGCGGCAGGAGGAGCTGGCGGCCGCCGGGCGGGAGCTGGCGCGCCGGCGGGGCGGCTTCGAGGCGTCGCGCGCGGCGCTGCAGGAGGAGGCCGCCCGGCTGGAGGCGGCGAGCGGCGAGACGCGGGAGCTGATCCGGCAGCGCGTGGAGCAGCTGGTGCGGCTGGTGCGGCGCGAGGAGGCCGAGCTGCTGGGGCTGGTGGAGCGGCGGCGGGAGCAGGGCCGGCGGGAGCTGGCGGGGGAGCTGCGGCGCGTGGAGGGCGTGCTGCGGCGGATGGAGGCGGGCGAGCGGCTGGTGGAGAAGATGAGGCTGTACGCCACGGAGCAGGAGGTGATGGACATGCAGCCCTTCGTCAGGGAGGCGCTGCGGGAGCTGCAGCGGCTGCGGCCACCGGTGGCCGGGGGCCGAGCGCAGCACGGGGACTTCGCCGAGTGCCGCGCCAGGCTGCAGGCGCTGGCCGAGCGCGTCGAGGCGCACGCAGGTACCTCTTCCCAGGCTGTCCCCGTGGTCGAGGTGGCCCTGGAGAATGACCAGCAAGAGGAGCCCACCCAGCGTGGGAGCCCAGGCATCGTGCCCACCTTCACCATCAGCCTCGGGGACATGCAGCTCCCCACTGCCACTGTACGGTGTAAGCGCTGGCGGCCCCAGGTGGAAAGGGGCAGCCAGGCGTCACCCAAGGTGCTGAAGCTGGAGCACAACACCACGGCGGATCCCAGTGAGCCCAGTTCAACCCAGCGGGACAGCAGGGGAGAGCCCAGCACCTCCGCCACCAGCCACAACTGCAGCAGCGTCCCCAAGGCTGGCAGGAGCCACGCTGATGATGCAGAAGACAACAGCATCATCATCAGCTCAGAGGACAGCGAGGAGGACACGGTGGTGAGCGTGACGCCGGACCTGCCTCCCTGCTGA

>TRIM19.2_NCBI_GeneID:101796105

ATGGCGGGCAAATATCCCCTCTTTGGGGGTCATCCCCTCTTCGGAGGTCGTTCCCTGTTGGTGGTGGTCCCCTCCTTGGTGGTGTCCCCCCCTTGGTGGCAGTCCCATAGCTGCCAGCGGCTGCTCCCAGCACACTGCTGGGTGTTGGATGGGATGGATGGGAAGCAGGGCACAGCCCCCCCCAGGACAAAGAGGGCCATGGAGATGCTCTGCAGAGCTCCACGTCCCCTCCAGGCCCCCTGTCCCCCAGGAAAAGCCCCTTTGGTGGCAGAGCCCTTCCTGCCAGCCATCAGCAGAGGGCTCTGCGCTGCCGGCCTGCCCACCTCGAAGGTGGAGCCTGGCTCCAGCGCAGGAGAAATCGAAAGTGGGCTGAAGGAGCAGAAAGAAGGAGCAGAAGGAGCAGAAGCCAACGGGAGGCTCGGTGGCGAGCTGGGGGTCCCTCACTCCACCATGGCCAGCCCCGACCAACAGCAGCAGCAGCAGCAGCAGCAGCAGGAGGAAGAGGAGGATTTCCAGTTCGTCCTGTGCGAGGGCTGCCGGCAGGAATCGCCCAGCCTGAAGCTCCTCACCTGCCTGCACAGCCTGTGCCTGGGCTGCCTGAGCGAGAAGAAGCCGGTGGGGCAGTGCCCCGTGTGCCAGGAGCCCATCCCGCAGCCCAACGGCATCCCCGAGGTGGACAACGTGCTCTTCGCCAGCCTGCAGGCCAGGCTGCGCGTCTACCGCAGGATCGCCGGCGGGGCCGAGCTGCTCTGCGACAACTGCAGGAGGGAGGGCGAGTACTGGTGCTCCGAGTGCGAGGAGTTCCTCTGCACCACCTGCTTCGAGGCCCACCAGCGCTACCTCAAGCGGGAGAGCCACGAGGCCAGGAAGGTGACGGACATCAGGGCGGGGGCACTGAAGGATTTCCTCCAGGGCACCAGGAGGACCGGCAGCTTGGCCTGCTCCAACCCCACCCACAAGAACCAGACCCTAAGCATCTACTGCAAGAAGTGCGAGAAGCCGGTGTGCTGCATCTGCGCCCTGCTGGACACGCAGCACGCCGGGCAGCACTGCGACATCGGCGCCGAGATCCAGCGGCGGCAGGAGGAGCTGGCGGCCGCCGGGCGGGAGCTGGCGCGCCGGCGGGGCGGCTTCGAGGCGTCGCGCGCGGCGCTGCAGGAGGAGGCCGCCCGGCTGGAGGCGGCGAGCGGCGAGACGCGGGAGCTGATCCGGCAGCGCGTGGAGCAGCTGGTGCGGCTGGTGCGGCGCGAGGAGGCCGAGCTGCTGGGGCTGGTGGAGCGGCGGCGGGAGCAGGGCCGGCGGGAGCTGGCGGGGGAGCTGCGGCGCGTGGAGGGCGTGCTGCGGCGGATGGAGGCGGGCGAGCGGCTGGTGGAGAAGATGAGGCTGTACGCCACGGAGCAGGAGGTGATGGACATGCAGCCCTTCGTCAGGGAGGCGCTGCGGGAGCTGCAGCGGCTGCGGCCACCGGCGGCCGGGGGCCGAGCGCAGCACGGGGACTTCGCCGAGTGCCGCGCCAGGCTGCAGGCGCTGGCCGAGCGCGTCGAGGGGCACGCAGAAGCTGCCCCTGCTCCAGCCACAGAAGACTCCCACCAGGCCCCCTCTACCTCCACTCCTGCCAAGAGGAAGACGGACAAGGACACCAACACGCTGCCATCCCCAGTGAAGGTGATGAAGGTTGAAGAGGACGATGATGGATGGAACATGCTGGCAGAGCCGCAGAGGCTGAGCTGCGAGGAGCAGCCTGGGACCAGCTTTTTGAGGCTCGCCATGGATGACAACCTGCTGGAAGGCATGCTGGATGGCAACGGGGGGCTCTGCGGCTCGGATAGCAATAATCCCAGCTTGGAAAGCGCTGAAGAGGACAGCGTGGATGAGGACTCCAAGGACTCCAGCCTGCTGGAAGGTCTCGGTAACATGCTTGATGACGGCACCAGCGAAGAGCACCTGGGCTTCCCCATCCGCCTCCAGAACACAATGGACACGAGACAAGGGTCCCTGGTCTTCTTTGACGTCAAGATTTTGAAAAACGAGATCATCCAGATGGCAGTGATCGACGGGGAGCAGATACTGCCCGTCCTCATCCAGCCGGTGAAATGTTTGCCCAGCCTGATGGCCAAAAACAGCGTCTGCGAGGTCGGCCTGAGGAGCCTGCTTGGCCACCTCTACGCCGTCCACCAGCCCATCCTGGGTGGGTTCAGGTTCTGCTCGCTGCCCCTTCCCACCCTCCTGGAGGCTCTGACGGTCCTGGGCAAGAGAGAGGAGTTCAGCGCCGCCGTGTACGGCTTCCTGGACATCCTGCCCCTGATAAAGGAGAAGGTCCCCGAGAGGGACAACTACAGGCTGAAGAACTTGGCCAGCAGCTACCTGTGGCGGGACCTCAGCGACCACAGCGCCATGGAGAGCGCCAGGGCCGTGAAGGACCTGTGCGAGGTGCTGGACATCGACCTGCTGAGGACACCCAGGCTGGTCCTCAGCCACGCCAGCCTGGAGTGCTGGGTGTCCCTGCAGCCCCTGCTGGAGGAGAAGCTCCTCAACAAGGCATCAGCCCAAAGGCTGGCATCCTGCAACGTCGGCCTCTCCGAGCTGTGGTCCTGCCACCGGCACGACCCCGGGCAAGGTCTCCAGAAGCTGCGTGCTCTGCTCAACGCTCACCGGCACGGCTCCGAAAAGAAAATCCGCACCCTGAGCAAGGTGCAGCTCTACTTCCAGCGCCAGCAAGAGGACAGCCGCGAGGCCCCGGCGGGCAGCAATGTGCCGAAAGATGTAAAAAACAAGGAAAACTGA

>TRIM23

ATGGCGGCGCTGGCTGTAAACAGGGCCGGGGCCGCGCAGGATGGCGGCCGCGCCTCCAGCACCGCCGGCAGTAGCAGCCGGGGCTCGGCGGGCGCCGCCGTGAAGGTTCTGGAGTGTGGAGTCTGCGAAGATGTGTTTTCTCTGCAAGGAGACAAAGTCCCCCGGCTGCTTCTGTGTGGCCATACCGTCTGCCATGACTGCCTCACCCGGCTTCCCCTTCACGGCAGAGCAGTTCGTTGTCCTTTTGATCGACAAGTTACTGAATTGGGAGATTCTGGTGTCTGGGGCTTGAAAAAGAATTTTGCTCTGTTGGAACTCTTGGAACGTTTGCAGAATGGACCTGCTGGGCAGTGTGGGACAGCAGAAGAAGCTATCGGTCTCTCTGGAGAGAGTATCATTCGTTGTGATGAAGATGAAGCCCATGTTGCATCTGTTTATTGCACTGTTTGTGCCACTCACCTGTGTGCAGACTGTTCCCAGCTTACTCATTCTACAAAGACACTAGCGAAACACAAGCGTGTACCTCTTGCTGATAAGCCTCATGAGAAGACCATGTGTTCCCAACACCAAGTGCATGCTATTGAGTTTGTCTGTTTGGAAGAGGGCTGTCAGGCCAGTCCTCTTATGTGTTGTGTCTGCAAAGAATATGGGAAACATCAAGGTCATAAGCATTCTGTCTTGGAACCAGAAGCAAACCAGATTCGTGCATCCATTTTAGACATGGCCCACTGCATACGAACTTTCACAGAAGAAATCTCAGATTATTCAAGAAAATTAGTTGGAATAGTTCAGCATATAGAAGGAGGAGAACAAATAGTTGAAGATGGAGTTGGAATGGCCCATACAGAACATGTGCCAGGGACTGCAGAGAATGCTCGCTCATGTGTCCGAGCCTATTTTTCTGATCTTCATGAAACCCTTTGTCGTCAGGAGGAAATGGCTCTTAGTGTTGTTGATGCTCATGTAAGAGAGAAGTTGATTTGGCTTAGGCAACAGCAAGAAGACATGACCATCCTATTGTCACAGGTTTCAACAGCTTGCCTTCATTGTGAAAAGACTTTACAACAGGATGACTGTAGAGTAGTATTGGCTAAACAGGAAATTACAAGATTGCTCGAGACATTACAGAAACAGCAGCAGCAATTTACGGAACTTGCAGATCATGTACAGCTGGATGCTAGCATTCCTGTCACTTTTACAAAGGACAACAGGGTACATATTGGACCAAAAATGGAAATTCGGGTTGTTACTCTAGGATTAGATGGAGCTGGCAAAACAACTATTTTGTTTAAGTTAAAGCAAGATGAATTCATGCAGCCAATTCCAACAATAGGTTTTAATGTTGAAACAGTAGAATACAAGAATCTGAAGTTTACTATTTGGGATGTAGGAGGGAAGCACAAGTTGAGGCCCTTGTGGAAGCATTATTATCTCAATACACAAGCGGTTGTGTTTGTTATTGATAGCAGTCACAGAGACAGGGTCAGTGAAGCACACAGTGAACTTGCAAAATTATTAACAGAAAAGGAGTTGCGGGATGCCTTGCTCTTGATCTTTGCTAATAAACAGGATGTAGCAGGCGCCCTTTCAGTGGAAGAAATTACGGAACTGCTGAGTCTCCATAAGCTCTGCTGTGGCCGTAGCTGGTATATCCAGGGGTGTGATGCCCGAAGTGGTACAGGACTTTTTGAAGGATTAGACTGGCTTTCAAGGCAGTTAGTAGCTGCTGGTGTTCTGGATGTGGCTTAAT

>TRIM24

ATGGAGGAGGCGGCGGCGGCTGTGGCAGCGGCGGCAGCAGCGGCAACAGCAGCAGCGGCAGGGGGGGCCCTGCCCTGCCCGGTTGATCGTGGCGGTGGGGCGGCGGCGCTGAGCGGGGAGAACGAGGCCGAGAGCCGGCAGGGCCCGGCGGAGCGCGGCGGGGAGGCGGCGCCGCTCAACCTGTTGGACACTTGCGGCGTGTGCGGGCAGCCCATCCAGAGCCGCCGGCCCAAGCTGCTGCCCTGCCTGCACTCCGTCTGCCTGCGCTGCCTGCCGCCGCCCGACCGCTACCTCATGCTGCCCCCCGCCGGCCCCCCCGTGCCCACCGCCGCCCCCCACAAGGAGCCGCAGCCGCCGGCGCCCCCCTCCCCGCCCGGCTCCTCGCCCCTGCACTGCACGCCCGTTGGTGTTATCCGCTGTCCAATTTGTGGTCAGGAATGTGCAGAGAGACACATCATAGATAACTTTTTTGTGAAGGATACCACTGAGGTTCCCAGTAGCACAGTAGAAAAATCAAATCAGGTCTGCACAAGCTGTGAAGATAATGCAGAAGCTAATGGGTTTTGCGTGGAATGTGTGGAATGGTTGTGCAAGACCTGCATCAGAGCTCACCAGAGGGTTAAATTCACAAAGGATCATACTGTAAGACAGAAAGAGGAAGTGTCTCCAGAGGCGGTTGGTGTGACCAGTCAACGACCTGTGTTTTGCCCTTACCACAAAAAGGAGCAGTTGAAATTATATTGTGAAACCTGTGACAAGTTGACCTGCAGAGACTGTCAGTTATTAGAACACAAAGAGCACAGGTACCAGTTTATAGAAGAAGCTTTTCAAAACCAGAAAGTGATCATTGAGACGCTCATCACTAAATTGATGGAGAAGACTAAGTATATAAAATATACAGGGAAGCAGATTCAAAACAGAATTCTTGAGGTGAATCAGAATCAAAAGCAGGTGGAACAGGATATTAAAGTTGCCATATTTACACTGATGGTAGAAATAAACAAAAAGGGAAAAGCTCTGCTGCATCAGTTGGAGACTCTGGCAAAAGAGCATCGGATGAAACTTCTGCAGCAACAACAGGAAGTGGCAGGTCTCTCTAAACAGCTGGAACATGTCATGAATTTTTCTAAATGGGCAGTTTCCAGTGGGAGCAGCACAGCACTACTGTACAGCAAACGCTTGATTACGTATCGACTACGCTATCTTCTCCGAGCGAGGTGTGACGCTTCACCAGTGACTAACAATACCATCCAGTTTCACTGTGATCCTAGCTTCTGGGCTCAAAATATTTTTAATCTAGGTTCTCTGGTAATAGAAGATAAAGAAACACCACCACATATGCCCAAGAGCCCTGTGATGGAAACAAATCTGCAGCCAGCAGGCAACTTACCCTCAAACCAGCTGTCTAAGTTCCCAACGCAAATAAACTTGGCTCAGCTCCGACTCCAGCACATGCAGCAACAGGTTATGGCTCAGAGGCAACAAGCTCAGCGCAGAGCAGGTCCAGTAGGTTTACCAAATCCGAGAATGCCTGGAGCTATGCAACAGCCTCCTGCTTCTCATCAGGCACCTCCGCGCTTGATTCATTTTCAGAATCATAACCCTAAGTCCAATGGTTCAGCTCCTCCTGCACAACAGATGAGATTTCCCCCAAGTCAGAACCTACCAAGGCAAGCAATAAAGCTCAACCCATTGCAGATGGCATTCTTGGCACAGCAAGCTATAAAACAGTGGCAGGTTGGTAATGGACAAACTTCAACCGCCACTTCAGCTGCAAGCAACATAACATCTACTCCATCCAGTCCCACAGTCACTAGTGCTGCGGGATGTGATGGGAAGACATATGGTGCTCCTGTGATAGACTTGAGTTCTCCAGTGGGTAGCTCCTACAATCTGCCATCTCTTCCTGATATTGATTGTTCAGGAAACATCACACTGGATACCGTTGCAAGGAAGGATGGCACAGCAGAGCAGAGCCAGGCAAAACCCCCTTCAAACAGAACTGTGCAGTCTCCAAATTCATCAGTACCATCACCAGGCCTCTCAGGAGGCGTGAGCGTGACAAACATACACCCTCCAATTCGTTCACCCAGTGCCTCCAGCGTTGGGAGCAGAGAGAGTTCTGGCTCTTCCAGTCGGCCACTGGGAGCTGATTCTACACACAAAGTCCCCGTTGTTATGTTGGAGCCGATCAGAATTAAACAAGAGTCAAGTGCACCAAATGAGAACTTTGATTTCCCAATTGTTATAGTGAAGCAAGAAACAGAGGAGGAATCCCGGCCCCGGAATACCACCTTTTCAAGAAGTATACTTACTTCATTGCTGTTAGATGGCAATCATAATTCCACTTCTGATGAAGCTGTCATAAGAACAGATGCACCAGACAGCACAGATGATCAACCAGGGATACTGCTGGAGAATACAACCACTGGGAAATCAGGATGGATAGGCCCCTCACACACTGGGGAGGGCAGAAAAGAGGATGACCCCAATGAAGACTGGTGTGCAGTATGTCAAAATGGAGGGGAGCTCCTTTGTTGTGAGAAATGTCCCAAAGTATTCCATCTTTCTTGTCATGTCCCTTCGCTGATGAACTTTCCAAGTGGGGAGTGGATTTGTACGTTCTGTCGGGATTTGTCCAAGCCAGAAGTTGAATATGATTGTGATAAACCCGCTCACAGCCCTGAAAAAAGAAAACTGGAAGATACTGTGGGTTTGGCGCCAATAGACCGCAGGAAATGTGAAAGATTGCTGCTATACCTCTACTGTCATGAAATGAGTCTTGCTTTTCAAGATCCAGTTCCTCCCACGGTTCCTGATTACTACAAAATAATCAAAAAGCCAATGGACTTGTCAACCATCAAGAAAAGACTTCAAGTGACCAGTTCATTCTACACAAAGCCAGAAGATATTGTGGCTGACTTCAGACTGATTTTCCAAAACTGTGCTGAATTTAATGAGCCTGATTCAGAAGTGGCTGATGCTGGCATGAAACTTGAAGCTTACTTTGAAGAACTTCTAACGAGCCTGTATCCTGAGAGAAAGTTCCCTGTACAGCCGAACTGCCAGAGTGAAAGAGAGAACACAGAACTTAGTGATGACTCAGATGATGACTTTGTACAGCCCCGGAAAAAACGCCTCAAAGGAGAAGACCGTCAGTTGCTTAAATG

>TRIM25

ATGGCGGCGCTGACCCGAGCCGTGTCCGAGCCGAACCTGGCGGGGCTGGAGGAGGATCTGACCTGCTCCATCTGCCTCTCCCTCTTCGACACCCCCGTGACGGTGCCGTGCGGCCACAACTTCTGCGCCTCCTGCCTGGACCTCACCTGGGCCGAGCTGGATGCCGGCTTCAGCTGCCCTCAGTGCCGGACCACCTTCCCGGGCCGGCCTCAGCTCCGCAAGAACACGGTGCTGTGCCGGGTGGTGGAGCAGCTGCAGGGCTGCACCGCGGCCGAGGAGCAGCAGAAGCAGGAGGATGAGGAAGATGAGGCGGTGGCGGAGGAAGCAGCGTCCCCCGTGTACTGCGACAGCTGCCTGCAGGCTCATGCAGCACAGACGTGCCTGACCTGCATGGCCTCCTTCTGCGCCGAGCACCTGCAGCCGCACCACGACAGCCCGGCCTTCCGCGACCACCAGCTCTGCCCACCCGTGCGTGACCTGCAGCAGCGCAAGTGCTCGCAGCACAACAAGGTCTTCGAGTTCTTCTGCAAGCAGCACGGCACCTGCATCTGCTCCCTCTGCCTCCTCAGCCACAAGCTGTGTAACGCCAGCCCCCTGCAGCAGGCCAAGGCTGAAGCTGAGTCAGCACTGAAGAAGAAACTGACAGAGCTGCATAATCACAGTGAAAAAGCCACTCGAGCGATGAACTCTGTGAAAACAAGCCAAACCCAAACTGCTGAGACAGCTGCCAGAAAGCGAGATTTGATGAGAAACGAGTTCTTGGAAATTAAAGCTTTGATTGAAGAAAAAGAAAACCAGATCTTTAAAGTAATCATGGAAGAAGAAAAAAGAGTTTGCACTAAGTTTGATTACATTTATACTGTTCTGGGAAGTAAGAAGAATGAAATTCAATCTCTCAGAGACCAGATTGAGATGGCACTGACAGAACATGATGACGTTCTGTTTTTAAAGAGAGCAGCAGCACTGCAACGAGCATCAACAAAAGAGGTTTTTGTGCCTGTAATTGAAATGGACCAAAACTTGATACATACTGCTTATCAGTCTGCCATTAACCTTAAAGAAATGGTCAAGCTTACAGTGAGTCAGCCTAAGGAGAAAAAAACAGAAGGATGCTATTGGCCTTCTTGGCCACAAGGGCACAGTGCTGGCTTATGGTCATCCGGCTGTTTACCAGGAACCCCAGGTCCCTTTCCCCTACGCTGGTCTCCAACAGAGAAACAAACAGCTAGGAAAGCTAAGCCCCCTCAAGCAGCTGCACTAAATAGACCTGTTCCTGGGAGAAAGCCGGTTGGACCACAGCGTCCAAACAAAGAGAAAAAACCTTCCCAGGTTCAAGAACCTTTGCAGGAGGAGGCAGATAACCGAGCACCAAACATGGGAGCACCAAGCACAGCAGCAACAACTGCAGGAGCATCAAAAGCTGCAACTGCGGCTAATACAAAAGATCTTATTAGCAGCTTTCTTCAGAAAGACAGAGAGGAGCTTTTGCAGTATGCTGCTAACATCACCCTGGATTTCAACACAGCTCATAACAAAGTGCATCTGTCTGAGAGATACACCAAGATGTCTGTCTCAGACACCCCCCTGAATTATAACCACCACCCTCAGCGTTTCACCTATTGTTCCCAAGTGCTGGGGTTCCAGTGCTTCAAGAGAGGCATCCACTACTGGGAAGTAGAACTGCAGCAGAAAAACTTCTGTGCCATTGGCATCTGCTATGGCAGCATGGACCGGGAGGGGCCAGACAGCCGCCTGGGTAGGAACAGCAGTTCTTGGTGTATTGAGTGGTTTAATTCCAAAATTTCAGCCTGGCATAATGATGTTGAAAAGAATTTACCCAATGTGAAGGCTACCAAGATTGGCGTGCTGCTGCACTGCGAGGGAGGCTTTGTGATTTTCTTGGCTGTTGGGGAGAAGCTTAACTTGATTTATAAATTCAAAACCCAGTTTACTGAGGCACTGTACCCTGCCTTCTGGGTATTTTCAAGTGGCACTGTTCTCTCCCTCTGCCAAATGAAAAAGTAA

>TRIM206

ATGGCCGAGTGCGACCCGCTGGAGAGCCTGCAGAAAGAAGCATCCTGCTCCATCTGCCTGGATTATTTCAGCGACCCCGTCTCCATCAACTGCGGGCACAGCTTCTGCCGCGACTGCATCACGCGATGCTCGGGCAAATCGGACCGGAGGTTCGCCTGCCCTCAGTGCCGTGGGATAGCCCAGAAGAGAAAATTTCGGCCAAACCGGGAGCTGAGGAACCTGGCGGAGATCGCCAAGAAGCTGATCTCGAGGGTCGGCGACGCGGCGCGAGCGGGCGGCGTGTGCCCGAAGCACCAGGAGCCGCTCAAGCTCTTCTGCCAGGAGGACCAGACGGCCATCTGCGTGGTCTGCGACCGGTCCCAGGCTCATCGTGCTCACACCGTGGCCCCCATCGAAGAAGCCGCCCAGGAGTGCAAAGAACATATCCAAAGCAAACTGAAGAGCCTCAAGGATGAAAGAGAGAGACTCCAAGGATTAAAAGTGATGGGGGAGAAGAGAAGCCAGAAGCATCTGCAGCAGGCGAGAGCCGAGAGGTGGAAAATCATGTCGGTGTTCAAGCAGCTGCACCAGTTCCAGGACGAGCAGGAGCGGCTCCTCCTGATGTGGCTGGAGGACACCGAGAAGGAGATAGTGCAGACCCAGAGTGAGAACGACAGGAGGATCTCTGCCGAGATCTCCCACCTGGGCAACCTCATCCGGGAGCTGGAAGGGATGAACCCCCAGCCGGAGAATAAATCCCTGCAGGATGCCAGGAGTGCCTTGACCAGGTGTGACACAAGGGCTTTCCAGCATCTGTCGGAGAAGTTTCCCCGAGTGGAAAAAAGCCTCAAGGATTTATCTCAGAAAAACATCATTCTGAAGGAAGCCCTGAGGAAATTCAAAGAGAGTCTCCCAGTTGAACTGGATGTGCAATGGGCAAACGTGACTCTGGATCCAGACACAGCAAACCCCCACCTTGTCCTCTCCGAGGACCGGAGGAGCGTGAGATGGGACGAAACACCCCAGAATTTGCCTGACAACCCGCAGAGATTTGACACCTACTGCTCGGTGCTGGGCCACGAAGGCTTCACGGCAGGGAGGCACTACTGGGAAGTGCAGCTGGGGAATAGGGGATTTTGGGCTGTGGGGGTGGCCAGAGACTCGGCTTGGAGAAAGGGTTGGATCAGCCTTGACCCTTCCCAGGGGATTTGGGCTGTTGGCATCTGTGGGGACAGGTTTCAAGCCTTCACCTCCTTCGAAACAGTTCAGCCTCTGAATGGGAGGCCAAGGACTATCCGAGTCTCTCTGGATTACGACAAGGGACAAGTGGCTTTCTTTGATGCCGATAATGAGACCTTGGCTTTTGCTTTCACACCGACTTCTTTCAACGGAGAGAAAATCCTGCCTTTCTTCTGGGTTTGGGAGTCCAAGATCCAGCTGGCTCCC

>TRIM205

ATGGCTTCGCAGAGCCCCTCGGAGAGCTTGCAAGGCGAAGCCTCCTGCTCCATCTGCCTGGGCTTCTTCCAAGACCCCGTCTCCATCCACTGCGGCCACAACTTCTGCCGGGAATGCATCACCCGCTGCTGGGAAGGGCTGGAGGCCAACTTCTCCTGCCCCCAGTGCCGGCAGACGGCTTCGCACAAAAGTTTCCGTCCCAGCAGGGAGCTGGCCAAGATCGCCGAAATCGCCCAGCAGCTGAGCTTGCAAGCAGGCAGAGGAGCAGCGGGGCACGAGGGTTGGTGCCAGCAGCACCAGGAGGCTCTGAAGCTCTTCTGCAAGGAGGACCAGCAGCCCATCTGCATGGTGTGCGACCGGTCCCAAGCTCACCGCCTCCACACCGTGCTCCCCGCCGAAGAAGCTGCCCAGGAGTACAAGGAAGAAATCCAGGCTCGCCTCGAGCTTTTAAAGGAAGAGAGAGAAAAATACCTGGAAAGCAGAAAATCCAGAGCAAGGAAAAACTTGCACTTGGAGAAAACCAAAAACGAAGGGAAGAAAATAGTGTGTGAATTCGAGCAGTTGCACCAATTTTTGAAAGACCAAGAGCGCCTCCTCCTGACCCAGCTGGCAGATCTGGACCGGGCCATCACCAGGGTGCAGGAGGAAGCGGTGGTGAAGGTCTCGGAGGAGATGGCCCACCTTGACACCCTGATTTGGGAGATGGAGGGGAAATTCCAGCAGCCGGCGAGCAAATTCCTGCTGGACGTCAGAAGACTCTTGAAAAGCTGTGAGGTGATGAAGTTCAACCCTCCAGTGGAGATTTCCCCCCATCTGGAAAGAAGACTTGAGGATTTTCTTCAGAAAAATGTCCTCGTGAGATGCACGCTGAGGAAATGCCAAGATAGCCTGATGTTTAAATTGCAAGAGCCAACCAACGTGACCCTGGACCCAGCCACGGCTCACCCCAACCTCCATCTCTCCGAGGACCGAAAACAAGTCAGGGGCCAACTGGTGCCCCAGGACCTTCCGGACAACCCAGAGAGATTCGACTTCGAGCCTTGTGTGCTGGGCTGCCAGGGTTTCACCTCGGGGAGGCATTTTTGGGAGGTGGAGGTGGGACAGGGGGGCGTCTGGGCTATAGGGGTGGCCCGAGAGACCGCGAAGAGGAAGGGACCCATGAGCCTCACCCCCAAGGAGGGCATTTGGGCGCTGGAGGCTTATCACTCCCTGACATCCCCCCGTGCTAACGTGCGTCTGAACCAGCTTCCCAGGAAGATACGGGTCTCCTTGGACTACGAAGGGGGTCGGGTGGCATTTTTCAGCTCGGATGATGATGCTCCTATCTTGGTCTATAGCAGGGCTGCGTTCAACGGGGAGAGGGTCCTCCCTTGGTTCAAGATGGGGATGGGGGCCCGCTTGCAAGAAATCACCCAAAACTCATCCTCAGAGGAGCAGTCCATGACCGGGCAGCTCATGTCCCCTCTGGATTGGGTTGGGTTCAGGTCTCCCCTCCGGATTTGTCCT

>TRIM203_NCBI_GeneID:101791534

ATGGCTGCACCGAGCCCTGTCCCCAAGCTCCCCAGCGAAGCCTCCTGCCCAATCTGCCTGGAATATTTCCGAGACCCCGTCTCCATCCACTGCGGTCACAATTTCTGCCGGGCGTGCATCACCCGCTGCTGGGAGTGGTCCACGGCGAATTTCTCCTGCCCGCAGTGCAAAGAGACGGCCCCGGAGAGAAGCTTTCGTCCCAGCCGGGAGCTGGCGAGGGTCCTCGAAATAGCCAAGAGGTTGAGTTTGCAAGCAGCCAGAGGGGATGTGGTGGAGGAGGAAGGATGCGAGAGGCACCGGGAGCCTCTGAACATCTTCTGCAAAGACGACGAAACCTTCATCTGCGTGATCTGCCGGGAGTCCCGGCTGCACCGGGCTCACACCATGCTTCCCGTGCAGGAGGCTGTCCAGGAATACAAGGGACAAATCCAAGCCCACCTGCAAACACTGAAGGAAGACAGAGACAGGCTCCTGGGCTTTCGAGAGGCTGAAATGAGGAGAAACTGGGAGTATTTAGAGAAGACCGAGGCTGAAAGGCAGAAGGTTTTGAGCACGTTTGAAGGGCTGCGTCTCTTCCTGGAGGACCAGGCACGTCACCTGCTGGCCCAGCTAGCGGAGCTGGAGAGGGCCATAGAAAAAATACAGGAAGAAAACATCACCAACCTGACAAAGGAGATCTCGCACCTGGACACCCTGATCCAGGAGTTGGAGGAGAAGTGCCAGGAGCCAGCAAGTAAATTTCTGCAGGACATCAGAGGCACCTTGAGCAGGTTTGGAAAGGAAAACTTCCAGCAGCCCACGTTGCTTCTTCCAGAGCTGGAAAATAAAATCAGTCACTTCAGGGAGAAAAATATTGCTCTAGAGGAGACTCTGAGGAACTTCAAAGACATCCTGATGTTTGAGCTGCCTGAAAAGATGAATGTGACCCTGGATCCAGCCACAGCTCACCCCCAGCTTGCCGTGTCGGAGGACAGGAGGAGCGTGAAGTGGGAAGATGCCCAGCAGGACGCATCCGATGAGGGATTTGGCCCCGATCCTTCTGTTCTGGGCTGCGAGGGCATCACCTCGGGGAGATGCTGCTGGGAGGTGGAGGTGACACCCAAAGGCTCCTGGGCTGTTGGTGTGGCCAGGGAGTCTTTGAAGAGGCGAGAAGAGACCCCTGTGAGCCCTGAGATTGAACTGTGGTCTATGGGTCTGTGTGAGGACCAGTTTTGGGCTCTCACCTCCTTTGAGCGCACGCCGTTATCCCAAATCCAGGTCCCTAGAAGGGTCCGGATCACCCTGGACTATGAGAGGGGTCAGGTGGCGTTTTTTGATGTCGATAGGAAGGCCTTGATCTTCATTTTCTCAGCAGCCTCGTTCAAAGGGGAGAGTGTTCACCCCTGGTTCTTGGTGTGGAGCGAGGGGTCTCGGATCACATTGTGCCCCTGA

>TRIM28

ATGTCGAGCCCGGCGAAGCGCCCGGACGCGGCGGCCGCCAACGGGGGGCCGAGTGAGGGGGCAGTGGTCAACTGCCCGGTGTGCAAGCAGCAGTGCTACCTGCAGGACGTGGTGGAGAACTACTTCCTAAAGGACAACCGCCCCGAAACGGCCCCCGACAGCCAGGCCTCCACCCAGTGTTGCACCAGCTGCGAGGACAACGCGCCGGCCACCAGCTTCTGCGTGGAGTGCTCGGAGCCGCTCTGCGAGACCTGCGTGGAGGCTCACCAGAGGGTCAAGTACACCAAGGACCACACGGTCCGAGCCGCAGGCAATGCCCGGGCCAAGGAGGGCGAGCACGCCGTCTACTGCGCCGTGCACAAGAGCGAGCCGCTCGTCCTCTTCTGCCAAACCTGCGACGTCCTCACCTGCCGGGACTGCCAGCTCAACGCCCACAAGGACCACCAGTACCAGTTCCTGGAGGACGCGGTGAGGAACCAACGCAAGATGCTGGCCACGCTGGTCAAACGCTTGGGCGACAAACACGCCAGCCTCCAGCGCTCCACCAAGGAAGTGCGCAGCTCCATCCGGCAGGTGACGGACGTGCAGAAGCGGGTGCAGGTGGACGTCAAGATGGCCATCCTGCAGATCATGAAGGAGCTCAACAAACGCGGCAAGGTGCTGGTGAGCGACGCCCAGAAGGTGACGGAGGGGCAGCAGGAGAAGCTGGAGAGGCAGCACTGGGCCATGACCAAGCTGCAGAGGCACCAGGAGCACGTCCTGCGCTTCACCTCCTGGGCCCTGGAGAGCGACAACAGCACGGCCCTGCTGCTCTCCAAGAAGCTGATTTATTTCCAGCTCCACCGCGCCCTGAAGATGATCGTGGACCCCGTGGAGCCGCAGGGGGACATGAAATTCAAGTGGGACCTCAACGCCTGGACCAAGAGCGCCGAGAGTTTCGGCACCATCGTTTCGGAGCGCAGCCTCCCCCCGCCGCCCCTCAGCCCCCAACCGCCGGCCGCCAGCCCCACTGCGGGCCCCTCGCAGGGCTCCCCGCACACCACGGTGGTCAGCGAAGGGCAATACGCCCCCAGCCCCCTCCTGCAGCCCCCCGAGGGACCCCAAATTGGGGACAAGGATGGGGGGGGCCCCCCCGGTGACCCCCAAGAGGGTGGGGGGGGGCTGGGGACCCCTCAGTTACCCTCGGGGACCGCGGAACTGGGCTGTCCCAACCTGTCCCCCCCCCACCTTGACCCTCAGGTGACCGAGACCCCCAGGGACGCCGGTGCGGAGCTGGGCAACTCCTCTCCTGAAGCCGCTGTCACGGGAACCAAGCGGAGAAAACAACGTGGGAGCCCCCCCGGGGAGGAGAAGTTCGTTAAGAAGCTGCTCGTTAAGCGCAGCCACCCCCCGGGGGCTTTGGGGAGCCCCCTCCTCCGCAAAGTGCCTCGGGTCAGCCTGGAGCGCTTGGACCTGGACCTGGCGGGGGCCGCTCAGCCCCCCTCCTTCCGCGTCTTCCCCGGCACCACGGCCGAAGAATTCAACCTCATCATCATCGAGAGGGGGGGGGCGCAACCCTGGACCCCCTCATCATCGAGAGGGGGGCGCAACCCTGGACCCCCACCCTGTGGGGATCTGGGCATTTATTTATGGACCTCTGCTCCCAGGGGAGCTGCTCCCCACGGGATCCACTCGATGCCAGCTCCCAGCAGGGAGCGAGCCGGGCGCGGTGGGATCCACCCTCGTCCCCTCCCCGGATTCCCTGAGATCCCTGGGATCCGCTCCTGCCCCCCCAGGCCCTACCGAAATGTCTCCTGCTGCCGCGTCTGCTGCCAGGCGGGCGCCGTGGTGATGTGCGACCTCTGCGAGCGCTGCTACCACCTCGACTGCCACCTCCCCGCCCTCCACGAGGTCCCCGGCCACGACTGGCTCTGCCTCCTGTGCCAGGACCCGGCGCCCCCCGGCGAGGACCCCGGCCCCACGGAGGAGCAGCCCCCCGCCCTCTCCCCCACGGATCAACGGAAGTGTGAGTACGTCCTGCTCCAGCTGCTCTGCCACGAGCCCTGGCGGCCCCTCCACCGCCTCTCCAGCTCCCTGGAAGGCTGCGACGCCATCGACCTGACCTTGATCCGCGCCAAGCTGCAGGGGAAGCTGTCCCCGGGCTACGGCCACCCCGAGGAGTTCGCCCGCGACGTCTGGCGCATGATCCGCCAGTTCAACCGCCTGACGGAGGACAAGGCGGACGTGCAGTCCATCCTGGGCCTGCAGCGTTTCTTCGAGGAGCGGCTCAGCGCCGCCTTCGGCGACCGCAAGTTCTGCGCCGCCCTCGAGTCCCTCGGCCCCCTGGATGGGGCTGAAGTTTCCCAGGCCCCCCCTCCCACCCTCCTGGCACCCTAA

>TRIM29

ATGGAAACGGGGAGCGCAGCAAGGACAAATGGTACCGCCGGCAAGCCAGAGGATGTGAAGAGCCCGTCCGCCCCCAAAAAAGATGAAGAAGTGAAGAAGAACTCGAACCCTGGTGGAGGGGAGAAGGAGCCAATGAAGGGCACTGGCGGTACTTCTCTGGAGACGGGGCAAATCAAGAGCTCCCTCTTCTCTGGGAGTGACTGGAAGAGGCCCATCATTCAGTTTGTGGAGTCGTCCGATGAGAAGAGATCGACCTACTTCAGCATGGACTCGGCAGACTCGAAGAAGATGCAGTACAGCAGCGGACAGATAGGAGACATGAGGAGACCCCCCNTCTCCTTCGCAGATAAAGGCGACCTCAGGAAGTCCCTCTTCTCCTTGGATTCCAAAAAGAGCTTCCTGCCTAACGAAGGGGAAGGGAGGAAGCCGCTGTTCTCCGGCGGGCAGATGGGGGACATGAAGAAGTCTTCCCTGCCTCTGGTGGAGACCGGGGACCTGAGAAGAGCCACCTTCAACAAGGTGCCCGACAGAGCAGCCGGGTCGCGGCCCAGGGTGAAGCTGGAGGATGTGCTGTGCGATTCCTGCATCGACAACAAGCAAAAGGCCGTGAAGTCCTGCTTGGTGTGCCAGGCTTCCTTCTGCGAGCTGCACCTCAAGCCCCACCTGGAGGGAGCGGCTTTCCGGGACCACCAGCTCCTGGACCCCATCAGGGACTTTGAAGCAAGAAAATGCCCTGTGCATGGGAAGACCATGGAGCTGTTCTGTCAGACAGACCAGATGTGCATCTGCTACCTCTGCATGTTCCAGGAGCACAAGAACCACAGCACGGTGACGGTGGAGATCGAGAAAGCGGGTAAAGAGGCTGAGCTTTCACTGCAGAAAGAGCAACTGCAGCTGAAGATCATCGAGGTAGAGGATGAAATGGACAAGTGGCAGAAGGAGAGGGACCGCATCAAGAACTACACCACCAACGAGAAAGCCACAGTAGACCAGCATTTCAAAGAGCTGATCCGTGACCTGGAGAGGCAGAGGGATGAAGTGAAGGCTGCCCTGGACCAGAGGGAAAAGATTGCATCAGAGAACGTGAAGGAGATTGTGGATGAGCTGGAAGAGAGGGCGAAGCTGCTGCGGGAGGACAAGGAGAACAGGGAGCAGATCCACCAGATCAGTGACTCCGTGCTCTTCCTCCAGGAGTTTGGGGCTTTGATGCGGAACTATGTCCCCCCTCCATCCCTCCCGACATACAGCGTGCTGCTTGAAGGGGAGAGCATGAGCCCCTCTATGGGGCTGCTCAGAGATGACCTCCTCAACGTCTGCATGAGGCACGTGGAGAAGATCTGCAAGGCAGACCTGGGCCGCAACTTCATCGAGAGGAACCACATGGAGAACGGCGACCACCGGTTCATGATGAACAACTACGAGTGGAACCAACCCGACAACTTGAAGAGATTTTCCATGTTCCTGTCTCCCAAAGCCAGTTTCAACCCACGATCATGGGAATTTTCCTCCTTCCAAGCGACTGAGGAAACACTTGGCAATGGCACTAAGCTGCCTTTTCAGTTCTCCTCGGTGGGACAGAATCCGCCCGGTGACTTCAGCAAACAGTCTGATGGGAGCCTCTTCACTAAGACCGCTTATCCCTCGATAGTGAGACATCAGTCTGCAAAGGTGACGCCACAGACGTGGAAATCCTCCAAGCAGTCTGTGTTGTCACATTACCGCCCCTTTTACGTCAACAAAGGCAATGGAGCCACCTCCAACGAGGCACCTTGA

>TRIM32

ATGGCAACCACGGCACTGAAAAACAGAACCAAAACAATTGGAGCTCTTTGGAATAAATGTGAGTACCGGGCTTGGCACAGGCTAAAGGCGGCTTCGAAAGCCATGGCTTCTGCCCCTCATCTCAACTCGGATGCGCTCCGCGAGGTCCTGGAGTGCCCCATCTGCATGGAGTCCTTCACCGAGGAGCACCTGAGGCCCAAGCTCTTACACTGCGGGCACACCATCTGCAAGCAGTGCCTGGAGAAGCTGCTGGCTAACAGCATCAATGGCATACGCTGCCCCTTCTGCAGCAAAATCACCCGCATCACCAACCTGGCCCAGCTGACCGACAACCTGACGGTGCTGAAGATCATAGACACCGCGGGGCTGGGGGAGGTGGTGGGGCTGCTCATGTGCAAGGTCTGCGGGAGGAGGCTGCCCAGGCACTTCTGCAAGAGCTGTGGCTTGGTTCTGTGTGAGCCCTGCAAGGAGGCATCGCACGTGCCCCAAGGGCACAGCGTCATCGCCATCAAAGAGGCCGCTGAGGAGCGTAGGAGGGAATTTGGGACAAGGCTTGCCAGGCTTCGGGAGCTCATGGATGATCTGCAGAAAAGGAAAGCATCTCTGGAGGGTGTTTCGAGAGACCTGCAGTCGAGATACAAGGCAGTTCTGCAGGATTACAGCAAAGAAGAGCGCAAGATCCAGGAAGAACTTGCCAGGTCACGCAAGTTCTTCACCACCTCTTTATCTGAAGTGGAGAAGGTAAATAATCAGGTAATGGAGGAACAAGCTTATCTGCTGAACTTAGCAGAAGTGCAGATTCTGTCTCGCTGTGATTATTTCCTCGCCAAAATAAAGCAGGGAGATATAGCTCTGCTGGAGGAGGCGGCAGATGAGGAGGAGCCCGAGCTGACGAACAGTCTCCCGAGGGAGCTGACCCTGCAGGAGGTTGAGCTCCTGAAGGTGAGCCACGTGGGGCCACTGCAGATCGGGCAGGTGGTGAAGAAACCCCGGACGGTGAACGTGGAGGAATCGCTGATGGAAACCGCATCCTCCTCCTCCTCCTCGGTGTCGTTCCGGGAGCCTGAGCTGCAGGAGGAGGCCAGCTGCACGCCGCATGCCTCCCCAGCCAAGCCGAGGATGCCCGAAGCGGCCGCAAGCATCCAGCAGTGTCACTTCATCAAGAGGATGGGTTCCAAGGGCAGCCTGCCGGGGATGTTCAATCTGCCCGTCAGCCTCCACGTCACTCAGCAAGGAGAGGTGCTCGTGGCAGACCGAGGCAACTACCGAATCCAGGTTTTCACCCGCAAGGGCTTCCTGAAGGAGATCCGCCGGAGCCCTAGCGGAATCGACAGCTTCGTGCTCAGCTTCCTCGGGGCAGACTTGCCCAATCTGACTCCCCTCTCGGTCACCATGAACTGCCACGGCCTGATAGGCGTGACCGACAGCTACGACAACTCGGTCAAGGTGTACACCATGGACGGCCACTGCGTGGCGTGCCACAGGAGCCAGCTGAGCAAGCCCTGGGGCATTGCGGCGCTGCCTTCCGGGCAGTTCGTGGTCACTGACGTGGAAGGGGGGAAGCTCTGGTGCTTCACCGTGGACCGTGGGGTGGGGGTGGTGAAGTACAGCTGCTTGTGCAGCGCGGTGCGCCCCAAATTTGTCACCTGCGATGCCGAGGGGACCATCTACTTCACCCAGGGGCTGGGGCTGAACCTGGAGAACCGCCAGTACGAGCACCACCTGGAAGGGGGCTTCTCCATCGGCTCCGTCGGCCCCGACGGGCAGCTGGGACGCCAGATCAGCCACTTCTTCTCCGAGAATGAAGACTTCAGGTGCATCGCTGGGATGTGCGTGGATGCCAGGGGAGACCTGATCGTTGCCGACAGCAGCCGGAAAGAAATCCTGCATTTTCCCAAAGGAGGAGGCTACAATATCTTGATCCGCGAAGGACTCACCTGTCCTGTTGGCATTGCCATTACCCCCAAAGGGCAGCTGCTGGTGCTGGACTGCTGGGATCATTGCATTAAGATCTACAGTTACCACCTGAGAAGATATTCCACCCCTTAA

>TRIM33

ATGGCGGAAAACAAAGGAGGCGGCGGCGGCGGCGACGGGGCTGCCGAGGCCGGGCCGGGCGGCGGCGGCGGCCCGGAGCCCGTGGCCGCGTCCCCCTCCGGCGCCGCCCCGCCCGCGCCCGCCGCCGCGCCCCCCGAGGAGCGGGACAGCCCGGGCGCGGCGGCGGCGGCGGCGGCGGAGCGCGCTCTCGGGGAGGCCGAGGCCGAGGCGGCGGCGGGGCCCGGTGCGGTCCCGGGGCCCGGCCCCAGCCCCGTGCCCCCGCTGACGCCGGCGGCGCCCGGGCCCTTCTCGCTGCTGGACACCTGCGCCGTGTGCGCGCAGAGCCTGCAGAGCCGGCGCGAGGCCGAGCCCAAGCTGCTGCCCTGCCTGCACTCCTTCTGCCGCCGCTGCCTGCCCGAGCCCGAGCGGCAGCTCAGCGTGCCCGCGCCCGGCGGCGCCAACGGCGACATCCAGCAAGTTGGTGTAATCAGATGTCCGATATGCCGCCAAGAATGCAGACAGATAGATCTGGTGGATAACTACTTTGTAAAAGACACATCCGAAACACCAAGCAGCTCTGATGAGAAATCAGAACAGGTGTGCACAAGCTGTGAAGATAATGCTAGTGCTGTAGGATTTTGTGTGGAATGTGGGGAATGGTTGTGCAAGACCTGCATAGAAGCTCATCAGCGAGTAAAATTTACTAAAGATCATATGATCAGAAAAAAAGAGGATGTATCTTCAGAGGCCGTGGGAGCATCTGGTCAACGTCCTGTTTTCTGTCCTGTCCACAAACAAGAGCAGTTAAAACTTTTCTGTGAAACATGTGACAGGCTGACATGCAGAGACTGTCAGTTACTGGAACACAAAGAACACAGGTATCAGTTTCTAGAAGAAGCTTTCCAGAATCAGAAGGGTGCAATCGAGAACCTGTTGGCCAAACTTCTTGAGAAGAAGAATTATGTAAATTTTGCAGCTGCCCAAGTTCAGAACAGGATAAAAGAAGTAAATGAAACTAACAAACGAGTAGAACAGGAAATCAAAGTGGCCATATTCACGCTCATCAATGAAATCAATAAAAAGGGAAAATCTCTCTTACAGCACCTTGAGAATGTAACAAAGGAGAGACAGATGAAGTTAATACAACAACAGAATGACATCACCGGTCTTTCGCGACAAGTGAAGCATGTGATGAACTTTACTAATTGGGCTATTGCAAGTGGCAGCAGTACTGCTTTACTGTACAGTAAACGACTGATAACGTTCCAGTTACGTCATATTTTAAAGGCACGTTGCGATCCTGTCCCAGCTGCCAATGGAGCAATACGGTTCCATTGTGACCCTACATTCTGGGCAAAGAATGTCGTCAATTTAGGTAACCTCGTTATTGAAAATAAACCAACTCCTAGTTACACTCCCAATGTAGTGGTTGGACAAGCTCCTCCAGGAACAAATCATGTCAACAAAACTCCAGGACAAATTAACCTAGCACAGCTTCGACTTCAGCATATGCAGCAACAGGTGTACGCACAAAAACATCAGCAACTGCAGCAGATGAGGATGGGACAGCCATCTGGGTCAGTTCCCAGACAGACAGGTCCTCAAATCTTACAGCAGCAGCCTCCCAGGTTGATCAGCATGCAGACCATGCAGAGGGGTAACATGAACTGTGGGGCTTTCCAAGCACATCAGATGAGAATGGCTCAGAATGCTGCTCGTATACCAGGAATACCACGCCACAATGGACCACAATACTCCATGATGCAACCTCACCTTCAAAGACAACATTCTAACCCTGGACATGCGGGGCCATTTCCAGTTGTTTCTGTGCACAACAACACTATTAATCCAACTAGTCCCACTACTGCAACAATGGCAACTGCAAACCGTGGTCCAACAAGTCCGTCTGTTACAGCAATTGAACTCATTCCTTCTGTAACAAATCCGGAGAATTTACCTTCCCTGCCAGATATCCCACCCATCCAGCTTGAAGATGCTGGTTCAAGTAGTTTGGATAACCTTTTAAGTAGATATATCACAGGCAGCCACCTACCCCCGCAACCTACAAGTACAATGAATCCCTCCCCAGGGCCTTCAGCTCTATCTCCAGGGTCATCAGGCTTATCCAACTCCCACACTCCTGTGAGGCCACCTAGTACCTCCAGCACAGGTAGCAGAGGAAGCTGTGGCTCCTCGGGCAGAACAGCTGAGAAAACTAGTGTTAACTTCAAGTCTGACCAAGTGAAAGTCAAGCAAGAACCAGGGACAGAGGAAGAGATATGCAGTTTCTCAGGAACAGTAAAACAGGAGAAAACGGAGGATGGCAGAAGGAGTGCTTGCATGCTTAGCAGTCCTGAGAGCAGCTTGACACCACCACTATCCACTAACTTGCATCTGGAGAGTGAATTGGAAGCATTAGGAAGTTTCGAAAACCATGTAAAAACTGAACCTGGAGATTTAAGTGAAAGTTGCAAGCAGTCTGGACATAGCCTTCTAAATGGAAAATCCCCAGTGCGGAGTCTAATGCATCGATCAGCTAGAATTGGAGGAGAAGGCAATAATAAAGATGATGATCCAAATGAAGACTGGTGTGCAGTATGCCAAAATGGAGGGGATCTATTATGTTGTGAAAAGTGCCCAAAAGTGTTTCATCTAACTTGTCATGTACCAACACTCCTCAGTTTTCCAAGTGGAGAGTGGATATGTACATTCTGCAGAGATCTGAGCAAACCAGAAGTAGAATATGACTGTGACAATTCGCAACACAGCAAGAAAGGGAAAACAGCACAAGGCCTGAGTCCTGTGGACCAAAGGAAATGTGAACGTCTCCTGCTTTACCTGTATTGTCATGAGCTGAGCATTGAATTTCAAGAGCCAGTCCCAGCCTCGATACCAAACTACTATAAAATTATAAAGAAACCAATGGATTTATCTACAGTTAAAAAGAAACTACAAAAGAAGCATTCCCAACATTACCAGACTCCTGAGGATTTTGTGGCAGATGTCCGGTTGATCTTCAAGAACTGTGAAAGGTTTAATGAAGCTGATTCAGAAGTAGCACAGGCAGGGAAGGCAGTTGCATTATACTTTGAAGATAAACTTACAGAGATCTACCCAGACAGGACCTTCCAGCCTTTGCCTGAATTTGAGCAGGAAGAGGATGATGGTGAAATAACTGAGGACTCCGATGAAGATTTTATACAACCACGTAGAAAACGCCTAAAATCAGATGAGAGACCAGTGCATATAAAGTAA

>TRIM35

ATGCTGCCGGCCCCCGCACGGCCCCCGCATCGCTCCGCACGCCGGAGGGGACGCTGCAAGCGCTGCCGCTCGCCCTCAGTAAGCGCCATGGAGAAGGCAACCAGCCCCCCGCCCGGCAGCTCGGTGATTTTAGCTTCGTCCTCCGCGTCCACCTTGAAGGAGGAGCTGCTGTGCCCCATCTGCTACGAACCCTTCCGTGAAGCCGTGACCCTCTGCTGCGGCCACAACTTCTGCAAGGGCTGCGTGAGCCGCTCCTGGGAGCACCAGTACCACCTCTGCCCCGTCTGCAAGGAACCCGCTTCCCCCGACGACCTCCACGTCAACCACACGCTCAGCAACCTGGTGGAGATGATCCTAAAAGAGGAAGGGCAGCGGCGGGGCCGCCCGGCCGCCCTCTGCACCGTGCACCACGAGGAAGCCAAACTCTTCTGCCTGGACGACAAGGAGCTGGCGTGTTTCTCCTGCCAGAGCTCCAAGCAGCACGAGGGGCACAAGATGAGGCCGGTGCAGGAGACGGCTGCGGATTTCAGGGCTCGCGAGGGTCCTGAAGGCGTCTCCCCCCCGCAGGCCAAGCTGAAGAACATGGAGACCTCCCTGCGGGATAAAGTGAAGGATTTCGGGACCGTGCACCGCAGCTACGAGTCCATCTCCAAACACAACCAGGTGGAAGCCGTGCGCCTGGAGGAGCAGATCAAGAAGGAGTTTGAGAAGCTGCACGAGTTCCTGCGGGACGAGGAGAAGGCGCTGCTGGCCCAGCTGCAGGAGGAGACGCGCCGCAAGCAGGACCTCGTCGAGGGCAAAATCAAGCAGCTGTCGGACGAGAGCCGGGCTCTGCTCAACGAAGCCTGCCAGCTCCAGGACGACCTCAAGGAGGACGACTACACCTTCCTCATGACCCACAAGAACCGCAAGCGCAGGATCGCCTGCACGGTGGAGGAGCCGGAGGCCGTGGCCTCGGGGATGCTGATCGACACCGCCAAGTACCTGGGCTCGCTGCAGTACAACGTGTGGAAGAAGATGCTGGACAACATCACCGTGGTCCCCTTCAGCTTCGACCCCAACTCCGCCGCGGGCTGGCTCTCGGTGTCCGAGGACCTGAGCAGCGTCACCAACGGGGGCTACAAGCTGCTGACGGAGAACCCCGAGCGTTTCACCTCCGCCCCCTGCATCCTGGGCTCCCGCGGCTTCTCCGCCGGCTTCCACAGCTGGGAGGTGGATCTGGGAGGCATCACCAACTGGCGGGTGGGGGTGGCTCGACCCCGAGGGAGAACCCCTTGGAATTTTCACCACGACGCCCGCTCCGGTTTTTGGTACCTCTACCGCCTGCCCACGGACGGCGAGACGTGCCGGGCTTCCAACGCCGCTCGCTCGGAGGCCACGCTGGGCGAGTTGGGGAGGCTCCGGGTGGAGCTGGACTGCGACGAAGGGGAGCTCTCCTTCTACGACGCCGACCGCAGGAGCCACATCTACACCTTCCACGAGAAATTCGGGGGCACCGTCTTCCCCTATTTCTACATGGGGGCCACCCCGGTGGGCGCGCTGCCCAGGGCGCTGCGCATTTGCCCCGTCCGGGTGCGCATCCACGAGGACACCCCCGTGTAG

>TRIM36

ATGGAGGGCGACGGGCTGGAGCCGGAGCCGGAGCCGGAGGTTGCCACAAAGAGTATTGAAAGAGAGCTCATCTGCCCAGCGTGCAAGGAGCTGTTCACCCACCCGCTGATCCTGCCGTGTCAGCACAACGTCTGTCACAAATGCGTGAAGGAAATACTCTTCACACTTGAGGACTCTTTCGCTGATGGGGGGTCTGAATCCTCTAATCAGAGCAGCCCTCGAATCCGGATCTCTTCTCCCAGCATGGATAGGATTGACAGGATTAGTAGATCAGGCAGAAAGCGCAATTCACTGACTCCTAGAACAACTCTGTTCCCTTGTCCTGGCTGTCAGCACGATATTGACCTTGGAGAACGTGGCATCAATGGCTTGTTTCGCAATTTAACGTTGGAAACCATTGTGGAAAGGCACAGGCAGGCAGCCAGGGCAGCCATAGCTATCATGTGTGACTTCTGCAAACCTCCACCTCAAGAGTCCACCAAGAGCTGCATGGACTGCAGCGCAAGCTATTGCAACGAGTGTTTCAAAGTTCACCACCCCTGGGGAACCCTCAAAGCCCAGCACGAGTATGTCGGACCAACCACCAATTTCAGACCCAAGATTTTGATGTGCCCAGAACATGAAATGGAAAGAGTAAACATGTACTGTGAAATCTGCAGAAGGCCTGTCTGTCATCTTTGTAAACTGGGTGGATCTCACGCAAACCACAGGGTAACAACGATGAGCACTGCCTATAAAACACTGAAGGAAAAGCTTTCAAAAGATATAGAGTACCTCATCAGTAAGGAGAGCCAGGTGAAAGCTCACATCTCACAGCTGGATCTGCTGCTGAAAGAAACAGAGTGCAACGGGGAAAGAGCTAAAGAAGAGGCATCTCAGAGTTTTGAGAAGTTAATTCATGTTCTGGAGGAGAAGAAATCTGCAGCTCTTCGGGCAATTGAAGCATCTAAGAATTTAAGACTGGACAAATTGAGAACCCAGGCAGAAGAATATCAAGGTCTCCTGGAAAATAATGGCCTCGTTGGATATGCTCAAGAAGTACTTAAAGAAACAGATCAGTCCTGTTTTGTTCAAACAGCAAAACAACTTCATGTCAGAATCCAAAAAGCTACTGAATCTCTGAAGAGCTTCAGACCAGCAGCTGAATCTTCTTTTGAAGACTTTGTGGTGGACACTGCCAAGCAAGAACAGATCCTTAGCGACTTGTCCTTCTATTCCAATGGTCTGGAAATACCAGAAATCAATGAGGAGCAGAGCAGAATATACAACAAAGCTGTGATTAGTTGGGAGTCCCCTGGGAAGACAGACGCAGCTGATATCTATGTTCTCGAGTATCGTAAGTTTAATAGAGAAGAAGAGAATGTGATGTGGCAGGAGATCGAAGTTTGCAGCAAGAGCAAAGTCATATCTGATCTTGATGACGACAGCAGCTATGCTTTTAGAGTTCGAGGATATAAAGGGTCCATCTGTAGCCCTTGGAGCAAAGAAGTTATTTTGCATACACCTCCCGCTCCAGTTTTTAGCTTTCTTTTTGATGACAAGTGCGGGTACAACAGTGAACGTCTACTGCTCAACCCAAGAAGAACTGCTGTGGAAAGTAGAGCTGGATTTCCTCTACTGCTAGGAGCTGAACGCATACAGGTTGGATGCTACACAACCCTGGATTACATCATTGGTGACACTGGGATTGCCAAAGGGAAGCACTTCTGGGCCTTTCGTGTGGAAGCCTATTCCTACCTGGTGAAAGTGGGAGTCGTTTCTAGCGCCAAGATACAGAAATTGTTCCATAATACCCATGATGTGACCAGCCCAAGGTATGAACAAGACAGTGGCCATGACAGCGGGAGTGAGGATGCCTGCTTTGACTCATCACAGCCTTTCACGCTGGTCACTTTGGGCATGAAGAAGTTCTTCATCCCCAAGGCCGCTGCTGACCCCAAGGACGCAGCGAGCAGAGTCCTCCCCCTGCCCTCGTGCCTGGGGATCTGCCTTGACTGTGACAGCGGCAAGGTGGGTTTCTACGACGCTGGCCGCATGAAATGCCTCTACGAGTGCGAGGTGGACTGCTCGGGCATAATGTACCCAGCGTTTGCCTTAATGGGTAGCGCAGCAATTCACCTTGAGGAAGCCATCGCAACAAAGTATTTGGAGTACCAGGATGACCTGTAG

>TRIM37

ATGGACGAGCAGAGCGTGGAGAGTATTGCTGAAGTATTCCGATGTTTTATTTGTATGGAGAAATTGCGTGATGCACGACTGTGTCCTCATTGCTCCAAGCTGTGCTGTTTCAGTTGTATTCGGCGTTGGCTGACTGAACAAAGAGCTCAGTGCCCTCATTGTAGAGCCCCGCTGCAGCTACGAGAGCTTGTAAACTGTCGTTGGGCAGAAGAGGTCACACAACAGCTTGACACACTTCAACTGTGTAATCTGACAAAGCATGAGGAAAATGAAAAAGACAAGTGTGAAAATCATCATGAAAAGCTTAGTGTATTCTGCTGGACCTGTAAGAAGTGTATCTGCCATCAATGTGCGCTTTGGGGAGGAATGCATGGAGGACATACTTTTAAGCCACTGGCGGAAATATACGAACAACATGTCACAAAAGTAAATGAAGAAGTGGCAAAGCTTAGGAGAAGACTCATGGAACTGATCAGTTTAGTGCAAGAAGTGGAGAGGAATGTGGAGGCTGTGCGCAGTGCGAAGGATGAACGAGTTCGGGAAATAAGAAATGCGGTAGAGATGATGATTGCCCGGTTAGACACTCAGCTGAAGAATAAGCTTATAACACTAATGGGTCAAAAGACATCTCTTACCCAAGAAACTGAACTTCTGGAATCCCTGCTTCAAGAAGTAGAACATCAGTTACGATCATGCAGCAAGAGTGAGTTGATATCCAAAAGTTCAGAGATCCTGATGATGTTCCAGCAGGTTCATCGGAAACCTATGGCCTCGTTTGTCACTACTCCTGTTCCTCCAGACTTCACAAGTGAATTGGTCCCAGCCTATGACTCAACTACCTTTGTACTGGAAAACTTCAGTACGTTGCGTCAGCGAGCAGATCCTGTTTACAGTCCACCTCTTCAAGTGTCAGGACTTTGTTGGAGGTTAAAAGTTTACCCAGATGGAAATGGAGTGGTAAGGGGCTACTACCTGTCTGTGTTCTTGGAGCTGTCAGCTGGACTGCCAGAGACTTCCAAGTACGAGTACCGTGTAGAAATGGTTCACCAGTCCACCAATGATCCCACTAAAAACATAATTAGAGAGTTTGCTTCTGATTTTGAAGTTGGAGAATGCTGGGGTTACAACAGATTCTTTCGTCTAGACTTGCTAGCCAATGAAGGCTATCTGAACAGACAGAATGACACCGTGATCCTAAGGTTTCAGGTGCGCTCACCAACCTTTTTCCAAAAGTGTCGGGATCAACACTGGTATATTGCTCAGTTGGAAGCAGCTCAGACAAGTTATATCCAACAAATAAATAATCTCAAAGAAAGGCTTGCGATTGAACTTTCCCGGACTCAGAAATCACGAGGCATTTCACCTCCAGACACCCATCTTAGTCCCCAGAACGATGATAGTCCAGAAACAAGATCAAAGAAATCTGGACAAAGCACTGAAGTACTACTTGAGAATGTTGCTGCTCCAGGATTAGCGCGAGACAGCAAGGAGGAGGATGAAGAGAAGATCCAGCATGAAGATTTTAATCATGAACTCTCTGATGGTGATTTGGATGTAGATCTTGCTGGAGAAGATGAGGTGAACCACCTCGACGGTAGCAGCTCTTCAGCTAGTTCGACTGCAACTAGTAACACTGAAGAAAATGATATTGATGAAGAAACTATGTCTGGAGAAAATGACGTGGAATACAGTAATAATATGGAGTTGGAAGAAGGAGATCTCATGGAGGATGCAGCAGCTGCTGCTACTCCTGGAGCGTCAGGTACCAGCCATGGCTACACCAGTGCAAGTGGAAGACCTTCTAGGCGGGGAGGAGGTGTCCTAGGCTCGACAGCCAGTAGCAGTTTACTAGATATTGATCCCTTGATTTTAATCCACTTGTTGGATCTCAAAGACAGAAATGGTATGGAAAACCTCTGGGGCCTTCAGCCACGTCCACCTGCCTCTCTTCTACAGAACAGAGCATCATCGTATTCTCTAAAAGATCGAGATCAGCGGAGACACCAGGCGATGTGGCGTGTGCCGCCTGACCTGAAGATGCTGAAGAGACTTAAAACTCAGATGGCTGAAGTACGAAGCAAGATGTCTGATGTGAAAAACCAACTATCAGAAGTAAGAAGCAGCAACGCTGGCTCATGCGACGGGCAGCCCAACTTCTTCTCCATTGAGCAAGGTGCTTTAGCTGCCTGCGGAACGGACAGCTGCAGCAAGCTGCAAGAAATAGGAATGGAACTACTGACCAAGTCTTCAGTTACCAGTTGTTACATAAGGAATTCTGCGAGTAAGAAAAGTAACTCCCCCAAGCCTATTCGCTCTGGAGCAGCAGGGAGTCTGTCTTTACGAAGAGCTATGGACTGCGGGGAAAGCAACCTTCGGCTAAAGGGAGACAGTCAAACTTCTGAGGGTGGCCTAGGGAGTTCGAAGTCAAGCAGTCGGCACCACTGTCCCAGGCCCCTGGCCAGCAGTAATGCTGCTGAGGCTCTGCCTAAGGCAGAGGAAAGACCTTGTGAAGTTTCAGATTCTGATGTGGGAGTTTCTGGCTTAAATGGCTTAACTGCTGTAGAGAAGACCAGAAAAGCTGGTGCTCTCGTTGCTCTAGGGTCAAACTCTAAAGGATATCGCACTGAGGGAACACAGTCAGGTAGTCTGGAAAACAACGCTGAAACTGGTGAACTGCAGGGCGTGCTTTCCGAAGGCGCTTCAGCGGGGCCAGAAGAAGGTGGGGCATGTCAGAAGCATGTCCTTCACCTCCTGCCGGGAATGAGCAGTGATAGTGACATTGAATGTGACACAGAAAACGAAGAGCAGGAGGATGCCACCTCTACGTCGGAGGGGTTTAACCATGCCTTTTCTGTGCAGTCCTCCAGTGAAGCCTCAGAGCGGTGCTCAGTGTTCCCAGAGGGTGATCAAGTTGGTCCTGATGATCTCAGCTTTGTTAATGGAGAAGACAACACCAGGTTGTATTTGCTGAAGCCTTGA

>TRIM202

ATGAATCTCTCAGCTCCCTTTCACCTCCCTCGGGGCCCCAGGCACCTGCAGGAAGGGGAGCAGCCAACGGCGAGACCAACCGGGGCGGAAAACGAAAGCTGCCCCGTCATCTTCGCCACCGAGAAGACCATGGCTGAAGACATCCCCGCCGAAAGCTTCCAGGATGAAGCCTCCTGCTCCATCTGCCTGGGCTTCTTCCAAGACCCCGTCTCCATCCACTGCGGCCACAACTTCTGCCGGGCGTGCATCACCCGCTGCTGGGAGGAAGAGGAGGCGAATTTCACCTGCCCGCGGTGCAAAGAGACGGCCCCGGAGAGAAACCTGAGACCCAACCGGGAGCTGGCGAAAATCATCGAGATAGCCAAGAGGCTGAGCCTGCAGGCAGCCAAAGGAGGGGCAGGGGGGGAGAGGCTGTGCGAGAAGCACCAGGAGGCTCTGAAACTCTTCTGCGAGGAGGACCAGACCCCCATCTGCCTGGTCTGCAGGGAGTCCCAGGCGCATCGCGCCCACCCCGTGGTCCCCATCGAGGAGGCTGCAGAGGAGCACAAGGAGAAATTCCAGGCTCACGTGCAGATCCTGAAGGACAGGAGAGAAAAACTGCTGGGGCTGAAAATGGCTGAGGAAGGGAGAAGCCTGAATTTCCTCGAAAGGGTAGAAGAGGAGAGGCAGAAGGTCGTGGCCGAAATCAAGGAGCTGCACCAGTTTGTGGAGCGACAGGAGCAGCTCCTCCTGGGCCAGCTAGCCAAGCTGGACCAGGAGATCGTGAGGAGGCAGGAGGAGAACATCGCCAAGATCTTGGAGGAGATCTCCTCCGTGGGTGAGCAGATCCGTGAGCTGGAAGAGAAGTGTCAGCAGCCAGCATATGAACTCCTACAGGACAGCAGGAACATCCTGAGCAGGCTTGAGAAAGAGTGCGCCCAGAAGCCACTGGAGGCATCGCCTGAGCTGGCCGAGACGCCCACTAGTCTTCCCCAGAAGAACATTGCCCTCAAGGAGATGCTGATGAAATTTCAAGTAAGCCTGACGCTGGATCCGGAGACGGCGCATCCCCGGCTGGTCCTGTCCGAGGATCGCAAGCGCGTGAGATGGGAAGACACCCGCCAGCCCGTGCCCGACAACCCCAAACGTTTCGATTCGTCTCGCTGCGTGCTGGGCTGCGAGGGCTTCAGCACGGGGAGGCACTACTGGGAGGTGGAGGTGGGCGACGGGGAAGCCTGGGCCGTCGGGGTGGCCAAGGAGTCGGTGAGGAGGAAGGGACGGATCAGCGTCAACCCCGAGGTGGGCATCTGGGCGGTGGGGCAGTGCGGGAGCCAGTACCAGGCTCTCACCTCTCCTACCAGCCCCATCTCCCTGNCTCGCTCCCCCAGGGGGATTGGGATTTACCTGGACTACGAGGCAGGGCGCGTGGCGTTTTTCGACTCCCACAACCTGGCGCCCATCTTCACCTACCCGCCGACGTCCTTTGCAGGGGAGCAAATCCTCCCCCTGCTCTGCTTGGGGAGGGGGTGCCAGTTCACTCTGTCCCCCTGA

>TRIM41

ATGGCGGTGGCGGGCGACGGGGGGCCCGGGGGGCGCCTGAACCCGGTGGAGACGCTGCAGGAGGAGGCGATCTGCGCCATCTGCTTGGACTACTTCCTGGAGCCCGTGTCCATCGGCTGCGGCCACAACTTCTGCCGCGTGTGCATCTCGCAGCTGTGGGGAGGCGAGGCGGAGTACGAGGCGGCCCCCGGAGCGGCGGCGGCTGGAGGAGGAGGAGGAGGAGGCGGAGGGGCCGTGGGGTTGGGGGACGACGTGTTGGAGGAAGAGCTGGAGGATGAGGAGGATGAGCTGGATGAGGATGAGCTGGAGGTGGAGCAGGAGGAAGAGGAGGAGGAGGATGGCGGCGGCGGAGGAGCCGAGGAGGAGGAGGACATGTGGAGCGAGGAGGAAGACGACGGCGAGCTGTGGGAAGAGCCGGTGGAGGAGGACATTTGGGACGCTGGGGTCGGGGGAGAGCTTTATTTCGGGGACGAGGACTATGACGAGGACGTGATGGAGGAAGACGTGGAGGAAGAGGAGGAGGTGGAGGAGGAGGAAGAAGAGGTGCAGACCCCTCCGCCCCCGGTTCTGGCAGCCCGGCCCCGGCGCCAGCAGACCTTTACCTGCCCCCAGTGCCGCAAAACCTTTTTCGAGAGGAATTTTCGGCCCAACCTCCAGCTGGCCAACATGGTGCAAATCATCCGGCAGCTTCACCCCTACCCCCCGCGCCTCGCGCCTCCCGCCGCCGGCCCCTCGGCTTCGGGGGCTGCGGCCGGAGGGCCTGGGGGGGTCCTGGTGGCCGGGGGGGGTCAGGGACCTCCGAACCTTTGCGAGAAGCACCAGGAACCCCTGAAACTCTTCTGCGAGGTGGACGAGCAGGCCATCTGCGTGGTGTGCAGGGAGTCGCGGAGCCACAAGCACCACAGCGTCGTCCCCCTGGAGGAGGTCGTGCAGGATTATAAGAACAAACTCCAGAGCCACCTGGAGCCGCTGAAGAAGAAGCTGGACGCGGTCCTGAAGCAGAAATCGAACGAGGAGGAGAAGATCACGGAGCTGAGGGACAAGATGAAGCTGGAGATCAAGGAATTGGAGTCGGATTTCGAGCTGCTGCACCAATTCCTCATCGGGGAGCAGGTCCTGCTCCTGCACCAGCTGGAGGAGCGCTACGAGAGCCTGCTGGTGCGCCAGAGCAGCAACATCAGCCAGCTGGAGGAGCAGAGCGCTGCCCTCAGCCGCCTCATCGCCGAAGCGGAGGACAAAAGCAAACAGGACGGGCTGCAGCTGCTCAAGGACATCAAAGGCACTTTTATCAGATGCGAGAATATCAAATTCCAGGAGCCCGAGATGGTGCCGGTGGACATGGGGAAGAAATACCGCAACTACTTCCTGCAGGACGTGGTGATGAGGAAGATGGAGAAAGTCTTCAGTAAAGTCCCTCAAGCTGACATCACCCTGGACCCCGACACCGCTCACCCTCGCCTGAGCCTTTCCCTGGACCGCCGCAGCGTCAAGCTGGGCGAACGCTGCAAGGACCTGCCCGACAACCCCAAGCGTTTCGATTCCGATTACTGCGTCCTGGGCTCGCAGGGTTTCACCGCCGGCCGCCACTACTGGGAGGTGGAAGTCGGGGGCCGTCGGGGCTGGGCGGTGGGGGCGGCCCGCGAAACGGCCCGTCGCAAAGAGAAAACCATGGGGCCCCACCAGGAACGGGAAATCTGGTGCGTGGGCACCAACGGGAAGAAATACCAAGCCTTGACGGCCACCGAGCAGACGGCTCTGTCGCCCAACGAGCGGCCCCGGCGCTTCGGCGTCTACCTGGACTACGAACGGGGCCAGATTTGCTTCTACAACGCCGAGAGCATGACCCACATCCACACCTTCAACGCCTCCTTCCACGAGCGCATCTTCCCCTTCTTCCGCATCCTGGCCAAGGGCACCCGCATCAAAATCTGTGCCTGA

>TRIM212

ATGGCCCTCTCCGGAGCCCTGGAGCGGCTGCAGGAGGAGGCCATCTGCTCTATCTGCCTGGAGTACATGACTGAGCCCGTCAGTGTCGACTGCGGGCACAACTTCTGCCACGGCTGCATCACCAAGCACTGCCAGGAGAAGTGTCTCTGGAATGACGCGCCCTTCTCCTGCCCGCAGTGCCGGGCCCCCTGCCGCCGCAGCAGCCTGCGCCCCAACAGGCAGCTGGCCAACATCGTGGAGAGCATCCGGCAGCTGAGGCTGGGGGGCGGCGTGGCGCCAGGGACCCTACTCTGTACCCAGCATGACGAGCGCCTCAAGCTCTTCTGTGAGGTGGATGAAGAGGCCATCTGCGTGGTGTGCCGGGAGTCCCAGCACCACCGCTCCCACACCGTCTACCCCATCGAGGAGGCTGCACAGGTGTACAAGGTGAAACTCCAGAAAGTGTTGGAACATCTTTCCAAGGAAGTAGAGGATATGAAGAAATGTGAGTCAGTGGCAAAGATGAAAATCCAGGAGTGCAAGGAGACAGTGAAGAAAAAGCGGGAGAGGATTGTGAGCGAGTTTGGGAAGCTGCATCGGCTGCTGGCTGCTGAGGAGAAGCTGCTTCTTCAGAAGCTGGAGGAGGAGGAGAAGCAGATTCTAGTGATGATCTCTGAAAGCATGTTTAGGCTGGTGGAGCAGAAGTCCTTGCTGGATGAGCTGATCCTGGAGATAAAGGAGAAAATGCACCTGCCGGACGAGGGGCTTCTCAAGGACATGAAATGCATCCTGAGCAGGTGTGAAGCGGTGAAGTTCCAGACCCCTAAAGCTGTGTCTGTGACCCTGAAGGAGGACTACAGCATTCCGGAGCGCTGTCTGGGCATGAGGGAAATGCTGAAGAAGTTCAAAGTGGACGTGACTCTAGACCCTGAGACAGCGCACCCTGATCTTATCCTATCTGAGGACCACAAGAGCGTGCGGCGTGGGGGCAGGAAACTGCTCCTGTCCTTCTTTGACAACACCAGGAGGTTTAACTCTGCTCCGGTGGTGCTGGGAGTGCAGTTCTTCTTCTCAGGTCGCCACTACTGGGAGGTGCATGTGGGAGACAAGCCGGAGTGGGGCTTAGGGCTGTGCAAGGAGGCTGCCAGCCGGAAAGGCAACATCCTCTTCTCTCCAAACAATGGCTACTGGGTGCTACGGCTGCAAAATGGCGGCAACTATGAGGCCCTGACCTGTCCCGTCTCCCATCTGACCCTGAGCATCAGACCCCGGTGCATCGGCATCTTCCTGGACTACGAGGCTGGAGAAATCTCCTTCTACAACGTGACCGACCGCTCCCACCTTTACACTTTCACTGATAAGTTCTCAGGGAAGCTCCGGCCTCTTTTCTACCTGGGTTCCTTCTTGGGGGGCAAAAATGCAGAGCCCTTGGTGATCTCCTGGATGAGGGACATGCAGGGGACTGGCTGCATCGTCCTGTAA

>TRIM42

ATGGATGAAAATGGATGTTCCTTTTCAATTTGCCCATGCTTCTCAAATTGCTTTTACCTGACTTGCCACAGCAGAAAGAAAGAATGCTGCTTATGTTGGCGCTTCCTATTTACCAGCGAGCAGAACTGCACCTGTTTCCCATGTCCATATGAAGAAGACAAACCTTATCAGTGCTGCCACTGCTCATCTGCAGAGCATGCAAATTGCTGGTGGTGCTGTTGCTCTTGTTCAAATGACCCAGACTGCAAATGCTGCTGCTGCAGCGGAGAGAATTCAGCATGTCAGTACTATGCAAGCAAATGCTGCAGAAATCCCATCAGTCGAGAACAGTCAAGATTATCAGCAACTTTTCAGTCAAAAGATGTCATGTCAAGATTCAGAACTAGGAATAATGCATTCGTCAATACACCAGAAAGGAATGCCTCTAACAATGCCTTCCGTGACCAGCTTGCCTGTCCGCTGTGCAAGCAGTTGTTTCTCCAGCCATTCATGCTGCCATGCAATCACTGCATTTGTGAGAAGTGCATAACTAAAAGCAAGACCAAAGCTGAAGCAACAGACAACTTTTATATCATTGTATGCCCAGTATGTAGTAAAGCACATTGTCTCCCCTACACAAATAAAATTCAGCTGAGGAAGAATTACCTCAGAGCAAAACTAGCCAAGAAATACATGCGCAGACACGGCATTCTGAGGTGGAGATTTGACCACAGTGAAAGACCAGTCCACTGTGATATCTGCAGGGAGGGAAGAAGAATGGCAACCAAAAGATGTAAAACATGCGGAATTAACATGTGCAATGAATGTCTGCATTTATACCATAGTGAGAATGATGCTCAAGACCACATCTTCACCAAAGCAGACCAAGCAGATAATGAACAGTGGCCCTGCCTGCTACACTGCAACTCACACCTCTCTGAATACTGCCTGGATGACCACAAACTGATCTGCAGGTTCTGCAAGAACTCACTGCACAACAATCACGAGACTATTCCCTTGGCAGCTGCATGTTCAAAAGAGGCCACTTCCCTCTCCAGCACAATTGTAAAATTTAAACAAGCACGCCAAGGGGTTGATAATGATCTAATGGAAGTTATCCTCTTAAAAAATAATTTCCAGACCTACAAGGACACCAAAAGGAAGGAGATCAGAAATGGATTCTTAAAATTACGTATGGTTCTTCATGAACAAGAAAAAGAGATGATGGAGTTGCTTGAGAACATTGAACTTAAAAAACAGAAGGAAATTTCAGAATATGTAAACTACACATCCAGTCAGCTGTCATATATGGATGGCCTTATTCAGTACGCTGAAGAGGCTCTTAAAGAGGAAAGTCAAATTGTGTTTCTACAATCTGCACACTGCTTGGTGAAAGAAATAGAAGACGCAATTCCTTCCATTTTCCATCCTAGTCCACTTCTCAGGGAAGATCCTTTAAGAAAACTTCAACTCAACTTTGATGAACTTTTCGCTACTTTACAGGGACTTGTCCCATCCCTTTGTGAAATAAACCAATCAGACGGTAAGGCAGAAAAGAATCCCTATCTTTTTAACCCAGAAATAATGCTTCCAATGCATGTTTCAAGCACTCACGAAGACAAACAAGCAACATTGTTCCGAAGCACCTCTTTAAATTCTGTGTTCGAATCAGGCACGATGATTAAGAACACTCCTGGAAGACAACCAAGCTCTATGCCTCCCCATCATTCTACACAGAGTAATAACATGTGTGCATTTTGGGATGCAGCGTGTGAAACTCCAACAAAAGAAAGAAAATATCAGTTTGTTAACCTTCCAAGTCCAGAACCTATAGAAAAATTATTAGTCCCAGTGCCAGGACATGTTGTTATATACCAGACTGTTGTCTACCCAAGGTCTGCCAAAATTTACTGGACTTGTCCAATAGAAGATGTAGATTTCTTTGAGGTAGAGTTTTATGAAGTTGTTGGCATTGGTTCTGATAACATTGTCCAGACACAACTAGATGGACAGCTAAGTAAAATACAACAGCAGAACCTTGAGATACGTAATCTCGATCCAAATACAGAATACCTTTTTAAAGTCCGTGCTGTCAATAGAAGTGGTGAAGGAGAATGGAGTGAAATCTGTAAGATAACCACTTCAGATGAACACAGAATAATTCAAGACAGATGGGGGATTCAGAAGAGTATGCAGGGTGCTCTGCATACACTGAAATGA

>TRIM45

ATGGCGGCCGCCTGGCGCTGCCCGGCCTGTGCCGAGCCCTGCGCGGCGCCTCGGCTGCTGCCGTGCCTGCACTCGCTGTGTGCGCCCTGCCTGAGGCGGCTCGGCAGGCTGGGGGAGCCGGCGCGGGCGGGCATCGCCACAGCCACCACAGCCACCACAGCCGCCGCCATCAGCGTGCTGTGCCCGCTGTGCGACGCCGAGGTGGTGCTGCCGCCGGGAGGGGTCGGGCAGCTCCCCCCCGACTACGTGGCGCTGGGCCGAGGCCGGCCGGGCTGCGACCTGTGCGCCCAAGGGGCGGCCGTGGGGCGCTGCCAGACGTGCGGGGCCGCCCTCTGCCCCTTCTGCCGCCAGGCACACAGGAGACAGAAGAAGACTGCCTCTCATGCTGTGACAGAGCTGGAGAGCAGCAAGGACTGTGGCCAGGCTGGGAGGCCTCTTTTCTGCCCGTCCCATCCATCGGAGGAGCTCCGGCTGTTCTGCGAGCAGTGTGACCAGCCCGTGTGCCGGGACTGTGTCGTGGACAGGCACCGGCAGCACCCCTATGACTTGGCCAGCAACGTTGTCCACAGGCACGGGGACTCCCTGCGGGTGCTGCTGGAAAGCACCCAGCAGCGCGTGAGCACCCTGGAGGACGCGCTGAGCCAGATCGATGGCATGGGCAGTGCCGTCCGCGTTCGTGCAGAGGCCGTGGCCGCAGAGATCTGTCTGTTTGCCAGCGGGTACGTGAAAGCCATCGAGGAGCACCGGGAGCGGCTGCTGAAGCAGCTGGAGGACTTGAAGGTGCAGAAGGAAAACCTGCTGCACTTGCAGAAGGCCCAGCTGCAGCAGCTGCTGCTGGACATGAGGACAGGCATGGAGTTCACGGAGCGCTTGCTGAGCAGCGGCTCGGACCTGGAGATCCTCATCACCAAAGGGGTGGTGGCGAGCCGGCTGGCGAAGCTGAACAGCGTCGCTTACAGCACCCGCCCCAGCGTGGACGACAGCATCCAGTTCTCTCCCCAGGAGAGGGCAGGGCAGTGTTGCGGCTATGAGGTTTTTGGGGCCATTCTCAATAAAGTGGTTGATCCAGCCAAGTGTACCCTGCACGGGGAAGATCTCTGCATCGCCCGTCAGAACGAGCTGACTGGCTTTACCCTGCTCTGCAATGACACCACGGGAGAGCAGATGCGGAGGGGAGGAGAGGCCGTCAGGGTCACCATCACCCACAAGGACAAGAAGGATTGTGCAGTCAAACCAACGGTGTGTGATAACGGTGATGGGACCTACCATGTTTCCTACAGCCCTGAGGAGCCAGGCTTATATGCTGTCTGCGTCTGTGTGAAGGGGCAGCATGTACAGGGCTCGCCATTCACCCTGACGGTGAAAAGCAAGTTCCGCAAGCACCAAGGAGTGTTCCACTGCTGCACGTTTTGCTCGAGTGGAGGGCAGAAAGCCGCTCGCTGCGCCTGTGGGGGGACCATGCCAGGTGGCTACCAAGGCTGTGGCCATGGGCACAAAGGCCACCCTGGCTGTCCCCACTGGTCATGCTGTGGACAAGTCACAAAGAGCTCGGAGTGTTTGGGGCCACCCAATGACAGCTCACAGAGGAGCTTGCTCAGGACGGTGGCACTCTGA

>TRIM46

ATGGCCGAGGGCGAGGACCTGCAGACCTTCACCTCCATCATGGACGCGCTCGTCCGCATCAGCACCAGCATGAAGAACATGGAGCGGGAGCTGGTGTGCCCGGTGTGCAAGGAGATGTACAAGCAGCCGCTGGCCCTGCCCTGCATGCACAACGTCTGCCACGTCTGCGCCAGCGAGGTGCTGCTGCAGCACGGTTACCTCTGCTGCGACCCCACCTCCGAGCCCTCCTCGCCCGCCGCCACCCCGGCCACCCGCAGCCCGCGCCTGGGCCGCAGGGGGGTCCCCAAACCCGACCGCCTGGACCGGCTCCTGAAGTCAGGGTTCGGCACCTACCCGGGGCGGAAGCGGGGCACCGTGCACCCCCAGACCATCAGCTTCCCCTGCCCGGCCTGCCAGCGGGACATTGACCTGGGCGAGCGGGGCCTCGGCAGCCTCTTCCGCAACCTGACCCTGGAGCGCGTGGTGGAGCGCTACCGCCAGACCATCAACATCAGCGCCGCCATCATGTGCCAGTTCTGCAAGCCCCCGCAGCTGGAGGCCACCAAGGGCTGCACGGAGTGCAAGTCCAGCTTCTGCAACGAGTGCTTCAAGCTCTACCACCCCTGGGGCACCCAGAAGGCTCAGCACGAACCCACGCCGCCCACGCTCACCTTCCGCCCAAAGGGTCTGATGTGCCCAGAGCACAAGGAGGAGGTGACTCACTACTGCAAGACCTGCCAGCGGCTGGTGTGCCAGCTCTGCCGCGTGCGACGCACTCACACCAGCCACAAGATCACGCCGGTGCTCAGCGCCTACCAGGCTCTCAGGGAGAAGCTGACAAAGAGCCTTGCCTACATCTTGAGCAGCCAGGACACGGTGCAGACCCAGATTGCTGAGCTGGAGGAGACGGTGAAGCACACGGAGGTGAACGGCTCGCAGGCCAAGGAGGAGGTGTCCCAGCTGATCCAGGGGCTGTGCTCCATGCTGGAGGAGAAGCGGGCGTCCCTGCTGCAGGCTATCGAGGAGTGCCAGCAGGAGCGGCTGGCCAGCCTGCACTACCAGATCCAAGAGCACCAGGCCATGCTGGAGAACTCGGGCATGGTGGGCTACGCCCAGGAGGTGCTGAAGGAGACCGACCACCCCTGCTTTGTCCAGGCTGCCAAGCAGCTGCACAACAGGATCCTCAGGGCCACCGACTCGCTGCAGAGCTTCCGACCTGCAGCCACGGCCTCCTTCAGCCACTTCCAGCTGGATGTCAGCAGGGAGCTGAAGCTGCTCACAGACCTGGCCTTCATCAAAGTGCCCGAGGCCCCCGTCATCGACACGCAGCGCACCTACGCCTACGACCAGATCTTCCTGTGCTGGCGGCTGCCGCAGCACTCGCCCCCCGCCTGGCACTACACCGTGGAGTACCGCAAGACGGACGCCAAGGCCAAAGGGCTGAAGCTGTGGCAGCGGCGCGAGGAGGTGCGCGGCACCAGCGCCCTGGTCGAGTACCTGGACACCGACAGCGTCTACGTGCTCCGGGTGAAGGGCTGCAACAAGGCCGGCTTCGGGGAGTACAGCGAGGACATCTACCTGCACACGCCGCCCGCCCCAGTCCTCAACTTCTTCCTGGACAACCGCTGGGGTTTCAACCGGGACCGGCTGGCCATCAGCAAGGACCAGCGCGCCGTGCGCAGCGTGCCCGGCATCCCCATGCTGTTCGCGGCGGAGCGGCTGATGACCAGCTGCCACCTCTCCATCGACCTGGTCATCGGGGACGTGGCCATCACGCAGGGCAAGAGCTACTGGGCCTGCTGCGTGGATCCCAGCTCTTACCTGGTCAAGGTGGGCGTGGGGCTGGAGAGCAAACTGCAGGAGTGGTTCCAGGTGCCTCAGGACGTGGTGAGCCCCAGGTACGACCCTGACAGCGGCCACGACAGCGGGGCAGAGGACACGACGGTGGACGCGCCCCCGCCCTACGCCTTCCTCACCATCGGCATGGGCAAGATCTTGCTCTCGCATGGCTCGGCGCTCACTTCTCGTGACCCCAACGGCTGCACCGTGCCCTTGCCACCGCGCATCGGCATCTGCCTGGACTACGAGCAGGGCAAGGTGAGCTTCTACGACGCCGTGTCCTTCCGTGAGCTCTGGGAGTGTGGCGTGGACTGCTCGGGGCCTGTCTGCCCCGCCTTCTGCTTCATCGGAGGGGGTGCCCTGCATCTCCAGGAGCTGGTGGCCAACAAGCAGGAGCGCAAAGTGACCATCGGGGGCTTCGCCAAGCTGGACTGA

>TRIM47

ATGGAGGCCACCGGCGGGAGCAGCCGGGCGGTCCCCTCCGCCGCCACCTCGGCGGCGTTGAGGCTAGCGCTGGCCGTGCCGGGTTTGCCCGACGGTCCTTTCGGTTGCCCCATCTGTTTGGATATCCTGAAGGATCCGGTGACGGTGCCGTGCGGGCACAATTTCTGCCAGGGTTGCTTGGGGAAGCTCCGGGGGAGGACGGGCCCCCCCGACGGCGGGGCGGCGGCGGGCGGGGCGGCGGCTGCTCGCTGCCCGCTGTGCCAGGAGCCCTTCCCGGCGGCTCTGCGGCTCCGCAAGAACCGAGCCCTGTGCGAGGTCCTCCCGCTGCTGGGGGCTGCCGGACCCGCGTCCCCCCCCGTCGAGGCGAGCCCAAAGCCGAGCCCACCCGGTGCCGGCTCCCCGCCTCTCACCGCCCCCAACCCGCCGATGGCGCCGGGAGCCGAGGAGGAGCAGCAGCCGGAGAGGGGAGAGGAGAAAGAGGAGAAGAAAGAGGAAGAGGAGGATGAGGAGGAAGGGGAGGAGGGGGTTTTGTGCGACGTGTGCCCCGAGGGGGCTCGGGCGGCGGCGGCTCGTTCGTGCCTGGTGTGCCTGGCGTCCTTCTGCGGGGCGCACCTGGAGCCCCACCGGCGGTCCCCGGCTTTCCGAGCCCACCGCCTGGTCGCTCCCCTCCGGAGGCTGGAGGAAGGGCTGTGCCCCCGCCACCTGCAGCCCTTCGACGGCTTCTGCCGAGCCGAGCAAACCTGCGTGTGCCCCCGCTGCCGCGCCCACGAGCACCGAGCCCACGACGTGGTGCCCCTCGAGCGGGAGCGGGAGCTGAAGGAGGCCCAGCAAGCCAAATTCCTCAGCAATGTGGAGAACGAGCTGGAGGAGCTGGCCGTCACCATCACGCAGACCAAGAAGATGGTGGAGCTCATTAAGGGTGTCGCCACGAAGGAGAAGGAGCGGGTGGAGAAGCTCTTTGCAGAAGCCTCCGAGGTCCTGGCCACCTTCCAGAAGGAGGTGGCTGGCTTCATCGAGGACGGGGAGCGCTCCATGCTGGGGGAGGCCGAGCTCGACCTCCGCTGGAAGGAGGAGAGGCGAGCCAAGCTGGCCCAGTGCAAGCAAAACCTGCAGAACGTCCCCAGCAAGGACACCATCTACTTCCTCCAGGAGTTTCAAGCCTTAAAAATAGCCATGGAAGACAACCTCTCCCCGGCTCCGAGCTTCCAGAACGAGCTCAACTTCACCAAGTGCACCCAGGCCGTGTGCGCCGTCAAGGACATGCTGGCGGCGGTCTGCAAAAAGCAGTGGGACCGCTTGCAGGGGAAAGGCGTCGACGGGCTGAATTTCCAGGAGATGGAGGAAGTGACCGAGTCACGGTTTCCAGACAAGCCGAACAATCCCGCCTGCCTGGAGAGCCGGGATTACTTCCTGAAATTTGCCTTCATCATTGACCTGGACAGCGACACGGCCGACAAATTCATCCAGCTCTTTGGCACCAAAGGAGCCAAGAGGGTGCTTAGCCCCATCCTTTACCCAGAGAGCCCGGTCCGCTTCATCAACTGCGAGCAAGTGCTGGGCATGAACCTCATGAACCGGGGCAACTACTACTGGGAGGTGGAGATCATCGACGGCTGGGTGAGCATCGGTGTCGTCACCGAGGACTTCAACCCCCGCGAAGCCTTCAACCGCTGCCGCCTGGGGCGCAACGAGAGGTCCTGCTGCCTGCAGTGGAACGGACAGAACTACGTGGCCTGGTTTGGTGGCTGTGAGTCTGTCATCCAGCAGCCGTTTTTCCACACGATCGGGGTCTTCCTGGAGTATTCGGAGAAGACTCTGACCTTCTACGGAGTCAAGGACTCCAAGATGACGTGCCTGCAGCAGCTCAAGGTCTCCCCTGTGGGGAAGACTCAGGTAAACCCCTTCCAGAACAAGATCAACTACCACTTCTCCTCTCTGTTCTCGTTAAAGCTGAAGCCGGCCTTCTTCCTGGAGAGCGTCGATGCCCACCTGCAGATCGGGCCGCTGAAGAAGGATTGCGTTTCGGTGCTCAAGCGCCGGTAA

>TRIM50

ATGCTAAAAGGTGGCAGCATGGCTCGGAGGATGAGCATCGATGAGTTGGAAGACCAGCTCCTCTGTCCCATCTGCTTGGAGGTCTTCAAGGAGCCCTTGATGCTGCAGTGCGGGCATTCTTACTGCAAGTCGTGTGTGGTGTCACTGTCTGGAGAGCTGGACGGGCAGTTCCTGTGCCCCGTGTGCCGCCAAACCGTGGACTGCAGCGCCTCGCCACCCAACGTCACACTGGCCCGTGTCATTGAGGCACTGCAGAGCCGAGGCGAGGCAGAGCCCGCCCCAGAGTCCTGCCCAACACACCACAACCCCCTCAGCCTCTTCTGCGAGACTGACCAAGAGGTGATCTGTGGGCTGTGCGGCACCATCGGGAACCACCGCCAGCACAAGATCACCCCCATCTCTACCACATACTGCCGGATGAAGGAGGAGCTGTCTGTGCTGCTAACCGATGTCCACCAGTACAAGAGGAACCTGGATGAACACTTCAGCAAGCTCATCAACAACAAAAGCCGCATTGCAAACGAGGCAGATGTCTTCAAGTGGGTGATCCGGAAGGAGTTCCAGGAGCTGCACAGATACATTGATGAGGAGAAGGCCACCTTCCTGGAGAGCGTTGAGGGGAAAGCAGCCCAGCTCATAACCTCCATTGAGTCCCAGGTCAAGCAGACATCAGATGCCCTGCAGAGGCTGAAGGAGATACATAGCTCTCTGGAGATGCTCAGCAATGAAAACCAGCTTGATTTCATCCGTAAATACAGCTCCTCCCAGTTCAGGTCAGAGCTTCCCAGCCTGCTCCCAGGTGATGGCATCTTTAGCCCCATTTCCTTCAAGCCATGTTTCCACCAAGATGACATCAAGATGACAGTTTGGAAGCGGCTGCACCGCCATGTCCTGCCAGCTCCAGAGATGCTGAAACTGGACCCAGTGACAGCACATCCCCTCTTGGAACTCTTCAAGGGTGACACAGTGGTGCAATGTGGACTCTACCAGCGCCGGGATAGCAACCCCAAGCGTTTTGACTCCAGCAGCTGCATCCTCACCTGCAAGGGCTTCTCCTGTGGCCAACACTACTGGGAGGTGATCGTGGGCACCCGCAACCACTGGCGCGTGGGCATCATCAAGGGCACAGTCAGCCGCAAAGGGAAGCTCAGCAAGTCTCCTGAGAACGGCGTGTGGCTCATTGGCCTGAAGGAAGGCAAAGTCTACGAGGCCTTCAGCACCCCGCGGGCCACCCTTCTGACCGCCCGGCCGCAGCGCATCGGCATCTACCTGCACTACGAGAGGGGCGAGCTGACCTTCTACAACGCCGACAGCCCCGACGAGCTCAGCCCCATCTACACCTTCCAGGCGGAGTTCCAGGGCCAGCTCTACCCCATTGTGGACCTGTGCTGGCCCGAGCGGGGGCCCTACTCGCCCCCTATCATCCTGCCCCCCCCCGGCGCCACCCGGCGCCCCCAGGGGCCGTGCCGCCAGCCGC

>TRIM54

ATGAACTTCGCGGTGGGGCTGAAGCCGCTGCTGGCGGAGGCGCGGAGCATGGAGAGCCTGGAGAAGCAGCTCATCTGCCCCATCTGCCTGGAGATGTTCACCAAGCCCGTGGTCATCCTGCCCTGCCAGCACAACCTGTGCCGCAAGTGCGCCAACGACGTCTTCCAGGCCTCCAACCCGCTGTGGCAGTCGCGGGGCTCCAGCGCGGTGCCGTCGGGCGGCCGGTTCCGGTGCCCGTCGTGCCGCCACGAGGTGGTGCTGGACCGGCACGGGGTGTACGGGCTGCAGCGGAACCTGCTGGTGGAGAACATCATCGACATCTACAAGCAGGAGTCGGCCAGGCCCCTGCACGCCAAGGCGGAGCAGCACCTGATGTGCGAGGAGCACGAGGACGAGCGGATCAACATCTACTGCCTGCGCTGCGAGGCGCCCACCTGCTCCCTCTGCAAGGTCTTCGGGGCGCACAAGGACTGCGAGGTTGCGCCGCTGCCCGCGGTCTACCAGCGCCAGAAGAGCGAGCTCAGCGATGGCATTGCCATGCTGGTGGCGGGGAACGACCGCATCCAGGCCATCATCACCCAGATGGAGGAGATCTGCCGGACCATTGAGGAGAACGGTCGGCGGCAGAAACAGCACCTGGGGCTGCGCTTCGACTCGCTGTACAGCATCCTGGAGGAGAGGAAGAAGGAGCTGCTGCAGAGCATCGCGCGGGAGCAGGAGGAGAAAGTGCAGCGTGTGCGGGGCCTCATCCGCCAGTACGGCGACCACCTGGAGGCTTCCTCCAAGCTGGTGGAGACAGCCATCCAGGCCATGGAGGAGCCCCAGATGGCAGTGTATCTGCAGCACTCCAAGGAGCTCCTGAAGAAGATCACAGACATGTCCAAGGTGTCGATGAGCAGCCGCCCGGAGCCTGGCTACGAGAACATGGACCACTTCTCCATCAATGTGGACTATGTGGCAGAGATGCTGAGGACCATCGAGTTCCAGACAGAGCCACTGGGAGAGGAGGAGGGGGACGGCCCCATGGAGGGCAGCGAGGCTGCGGCGGATGAGGACCGGCTGGACAGCCTGGAGGCACCCGAGGCTGCTGAGGATGTGGGGCCGAGGCAGAAGCCAGCAAGCTCTCCACATGGTCAGCACTGA

>TRIM55

ATGCCCCTGGAAAAGCTAGGGATGAGCACCTCCCTGAACTACAAGTCCTTCTCCAAAGAGCAGCAGACCATGGATAACCTGGAGAAGCAGCTGATTTGCCCCATCTGTTTGGAGATGTTCACCAAGCCGGTGGTCATCTTGCCCTGCCAGCACAACCTGTGTAGGAAGTGTGCCAGCGACATTTTCCAGGCCTCGAACCCCTACCTGCCCACCAGGGGAGGCACGACTGTGGCTTCGGGAGGCCGGTTTCGCTGCCCCTCATGCAGGCATGAAGTGGTCCTCGACCGGCACGGGGTGTACGGGCTGCAGAGAAACCTGCTGGTGGAGAACATCATCGACATATACAAGCAGGAGTCCACAAGGCCTGAAAGAAAATGTGACCAGCCAATGTGTGAAGAGCATGAAGATGAGAGAATTAATATCTACTGTTTGAACTGTGAAATGCCCACCTGCTCCTTGTGCAAAGTCTTTGGTGCCCACAAAGACTGTCAGGTTGCTCCTCTCACAAACGTTTACCAGAGACAGAAGTCCGAGCTGAGTGACGGCATCGCAGTCCTAGTGGGGAGCAATGACAGAGTGCAAGGGATAGTCACACAGCTGGAGGAGACCTGCAAGACGGTTGAGGAATGCTGCAGACGACAGAAAGAACAGCTGTGTGAAAAATTTGATTATCTCTATTCTGTACTGGAAGAAAGAAAAAATGAGATGACACAAATAATCACTAGAACCCAAGAGGAGAAACTGGAACATGTCCGCTCCTTGATGAAGAAGTATGCGGATCATTTGGAAGCCGTGTCAAAGCTGGTGGAATCAGGAATCCAGTTCATGGAAGAACCAGAAATGGCTGTGTTTTTGCAGAATGCCAAAACATTGCTACAAAAAATTACTGAAGCATCTAAAGGATTTCAAATGGAAAAAATAGAGGATGGGTATGAAAATATGAACCAGTTCACTGTGAACCTCAGTAGAGAAGAAAAGATAATAAGAGAAATTGATTTTGACAGAGAGGAGGAGGTGGAAGAGGAAGAGGAGGAGACAGTGGATGGGGAAGATTTGGATGAAGTCCACACAGAGTCATCAGGAGAGGAAGAGGAGGAAGAAGAGAAGGAGGAGGAGGAAGGGGCTGAGAGAGCATCACAGCCACCTCAGCAGGATCCTGAACCACAGAGTGCAGCAGTCGAGCCCCCAGCCGAACCTGCCCCAGCACCACAGCCTGCCCCCCCAGCTGGCCAGACCGGCTCTGAACCGTCCCCCTCAAGGACGAGCGGAGGAAACTCCCGTGAGCAATGA

>TRIM59

ATGCATCACTTTGAGGAAGAGTTAACGTGCTCCATCTGCTACAGCCTGTTTTCAGATCCCCGTGTTCTGCCTTGTTCCCATACATTCTGTAGAAATTGTCTGGAGGGTGTCCTTGATCTGTCAGGCAACTTTTCCATTTGGAGACCCCTGAGAATTCTTCTGAAGTGCCCAAACTGTAGGAGCGTTGTTGAGATTCCTGACTCTGGCACTGAATCGCTGCCGATCAACTTTGCACTGAAAGCTATTATTGAGAAATACCGTCAGGAAGATCACTCTGATGTTGCAACTTGCAGTGAGCATTATAGGCAGCCGCTGAACGTTTACTGCCTTTTGGATAAAAAAATGGTGTGTGGGCATTGCCTTACAATAGGAAAACACAACGGGCATCCCATAGACGACCTTTACAGTGCCTACCTAAAAGAGAAGCAGTCTTCTGGAAAAATTCTCGAGCAGCTGACTGATAAACACTGGGCTGATGTATATTTGCTCATTGAAAAGCTGAAAGAACAGAAATCCCAGTGTGAAAGCGTCATTCAAGATGATAAAAAAGTAGTGGTTCTGTACTTTAAGAAACTTAGCGAGACATTGGAGAATAAAAAACAAGCTCTGCTCTCTGCACTGGATGAAATCAACAGGCAGGTTTTGGAAGAATATGATCCTCTCATTGAGAATTTAAAAAAAATGAGGGAAGAACAGCTTGAATTAATGTCACTGAACACATCTATTCAGAAAGAAGAGTCCCCACTTGTTTTTCTTGAGAAGGTGGATGGGGTGTACCAACGGCTAAAAGCTTTGAAAGAGAAGCAGCTGCCGGATGTTAAACCTGTCGAGATCTACCCCAGGGTTGGGCACCTGTTGAAGGATGTGTGGTCCAACACCGAAATTGGTCAGATCAACAAGATCCTTACTCCAAAGATAAAACTGATTCCAAAAAGGAAATTACAGATCAAAAGCAAGCAAAAAGAAAGAGGAAAATCTGAAGAACTCCCCCCAGCTGTAAATTCTCTAGCAGTCATTCTTCTTTTTGTAATAGCAGCGATAGCTCTGTTTGCATTTTCCAAACTGGTGTCATCATTTGGAATTGAAACTGTTCCCACTTACATTTCAGAATTCTTGCACTCTATTTATCAAGGTTTTTGTGCTCATTTGCAGACTATAGTGGATGCACTGTGCCATAAATTTAATCTTACGGTGAAGTTTTTAGGGATGATTGTTCCTCTTTGGCTATTTCAATGA

>TRIM59L

ATGGAGGCCAGCCTGACGTGCGCCGTGTGCCTGTCCCTCTTCGAGGAGCCGGTGACGCTGCCGCTCTGCTCGCACAACTTCTGCCGGGGCTGCGTGCTCGAGTGCCTGGCCTCGGCGCAGCACCAGCAGCGGGGGCCGGGGCCGGGCCAGGGCCAGGGCCAGGGCCAGGGCCAGGCGCGGCTCCCCCGGGGCGGGCCGGGCCCGGGCCCGGGCCCGGGGCCGGGTCCGGGTCCGGGCGGGGGCCGGGTGCCGTGCCCGCTGTGCAGGAAGCTCAGCCCCCTGCCCCGCGGCGGCGCGGCCGCGCTGCCCGTCAACACCACGCTGGCGGAGGTGGTGAAGCTGTACCGCTCCGGAGCGGCCGGGGAGCCCGGGGAGCAGGGCCCGGGGCTGGGGCTGGGGCCGGGGCAGGGCCCGCTGGCGCTGAGGGGCACCTGCCAGAAGCACCCGGGCCGCCTGGTGCAGCTCTACTGCCGCATGTGCCGCCGCGCCGGCTGCGGGCAGTGCGTGTCCGAGGAGCACCAGGGCATCTTCCACTCCGTCAACCTGCTGGACACGGTGTTCCAGGAGGAGAAATTAACCTTCTTCAGTAGTCTGAAAAAAATGAGAATAATAAACGAGAAGCTGATGAATGAAGTCTCAAGTCATCCAAATGATACTGATATGGTGGTGAATAGTGAAGCAGACATAATTGCACTGGAATTTGGAGAAATTTTCAGAACTCTGGAAATGAAAAAGCAGCAATTGATAGAAGATCTTGAAAATCAAAGAAGTAAAAAAGAGAAGGAGTTTCAGATTTGGAAGAAGATGAAGGAAACTCACAAGAAAACCATTGAGAATTTTTTGAAGGACTGTGAAAAGCTTGTACATGAATGTGACCCTCAGCGTTTCCTAGAGGTGGCCTGTGGCTTGAATGCAAGGATGAAGACTCAGCTTGACCTGATGAGTATAGCATCCAGCTATGAAAAACCACCAGAGTATACTCAGAAGAAAATGGATATTAAACCTGTGGTTAATGAAATCTTGGCTTTGAAGCTAGTGCCAGTTAACGTAGACATGGCTAAAGACCTACCTTCTGGAGGAAATCAGAACTCAACGAAAAATACTATAAAGCAATGGCAGGACCAGAAGAATATGCCCAAAATGTTTATTCCAGTAGCAGGACAAGAGGAAGCACTGACAGACGGTGGCAGGATCTGTACTCGCTTAATGTCAATATCAGAAATGTCAGCGTTTCAGAATATGAGTCATGAGGAGCTACGTTATAAATACTACATGGAACGTCAGAAACTTGCTGATGAGTTCAAGACACAAACTTTACCTGCAAATAAGAAACATAAATTTGTGGCTGCTGAGGCTTTGAAGGATAAGTCCTCAGGAATTCCTTCTGTATCTTTACCTACCACAGCAAATAACACAAATGGAGTGAATATGGGAATCCTGCAAAAAGAAGGTGGTTTTGATGAGTTAAACTTTTTTGGAACCGGTAATCATAGGATCCCATACACCGCTACTAACTTTTCTGAAAAAAATGGTAACTTAAGCTTAGTTCATGAAAGAGGTTCTGAGGAAACAACCACTCCAGCCTTATCAGAAAACACTAAAGATTTATTAATGAGAGAAAAAATGCCAATGCAGTCATCAGCAGTTACTGTTTCGAATGGAGTGGACACAAATTCTAGCATTCTGGGAGGCGTAGCAGCGTCAGTAGCTGTTACTGTTTCAAATTCAGAATTTTTAGATGTTTCTGCAGAAAGACCTTCTGCTTCACCCTTTGCTTTTGGTGCATGTAGCAACTCACTACCCAGAGTTACTAAAGATGCAGCTACATTTTCTTTTAAAAAGGAAGCCAGTAAATATGTTTTCCCTAAATTTTATCTAGGGAAATGTGATCGTGAAGCTAAAGCAGTTAACCAGGGTGGAAACAAATTCGGAAAACATAGTTCTGTAACTAAAACCACAGTTTCTGATGCTTCAAACAGTTGTAATGTAGAATTAGCAGAAAATAAGAAAACTTGTTTTTCATTTCCCTTTGACCATTCAGAAAGAGATTGTTCTGCAGTATCAGGAGTCAGCAATTCATCCAAGATTTTACCATTATCCTCATTTTCTAACCAATCAGAGAAGCCAGCTGACCAAAATACATTATCTCACATGGTAGAAAATGCATTTTCTCCAAAGAAAACTGTAGAAAATGATACGCTGAAACCATCTGTCTCAGTGGAGCAAAAAGCTAATGCATCAGAATCCATCACAACAGCACCTTGCAGTACTTCAGAAACCGGCATTGCAGCTGGGGTGGATGATGTCTCAGAGTCTTCCCTTCTCCCCAGCACGTGTGTATTTTCCTTCAAAAATAACTGTTTTCAGTTGCCATCACCAGTGTTTTCATTTGGAAGTATTGTTAAAAATACAACTGATTCACTGACTTCCTCCTCCATTTTTTTGTCTAGCAATGGTACTGAAAAAAGTGAACAAGAGAAGATGAAACCTGTTGACAAAACACCTCCAAATCTAGTAAAGTCTGCATCTCCAGAGTGTACAGAGACTGCATCTAGACACAGTCACCCAAAAGATGAAGGTTCTTTTCCAATGGGCTCATCTAAGAAAATAGAAAGTGCAGAAATACTTGCTGATAGTAATTCTTCTTGTAGTCCACTTTTATGTTCAGCTGTCCTTCCTGCTAAAGATGAGAATGCATCATCTGATCACCTGACCATTACTTCAACACAACAGGAAGTAAAGGTAAAAGATCAAGGAAGTATTGCTGAAAACAACTGCTCTATTCCTGGGAGGGAAGATGAACTTAAGTCCAGAATGCTTCAGAATGCAGCTTGTTCTGCTCCGGGCATGTGCAATGATTCAGTTTCAAGAGCTTCTGTACTTACAGTAAATGAGTCTGGAGGAATGCTGAGTGACAGTGATTCTGACACTGAAGCGCTAAGTCAAACGTCTGTGTCTAGTGATACCAGCAGTGTATCAGAATATTTTTCTGTTGCAGAAGACAAGATACCTACTAGAAGAAAATCAGAGGCATGA

>TRIM62

ATGGCTTGCAGCCTGAAGGACGAGCTGCTGTGCTCCATCTGCCTGAGCATCTACCAGGACCCGGTGAGCTTCGGCTGCGAGCACTACTTCTGCCGGCGGTGCATCACCGAGCACTGGGTGCGCCAGGAGCCCCAGGGTGCCCGCGACTGCCCCGAGTGCCGGCGCACCTTCGCCGAGCCCACCCTGGCGCCCAGCCTCAAGCTGGCCAACATCGTGGAGCGGTACAGCGCCTTCCCTCTGGACGCCATCCTGGGGGCCCAGCGCAGCCCCTTCCCCTGCAAGGACCACGAGAAGGTCAAGCTCTTCTGCCTCACCGACCGCGCCGTCGTCTGCTTCTTCTGCGACGAGCCTGCCGTGCACGAGCAGCACCAGGTCACCAACGTGGACGATGCCTTCGAGGAACTGCAGCGGGAGCTGAAGGAGCAGCTCCAGGGCCTGCAGGAGAGCGAGCGAGGCCACACCGAGGCCCTGCACCTCCTCAAACGGCAGCTGGCAGAGACCAAGTCATCGGCCAAGAGCCTGCGGGCGACCATTGGGGAGGCCTTCGAGCGGCTGCACCGGCTGCTGCGGGAGCGGCAGAAGGCGATGCTGGAGGAACTGGAGGCGGACACGGCGCGGACACTGACCGACATCGAGCAGAAGATCCAGCGCTACAGCCAGCAGCTGCGCAAGGTGCAGGAGGGCAGCCAGATCCTGCAGGAGCGCCTGGCCGAGGCTGACAAGCATGCCTTCCTGGCCGGCGTCGCCTCCTTGTCCGAGAGGCTCAAGGGGAAGATCCACGAGACCAACCTCACCTATGAGGACTTTCCTACCTCCAAGTACATGGGCCCGCTGCAGTACACCATCTGGAAATCTCTCTTCCAGGACATCCACCCGGTGCCGGCGGCGCTGACGCTGGACCCCGGCACAGCCCACCAGCGCCTCATCCTCTCCGACGACTGCACCATTGTGGCTTACGGCAACCTGCACCCGCAGCCGCTGCAGGACTCGCCACGGCGCTTCGACGTGGAGGTGTCGGTGCTGGGCACAGAGGCTTTCGGCGGCGGTGTGCACTACTGGGAGGTGGTGGTGTCTGAGAAGACGCAGTGGATGATCGGGCTGGCGCACGAGGCCGTCACCCGCAAGGGCAGCATCCAGATCCAGCCCAGCCGGGGCTTTTACTGCATCGTCATGCACGATGGGAACCAGTACAGCGCCTGCACCGAGCCCTGGACCCGGCTCAACGTCAAAAGCAAGCTGGAGAAGGTGGGCGTCTTCCTGGACTATGACAAGGGGCTGCTCATCTTCTATAACGCCGACGACATGTCCTGGCTCTACACCTTCCGGGAGAAGTTCCCTGGCAAGCTCTGCTCCTACTTCAGCCCCGGGCAGAGCCACGCCAACGGCAAGAACGTCCAGCCCCTGCGCATCAACAC

>TRIM63

ATGGGCTGCCATGTGCATTTTGGGGTGCAGAGAAGCCCTGTGTGCAGAGGGGGCCGCACACACCGAGCATGGTGCTGCCATCCACCCAGAAAGCCCCCCACCCACCCTATTTGTGCTCAGCACCCCAAAAGCGCTCACATTTTATGGGTACAAACACAGATGCTCCACACAAACCACTCTACCAGCTTGGTGTTGCCCATTCCTCACCCTAAACCCCCCGTAGGACCGAGTTCCACCTTATACCCCCCCACGGGGCCCCGCTGCCCCCCAGCACGGGCGGCCGGGCCACGCGGACAGCCTGTCCCCAGCTTGTTTACCGCCGGCCTTGCGCCCCGATTGTCACCGCCATGTGACAGGTGGCAGCGCCTCGCCGGACACCCGAGCCGCCCGGCAGATTTTTGGGCTCCACGTGACGCCGAGCGAGTGATAAAAAGCCGCGCGAGGGGCGCCAGCGGCAGAAGCGGGGGCTGCTCCGACGAGGTGCCATATAGGATGGATTTCCAGCCCAGCATCCTGCGGGATGGCAGCCCCATGGAGAGCCTGGAAAAGCAGCTGCTCTGCCCCATCTGCCTGGAGATGTTCAGCAAGCCCGTGGTGATCCTGCCCTGCCAGCACAACCTCTGCCGCAAGTGCGCCAACGACGTCTTCCAGGCTGCCAACCCCTGCTGGCAGAGCCGGGGCAGCATCATCCCGGGGGGCCGGTTCCGATGCCCCTCATGCCGCCACGAGGTGCTGCTGGACCGCCACGGCGTCTACGGGCTGCAGAGGAACCTGCTGGTGGAGAACATCATCGACATCTACAAGCAGGAGTGCTCCAGCAGGCCCATGAAGAAGGGGGAGCACCCCATGTGCAAGGAGCACGAGGACGAGCGGATCAACATCTACTGCGTCACCTGCGAGGTCCCCACCTGCTCCATGTGCAAGGTCTTCGGGGCCCACAAGGACTGCGAAGTCGCCCCTCTGCAGAGCGTCTTCCAGGGCCAGAAGAGCGAGCTCAACAACTGCATCTCCATGCTGGTGGCGGGCAACGACCGGATCCAGACCATCATCTCCCAGCTGGAGGACTCCTGCCGCAGCACCGAGGAGAACAGCGAGGCAGCCAAGCGGGAGCTGTGCGCCCGTTTCGACACCTTCGTGGCGCTGCTGGAGGAGAAGAAGACGGAGCTGCTGGGGCGCATCAGCCGCGAGCAGGAGGACAAGACGGGCTTCGTGCAGGGCCTCATCCACAAGTACAAGGAGCAGCTGGAGAAATCCAGCCGGCTGGTGGAGACGGCCATCCAGGCCATGGAGGAGACCGGGGAGGCCACCTTCCTCATGAACGCCAAGCAGCTCATTAAAACGATCGTGGAGGCCTCGAAGGGCGGCAGGCTGGAGAAAATCGAGCAAGGCTACGAGAGCATGGACGCCTTCTCGGTGAGCCTGGACCACCTCGCCGACGCCGTCCGCGCCTTGGACTTCGAAGCAGATGAGGAGGATGAGGAGTTCTATGAGGAGGTGGAGGAAGAGACAGAAGGGGACTCAGCGCCCGGGAGGATGGTGACAGCCCCCCAGTAG

>TRIM65

ATGCCAGGAAGCTCCTTCCAAAAAAAAAAGAGTGAAATTAAATACAGAGGAACGTGTATCCATGGGAACACGCTTGCTTCGGATGCCGTTAGCCTCTGCCATGTGTCTCCCCGTGCTGTCACCATGGCACTTTCTCCTCCCGCAGCGCCCTGCTGCTCCCAGGGGGGCACAGGGCAGAGCCCGCATGGCCGAGGGGCGAGCGACCCCCCACTGGGTGTCCCTTTAAGGGTGCACCTGGCTCGGAGCCCTGACCCGCCTCTGAGCTCGGCGTCACCCAGCTCTTCCTCCTTCCCCAGCGGGGTTTCTGTTTCTCCAGCTTCTTTCCCTCGCTGCCCCGATCCCTGGCACGAGATGGCTTTGTCCATCTCGCCCAAGCTGGAGGAGAAGCTGGTGTGCTCCATCTGCCTGGAGCTCTTCAAGGTGCCCATCACCTTGCCCTGCGGCCACAACTTCTGCAAGCGCTGCATCAGCGACCACCAAGGCAAGCAGGAGCAGGCGGCCGCCGGGGCCAAGCAGGGCTTCTCGTGCCCCGAGTGCCGCCAGAGCTGCGCGCCGCAGCTGGAGCTGAAGAAGAACGTCACCCTGAGCAAAGTGCTGGAGCTGGTGCGGGCGAGCAAGACGGGGGTGAAGCAGTGCGAGGTGACCCCCGGCGGGCTGTGCCCGCGGCACGGGAGACCGCTGGAGCTGTACTGCGAGGACGAGCAGCGGTGCATCTGCTGCGTCTGCACCGTGCAGCAGTGCCAGCGGCACCGGCGGGCGCTCCTCGAGGACGTGCACTCCAGGAAGCAGGCCCTCCTGGAAAAGTCTGTGAAAGAAGCCCAGGAGGAGTCGGAGAAGATCGAGCGGGCGCTGCAGGAGCTGGAGGAGCGAACGCAGAGCATCAAGGACTCCTCCGAGGGGCTCAGATCGGTGATCCTGGGCAAATTCGCCCACCTGGAGAAAAGCCTGCAGGCTTTCCAGTGCCAGATGGTGGCCAAGGTCGAGCAAGAGCTGTCGGCGGCGCTGAGACGCGTGGAGGAGAACTCCAACACCCTGAAGGGGCACCTGGACACCCTCAGACAGCACCAGGAGCAGGCACGGGACCTGCTGGTCTCCACCACAGACCACAGGACCTTCCTCGAGGAATTCCCCCTGCTCCCAGCCTGGGAGAGCCTGGCGGTGCCACCCCCGGTGCAGTTCGATGCGGCCGGCGTGGTGGAGCCCCTCAGCGAGATCCTCGCCGGCATCTCCCGGCTCCTGCTGGAGGATTTGCCCGGCGCCGTGGCCCCCAAATCCCCTGACCCCATTGTCCCAGGCCCGGTGCAGCCCAAGGGGACAGAGATGAAGGTGGCGACCCCTCTTCCCAAGTGCCAGATCCGAGCTGAGTTTCTGAAGGACCACCGCAACCTCACCTTCGATCCTGACACGGCCAACAAGTACCTGGAGCTGTCCAAGGGCCAGCGGCGAGCCCGGCACGGCACCGGCGCCGCAGGCGGGTGGCAGGAGCGGGGCAGCCCCTTCGAGCCCTGGCAGGTGCTGTGTGAGCAGGGCTACGGGCAGGGCTGCCACTACTGGGAGGTGGCCATCTCCAGCCACTCCGTCATCCTGGGGGCCACGTACCGCAGCCTCCCCCAGCGGCAGCCGCCGGGCCACAAGTTCAGCATCGGGCTGGACGGGGGCTCGTGGGGGCTGCAGGTGCGGGAGGACGGCTACCTGGCCTGGCACAAGGGCCGGGAGGAGAAAATCCAGGAGAGGCTTTACACCCAGCTGGGGGTCCGCCTGGATTACGGCCGGGGGCTGCTTTCGTTCTACGGGCTCGGGGAGGAGACGCGGCTCATCCACTGCTTCCACGCCGTCTTCACCGAGCCGCTCTACCCCGTCTTCTGGCTCTGCGAGGGCCGCGCCGTCACGCTGGGCCGCAGGGATCAGCCCCAGCCGGCTCCCCAAGCACCATCATCGGGGCAGGATGGGGTGCAGTGA

>TRIM66

ATGAGCCATTTTGTGACTCTCCACGTTTTTGATGTCAAATTTCAGCCAACAGCAGTGGGGACTCTGGATCTGCTGAGGAATTGCTTGGTATGCAAACGGGATCTGGGCATGAGAGACCCCAGAATGCTACCTTGCCTTCATTCCTTCTGTAAGGACTGTCTTCCAGGTCTGATTCAAGGATATAGCTGTATTCCCACTGGATATGAAGTTCTATATGAAGGTATCCTTTCCTGCCCTGTGTGTAAACAGACCTGCTTTGCAAGAGATGTAGTTGAGAATTTCTTTCTCAAGGACTTTCACACTGATAAGTCAGCTATGGCAAAGAGCTGCTCCATGTGTAAAGAGAAGAAACCTGCTCACAGTCTCTGCACGAGCTGCAATAAGTGGTTGTGCAGCACATGTACAGAGGAACACAGACATGGCAATGAAACTGGAGACCGCTTCCTGTCTGTATCCCTGAAGGGCTGCACAGCAACTGAAGACGAGGCAAGTGAATTTTCCTTATTTTGTCCAATGCATGCTCAAGAGCCACTCAAGTTGTTCTGTGAGACCTGTGATATCCTTACATGCCACAGCTGCCTTCTGACAGAGCATAAGGAACACAGGTTCAGACATCTTGATGAAGCTTTGCAAAATCAGAGAGCTATCCTGAAAAATGTCATAGCTAAAGTGGAAGAGAAAAAAAATGGAATTCAGGTTTCAGCTAAACAGATTGAAGACAGGTTGCTTGAAGTAAAACACTTATACAAAAAAGTAGAAAATCAAATAAAAATGGCCAAGATGGTTTTGATAAATGAAATCAATAAACGAACAAACATCCTTCTTGAACAACTTGAAAAAATCACAAGTGAAAGGAAGCAGAAATTGGAGCAGCAACTGCAAGGTGTTGTGGTTTTAAGCAGACAGGTTGAACATGTGCAGAACTTCACCAGTTGGGCAGTGTGCAGTAAAAACAGCATCCCATTTCTCTTCAGTAAAGAACTGATTGTATTTCAGATCCAGCGCTTGTTAGAGACAAATTGTAACACAGACGTAGGCCCTCCACTGAAGATCAGATTTACTTGGGATCCATCCTACTGGACTAAACAGCTGTCCAATTTTGGAGCTTTCACTATGGAAGGTGGACACATTTCTCATTCAGATGTGCTACTCTATGGAAATGTACAAGGATTACAGACATCCTTGTATCACGGACACTATTCCCCAGCATCACAGCTGGAGCCTGTGAATAGCCAGCCACATCAGCTTCCACCTGCAGTTCAGTGTCCTATGCCCATGTGTTGCTCACACTGCCTCAGTGTCCCTCATCCAAACAAAGCTCAGCCTTCTCACCAAAACATAAACCGCCACCAAAATTTTCAACATCCCGAACTGCATCAGCAGCAGTTTCCTCTGCAGTACAGCATGCAGCAGCGTGACAAAGAGCAAAGGGATGTTCCCCCGCCTCTGAAGTTAACACAGCCTAGGCTGGAGCAACAGTCACGACCAGAGTCAGAGAATACATCAGGGAAGATGGGAAAGCATTTACCACCCCAGCAGCTGCAGCAGACTGCACCTCTGGGTTATGCTGTTGTATCACATGAGGCTCAGCAAGTTCACACAAGCCATCCACAGTCATTCCGCACACAGACTGCTTTGCAAACATCAACTGTGCAAGTGCAGCTTGGCCATCTTCAGAAGATAAAGTCTAACCACTTGCAGCAGCCACCACAGCAGCAGCAGCTGCCTCCAGCATCGCCACTGTCAGGTCAGAATGAGAACACCCACAAACAAGTCATCCAGCAGAGCCTGGACATAATGCACCACCAGTTTGAACTGGAGGAAATGAAAAAGGATCTGGAGCTTCTTCTCCAGGCCCAGGGACAGTCCAGCTTCCAGCTGAACCAACCCAAGCCAGCTCAGCATGTTCAACAAACCATAGTTGGTCAGATCAATTACATAGTGAGGCAGCCAGCCCCGGTTCAGCAGCAAATCCAAGACGAATCTCCAGTCTGTGAGAGTTCCCCTGAACTCGATGTCCAGAAGCCTGTAGTTCCTCTTGACAGAAGTGATATTCCCTCCCTGTCACAGTCATTAGATGAAGAAGCCAGTGTCAGCAGCCACTCCCCTGAGTCAACATTGCAACAATCAGCTTACAACCCAGTGAGAAAGCGTTCAGCATCATTAAGCATTGTGGGCTTTTCAAACGATTTAGAAATGGAACTACCTTCAACAAGGTTATCCAGGTCCGCTGATCCACAGATACAAGATGTGGCTGCTGTAGCACTTGGTTCATCCCCGAACACCCGTTGTGACTGTGATACTCCACCAGAACCAGTACCCAGCTACAGCTTAGTACTGGGCAGAGCTCCAGGCGACTTGAGCCCTGGCCTTGCACCAGGTGATACGCTCCAGAGCAGCGCTAAGTGCAAGCTGGAAAATGAAGACTTGAATACTGTGGATCATCCTCTAGAAAACAGCATGGCCTCTGATGGGCAAGATGTAGTAAATGAATTAAGTCTTTCTATGCAAAAAGTGCTGGAAGAACCTATTAATCTTTCGGTCAAAAAATCTCAGCGTTGTACTTCTCCTTCTGAAGTGCTCAGCAACAATTCTTCCCTGCCTGTGAATGACAGAATGAGACAACTTAGAAATGAAGAAGATTCCAATAACTGTGAAAAGGAACATTTAGAAATGGATATGAAAAGCAATCAGGATGTCAGATCTGGCCCTAGAGAGCTGAAGATTCCTTATGTGAGACTGGAACGTCTGAAGATACATGCATCAGAATCTGGGGAGCTCCCAGTTTTTAAGCTCCAGCCACAGGATAGTGAGCAGGAGGGCAGTTTCCTCCTGATAATTGAATGTGGGACCCAGTCTTCAAGTATGGCTATAAGAATAAATAAAGATGGTCCACCTGAAGGACTGAAATGCAAAGAGGAGAGCATGGAAGACAGAAAATTTGTTATAACCCAGGCTGAAGGACAGATACAATCTCCTCCTGTAGATACTCTACCTTCTGATCAGAAGTTCAGTAATGGTACCTCCCTCACGATGAAAAAATCTCCAGTCACTCAGGAAGTCAATACAATAGAGAATGAAGATTTCTGCGCTGTGTGTCTTAATGGAGGAGAACTGTTGTGTTGTGATCACTGCCCCAAGGTCTTCCATCTCTCCTGCCATGTTCCAGCCCTCCTCAGCTTTCCAGTGGGCGAATGGGTGTGTACGCTTTGCCGTAATCCAGTGAAGCCAGAGGTAGAATATGACTGTGAAAACACACGCTACGGCCACAGTTACAATGCACAGTATGGCCTTGATGATTATGATCAGAAGAAGTGTGAAAAGCTGGTGCTTTCCCTGTTCTGCAGTAGTCTGAGCCTCCCATTCCATGAACCCGTCAGCCCTCTGGCTCGACATTATTATCAGATCATAAAAAGGCCAATGGATTTGTCAATCATACGAAAAAAGCTGCAAAAAAAGGACAAGTTCCACTATTCTGCACCTGAGGAACTTGTGACTGATGTGCGTCTCATGTTTTGGAACTGTGCAAAGTTCAATTATCCAGATTCAGAGGTTGCTGAGGCTGGACGATGCTTAGATGTATTCTTTGAGAGCAAACTGAAGGAGATCTATCCAGATAGGCACTTTCCTTCAATGCAACAGGATGACTCTGATTCTGAAGAGGTGGAGAGCCAGAATAGCAAAATGCCCCCCCAAGATTTTCAGTGGCCATCGTATGGACAGGAGTGCATTCAGCCTAAAAGGAGACGGCGCCATGCTGTAAGATTGCAAAAACAAAAGCATTTGGTTGCTAAGTGGCCACCCTGCTAA

>TRIM67

ATGGAAGAGGAGCTGAAGTGCCCGGTGTGCGGCTCGCTCTTCAGGGAGCCCATCATCCTGCCCTGCTCGCACAATGTCTGCCTGCCCTGCGCCCGCACCATCGCCGTGCAGACCCCGGAGAGCGAGCAGCACCTGCCGGCCCTGCTCCACCCGCGGGGCACCGCCGCTCCGGCCCCGACCCCAACAACAACAACGACAACCCCGGGGCCGCCCGGGGGCTCCGCCGCCCCCTTGGACCCGGAGTGCTCGGCGGGCGGCGGCGGGGACCACGCGGACAAGCTGAGCCTGCACAGCGAGACCGACAGCGGCTACGGCTCCTACACGCCCAGCCTCAAGTCCCCCAACGGCGTGCGGGTGCTGCCGCTGGTGCCCGCACCCCCGGGGGGAGCAGCAGCAGCAGCAGCTCCGCGGGGCGCCGTCCCCAGCCCCAGCCCCATCCCCGGCCCCGTGGGCTCGTCCCTCACCTGCCCGCAGTGCCACCGGAGCGCCTCGCTGGAGCAGCGCGGCCTCCGCGGCTTCCAGCGCAACCGGCTGCTGGAGGCCATCGTGCAGCGCTACCGCCAGGGCCGCACGGCCAGGTGCCAGCTGTGCGACCGCAGCCCGGCCGAGCCGGCGGCCGTGCTGTGCGAGCAGTGCGAGGTGCTGTACTGCGCCGCCTGCCAGCTCCGCTGCCACCCGGCCCGCGGGCCCTTCGCCAAGCACCGCCTGGCGCCGCCGCCCGGCCAGCCCGGACCCCCCCCCGGGGCCGAAGGGAAAGGGGCGGGCGGCGGGCGCAAGCCGGCGACGTGCGCCGAGCATGAGCTGGAGCAGTACAGCATGTACTGCCTGAGCTGCCGCAGCCCCGTCTGCTACCAGTGCCTGGAGGACGGCCGGCACGGCAAGCACGACGTCAAGGCCCTGGGAGCCATGTGGAAGCAGCACAAGGCACAGCTCTCTCAGGCTTTAAATGGTGTTTCAGATAAAGCAAAAGAAGCTAAAGAATTTTTGGTTCAGCTGAAAAATCTATTGCAGCAGATACAGGAGAATGGGTTGGATTACGAAGCCTGTCTCGTTGCTCAGTGCGATGCCCTGGTCGATGCGTTAACACGACAAAAGGCCAAACTGCTCACAAAAGTTACCAAGGAACGTGAGCACAAACTAAAAGTTGTTTGGGACCAGATAAATCACTGTACGCTGAAGCTACGTCAGTCAACAGGGCTTATGGAGTACTGCCTAGAAGTTATCAAAGAAAATGATCCCTCTGGGTTCTTTCAGATTTCAGATGCTTTAATCAAACGTGTTCAGGTATCCCAGGAACAATGGGTCAAAGGAGCCTTGGAGCCAAAAGTGTCTGCTGAGTTTGACCTGACATTGGATAGCGAACCTTTACTGGAGGCGATTCACCATTTGGATTTTATTCAGATGAAATTGCCACCCGTCCCATTACTGCAGCTGGAGAAGTGTTGCACCAGAAACAACAGTGTGACATTGGCCTGGAGGATGCCACCTTTGAGCCACAACCCGGTGGAGGGTTATATCTTGGAACTTGATGATGGAGATGGTGGCCAATTCCGAGAAGTATATGTTGGCAAGGAGACTCTCTGCACTATTGATGGCCTTCATTTCAACAGCACCTATAATGCAAGAGTCAAAGCTTTTAATTCATCGGGAGTTGGTCCTTACAGCAAGACAGTTATCTTACAGACATCTGATGTGGCGTGGTTCACCTTCGACCCCTCCTCAGCTCACAGGGACATCGTGCTGTCAAATGACAACCAGACGGCCACCTGCAGCAGCTACGATGACCGGGTTGTTCTGGGCACGGCTGCCTTCTCCAAGGGCGTGCATTACTGGGAACTGCACGTGGACCGCTATGACAACCATCCTGATCCAGCCTTTGGCATTGCCAGGATTAATGTCGTCAAGGACATGATGCTGGGCAAGGACGACAAGGCTTGGGCCATGTACGTTGACAACAACCGCAGCTGGTTCATGCACTGCAACTCTCACACCAACCGGACTGAAGGAGGAGTCTCTAAAGGTGCCACCGTTGGTGTTCTGCTGGATCTGAACAAGCACAACCTGACCTTCTATATAAATGGGCAGCAGCAAGGACCTCCAGCTTTTGAAAACATTGAGGGTGTCTTCATGCCTGCACTGAGCCTCAATCGAAACGTCCAGGTGACACTGCAGACAGGACTGGAGGTGCCACAATGTGTAAAGCAGCCAAAGTTGCCCAGCAACTAA

>TRIM71

ATGGCTTCGTTCCCGGAGGCCGACTTCCAGATCTGCCCGCTGTGCAAGGAGATGTGCGGCTCGCCGGCTCCCCTCTCCTCCAACTCCTCCACCTCGTCGTCTTCCTCGCAGACCTCCAGCTCCTCCGGGGGGGTGGAGGAGGAGGCGGCGGGCGGCGGCTCCTCGTCCTGCGGGGGGGGCCCTCCGAGGCGGCTGCACGTCCTGCCCTGCCTGCACGCCTTCTGCCGCCAGTGCCTGGAGGCGCAGCGGCACCCCGGCGCGGGGGACGCGCTCAAGCTGCGCTGCCCCATCTGCGACCAGAAGGTGGTGATCTCGGAGCCGTCGGGCATGGACGCGCTGCCCTCCTCCAACTTCCTCCTCAGCAACCTGCTGGACGTCGTGGTGGTGGCGGCGGCCGCCGACGAGCACAAGAACGGCCGCCCCGTCGCCCCCGGCCCCTCCGCCGCCGGCTCGGCCCCGGGGGTGGGCGGCGGCAACAACCGGCACCACGGCCGCCCCCCGCCGCACCGCTCGGCTCCGCCGGGCTCCTCGCCCGCCGCCGCCGTCTCCTCGTCGTCGTCCTCCTCCTCCTCGTCGTCCGCGGCGCCTTCCTCGACGTCCTCGTCCTCCTCCTCGTCGTCGGGGGGCGGCGGCGGCTCCTCCACGGCCGCGCTGCTGCTGCGGCGGCCGCACAGCCGGCAGGGCGAGCCCCGCTGCAGCTCCTGCGACGAGGGCAACGCCGCCAGCTCCCGCTGCCTCGACTGCCAGGAGCACCTGTGCGACAACTGCGTGCGGGCTCACCAGCGGGTCCGCCTCACCAAGGACCACTTCATCGAGCGCTTCGCCGCCGGGCCGCCCCCCTCCGCTGCTGCTGCTGCCGCCGGGCCGGCTGCGCCGCTCGCCCTCAGCCCGCCCTACCCCGCCTCGCCCTACAACATCCTCTCCGTCTTCCCCGACCGGGCCAGCTACTGCCAGCACCACGACGATGAGGTACTGCATTTCTACTGTGATACTTGCTCTGTCCCCATATGTCGGGAATGTACCATGGGACGACATGTGGGTCACAGCTTTATCTACCTCCAAGATGCTCTTCAGGACTCCAGGACTCTTACCATCCAGCTCCTGGCGGATGCTCAGCAAGGACGACAGGCTATTCAGTTGAGTATTGAACAGGCCCAAGCGGTGGCAGAGCAAGTTGAGATGAAAGCTAAGGTGGTACAGTCTGAAGTCAAAGCAGTGACAACTAGACACAAGAAGGCCTTGGAAGAGCGGGAGTGCGAGCTGCTCTGGAAGGTGGAAAAGATTCGTCAAGTCAAGGCTAAGTCACTCTACCTGCAAGTTGAAAAGTTACGTCAGAACCTAAATAAGCTGGACAACACCATTAGTGCTGTTCAGCAGGTCCTGGAGGAAGGTCGTACCATGGATATTCTTCTGGCCCGGGACCGTATGCTGGCTCAGGTGCAGGAGCTGAAGAATGTGAGGGGCCTTCTCCAGCCCCAGGAGGATGACAGGATCATGTTCACACCCCCTGACCAGGCCTTGTATATGGCCATTAAATCCATGGGCTTTGTTAGCAGTGGAGCTTTTGCTCCTCTGACCAAAGCCACTGGGGAAGGCCTCAAGCGTGCGCTGCAGGGCAAAGTGGCCTCTTTCACAGTGATAGGCTATGACCACGACGGTGAGCCTCGCCTTTCAGGAGGCGACATGATCTCTGCAGTGGTGATGGGTCCAGATGGAAACCTTTTTGGGGCAGATGTCAGTGATCAACAGAACGGGACCTACCTGGTCAGCTACCGTCCACAGCTAGAGGGAGAGCACCTGGTGTCTGTGATGATGTGCAACCAACACATTGAGAACAGTCCCTTCAAAGTCATGGTGAAATCTGGGCGCAGCTACATTGGTATCGGGCTGCCTGGGCTCTCGTTTGGCAGCGAGGGAGATAGCGACGGCAAGCTCTGCCGCCCCTGGGGGGTTAGCGTAGACAAAGAAGGCTACATCATTGTGGCTGACCGCAGCAATAACCGCATCCAGGTGTTCAAGCCCTGCGGGACTTTCCACCACAAGTTCGGCACGCTGGGCTCCCGGCCCGGCCAGTTCGATCGTCCCGCTGGTGTGGCCTGTGACATCTCGCGTAGGATCGTCGTGGCTGACAAGGACAATCATCGCATCCAGATCTTCACCTTCGAAGGGCAGTTCATTCTGAAGTTTGGGGAGAAAGGAACGAAGAATGGGCAATTCAATTACCCGTGGGATGTGGCCGTCAACGCAGAGGGCAAAATCCTGGTCTCTGACACGAGGAACCATCGCGTTCAGCTGTTCGGGCCCGATGGAGCGTTTCTTAACAAGTATGGCTTTGAAGGGGCACTCTGGAAGCACTTTGATTCCCCCAGAGGTGTGACTTTCAATCACGAGGGCCACTTGGTCGTGACAGACTTCAACAACCATCGCCTGCTGGTCATCCACGCCGATTGCCAGTCAGCACGCTTCCTGGGCTCGGAGGGCAGTGGCAACGGGCAGTTCCTGCGCCCGCAGGGAGTGGCAGTGGATCAAGAGGGGCGCATCATTGTGGCCGATTCCAGGAACCACCGGGTGCAGATATTTGAGTCCAATGGTAGCTTTTTATGCAAGTTTGGCACTCAGGGAAGCGGCTTTGGTCAGATGGACCGTCCTTCAGGTATAGCCGTCACCCCTGATGGCATGATTGTTGTGGTCGACTTTGGAAACAATCGAATTCTCGTCTTCTAA
