# Supplementary material for: Evolution and expression of the duck TRIM gene repertoire
Source: Front Immunol. 2023 Aug 9;14:1220081. doi: 10.3389/fimmu.2023.1220081 (PMC10445537; doi:10.3389/fimmu.2023.1220081)
Supplement: Supplementary File 4 — Amino acid sequences of duck TRIM or TRIM-like genes used in this study in fasta format. [file DataSheet_4.docx]

>duFSD1L

MGEQREDLQRIVSTLANKKDEIVNFIDILKQTIRNVQVNSSNVFRELDKEFDALHSVLDEMKESMANTIQQERTEKIQALNNQLGQCSSALESSEELLECATTILNIKNPTEFSKAARQIKDRITACPAFRICLKPTISDNMSHLMVDFTLERHVLQALKFLPVPKVPEINTAACLVADNCVTVAWKMPEEDSRVDHFVLEYKKTDYDGLARVKDEQPWEVIDYVKDYVYTLSGLKFDKKYMTVRVQACNKALAGDYSDPVTLETKAFVFGLDDTSSHLNLKVEENYVEWDPTGGKGQEKAKGKENKGSGQYPVLKSNKRSGASPKRTSASRSAAKGCRDRFTGESYTVLGDTAIESGEHYWEATAQKDCKSYSVGVTYRNLGKFDQLGKTNSSWCIHINNWLQSTLSAKHNNKSKTLDLPITDRIGVYCNFDGGQLMFYNANSMELLYAFKTKFTQPLLPGFMVWCGGLSVSTCLQVPSVVKAFQKSVNGLNSSTCSLYSVPVMPIESSHC

>duFSD2

MSARSGRVRQYESSGQLPDQSGSPPTADSSETEAEGLIFYHMDLYGSKERFDIFPEEPSGQADRSRGNSRKEPALSSEKIQHSQEAGYDLEKEVAELAKMYGLDEDKEKELELLGGHPQTVERRWPPAHTQKAGSQGSVYSASKSSSPVKDQSTKQQGLPDEASQDEDQSKARAEDDTESHTWSREGLSSGGMSDERSSQAVSEEEETADVFCSTCKMPIRAFDKLFGEHRDHEVAQLSNAVESEKEEIHKNMCKLEEQIAQIENFASHLEEIFITVEENFGRQEQNFEVHYNDAVQVLAQKYEEQLEALGEEKRQKLEALYGQLVSCGEHLDTCKELTDTTQELYLENDKANFMKAAVTMVDRLEEFLRKEVDLELSTLPDFEERKIDFSEVEQLMNSINTIPAPCAPVINPQAPNAATGTSLRVCWGLFSDDTVECYQLCYKPVSNERHGDEQAEHMLKVKETYCTITNLLPNTQYEFWVSALNASGISPPSERAVYVTAPSPPIIKSKKIRSCENAALVCWESRDINPVDSYMVELSKLTDEENDDTITESIVGIPNCEVLIHLQPIQSYRICVRALNLGGSSESSEPVLIHTTGTYFCLNEDTAHPLLAILDDGFTIACDELENPECDLPVYDNSFTRCIGILGSLIPFPGKHYWEVEAEEDTEYRIGVAFENTPRHGYLGANNSSWCMRHIITPSRHKYEFLHSGMTPDIRITIPPTRIGVLLDYENYRLSFFNADIAQHLYTFNSHFQHYVHPCFALETPGILQIRTGIAAPPWTALP

>duFSD1

MGEQEALRKIITTLAVKNEEIQNFIYSLKQMLQNVEDNTARVQEDLEGEFQSLYVLLDELKDGMVTKIKQERASRTYELQTQLAACAKALESSEELLETANQTLQTANNHDFIEAAKQIKDSVTMAPAFRLSLKAKVSDNMSHLMVDFAQERRLLQALAFLPVPSTPEIDLAESLVADNCVTLAWRMPDEDSKIDHYVLEYRRTNFEGPPRAKEDQPWMVVEGIKGTEYTLAGLKFDMKYMNFRVRACNKAVAGEFSEPVTLETRAFTFKLDASTCHQNLRVEELSVEWDATGGKVQDVKAREKDGKGRTASPANSPARVVQSPKRMSSGRGGRDRFTAESYTVLGDTLIDGDDHYWEVKYDRDSKAFGVGVAYRSLGKFDQLGKTSASWCLHLNNWLQVSFSAKHANKAKVLDVPVPDCIGVYCNFHEGFLSFYNARTKQLLHTFKARFTQPVLPAFMVWCGSFHVTVGLQVPSAVKCLQKRNSTASSSSSLP

>duTRIM200

MAQGNAVKNLREEATCAVCLDFFRRPVMLLPCGHNFCRSCVARCAQKSQDGAGSCPLCRLPFPPGGFCPNRQLANVVAAVRELVEGEDLCLGGDLERRVPPAEPLEDREPTPSSSRPQEHLGPPQNKRQEDERAATLLTPPGGAQQALMARVEAERQKLLAVLGGLRGLVGQQESWLLARLGHLRRGLEEAKGWQGGGNPWDVGVAPSRSTEQRPRPLELEAELEDFSQKNNALEAVVERLKDVLACSLEEDLGGYQKASVTLDPATAHPQILVSPDGRSARRRKTPRDPPPGSSERFEALRCVLGRQGFAAGRHRWAVEVSPGPDWALGVAREFVPRKGCFGLSPARGVWAVGQWLGQLRALTWPSPTCLHLARVPKRIEVALDYGGGRVAFRDADSEVEIFAFPPAAFAGERLRPLLWLGEGPALLTFCP

>duTRIM39

MSSSDPLETLRVEASCSVCLGYLRDPVLLECGHNFCRSCITRWWAELSRDFPCPVCRKTSRRRALRPNRQLANMVEAARQLRGPKRKASPENPEICCQIHGRALVRFCREDQAPACRLCGISSAHRAHQMVPLEEAAREYKEKLQNFLESLERRLQAVSSCRAREEKKPSELKRKVALRRERIAREFQELHQLLEEEERLLLGRLEEEEREILQRLQANLELLGEQRRALGALVAELEEKCLQPGAEMLKDIKDTLARCEAAAAGAEPTSVPSELEKNFGSFPRQYFALRKITRRLLGDVTLDPSTAHPNLLLSPDLKSVRFAPNPPRRLPHTPRRFTLYPCVLGSRGFTSGRHYWEVEVGDKTHWALGVCKDSVNRQGDPPNFPETGFWRVRLWNGENYAATTHPFTPLRLRVKPKRVGVFVDYEEGEVAFYNVTDRSHIFTFSGTFTEKIWPLFYPGLRAGKKNAAPLVIRSPTDWE

>duRNF135

MAVPAELGRLLADVELSCSCCLQYFTEPVRLASCSHSFCRSCIDTYCRGRRRAPCPLCREDFEPKDLRPNRELAALVSLVLGGGRGEGLGAWDQSTASGDGAGGGCSSAWRRPGEKEEQIRDISKQLEITKETINALRKDLSKTKEYTSQILSQITEDFCCMKEYIERQEENTLMFIEQEQRAARQKIVQTIHQLCVEKYKLIDIKAQMEKGLESDEMEWQTSNLLERGGGSPSTMHKFTIDEKFNVVRSAVGDLKRKLEILLLEEYPQQFPPAQSPDLHQETSVCSLSSESAAKSPEPSISSQFSRWADNVTFDLTTAYDRLAITAQNRKVMVSSNPTYYEPSLKRFCISQVLCSQGFSTGCHYWEVITKDSDGWAVGVARGTIGRRDRLGRTESSWCVEWVGPQKQLSAWHRNQETLLRNDKPLKVGVFLELQKTVSFYAITDREMLLHTFEINNSNPLYPAFWLYSLDKNGSLTINHINRK

>duBSPRY

MAQRAGGAPAGRAAELRNKLVDQCERLQLQSAAIAKHMAEVLPAKSQSVLTAANGARELVIQRLMFVGKVCENEEQRLLENVHTEEERVHQSILTQQEHWTEALQKLDALRTYLVDMITNKDDQGLVRAEKEIFERTEVAEGILEPQESVKLNFNQQCVQSPLLHRLWASAVLSCITGSQEILIDEKTVSPHLSLSEDKKTLTFSPKKAKLDLDCPDRFDHWPNALATAAFQTGLHAWKISVEKSCAYKLGVCYGSLPRKGSGNEVRLGFNTASWVFSRYDKEFRFLHAGQPQPVELIKSPAEIGVLVDFAGGEVLFYDPDSCAILFSHRETFAAPLYPVFAVAHHSISLVQ

>duNHLRC1

MAAAGEAGEAELGLLECRVCFEPYGPGGQRRPRNLPCGHVLCRGCLSALGGRGRLECPFCRRVCGPADTSDCLPLLQLLEVLGPACGILPAASSGAPALRLALGGWGALVNPTGVAACPKSGRLAVAHDGKKRIHVYGPSGSCLQRFGERGEAGCDVKYPLDVAVTADGHVVVSDGGDRAVKAFDWEGRGVLAVREGFCLPWGLDATPESDVILADAEAGVLYRLTADYGKGELKKCQVLRAKLSSPRAVAVCRSSGAVVVVEHLKARGPKGSSTRIKIFSADMDLVGQMDSFGLNLFFPSKIYTTAVAFDKEGRIVVTDVCSQAVICLGKPGEFPIFNPLISHGLSYPIGLTYTADNSLVVLDSGDHTVKIYSST

>duRNF207

MAGGIFSPLGSCSELEKGAWHPLVCLLCHEPFQHPCLLDCYHNFCASCLRGRASDGRLRCPLCGHPSVVRGGTGLPPVDRLLQFLVDSSADGEEDVQCANCDRRCAKADLDAMCFCNTCSQPLCAPCREETHRAKVFARHEIVSLSKRTKDIHKKCPLHEEPYIMFSTEKKSMLCINCFRDMQGESRAHCIDIETAYVQGCEKLDQAVLAVKELQTSTREAIVLLKAMIEEVRNSASQEETAINALFSGMQEQLSERKKALLKAVQSQHEEKEKAFREQLAHLASLLPTLQVHLVICSAFLSSANKAEFLDLGYQLMERLQRIVKLPHRLRPAQTSKINTEYRAEFARCLEPLLMLTPRRSVVGSAGGIGPGIAGTNMIPVGQSSKTLMVPGCPPSGDKMSTGSMVRKPTLHRYISTKVLLAEGRETPFAEHCRNYENTYRMLQTEIQGLKDQVQELHRDLTKHHSLIRTEIMSEILQKSLQMDVQIAAHYSSVEMMRSVFEEVWEETYQRVANEQEIYEAQLHDLLQLRQENSCLTTITKQIAPYVRSIAKVKERLEPRLQEPREPKDEHTQTLLRIEDSSEAAQRDGSPGSKESRERALGSRGGGRTPTFAPEDPLLKNEERCQSKQRSGTEGTTREDPTASS

>duRNF39R

MSPQCPPPVPQRPPHVPPPPPIPTPPSPLPLPAPPHPFRAPPQPQNPPPPPPPRPQTPQDPPPKTPKMSPSSPLPPPQRGPLALLASATRCGVCGGAFRDPVLLGCEHGCCRRCLPPGDPVNCPQCHRGWPRNRIRTPVALAVEVRIARRLAGVAGGGTGGGQEEDEEEEEEEERRKGMKRRRRKAQRWSLGAQLRADTAPPRPPPPPHEDPRDGGAPAAAPPSPSPG

>duTRIM1

METLESELTCPICLELFEDPLLLPCAHSLCFNCAHRILVSHCATNEPVESITAFQCPTCRYVISLNHRGLEGLKRNVTLQNIIDRFQKASLSGPNSPSESRRERTYRNSPTMSVAGERIACQFCEQDPPRDAVKTCITCEVSYCDRCLRATHPNKKPFTSHRLVEPVPDAHFRGLTCLEHENEKVNMYCVADDQLICALCKLVGRHRDHQVASLSDRFEKLKQTLETNLTNLVKRNSELENQMAKLIQICQQVEVNTAMHEAKLMEECDELMEIIRQRKQVIAVKIKETKVMKLRKLAQQVANCRQCLERSTVLINQAEHILKENDHARFLQTARNVAERVAMATASSQVLIPDINFNDAFENFALDFSREKKLLEGLDYLTAPNPPSVREELCTASHDTITVHWISEDEFSVSSYELQYTIFTGQANFISLYNSMDSWMIVPNIKQNHYTVHGLQSGTRYIFLVKAINQAGSRNSEPARLKTNSQPFKLDPKMAHKKLKISNDGLQMEKDESSLKKSHTPERFSGTGCYGAAGNVFIDSGCHYWEVVVGSSTWYAIGVAYKSAPKNEWIGKNSSSWVFSRCNNNFVVRHNNKEMLVEVHPQMKRLGVLLDYDNNALSFYDPANSLHLHTFEVSFILPVCPTFTIWNKSLMILSGLPAPDFIDYPEQQECNCRPQESPYVSGMKACH

>duTRIM2

MASEGSNIPSPVVRQIDKQFLICSICLDRYKNPKVLPCLHTFCERCLQNYIPAHSLTLSCPVCRQTSILPEKGVSALQNNFFITNLMDVLQRTPDNSIEESSILETVTAVAAGKPLSCPNHDGNVMEFYCQSCETAMCRECTEGEHAEHPTVPLKDVVEQHKASLQVQLDAVNKRLPEIDSALHFISEIIHQLTNQKASIVDDIHSTFDELQKTLNVRKSVLLMELEVNYGLKHKVLQTQLDTLLEGQESIKSCSNFTAQALNHGTETEVLLVKKQMSDKLNELAERDFPLQPRENDQLDFIVETEGLKKSIHNLGTILTTNAVASETVATGEGLRQTVIGQPMSVTITTKDKDGELCKSGNAYLTAELSTPDGSVADGEILDNKNGTYEFLYTVQKEGDFTLSLRLYDQHIKGSPFKLKVVRSADVSPTTEGVKRRVKSPGSGHVKQKAVKRPASMYSTGKRKENPIEDDLIFRVGTKGRNKGEFTNLQGVAASTNGKILIADSNNQCVQIFSNDGQFKSRFGIRGRSPGQLQRPTGVAVHPSGDIIIADYDNKWVSIFSSDGKFKAKIGSGKLMGPKGVSVDRNGHIIVVDNKACCVFIFQPNGKIVTRFGSRGNGDKQFAGTLDGNM

>duTRIM3

MAKREASASPVVRQIDKQFLVCSICLDRYRNPKVLPCLHTFCERCLQNYIPPQSLTLSCPVCRQTSILPERGVAALQNNFFITNLMEVLQRDPDSRGPHPGQGLDPVSAVTGQPLSCPNHEGKVMEFYCESCETAMCHECTEGEHREHVTVPLRDVVEQHKAALQQQLEAIRGRLPQLAAAIGLVSEISQQLVERKNEAVSEIGSTFEELEKALQQRKGLLVRDLEAICGAKQKVLQAQLDALRQGQENILSSCAFTEQALHHGTAPEVLLVKKQMSERLSELASQEFPEHPHENDQLDYVVETDGVRKSILNLGVLITTSATAHKTVATGEGLRHAVVGQPASLSVTTKDKDGELVRSGSASLRFQVTGPDGSVAESEVLDNKNGTYELLYTPRAEGDFMLSILLYGQPIRGSPFRVRAVKACDVPPSPDDVKRRVKSPSSGHIRQKAVRRPSSMYSSGKKKENPIEDELIFRVGSRGREKGEFTNLQGISTSSAGRIVVADSNNQCVQVFSNEGQFRLRFGVRGRSPGQLQRPTGVTVDMNGDIIIADYDNRWVSIFSPEGKFKVRGGPGRGAVIRGPL

>duTRIM7

MGLGWFPPSQWPAVVPRCLPLCPWVLGPLGLPAKTLPMQKAGRFGRTPLSYTSAARREAMAAMFLPGNLQDEATCSVCLEFFKDPVSIECGHNFCRACIIKSWKDLEMDFPCPQCREVFQQKSFRPNRQLANMAEIISQFALRGAKGAEEDGLCAKHREALKLYCKDDRRTICVVCDRSREHRPHAVVPVDEASEEYKEKIQGRLDFLKKERQELLEFKVNDDKKTQELLKTIENERQKLLVEFEGLRQFLHDQEHILLGQLEKMEKSIAKRQNENISDLSKEITLLNKLITELEEKIQQPMLEFLKDVMSIISRSDDVKSHKPVPVCTDMKMHVCNFSLKTVVLEKVVKKFKENLRDELGRGEKEDLTLDPESANHLLILSADLKSVRMGCRKQELPDNPKRFDTNSRVLASTGFKSGRHYWEVEVGASDGWAFGVARESVRRKGLTQFSPEEGIWAVQQNGGRYWAVTSPQRTPLCLNQKLNKVRVYLDYEGEEVSFYNADNMQHIFTFNVAFQEKVFPLFSVCSTVTYIKLCP

>duTRIM201

MRTLPPARSPRAPRMRGPAPSPHACAAPPPPAASRGGGAEGCGARERCAARGGDLGGGRGKKGEGSDMEAPPAPPAVPPAPCSAARSLQDELTCPVCLEYFTDPVLVAECGHNFCRACVTQCWEDSARRLCCPQCREPVPQRLFRPNRSLGNIVHIVRQLGLPPGPAEPPPGPPAPSPPLPAAAPPGPPGPPRCPRHGEPLRLFCVQDRRAVCVVCHLSREHRTHTVLPAEEAAHAAEISSSFQEVPQEHLSSLRKGREEAKAERERQSEDLLKQTEVERQKIVAECKELRGFLEEKEQLLLSRLEELDRDIVKRRDESVSRLSEEIAQLDKLLGEQGGENGPGNQSGQVVTTAGSSFESWMFCKPEAGFAELEKKLKSFSQKSAVLKEVLLEFKENLRFELENDTGDLSLDPDTANPYLVLSEDKRSVRLRSAPQELPANPKRFDYSFCVLAAEGFVAGRHYWEVEVGDGESWVLGAARESVRRKEKIDFAPEEGIWAVGLNWKGKNWDQYQAFTSPETPLSLCERPRKIGVYLDYEGGWVAFYNADNMAPIFTFTAAFTEKIFPFFWLFYVGSSLSLCN

>duTRIM8

MAENWKNCFEEELICPICLHVFVEPVQLPCKHNFCRGCIGEAWAKESGLVRCPECNQAYNQKPNLEKNLKLTNIVEKFNSLNLEKPPSVLHCVFCRRGPPLPAQKICLRCEAPCCQSHVQTHLQQPSTARGHLLVEADDVRAWSCPQHNAYRLYHCEAEQVAVCQFCCYYSGAHQGHSVCDVEIRRNEIRKMLMKQQDRLEEREQDIEEQLYKLESDKRLVEEKVSQLKDEVRLQYEKMHQILDEDLRKTMEILDKAQAKFCNENAAQVLHLNERMQEAKKLLSSVQVMFDKTEDINFMKNTKSVKILMDRTQNCTGGSLPPPKIGHLNSKLFLNEIAKKEKQLRKLLEGPLSTPVPFLQSIPMYPCTVSNSGAEKRKHSTAFPEGSFLEPSSGTVASQYMSQGASAGEGQSAQAMVPCSSTQHIVGLPSGPQPVHSGSVFNPSHYPNTTSSQQSVLSQYGGRKILVCSVDNCYCSSVSNHGGHQPYPRSGHFPWTVSSQEYSHPLPPAPAVPQSLPGLAVREWIDASQQHGRQDFYRVYGQPSAKHYVTS

>duTRIM9

MEEMEEELKCPVCGSFYREPIILPCSHNLCQACARNILVQTPDSESPQSRRASGSAVSDYDYLDLDKMSLYSEADSGYGSYGGFASAPTTPCQKSPNGVRVFPPAAPPPPAALAPPPPPPRNACLTCPQCHRSLVLDERGLRGFPRNRLLEGVIDRYQQGRAAALRCQLCEKAPKEAAVMCEQCDVFYCEPCRLRCHPPRGPLAKHRLVPPAQGRVSRRLSPRKISTCTDHELENHSMYCVQCKSPVCYQCLEEGKHSSHEVKALGAMWKLHKSQLSQALNGLSDRAKEAKEFLVQLRNMVQQIQENSVEFEACLVAQCDALIDALNRRKAQLLSRVNKEHEHKLKVVRDQISHCTVKLRQTTGLMEYCLEVIKENDPSGFLQISDALIRRVHLTEDQWGKGTLTPRMTTDFDLNLDNGPLLQSIHQLDFVQMKVSSPVPAPPILQLEECCTHNNSATLSWKQPPLSTVQVEGYILELDDGNGGQFREVYVGKETMCTVDGLHFNSTYSARVKAFNKTGVSPYSKTLVLQTSEVAWFSFDPASAHADIIFSNDNLTVTCNSYDDRVVLGKTGFSKGLHYWELSIDRYDNHPDPAFGVARIDVLKDAMLGKDDKAWAMYVDNNRSWFMHNNSHTNRTEGGITKGATVGVLLDLTRRTLTFSINEDQQGPVAFENLEGLFFPAVSLNRNVQVTLHTGLPVPEFYASRSAMQ

>duTRIM207

MMESGKRNADLEEWDAKIRKLTEEFEESDTELEECDTENEIGELTAEIDNLTAELEERDRKIRKLTSDLGECDTKIKERDRKITKLSAEIRRLTALLGDRDKQLRKLTAELAKCDATIRLFTVELGERHTKIGELTAEVGDYDRQLRKHAAELVERDEKIREHEAEIRRLTELLENRDLEARNQDALIRKLTEELEELKGLEADESSEDLEESDLERDELTEELDEITEELGERDKKIEELTEELAKQTAQIDELTTELGERDAKIDELSAEMEKRDRKIDELTAELEEYKAKMRKCMEEHRKEEEKLANVTLDPETAHPRLILSKDQKSVRWEYMLQESPDSPERFDADPCVLGCEAFTSGRHYWVVDLAEGQYCAVGVSRESLPRKGPVSFNPEEGIWAVQQWGFKNRALTSPPTLLNLPRVPKKIRISLDYEWGEVAFFDVENKVPIFTFPPASFAGERIRPWFWVELGSLSLVR

>duTRIM208_NCBI_GeneID:110354628_ProteinID:XP_038043779.1

MASAASRARERGEEGAHHIAPKHRPDPEHLSCGGGTAKSCPERGRLGDAEAISHRNHLRKGNFQPKLHLEHLAEKLKLLGLEGGGEEEQLCSWHKRTFAFRRDAKASGCGAPRACGEAHREEPAQEDREQIHRDLENLKKQREELLELKASGERRCQGYLTQTEAERQKIVSEFRQLRRFLKDQEVVLLAQLGELDREVMRRQEEEEAKVSGEISLLDILIWEAERKLEQPTSGFLQSARSTMGRWETSSTRRMMETFSDLERRLRVISQQNDILREALGRFQDILPSELEKEVEPSLGGEGKAFITLDPDTAHASLIVSRDRRGVRWMDAGQDLPPNPRRFDVFCCVLGHRGFTTGRHSWEVEVVAGGTWALGVARNSLSRRGCLEFRPEEGIWAVGRCGNRYRAFASPPTTLPTSGNPRRIRVVLDYEGEQVAFYFAEQLPPVFTFQKACFGGESIFPFFWVGRGSHLRLCP

>duTRIM213

MGGVPKPGGGPEVTGVSRRHGGEVPQPSGSRSCEAPLPPRAPLPVPKAKELQSQAGSRTLGPSAMAGSSEAAAELQEEATCAICLDLFRSPVMLDCGHNFCQACIGLCWARSAGAPSCPQCRQALPSRSLRPNRQLGNIAARLRRLGQTVEPRQEQNQSELESLRRERGELEEQLKKERCTCQGYLDKVKAERQKIESEFKQLHQCLEEQECLLMARLGELERQIETRVKEKADKLSKRIFHLDGLIREKEESQLSGCEFPQDTGDILGRCEKGKFQQKEWTSQNLEKAAGVCSKKPPELEETARKLQDASTTALREEGEESQGLYTKVNVTLDPDTAQSRLILSEDGKSVMQGATQQHRPDSTKRFDPWPCVLGCEGFDSGRPCWEVEVGSGSCWAVGVALESVRRKGPIDMNPVGGIWAVGQYKEKFQALTSPTPTPFLPSMVPRRVRVCLNHAEGRVTFVNADNEATIFTFLQATFSGKRIYPWFWVGKGSQLKL

>duTRIM13

MELLEEDLTCPICCSLFDDPRVLPCSHNFCRKCLEGILEGNVRNVLWRPAPFKCPTCRKETPVTGVTSLQVNYSLKGIVEKYNKIKVAPKMPVCKVHSGQPLNIFCRTDMQLICGVCATRGDHTKHVFCSIEEAYSQEKRAFETLFQGFETWRCGDALSRLDTLETSKRKALQMLTKDSDKVKEFFEKLQHTLEQKRNEILSDFETMKLAVMQAYDPEINKLNAILQEQRMAFNIAEAFKDVSEPIIFLQQMQEFREKIKVLKETPLPCSNVDVSPTMKSFDTSQWNGIKLVDVDKLALPQENSTLKLKIPSVFSRRLIVTTLICLLLLAVTRMSFVESVVDNLQGWKSQLFTISLSYLADTVEIADHAVFYWEQMTDGASLLSEKCKNYTLVVLDNVAQFVCKYKLL

>duTRIM14

MALGEPRRGCGAHAGRPLELLCEDCGRCVCALCPALGPHRGHRACLLHHAARHRQELLTLCLKDLEERKEQEAGNRRCIEQAANDLKAHAAMTKKQLSDRMTELQLLLREEESLARNLIDEKTQQALEAHGQQMESCQEKLAALDTFSYRIREMQQNNDIIQFLEKSIEIEKELQESKSQLEQCHPIPLSFEHVLNYYKHLMTGLQSVLQKPLEVRLKEDVFSSLNATTKKEPGTMLKTMSPVDRLLFLKHARSPTWEYDSLHPRLKLSDDRLVVSCNWRRIFYPCGPQRFDKLWQVLSRDGFLSGSHYWEVDLLQAGTGWWIGAAYPSIGRKGDSETCRLGWNRASWCIKRFDFEYWAFHKGERIPLTVEDDPDRVGVFLDYEAGILSFYNVTDGMAHLHTFRCKFTEPVYPALRLWEGSIGTCKLT

>duTRIM18

METLESELTCPICLELFEDPLLLPCAHSLCFNCAHRILVSHCATNEPVESITAFQCPTCRYVITLSQRGLEGLKRNVTLQNIIDRFQKASVSGPNSPSETRRERAFDSNSMSSCEKVLCQFCDQDPAQEAVKTCVTCEVSYCEECLKATHPNKKPFTGHRLIEPIPDSHIXGLMCLEHEDEKVNMYCVTDDQLICALCKLVGRHRDHQVAALSERYDKLKQNLESNLTNLIKRNTELETLLAKLIQTCQHVEVNASRQETKLMEECDQLIEIIQQRRQIIGTKIKEGKVVRLRKLAQQIANCKQCIERSTSLISQAEQSLKENDHARFLQTAKNITERVSMATASSQVLIPEINLNDTFDTFALDFTREKKLLECLDYLTAPNPPTIREELCTASYDTITVHWTSDDEFSVVSYELQYTIFTGQANVVSLCNSADSWMIVPNIKQNHYTVHGLQSGTKYIFIVKAINQAGSRNSEPGKLKTNSQPFKLDPKSAHRKLKVSHDNLTVERDETSSKKSHTPERFTSQGSYGVAGNVFIDSGRHYWEVVISGSTWYAIGISYKSAPKHEWIGKNSASWVLCRCNNTWVVRHNSKEIPIEPAPHLRRVGILLDYDNGSLAFYDALNSLHLYTFDITFGQPVCPTFTVWNKCLTIITGLPIPDHLDSSEQLA

>duTRIM19.1_NCBI_GeneID:113844857_ProteinID:XP_038040928.1

MPGSTEPPRPSGTPDAGPAASTEPSVPMEPAPPRPQEQEEEEDFQFVLCEGCRQESPSLKLLTCLHSLCLGCLSEKKPVGQCPVCQEPIPQPNGIPEVDNVLFASLQARLRVYRRIVSGALSCSRCRREPAAVWCSECEEFLCPGCFEDHQWFFKKRSHEARKVEELRAESAHRFLEGTKKSCTLFCSSHGHTEQGHITSIYCKKCEKALCCSCALLDAQHSPFYCDIRAEIQRRQEELAAAGRELARRRGGFEASRAALQEEAARLEAASGETRELIRQRVEQLVRLVRREEAELLGLVERRREQGRRELAGELRRVEGVLRRMEAGERLVEKMRLYATEQEVMDMQPFVREALRELQRLRPPVAGGRAQHGDFAECRARLQALAERVEAHAGTSSQAVPVVEVALENDQQEEPTQRGSPGIVPTFTISLGDMQLPTATVRCKRWRPQVERGSQASPKVLKLEHNTTADPSEPSSTQRDSRGEPSTSATSHNCSSVPKAGRSHADDAEDNSIIISSEDSEEDTVVSVTPDLPPC

>duTRIM19.2_NCBI_GeneID:101796105_ProteinID:XP_038040922.1

MAGKYPLFGGHPLFGGRSLLVVVPSLVVSPPWWQSHSCQRLLPAHCWVLDGMDGKQGTAPPRTKRAMEMLCRAPRPLQAPCPPGKAPLVAEPFLPAISRGLCAAGLPTSKVEPGSSAGEIESGLKEQKEGAEGAEANGRLGGELGVPHSTMASPDQQQQQQQQQQEEEEDFQFVLCEGCRQESPSLKLLTCLHSLCLGCLSEKKPVGQCPVCQEPIPQPNGIPEVDNVLFASLQARLRVYRRIAGGAELLCDNCRREGEYWCSECEEFLCTTCFEAHQRYLKRESHEARKVTDIRAGALKDFLQGTRRTGSLACSNPTHKNQTLSIYCKKCEKPVCCICALLDTQHAGQHCDIGAEIQRRQEELAAAGRELARRRGGFEASRAALQEEAARLEAASGETRELIRQRVEQLVRLVRREEAELLGLVERRREQGRRELAGELRRVEGVLRRMEAGERLVEKMRLYATEQEVMDMQPFVREALRELQRLRPPAAGGRAQHGDFAECRARLQALAERVEGHAEAAPAPATEDSHQAPSTSTPAKRKTDKDTNTLPSPVKVMKVEEDDDGWNMLAEPQRLSCEEQPGTSFLRLAMDDNLLEGMLDGNGGLCGSDSNNPSLESAEEDSVDEDSKDSSLLEGLGNMLDDGTSEEHLGFPIRLQNTMDTRQGSLVFFDVKILKNEIIQMAVIDGEQILPVLIQPVKCLPSLMAKNSVCEVGLRSLLGHLYAVHQPILGGFRFCSLPLPTLLEALTVLGKREEFSAAVYGFLDILPLIKEKVPERDNYRLKNLASSYLWRDLSDHSAMESARAVKDLCEVLDIDLLRTPRLVLSHASLECWVSLQPLLEEKLLNKASAQRLASCNVGLSELWSCHRHDPGQGLQKLRALLNAHRHGSEKKIRTLSKVQLYFQRQQEDSREAPAGSNVPKDVKNKEN

>duTRIM23

MAALAVNRAGAAQDGGRASSTAGSSSRGSAGAAVKVLECGVCEDVFSLQGDKVPRLLLCGHTVCHDCLTRLPLHGRAVRCPFDRQVTELGDSGVWGLKKNFALLELLERLQNGPAGQCGTAEEAIGLSGESIIRCDEDEAHVASVYCTVCATHLCADCSQLTHSTKTLAKHKRVPLADKPHEKTMCSQHQVHAIEFVCLEEGCQASPLMCCVCKEYGKHQGHKHSVLEPEANQIRASILDMAHCIRTFTEEISDYSRKLVGIVQHIEGGEQIVEDGVGMAHTEHVPGTAENARSCVRAYFSDLHETLCRQEEMALSVVDAHVREKLIWLRQQQEDMTILLSQVSTACLHCEKTLQQDDCRVVLAKQEITRLLETLQKQQQQFTELADHVQLDASIPVTFTKDNRVHIGPKMEIRVVTLGLDGAGKTTILFKLKQDEFMQPIPTIGFNVETVEYKNLKFTIWDVGGKHKLRPLWKHYYLNTQAVVFVIDSSHRDRVSEAHSELAKLLTEKELRDALLLIFANKQDVAGALSVEEITELLSLHKLCCGRSWYIQGCDARSGTGLFEGLDWLSRQLVAAGVLDVA

>duTRIM24

MEEAAAAVAAAAAAATAAAAGGALPCPVDRGGGAAALSGENEAESRQGPAERGGEAAPLNLLDTCGVCGQPIQSRRPKLLPCLHSVCLRCLPPPDRYLMLPPAGPPVPTAAPHKEPQPPAPPSPPGSSPLHCTPVGVIRCPICGQECAERHIIDNFFVKDTTEVPSSTVEKSNQVCTSCEDNAEANGFCVECVEWLCKTCIRAHQRVKFTKDHTVRQKEEVSPEAVGVTSQRPVFCPYHKKEQLKLYCETCDKLTCRDCQLLEHKEHRYQFIEEAFQNQKVIIETLITKLMEKTKYIKYTGKQIQNRILEVNQNQKQVEQDIKVAIFTLMVEINKKGKALLHQLETLAKEHRMKLLQQQQEVAGLSKQLEHVMNFSKWAVSSGSSTALLYSKRLITYRLRYLLRARCDASPVTNNTIQFHCDPSFWAQNIFNLGSLVIEDKETPPHMPKSPVMETNLQPAGNLPSNQLSKFPTQINLAQLRLQHMQQQVMAQRQQAQRRAGPVGLPNPRMPGAMQQPPASHQAPPRLIHFQNHNPKSNGSAPPAQQMRFPPSQNLPRQAIKLNPLQMAFLAQQAIKQWQVGNGQTSTATSAASNITSTPSSPTVTSAAGCDGKTYGAPVIDLSSPVGSSYNLPSLPDIDCSGNITLDTVARKDGTAEQSQAKPPSNRTVQSPNSSVPSPGLSGGVSVTNIHPPIRSPSASSVGSRESSGSSSRPLGADSTHKVPVVMLEPIRIKQESSAPNENFDFPIVIVKQETEEESRPRNTTFSRSILTSLLLDGNHNSTSDEAVIRTDAPDSTDDQPGILLENTTTGKSGWIGPSHTGEGRKEDDPNEDWCAVCQNGGELLCCEKCPKVFHLSCHVPSLMNFPSGEWICTFCRDLSKPEVEYDCDKPAHSPEKRKLEDTVGLAPIDRRKCERLLLYLYCHEMSLAFQDPVPPTVPDYYKIIKKPMDLSTIKKRLQVTSSFYTKPEDIVADFRLIFQNCAEFNEPDSEVADAGMKLEAYFEELLTSLYPERKFPVQPNCQSERENTELSDDSDDDFVQPRKKRLKGEDRQLLK

>duTRIM25

MAALTRAVSEPNLAGLEEDLTCSICLSLFDTPVTVPCGHNFCASCLDLTWAELDAGFSCPQCRTTFPGRPQLRKNTVLCRVVEQLQGCTAAEEQQKQEDEEDEAVAEEAASPVYCDSCLQAHAAQTCLTCMASFCAEHLQPHHDSPAFRDHQLCPPVRDLQQRKCSQHNKVFEFFCKQHGTCICSLCLLSHKLCNASPLQQAKAEAESALKKKLTELHNHSEKATRAMNSVKTSQTQTAETAARKRDLMRNEFLEIKALIEEKENQIFKVIMEEEKRVCTKFDYIYTVLGSKKNEIQSLRDQIEMALTEHDDVLFLKRAAALQRASTKEVFVPVIEMDQNLIHTAYQSAINLKEMVKLTVSQPKEKKTEGCYWPSWPQGHSAGLWSSGCLPGTPGPFPLRWSPTEKQTARKAKPPQAAALNRPVPGRKPVGPQRPNKEKKPSQVQEPLQEEADNRAPNMGAPSTAATTAGASKAATAANTKDLISSFLQKDREELLQYAANITLDFNTAHNKVHLSERYTKMSVSDTPLNYNHHPQRFTYCSQVLGFQCFKRGIHYWEVELQQKNFCAIGICYGSMDREGPDSRLGRNSSSWCIEWFNSKISAWHNDVEKNLPNVKATKIGVLLHCEGGFVIFLAVGEKLNLIYKFKTQFTEALYPAFWVFSSGTVLSLCQMKK

>duTRIM206

MAECDPLESLQKEASCSICLDYFSDPVSINCGHSFCRDCITRCSGKSDRRFACPQCRGIAQKRKFRPNRELRNLAEIAKKLISRVGDAARAGGVCPKHQEPLKLFCQEDQTAICVVCDRSQAHRAHTVAPIEEAAQECKEHIQSKLKSLKDERERLQGLKVMGEKRSQKHLQQARAERWKIMSVFKQLHQFQDEQERLLLMWLEDTEKEIVQTQSENDRRISAEISHLGNLIRELEGMNPQPENKSLQDARSALTRCDTRAFQHLSEKFPRVEKSLKDLSQKNIILKEALRKFKESLPVELDVQWANVTLDPDTANPHLVLSEDRRSVRWDETPQNLPDNPQRFDTYCSVLGHEGFTAGRHYWEVQLGNRGFWAVGVARDSAWRKGWISLDPSQGIWAVGICGDRFQAFTSFETVQPLNGRPRTIRVSLDYDKGQVAFFDADNETLAFAFTPTSFNGEKILPFFWVWESKIQLAP

>duTRIM205

MASQSPSESLQGEASCSICLGFFQDPVSIHCGHNFCRECITRCWEGLEANFSCPQCRQTASHKSFRPSRELAKIAEIAQQLSLQAGRGAAGHEGWCQQHQEALKLFCKEDQQPICMVCDRSQAHRLHTVLPAEEAAQEYKEEIQARLELLKEEREKYLESRKSRARKNLHLEKTKNEGKKIVCEFEQLHQFLKDQERLLLTQLADLDRAITRVQEEAVVKVSEEMAHLDTLIWEMEGKFQQPASKFLLDVRRLLKSCEVMKFNPPVEISPHLERRLEDFLQKNVLVRCTLRKCQDSLMFKLQEPTNVTLDPATAHPNLHLSEDRKQVRGQLVPQDLPDNPERFDFEPCVLGCQGFTSGRHFWEVEVGQGGVWAIGVARETAKRKGPMSLTPKEGIWALEAYHSLTSPRANVRLNQLPRKIRVSLDYEGGRVAFFSSDDDAPILVYSRAAFNGERVLPWFKMGMGARLQEITQNSSSEEQSMTGQLMSPLDWVGFRSPLRICP

>duTRIM203_NCBI_GeneID:101791534_ProteinID:XP_012953800.3

MAAPSPVPKLPSEASCPICLEYFRDPVSIHCGHNFCRACITRCWEWSTANFSCPQCKETAPERSFRPSRELARVLEIAKRLSLQAARGDVVEEEGCERHREPLNIFCKDDETFICVICRESRLHRAHTMLPVQEAVQEYKGQIQAHLQTLKEDRDRLLGFREAEMRRNWEYLEKTEAERQKVLSTFEGLRLFLEDQARHLLAQLAELERAIEKIQEENITNLTKEISHLDTLIQELEEKCQEPASKFLQDIRGTLSRFGKENFQQPTLLLPELENKISHFREKNIALEETLRNFKDILMFELPEKMNVTLDPATAHPQLAVSEDRRSVKWEDAQQDASDEGFGPDPSVLGCEGITSGRCCWEVEVTPKGSWAVGVARESLKRREETPVSPEIELWSMGLCEDQFWALTSFERTPLSQIQVPRRVRITLDYERGQVAFFDVDRKALIFIFSAASFKGESVHPWFLVWSEGSRITLCP

>duTRIM28

MSSPAKRPDAAAANGGPSEGAVVNCPVCKQQCYLQDVVENYFLKDNRPETAPDSQASTQCCTSCEDNAPATSFCVECSEPLCETCVEAHQRVKYTKDHTVRAAGNARAKEGEHAVYCAVHKSEPLVLFCQTCDVLTCRDCQLNAHKDHQYQFLEDAVRNQRKMLATLVKRLGDKHASLQRSTKEVRSSIRQVTDVQKRVQVDVKMAILQIMKELNKRGKVLVSDAQKVTEGQQEKLERQHWAMTKLQRHQEHVLRFTSWALESDNSTALLLSKKLIYFQLHRALKMIVDPVEPQGDMKFKWDLNAWTKSAESFGTIVSERSLPPPPLSPQPPAASPTAGPSQGSPHTTVVSEGQYAPSPLLQPPEGPQIGDKDGGGPPGDPQEGGGGLGTPQLPSGTAELGCPNLSPPHLDPQVTETPRDAGAELGNSSPEAAVTGTKRRKQRGSPPGEEKFVKKLLVKRSHPPGALGSPLLRKVPRVSLERLDLDLAGAAQPPSFRVFPGTTAEEFNLIIIERGGAQPWTPSSSRGGRNPGPPPCGDLGIYLWTSAPRGAAPHGIHSMPAPSRERAGRGGIHPRPLPGFPEIPGIRSCPPRPYRNVSCCRVCCQAGAVVMCDLCERCYHLDCHLPALHEVPGHDWLCLLCQDPAPPGEDPGPTEEQPPALSPTDQRKCEYVLLQLLCHEPWRPLHRLSSSLEGCDAIDLTLIRAKLQGKLSPGYGHPEEFARDVWRMIRQFNRLTEDKADVQSILGLQRFFEERLSAAFGDRKFCAALESLGPLDGAEVSQAPPPTLLAP

>duTRIM29

METGSAARTNGTAGKPEDVKSPSAPKKDEEVKKNSNPGGGEKEPMKGTGGTSLETGQIKSSLFSGSDWKRPIIQFVESSDEKRSTYFSMDSADSKKMQYSSGQIGDMRRPPXSFADKGDLRKSLFSLDSKKSFLPNEGEGRKPLFSGGQMGDMKKSSLPLVETGDLRRATFNKVPDRAAGSRPRVKLEDVLCDSCIDNKQKAVKSCLVCQASFCELHLKPHLEGAAFRDHQLLDPIRDFEARKCPVHGKTMELFCQTDQMCICYLCMFQEHKNHSTVTVEIEKAGKEAELSLQKEQLQLKIIEVEDEMDKWQKERDRIKNYTTNEKATVDQHFKELIRDLERQRDEVKAALDQREKIASENVKEIVDELEERAKLLREDKENREQIHQISDSVLFLQEFGALMRNYVPPPSLPTYSVLLEGESMSPSMGLLRDDLLNVCMRHVEKICKADLGRNFIERNHMENGDHRFMMNNYEWNQPDNLKRFSMFLSPKASFNPRSWEFSSFQATEETLGNGTKLPFQFSSVGQNPPGDFSKQSDGSLFTKTAYPSIVRHQSAKVTPQTWKSSKQSVLSHYRPFYVNKGNGATSNEAP

>duTRIM32

MATTALKNRTKTIGALWNKCEYRAWHRLKAASKAMASAPHLNSDALREVLECPICMESFTEEHLRPKLLHCGHTICKQCLEKLLANSINGIRCPFCSKITRITNLAQLTDNLTVLKIIDTAGLGEVVGLLMCKVCGRRLPRHFCKSCGLVLCEPCKEASHVPQGHSVIAIKEAAEERRREFGTRLARLRELMDDLQKRKASLEGVSRDLQSRYKAVLQDYSKEERKIQEELARSRKFFTTSLSEVEKVNNQVMEEQAYLLNLAEVQILSRCDYFLAKIKQGDIALLEEAADEEEPELTNSLPRELTLQEVELLKVSHVGPLQIGQVVKKPRTVNVEESLMETASSSSSSVSFREPELQEEASCTPHASPAKPRMPEAAASIQQCHFIKRMGSKGSLPGMFNLPVSLHVTQQGEVLVADRGNYRIQVFTRKGFLKEIRRSPSGIDSFVLSFLGADLPNLTPLSVTMNCHGLIGVTDSYDNSVKVYTMDGHCVACHRSQLSKPWGIAALPSGQFVVTDVEGGKLWCFTVDRGVGVVKYSCLCSAVRPKFVTCDAEGTIYFTQGLGLNLENRQYEHHLEGGFSIGSVGPDGQLGRQISHFFSENEDFRCIAGMCVDARGDLIVADSSRKEILHFPKGGGYNILIREGLTCPVGIAITPKGQLLVLDCWDHCIKIYSYHLRRYSTP

>duTRIM33

MAENKGGGGGGDGAAEAGPGGGGGPEPVAASPSGAAPPAPAAAPPEERDSPGAAAAAAAERALGEAEAEAAAGPGAVPGPGPSPVPPLTPAAPGPFSLLDTCAVCAQSLQSRREAEPKLLPCLHSFCRRCLPEPERQLSVPAPGGANGDIQQVGVIRCPICRQECRQIDLVDNYFVKDTSETPSSSDEKSEQVCTSCEDNASAVGFCVECGEWLCKTCIEAHQRVKFTKDHMIRKKEDVSSEAVGASGQRPVFCPVHKQEQLKLFCETCDRLTCRDCQLLEHKEHRYQFLEEAFQNQKGAIENLLAKLLEKKNYVNFAAAQVQNRIKEVNETNKRVEQEIKVAIFTLINEINKKGKSLLQHLENVTKERQMKLIQQQNDITGLSRQVKHVMNFTNWAIASGSSTALLYSKRLITFQLRHILKARCDPVPAANGAIRFHCDPTFWAKNVVNLGNLVIENKPTPSYTPNVVVGQAPPGTNHVNKTPGQINLAQLRLQHMQQQVYAQKHQQLQQMRMGQPSGSVPRQTGPQILQQQPPRLISMQTMQRGNMNCGAFQAHQMRMAQNAARIPGIPRHNGPQYSMMQPHLQRQHSNPGHAGPFPVVSVHNNTINPTSPTTATMATANRGPTSPSVTAIELIPSVTNPENLPSLPDIPPIQLEDAGSSSLDNLLSRYITGSHLPPQPTSTMNPSPGPSALSPGSSGLSNSHTPVRPPSTSSTGSRGSCGSSGRTAEKTSVNFKSDQVKVKQEPGTEEEICSFSGTVKQEKTEDGRRSACMLSSPESSLTPPLSTNLHLESELEALGSFENHVKTEPGDLSESCKQSGHSLLNGKSPVRSLMHRSARIGGEGNNKDDDPNEDWCAVCQNGGDLLCCEKCPKVFHLTCHVPTLLSFPSGEWICTFCRDLSKPEVEYDCDNSQHSKKGKTAQGLSPVDQRKCERLLLYLYCHELSIEFQEPVPASIPNYYKIIKKPMDLSTVKKKLQKKHSQHYQTPEDFVADVRLIFKNCERFNEADSEVAQAGKAVALYFEDKLTEIYPDRTFQPLPEFEQEEDDGEITEDSDEDFIQPRRKRLKSDERPVHIK

>duTRIM35

MLPAPARPPHRSARRRGRCKRCRSPSVSAMEKATSPPPGSSVILASSSASTLKEELLCPICYEPFREAVTLCCGHNFCKGCVSRSWEHQYHLCPVCKEPASPDDLHVNHTLSNLVEMILKEEGQRRGRPAALCTVHHEEAKLFCLDDKELACFSCQSSKQHEGHKMRPVQETAADFRAREGPEGVSPPQAKLKNMETSLRDKVKDFGTVHRSYESISKHNQVEAVRLEEQIKKEFEKLHEFLRDEEKALLAQLQEETRRKQDLVEGKIKQLSDESRALLNEACQLQDDLKEDDYTFLMTHKNRKRRIACTVEEPEAVASGMLIDTAKYLGSLQYNVWKKMLDNITVVPFSFDPNSAAGWLSVSEDLSSVTNGGYKLLTENPERFTSAPCILGSRGFSAGFHSWEVDLGGITNWRVGVARPRGRTPWNFHHDARSGFWYLYRLPTDGETCRASNAARSEATLGELGRLRVELDCDEGELSFYDADRRSHIYTFHEKFGGTVFPYFYMGATPVGALPRALRICPVRVRIHEDTPV

>duTRIM36

MEGDGLEPEPEPEVATKSIERELICPACKELFTHPLILPCQHNVCHKCVKEILFTLEDSFADGGSESSNQSSPRIRISSPSMDRIDRISRSGRKRNSLTPRTTLFPCPGCQHDIDLGERGINGLFRNLTLETIVERHRQAARAAIAIMCDFCKPPPQESTKSCMDCSASYCNECFKVHHPWGTLKAQHEYVGPTTNFRPKILMCPEHEMERVNMYCEICRRPVCHLCKLGGSHANHRVTTMSTAYKTLKEKLSKDIEYLISKESQVKAHISQLDLLLKETECNGERAKEEASQSFEKLIHVLEEKKSAALRAIEASKNLRLDKLRTQAEEYQGLLENNGLVGYAQEVLKETDQSCFVQTAKQLHVRIQKATESLKSFRPAAESSFEDFVVDTAKQEQILSDLSFYSNGLEIPEINEEQSRIYNKAVISWESPGKTDAADIYVLEYRKFNREEENVMWQEIEVCSKSKVISDLDDDSSYAFRVRGYKGSICSPWSKEVILHTPPAPVFSFLFDDKCGYNSERLLLNPRRTAVESRAGFPLLLGAERIQVGCYTTLDYIIGDTGIAKGKHFWAFRVEAYSYLVKVGVVSSAKIQKLFHNTHDVTSPRYEQDSGHDSGSEDACFDSSQPFTLVTLGMKKFFIPKAAADPKDAASRVLPLPSCLGICLDCDSGKVGFYDAGRMKCLYECEVDCSGIMYPAFALMGSAAIHLEEAIATKYLEYQDDL

>duTRIM37

MDEQSVESIAEVFRCFICMEKLRDARLCPHCSKLCCFSCIRRWLTEQRAQCPHCRAPLQLRELVNCRWAEEVTQQLDTLQLCNLTKHEENEKDKCENHHEKLSVFCWTCKKCICHQCALWGGMHGGHTFKPLAEIYEQHVTKVNEEVAKLRRRLMELISLVQEVERNVEAVRSAKDERVREIRNAVEMMIARLDTQLKNKLITLMGQKTSLTQETELLESLLQEVEHQLRSCSKSELISKSSEILMMFQQVHRKPMASFVTTPVPPDFTSELVPAYDSTTFVLENFSTLRQRADPVYSPPLQVSGLCWRLKVYPDGNGVVRGYYLSVFLELSAGLPETSKYEYRVEMVHQSTNDPTKNIIREFASDFEVGECWGYNRFFRLDLLANEGYLNRQNDTVILRFQVRSPTFFQKCRDQHWYIAQLEAAQTSYIQQINNLKERLAIELSRTQKSRGISPPDTHLSPQNDDSPETRSKKSGQSTEVLLENVAAPGLARDSKEEDEEKIQHEDFNHELSDGDLDVDLAGEDEVNHLDGSSSSASSTATSNTEENDIDEETMSGENDVEYSNNMELEEGDLMEDAAAAATPGASGTSHGYTSASGRPSRRGGGVLGSTASSSLLDIDPLILIHLLDLKDRNGMENLWGLQPRPPASLLQNRASSYSLKDRDQRRHQAMWRVPPDLKMLKRLKTQMAEVRSKMSDVKNQLSEVRSSNAGSCDGQPNFFSIEQGALAACGTDSCSKLQEIGMELLTKSSVTSCYIRNSASKKSNSPKPIRSGAAGSLSLRRAMDCGESNLRLKGDSQTSEGGLGSSKSSSRHHCPRPLASSNAAEALPKAEERPCEVSDSDVGVSGLNGLTAVEKTRKAGALVALGSNSKGYRTEGTQSGSLENNAETGELQGVLSEGASAGPEEGGACQKHVLHLLPGMSSDSDIECDTENEEQEDATSTSEGFNHAFSVQSSSEASERCSVFPEGDQVGPDDLSFVNGEDNTRLYLLKP

>duTRIM202

MNLSAPFHLPRGPRHLQEGEQPTARPTGAENESCPVIFATEKTMAEDIPAESFQDEASCSICLGFFQDPVSIHCGHNFCRACITRCWEEEEANFTCPRCKETAPERNLRPNRELAKIIEIAKRLSLQAAKGGAGGERLCEKHQEALKLFCEEDQTPICLVCRESQAHRAHPVVPIEEAAEEHKEKFQAHVQILKDRREKLLGLKMAEEGRSLNFLERVEEERQKVVAEIKELHQFVERQEQLLLGQLAKLDQEIVRRQEENIAKILEEISSVGEQIRELEEKCQQPAYELLQDSRNILSRLEKECAQKPLEASPELAETPTSLPQKNIALKEMLMKFQVSLTLDPETAHPRLVLSEDRKRVRWEDTRQPVPDNPKRFDSSRCVLGCEGFSTGRHYWEVEVGDGEAWAVGVAKESVRRKGRISVNPEVGIWAVGQCGSQYQALTSPTSPISLXRSPRGIGIYLDYEAGRVAFFDSHNLAPIFTYPPTSFAGEQILPLLCLGRGCQFTLSP

>duTRIM41

MAVAGDGGPGGRLNPVETLQEEAICAICLDYFLEPVSIGCGHNFCRVCISQLWGGEAEYEAAPGAAAAAGGGGGGGRGAVGLGDDVLEEELEDEEDELDEDELEVEQEEEEEEDGGGGGAEEEEDMWSEEEDDGELWEEPVEEDIWDAGVGGELYFGDEDYDEDVMEEDVEEEEEVEEEEEEVQTPPPPVLAARPRRQQTFTCPQCRKTFFERNFRPNLQLANMVQIIRQLHPYPPRLAPPAAGPSASGAAAGGPGGVLVAGGGQGPPNLCEKHQEPLKLFCEVDEQAICVVCRESRSHKHHSVVPLEEVVQDYKNKLQSHLEPLKKKLDAVLKQKSNEEEKITELRDKMKLEIKELESDFELLHQFLIGEQVLLLHQLEERYESLLVRQSSNISQLEEQSAALSRLIAEAEDKSKQDGLQLLKDIKGTFIRCENIKFQEPEMVPVDMGKKYRNYFLQDVVMRKMEKVFSKVPQADITLDPDTAHPRLSLSLDRRSVKLGERCKDLPDNPKRFDSDYCVLGSQGFTAGRHYWEVEVGGRRGWAVGAARETARRKEKTMGPHQEREIWCVGTNGKKYQALTATEQTALSPNERPRRFGVYLDYERGQICFYNAESMTHIHTFNASFHERIFPFFRILAKGTRIKICA

>duTRIM212

MALSGALERLQEEAICSICLEYMTEPVSVDCGHNFCHGCITKHCQEKCLWNDAPFSCPQCRAPCRRSSLRPNRQLANIVESIRQLRLGGGVAPGTLLCTQHDERLKLFCEVDEEAICVVCRESQHHRSHTVYPIEEAAQVYKVKLQKVLEHLSKEVEDMKKCESVAKMKIQECKETVKKKRERIVSEFGKLHRLLAAEEKLLLQKLEEEEKQILVMISESMFRLVEQKSLLDELILEIKEKMHLPDEGLLKDMKCILSRCEAVKFQTPKAVSVTLKEDYSIPERCLGMREMLKKFKVDVTLDPETAHPDLILSEDHKSVRRGGRKLLLSFFDNTRRFNSAPVVLGVQFFFSGRHYWEVHVGDKPEWGLGLCKEAASRKGNILFSPNNGYWVLRLQNGGNYEALTCPVSHLTLSIRPRCIGIFLDYEAGEISFYNVTDRSHLYTFTDKFSGKLRPLFYLGSFLGGKNAEPLVISWMRDMQGTGCIVL

>duTRIM42

MDENGCSFSICPCFSNCFYLTCHSRKKECCLCWRFLFTSEQNCTCFPCPYEEDKPYQCCHCSSAEHANCWWCCCSCSNDPDCKCCCCSGENSACQYYASKCCRNPISREQSRLSATFQSKDVMSRFRTRNNAFVNTPERNASNNAFRDQLACPLCKQLFLQPFMLPCNHCICEKCITKSKTKAEATDNFYIIVCPVCSKAHCLPYTNKIQLRKNYLRAKLAKKYMRRHGILRWRFDHSERPVHCDICREGRRMATKRCKTCGINMCNECLHLYHSENDAQDHIFTKADQADNEQWPCLLHCNSHLSEYCLDDHKLICRFCKNSLHNNHETIPLAAACSKEATSLSSTIVKFKQARQGVDNDLMEVILLKNNFQTYKDTKRKEIRNGFLKLRMVLHEQEKEMMELLENIELKKQKEISEYVNYTSSQLSYMDGLIQYAEEALKEESQIVFLQSAHCLVKEIEDAIPSIFHPSPLLREDPLRKLQLNFDELFATLQGLVPSLCEINQSDGKAEKNPYLFNPEIMLPMHVSSTHEDKQATLFRSTSLNSVFESGTMIKNTPGRQPSSMPPHHSTQSNNMCAFWDAACETPTKERKYQFVNLPSPEPIEKLLVPVPGHVVIYQTVVYPRSAKIYWTCPIEDVDFFEVEFYEVVGIGSDNIVQTQLDGQLSKIQQQNLEIRNLDPNTEYLFKVRAVNRSGEGEWSEICKITTSDEHRIIQDRWGIQKSMQGALHTLK

>duTRIM45

MAAAWRCPACAEPCAAPRLLPCLHSLCAPCLRRLGRLGEPARAGIATATTATTAAAISVLCPLCDAEVVLPPGGVGQLPPDYVALGRGRPGCDLCAQGAAVGRCQTCGAALCPFCRQAHRRQKKTASHAVTELESSKDCGQAGRPLFCPSHPSEELRLFCEQCDQPVCRDCVVDRHRQHPYDLASNVVHRHGDSLRVLLESTQQRVSTLEDALSQIDGMGSAVRVRAEAVAAEICLFASGYVKAIEEHRERLLKQLEDLKVQKENLLHLQKAQLQQLLLDMRTGMEFTERLLSSGSDLEILITKGVVASRLAKLNSVAYSTRPSVDDSIQFSPQERAGQCCGYEVFGAILNKVVDPAKCTLHGEDLCIARQNELTGFTLLCNDTTGEQMRRGGEAVRVTITHKDKKDCAVKPTVCDNGDGTYHVSYSPEEPGLYAVCVCVKGQHVQGSPFTLTVKSKFRKHQGVFHCCTFCSSGGQKAARCACGGTMPGGYQGCGHGHKGHPGCPHWSCCGQVTKSSECLGPPNDSSQRSLLRTVAL

>duTRIM46

MAEGEDLQTFTSIMDALVRISTSMKNMERELVCPVCKEMYKQPLALPCMHNVCHVCASEVLLQHGYLCCDPTSEPSSPAATPATRSPRLGRRGVPKPDRLDRLLKSGFGTYPGRKRGTVHPQTISFPCPACQRDIDLGERGLGSLFRNLTLERVVERYRQTINISAAIMCQFCKPPQLEATKGCTECKSSFCNECFKLYHPWGTQKAQHEPTPPTLTFRPKGLMCPEHKEEVTHYCKTCQRLVCQLCRVRRTHTSHKITPVLSAYQALREKLTKSLAYILSSQDTVQTQIAELEETVKHTEVNGSQAKEEVSQLIQGLCSMLEEKRASLLQAIEECQQERLASLHYQIQEHQAMLENSGMVGYAQEVLKETDHPCFVQAAKQLHNRILRATDSLQSFRPAATASFSHFQLDVSRELKLLTDLAFIKVPEAPVIDTQRTYAYDQIFLCWRLPQHSPPAWHYTVEYRKTDAKAKGLKLWQRREEVRGTSALVEYLDTDSVYVLRVKGCNKAGFGEYSEDIYLHTPPAPVLNFFLDNRWGFNRDRLAISKDQRAVRSVPGIPMLFAAERLMTSCHLSIDLVIGDVAITQGKSYWACCVDPSSYLVKVGVGLESKLQEWFQVPQDVVSPRYDPDSGHDSGAEDTTVDAPPPYAFLTIGMGKILLSHGSALTSRDPNGCTVPLPPRIGICLDYEQGKVSFYDAVSFRELWECGVDCSGPVCPAFCFIGGGALHLQELVANKQERKVTIGGFAKLD

>duTRIM47

MEATGGSSRAVPSAATSAALRLALAVPGLPDGPFGCPICLDILKDPVTVPCGHNFCQGCLGKLRGRTGPPDGGAAAGGAAAARCPLCQEPFPAALRLRKNRALCEVLPLLGAAGPASPPVEASPKPSPPGAGSPPLTAPNPPMAPGAEEEQQPERGEEKEEKKEEEEDEEEGEEGVLCDVCPEGARAAAARSCLVCLASFCGAHLEPHRRSPAFRAHRLVAPLRRLEEGLCPRHLQPFDGFCRAEQTCVCPRCRAHEHRAHDVVPLERERELKEAQQAKFLSNVENELEELAVTITQTKKMVELIKGVATKEKERVEKLFAEASEVLATFQKEVAGFIEDGERSMLGEAELDLRWKEERRAKLAQCKQNLQNVPSKDTIYFLQEFQALKIAMEDNLSPAPSFQNELNFTKCTQAVCAVKDMLAAVCKKQWDRLQGKGVDGLNFQEMEEVTESRFPDKPNNPACLESRDYFLKFAFIIDLDSDTADKFIQLFGTKGAKRVLSPILYPESPVRFINCEQVLGMNLMNRGNYYWEVEIIDGWVSIGVVTEDFNPREAFNRCRLGRNERSCCLQWNGQNYVAWFGGCESVIQQPFFHTIGVFLEYSEKTLTFYGVKDSKMTCLQQLKVSPVGKTQVNPFQNKINYHFSSLFSLKLKPAFFLESVDAHLQIGPLKKDCVSVLKRR

>duTRIM50

MTVWKRLHRHVLPAPMLKGGSMARRMSIDELEDQLLCPICLEVFKEPLMLQCGHSYCKSCVVSLSGELDGQFLCPVCRQTVDCSASPPNVTLARVIEALQSRGEAEPAPESCPTHHNPLSLFCETDQEVICGLCGTIGNHRQHKITPISTTYCRMKEELSVLLTDVHQYKRNLDEHFSKLINNKSRIANEADVFKWVIRKEFQELHRYIDEEKATFLESVEGKAAQLITSIESQVKQTSDALQRLKEIHSSLEMLSNENQLDFIRKYSSSQFRSELPSLLPGDGIFSPISFKPCFHQDDIKMTVWKRLHRHVLPAPEMLKLDPVTAHPLLELFKGDTVVQCGLYQRRDSNPKRFDSSSCILTCKGFSCGQHYWEVIVGTRNHWRVGIIKGTVSRKGKLSKSPENGVWLIGLKEGKVYEAFSTPRATLPLTARPQRIGIYLHYERGELTFYNADSPDELSPIYTFQAEFQGQLYPIVDLCWPERGPYSPPIILPPPGATRRPQGPCRQPLPEAPAEP

>duTRIM54

MNFAVGLKPLLAEARSMESLEKQLICPICLEMFTKPVVILPCQHNLCRKCANDVFQASNPLWQSRGSSAVPSGGRFRCPSCRHEVVLDRHGVYGLQRNLLVENIIDIYKQESARPLHAKAEQHLMCEEHEDERINIYCLRCEAPTCSLCKVFGAHKDCEVAPLPAVYQRQKSELSDGIAMLVAGNDRIQAIITQMEEICRTIEENGRRQKQHLGLRFDSLYSILEERKKELLQSIAREQEEKVQRVRGLIRQYGDHLEASSKLVETAIQAMEEPQMAVYLQHSKELLKKITDMSKVSMSSRPEPGYENMDHFSINVDYVAEMLRTIEFQTEPLGEEEGDGPMEGSEAAADEDRLDSLEAPEAAEDVGPRQKPASSPHGQH

>duTRIM55

MPLEKLGMSTSLNYKSFSKEQQTMDNLEKQLICPICLEMFTKPVVILPCQHNLCRKCASDIFQASNPYLPTRGGTTVASGGRFRCPSCRHEVVLDRHGVYGLQRNLLVENIIDIYKQESTRPERKCDQPMCEEHEDERINIYCLNCEMPTCSLCKVFGAHKDCQVAPLTNVYQRQKSELSDGIAVLVGSNDRVQGIVTQLEETCKTVEECCRRQKEQLCEKFDYLYSVLEERKNEMTQIITRTQEEKLEHVRSLMKKYADHLEAVSKLVESGIQFMEEPEMAVFLQNAKTLLQKITEASKGFQMEKIEDGYENMNQFTVNLSREEKIIREIDFDREEEVEEEEEETVDGEDLDEVHTESSGEEEEEEEKEEEEGAERASQPPQQDPEPQSAAVEPPAEPAPAPQPAPPAGQTGSEPSPSRTSGGNSREQ

>duTRIM59

MHHFEEELTCSICYSLFSDPRVLPCSHTFCRNCLEGVLDLSGNFSIWRPLRILLKCPNCRSVVEIPDSGTESLPINFALKAIIEKYRQEDHSDVATCSEHYRQPLNVYCLLDKKMVCGHCLTIGKHNGHPIDDLYSAYLKEKQSSGKILEQLTDKHWADVYLLIEKLKEQKSQCESVIQDDKKVVVLYFKKLSETLENKKQALLSALDEINRQVLEEYDPLIENLKKMREEQLELMSLNTSIQKEESPLVFLEKVDGVYQRLKALKEKQLPDVKPVEIYPRVGHLLKDVWSNTEIGQINKILTPKIKLIPKRKLQIKSKQKERGKSEELPPAVNSLAVILLFVIAAIALFAFSKLVSSFGIETVPTYISEFLHSIYQGFCAHLQTIVDALCHKFNLTVKFLGMIVPLWLFQ

>duTRIM215

MEASLTCAVCLSLFEEPVTLPLCSHNFCRGCVLECLASAQHQQRGPGPGQGQGQGQGQARLPRGGPGPGPGPGPGPGPGGGRVPCPLCRKLSPLPRGGAAALPVNTTLAEVVKLYRSGAAGEPGEQGPGLGLGPGQGPLALRGTCQKHPGRLVQLYCRMCRRAGCGQCVSEEHQGIFHSVNLLDTVFQEEKLTFFSSLKKMRIINEKLMNEVSSHPNDTDMVVNSEADIIALEFGEIFRTLEMKKQQLIEDLENQRSKKEKEFQIWKKMKETHKKTIENFLKDCEKLVHECDPQRFLEVACGLNARMKTQLDLMSIASSYEKPPEYTQKKMDIKPVVNEILALKLVPVNVDMAKDLPSGGNQNSTKNTIKQWQDQKNMPKMFIPVAGQEEALTDGGRICTRLMSISEMSAFQNMSHEELRYKYYMERQKLADEFKTQTLPANKKHKFVAAEALKDKSSGIPSVSLPTTANNTNGVNMGILQKEGGFDELNFFGTGNHRIPYTATNFSEKNGNLSLVHERGSEETTTPALSENTKDLLMREKMPMQSSAVTVSNGVDTNSSILGGVAASVAVTVSNSEFLDVSAERPSASPFAFGACSNSLPRVTKDAATFSFKKEASKYVFPKFYLGKCDREAKAVNQGGNKFGKHSSVTKTTVSDASNSCNVELAENKKTCFSFPFDHSERDCSAVSGVSNSSKILPLSSFSNQSEKPADQNTLSHMVENAFSPKKTVENDTLKPSVSVEQKANASESITTAPCSTSETGIAAGVDDVSESSLLPSTCVFSFKNNCFQLPSPVFSFGSIVKNTTDSLTSSSIFLSSNGTEKSEQEKMKPVDKTPPNLVKSASPECTETASRHSHPKDEGSFPMGSSKKIESAEILADSNSSCSPLLCSAVLPAKDENASSDHLTITSTQQEVKVKDQGSIAENNCSIPGREDELKSRMLQNAACSAPGMCNDSVSRASVLTVNESGGMLSDSDSDTEALSQTSVSSDTSSVSEYFSVAEDKIPTRRKSEA

>duTRIM62

MACSLKDELLCSICLSIYQDPVSFGCEHYFCRRCITEHWVRQEPQGARDCPECRRTFAEPTLAPSLKLANIVERYSAFPLDAILGAQRSPFPCKDHEKVKLFCLTDRAVVCFFCDEPAVHEQHQVTNVDDAFEELQRELKEQLQGLQESERGHTEALHLLKRQLAETKSSAKSLRATIGEAFERLHRLLRERQKAMLEELEADTARTLTDIEQKIQRYSQQLRKVQEGSQILQERLAEADKHAFLAGVASLSERLKGKIHETNLTYEDFPTSKYMGPLQYTIWKSLFQDIHPVPAALTLDPGTAHQRLILSDDCTIVAYGNLHPQPLQDSPRRFDVEVSVLGTEAFGGGVHYWEVVVSEKTQWMIGLAHEAVTRKGSIQIQPSRGFYCIVMHDGNQYSACTEPWTRLNVKSKLEKVGVFLDYDKGLLIFYNADDMSWLYTFREKFPGKLCSYFSPGQSHANGKNVQPLRINTVRI

>duTRIM63

MGCHVHFGVQRSPVCRGGRTHRAWCCHPPRKPPTHPICAQHPKSAHILWVQTQMLHTNHSTSLVLPIPHPKPPVGPSSTLYPPTGPRCPPARAAGPRGQPVPSLFTAGLAPRLSPPCDRWQRLAGHPSRPADFWAPRDAERVIKSRARGASGRSGGCSDEVPYRMDFQPSILRDGSPMESLEKQLLCPICLEMFSKPVVILPCQHNLCRKCANDVFQAANPCWQSRGSIIPGGRFRCPSCRHEVLLDRHGVYGLQRNLLVENIIDIYKQECSSRPMKKGEHPMCKEHEDERINIYCVTCEVPTCSMCKVFGAHKDCEVAPLQSVFQGQKSELNNCISMLVAGNDRIQTIISQLEDSCRSTEENSEAAKRELCARFDTFVALLEEKKTELLGRISREQEDKTGFVQGLIHKYKEQLEKSSRLVETAIQAMEETGEATFLMNAKQLIKTIVEASKGGRLEKIEQGYESMDAFSVSLDHLADAVRALDFEADEEDEEFYEEVEEETEGDSAPGRMVTAPQ

>duTRIM65

MPGSSFQKKKSEIKYRGTCIHGNTLASDAVSLCHVSPRAVTMALSPPAAPCCSQGGTGQSPHGRGASDPPLGVPLRVHLARSPDPPLSSASPSSSSFPSGVSVSPASFPRCPDPWHEMALSISPKLEEKLVCSICLELFKVPITLPCGHNFCKRCISDHQGKQEQAAAGAKQGFSCPECRQSCAPQLELKKNVTLSKVLELVRASKTGVKQCEVTPGGLCPRHGRPLELYCEDEQRCICCVCTVQQCQRHRRALLEDVHSRKQALLEKSVKEAQEESEKIERALQELEERTQSIKDSSEGLRSVILGKFAHLEKSLQAFQCQMVAKVEQELSAALRRVEENSNTLKGHLDTLRQHQEQARDLLVSTTDHRTFLEEFPLLPAWESLAVPPPVQFDAAGVVEPLSEILAGISRLLLEDLPGAVAPKSPDPIVPGPVQPKGTEMKVATPLPKCQIRAEFLKDHRNLTFDPDTANKYLELSKGQRRARHGTGAAGGWQERGSPFEPWQVLCEQGYGQGCHYWEVAISSHSVILGATYRSLPQRQPPGHKFSIGLDGGSWGLQVREDGYLAWHKGREEKIQERLYTQLGVRLDYGRGLLSFYGLGEETRLIHCFHAVFTEPLYPVFWLCEGRAVTLGRRDQPQPAPQAPSSGQDGVQ

>duTRIM66

MSHFVTLHVFDVKFQPTAVGTLDLLRNCLVCKRDLGMRDPRMLPCLHSFCKDCLPGLIQGYSCIPTGYEVLYEGILSCPVCKQTCFARDVVENFFLKDFHTDKSAMAKSCSMCKEKKPAHSLCTSCNKWLCSTCTEEHRHGNETGDRFLSVSLKGCTATEDEASEFSLFCPMHAQEPLKLFCETCDILTCHSCLLTEHKEHRFRHLDEALQNQRAILKNVIAKVEEKKNGIQVSAKQIEDRLLEVKHLYKKVENQIKMAKMVLINEINKRTNILLEQLEKITSERKQKLEQQLQGVVVLSRQVEHVQNFTSWAVCSKNSIPFLFSKELIVFQIQRLLETNCNTDVGPPLKIRFTWDPSYWTKQLSNFGAFTMEGGHISHSDVLLYGNVQGLQTSLYHGHYSPASQLEPVNSQPHQLPPAVQCPMPMCCSHCLSVPHPNKAQPSHQNINRHQNFQHPELHQQQFPLQYSMQQRDKEQRDVPPPLKLTQPRLEQQSRPESENTSGKMGKHLPPQQLQQTAPLGYAVVSHEAQQVHTSHPQSFRTQTALQTSTVQVQLGHLQKIKSNHLQQPPQQQQLPPASPLSGQNENTHKQVIQQSLDIMHHQFELEEMKKDLELLLQAQGQSSFQLNQPKPAQHVQQTIVGQINYIVRQPAPVQQQIQDESPVCESSPELDVQKPVVPLDRSDIPSLSQSLDEEASVSSHSPESTLQQSAYNPVRKRSASLSIVGFSNDLEMELPSTRLSRSADPQIQDVAAVALGSSPNTRCDCDTPPEPVPSYSLVLGRAPGDLSPGLAPGDTLQSSAKCKLENEDLNTVDHPLENSMASDGQDVVNELSLSMQKVLEEPINLSVKKSQRCTSPSEVLSNNSSLPVNDRMRQLRNEEDSNNCEKEHLEMDMKSNQDVRSGPRELKIPYVRLERLKIHASESGELPVFKLQPQDSEQEGSFLLIIECGTQSSSMAIRINKDGPPEGLKCKEESMEDRKFVITQAEGQIQSPPVDTLPSDQKFSNGTSLTMKKSPVTQEVNTIENEDFCAVCLNGGELLCCDHCPKVFHLSCHVPALLSFPVGEWVCTLCRNPVKPEVEYDCENTRYGHSYNAQYGLDDYDQKKCEKLVLSLFCSSLSLPFHEPVSPLARHYYQIIKRPMDLSIIRKKLQKKDKFHYSAPEELVTDVRLMFWNCAKFNYPDSEVAEAGRCLDVFFESKLKEIYPDRHFPSMQQDDSDSEEVESQNSKMPPQDFQWPSYGQECIQPKRRRRHAVRLQKQKHLVAKWPPC

>duTRIM67

MEEELKCPVCGSLFREPIILPCSHNVCLPCARTIAVQTPESEQHLPALLHPRGTAAPAPTPTTTTTTPGPPGGSAAPLDPECSAGGGGDHADKLSLHSETDSGYGSYTPSLKSPNGVRVLPLVPAPPGGAAAAAAPRGAVPSPSPIPGPVGSSLTCPQCHRSASLEQRGLRGFQRNRLLEAIVQRYRQGRTARCQLCDRSPAEPAAVLCEQCEVLYCAACQLRCHPARGPFAKHRLAPPPGQPGPPPGAEGKGAGGGRKPATCAEHELEQYSMYCLSCRSPVCYQCLEDGRHGKHDVKALGAMWKQHKAQLSQALNGVSDKAKEAKEFLVQLKNLLQQIQENGLDYEACLVAQCDALVDALTRQKAKLLTKVTKEREHKLKVVWDQINHCTLKLRQSTGLMEYCLEVIKENDPSGFFQISDALIKRVQVSQEQWVKGALEPKVSAEFDLTLDSEPLLEAIHHLDFIQMKLPPVPLLQLEKCCTRNNSVTLAWRMPPLSHNPVEGYILELDDGDGGQFREVYVGKETLCTIDGLHFNSTYNARVKAFNSSGVGPYSKTVILQTSDVAWFTFDPSSAHRDIVLSNDNQTATCSSYDDRVVLGTAAFSKGVHYWELHVDRYDNHPDPAFGIARINVVKDMMLGKDDKAWAMYVDNNRSWFMHCNSHTNRTEGGVSKGATVGVLLDLNKHNLTFYINGQQQGPPAFENIEGVFMPALSLNRNVQVTLQTGLEVPQCVKQPKLPSN

>duTRIM71

MASFPEADFQICPLCKEMCGSPAPLSSNSSTSSSSSQTSSSSGGVEEEAAGGGSSSCGGGPPRRLHVLPCLHAFCRQCLEAQRHPGAGDALKLRCPICDQKVVISEPSGMDALPSSNFLLSNLLDVVVVAAAADEHKNGRPVAPGPSAAGSAPGVGGGNNRHHGRPPPHRSAPPGSSPAAAVSSSSSSSSSSSAAPSSTSSSSSSSSGGGGGSSTAALLLRRPHSRQGEPRCSSCDEGNAASSRCLDCQEHLCDNCVRAHQRVRLTKDHFIERFAAGPPPSAAAAAAGPAAPLALSPPYPASPYNILSVFPDRASYCQHHDDEVLHFYCDTCSVPICRECTMGRHVGHSFIYLQDALQDSRTLTIQLLADAQQGRQAIQLSIEQAQAVAEQVEMKAKVVQSEVKAVTTRHKKALEERECELLWKVEKIRQVKAKSLYLQVEKLRQNLNKLDNTISAVQQVLEEGRTMDILLARDRMLAQVQELKNVRGLLQPQEDDRIMFTPPDQALYMAIKSMGFVSSGAFAPLTKATGEGLKRALQGKVASFTVIGYDHDGEPRLSGGDMISAVVMGPDGNLFGADVSDQQNGTYLVSYRPQLEGEHLVSVMMCNQHIENSPFKVMVKSGRSYIGIGLPGLSFGSEGDSDGKLCRPWGVSVDKEGYIIVADRSNNRIQVFKPCGTFHHKFGTLGSRPGQFDRPAGVACDISRRIVVADKDNHRIQIFTFEGQFILKFGEKGTKNGQFNYPWDVAVNAEGKILVSDTRNHRVQLFGPDGAFLNKYGFEGALWKHFDSPRGVTFNHEGHLVVTDFNNHRLLVIHADCQSARFLGSEGSGNGQFLRPQGVAVDQEGRIIVADSRNHRVQIFESNGSFLCKFGTQGSGFGQMDRPSGIAVTPDGMIVVVDFGNNRILVF
