# Supplementary material for: Evolution and expression of the duck TRIM gene repertoire
Source: Front Immunol. 2023 Aug 9;14:1220081. doi: 10.3389/fimmu.2023.1220081 (PMC10445537; doi:10.3389/fimmu.2023.1220081)
Supplement: Supplementary File 5 — Nucleotide and amino acid sequences of chicken TRIM or TRIM-like genes used in this study in fasta format. Nucleotide sequences only for genes pulled from de novo chicken transcriptome. [file DataSheet_5.docx]

>chTRIM46_AA

MRFSRLLKAVREQTKSAAPSSPPWMVSHSVAASCLLYICIPANSSSDSSDD

SAREVPVPTAPRFRRSVHRRAPTAAGGAAGTGSGAMAEGEDLQTFTSIMDALVRISTSMK

NMELELVCPVCKEMFKQPLALPCTHNVCHVCASEVLLQHGFLCCDPNSEPSSPAATPSTR

SPRMARRGGPKPDRLDRLLKSGFGTYPGRKRSSAHAQSVPFPCPLCRRDVDLGERGLAGL

FRNPTLERVVERYRQSVSAAAAVVCQFCKAPPPEATKGCTECQASFCNECFKLYHPWGTQ

KAQHEPTPPGITFRTKGLLCPEHKEEVTHYCKTCQRLVCQLCRVRRAHSSHKISPVLSAY

QALREKLSKSLAYILSRQDAVQSHIAELEETIKRTEANGSQAKEAVAQLMEALSAALDEK

RTALLRSIEECQQERLGALQLQVREHRAMLDSSGMLGYAHEVLKETDQPCFVQAAKQLHG

RILRATDALQSFRPAASASFSHFQLDVSRELKLLTDLAFIKAPEAPVIDTQRTYAYDQIF

LCWRLPQHSPPAWHYTVEYRKSDPKAKGLKLWQRREEVRGSSAVLEDPDTDSVYVLRVRG

CNKAGYGDYSEDIYLHTPPAPVLNFFLDNRWGLNRDRLAVSKDQRAVRSVPGVPMLFAAE

RLLSSCHLSVEVVIGDVAVTQGRSYWACCVDPNSYLVKVGVGLESKLQEWFQAPQDVVSP

RYDPDSGHDSGAEDTTVESPPPYAFLTIGMGKILLSHGSAPSPRDPPGCTAPLPPRVGIC

LDYEQGRVSFYDAVSFRELCDLGVDCSGPVCPAFCFVGGGALQLQELVAGRQERRGTGGG

FAKTD

>chTRIM46_nt

ATGAGGTTCTCCAGGTTGTTGAAGGCGGTGAGGGAGCAGACGAAGAGCGCGGCCCCCAGCAGCCCCCCCTGGATGGTCAGCCACTCGGTGGCCGCCAGCTGCCTGCTGTACATCTGCATCCCCGCGAACAGCAGCAGCGACAGCAGCGACGACAGCGCCAGGGAGGTGCCCGTGCCTACAGCTCCCCGGTTCCGCCGCTCCGTGCATCGCAGAGCGCCGACGGCAGCCGGTGGTGCCGCCGGTACCGGGAGCGGAGCCATGGCCGAGGGCGAGGATCTGCAGACCTTCACCTCCATCATGGATGCGCTCGTCCGCATCAGCACCAGCATGAAGAACATGGAGCTGGAGTTGGTGTGCCCGGTGTGCAAGGAGATGTTCAAGCAGCCGCTGGCGCTGCCCTGCACGCACAACGTGTGCCACGTCTGCGCCAGCGAAGTGCTGCTGCAGCACGGCTTCCTGTGCTGCGACCCCAACTCCGAGCCCTCCTCCCCCGCCGCCACCCCCTCCACCCGCAGCCCCCGCATGGCGCGCAGGGGGGGCCCCAAACCCGACCGGCTGGACAGGCTGCTGAAGTCAGGTTTCGGCACCTACCCGGGCCGGAAGCGCAGCTCCGCGCACGCGCAGTCGGTGCCGTTTCCGTGCCCGCTGTGCCGCCGCGACGTGGATTTGGGGGAGCGCGGCCTGGCGGGGCTGTTCCGCAACCCCACGTTGGAGCGCGTGGTGGAGCGCTACCGGCAGAGCGTCAGCGCCGCCGCCGCCGTCGTGTGTCAGTTCTGCAAAGCGCCGCCCCCGGAGGCCACCAAAGGCTGCACGGAGTGCCAGGCGAGCTTCTGCAACGAGTGCTTCAAGCTCTACCACCCCTGGGGCACGCAGAAGGCGCAGCACGAGCCCACGCCGCCCGGCATCACCTTCCGCACCAAGGGCCTGCTGTGCCCGGAGCACAAGGAGGAGGTGACGCATTACTGCAAGACGTGCCAGCGCCTCGTCTGCCAGCTCTGCCGCGTGCGCCGCGCGCACAGCAGCCATAAGATCAGCCCCGTGCTCAGCGCATACCAGGCCCTCAGGGAGAAGCTGAGCAAAAGCCTGGCGTATATCCTGAGCCGGCAGGACGCGGTGCAGAGCCACATCGCCGAGCTGGAGGAGACCATCAAACGCACCGAGGCCAACGGCTCGCAGGCCAAGGAGGCGGTGGCGCAGCTGATGGAGGCGCTGAGCGCGGCGCTGGACGAGAAGCGGACGGCGCTGCTGCGGAGCATCGAGGAGTGCCAACAGGAGCGGCTGGGGGCGCTGCAGCTGCAGGTCCGGGAGCACCGCGCCATGCTGGACAGCTCCGGAATGCTGGGATACGCGCACGAGGTGCTGAAGGAGACCGACCAGCCGTGCTTCGTGCAGGCGGCCAAACAGCTGCACGGCAGGATCCTCAGAGCCACCGACGCCCTTCAGAGTTTCCGCCCCGCAGCGTCCGCCTCCTTCAGCCACTTCCAGCTGGACGTCAGCAGGGAGCTGAAGCTGCTCACCGACCTGGCCTTCATCAAAGCCCCCGAGGCTCCGGTGATCGACACTCAGCGCACGTACGCGTACGATCAGATCTTCCTTTGCTGGCGGCTCCCGCAGCACTCCCCCCCCGCGTGGCATTACACGGTGGAGTACCGGAAGTCCGACCCCAAAGCCAAAGGGCTGAAGCTGTGGCAGAGGCGGGAGGAGGTGCGGGGCAGCAGCGCGGTGCTGGAGGACCCGGACACCGACAGCGTCTACGTGCTGCGGGTGCGGGGCTGCAACAAGGCGGGCTACGGCGACTACAGCGAGGACATCTACCTGCACACCCCGCCCGCGCCAGTCCTGAACTTCTTCCTGGACAACCGGTGGGGTTTGAACCGGGACCGCCTGGCCGTCAGTAAGGACCAGCGCGCCGTGCGCAGCGTGCCGGGCGTCCCAATGCTGTTCGCCGCGGAGCGCCTGCTGAGCAGCTGCCACCTCTCCGTCGAGGTGGTGATCGGCGACGTGGCCGTGACGCAGGGCAGGAGCTACTGGGCGTGCTGCGTGGACCCCAACTCCTACCTCGTCAAGGTGGGAGTGGGGCTGGAGAGCAAACTGCAGGAGTGGTTCCAGGCGCCGCAGGACGTGGTGAGCCCCAGATACGACCCCGACAGCGGCCACGACAGCGGCGCGGAGGACACGACGGTGGAATCCCCCCCCCCCTACGCCTTCCTGACCATCGGCATGGGCAAAATCCTGCTGTCCCACGGCTCGGCCCCCTCCCCTCGCGACCCCCCCGGCTGCACGGCGCCCCTCCCGCCCCGCGTGGGGATCTGCCTGGACTACGAGCAGGGCAGGGTGAGCTTTTACGACGCCGTGTCGTTCCGGGAGCTGTGCGATTTGGGCGTGGATTGCTCCGGCCCGGTGTGCCCCGCCTTCTGCTTCGTCGGGGGGGGCGCTCTGCAGCTGCAGGAGTTGGTGGCCGGCAGGCAGGAGCGCAGGGGCACCGGCGGGGGCTTCGCCAAGACGGACTGA

>chRNF39_AA

MSASESAPPPIPV

PKSSPVPVSSSVPVSKPIPVPKSSPVPKSAPVPMSDPVPVPVPTSIPVSVPESVPVPKPP

PVPMSDPVPVPVPKSSSVPVPTSIPVSIPVPKPPPVPMPVPFPVPVPVPVPSLPLPVQRL

LTASRCSACGSPARDPVLLSCDHRFCRRCVPSAATAASAAVRCPRCRRSCPRSRLRTAVA

AAVEGRIAERLAAAAAAAVGNGNDGRGNGRRRERGSLGAQLRADAAPPSRPPPPKD

>chRNF39_nt

ATGTCCGCCTCGGAGTCCGCTCCGCCGCCGATCCCGGTTCCGAAGTCCTCCCCGGTCCCGGTCTCCTCCTCGGTCCCGGTCTCGAAGCCCATCCCGGTTCCGAAGTCCTCCCCGGTTCCGAAGTCTGCCCCGGTTCCGATGTCGGACCCGGTGCCGGTCCCGGTTCCGACGTCCATCCCGGTGTCAGTCCCGGAATCCGTCCCGGTTCCGAAGCCCCCCCCGGTCCCGATGTCGGACCCGGTGCCGGTCCCGGTTCCGAAGTCCTCCTCGGTCCCGGTTCCGACGTCCATCCCGGTATCCATCCCGGTCCCGAAGCCCCCCCCGGTCCCAATGCCGGTTCCGTTTCCGGTTCCGGTTCCGGTCCCGGTACCGTCGCTGCCTCTCCCCGTGCAGCGGCTGCTGACGGCGTCGCGGTGCTCCGCCTGCGGTTCCCCCGCCCGGGATCCGGTGCTGCTCAGCTGCGATCACCGCTTCTGCCGCCGCTGCGTCCCCTCCGCCGCCACCGCCGCCTCCGCCGCCGTTCGGTGCCCGCGGTGCCGCCGGAGCTGTCCCCGATCGCGGCTGCGGACGGCGGTGGCGGCGGCGGTGGAGGGGCGGATCGCGGAGCGGTTGGCGGCGGCGGCGGCGGCGGCGGTGGGGAACGGAAACGACGGGAGAGGAAACGGGAGGAGGAGGGAGAGGGGAAGTTTGGGGGCTCAGCTCCGCGCCGACGCCGCCCCCCCCTCCCGTCCGCCGCCCCCCAAAGAC

>chTRIM39_AA

MAASDPLQTLRMDTNCSPPPM

SSDPLETLRAEASCSVCLDFLQDPVLIECGHNFCRRCINRWWAGMEAPFPCPVCRKSARR

RRLRPNRQLGNMVEAARRLGVAKKRAGGPLGGPLGGPLGGGGGDEEAVVVVEGDEEEEEE

ERRCAEHGRPLRSFCMQDMRALCAACEMRCAAGRHQLSAVRQAEAECLERLQRCVEPLEQ

RLLALRHCQEQEDRKPAELKRSACSLRRRIAAEFAELRRALSAEEAELQRRVDDEEADIA

QRLRRNAAALRQRRAHLLRLLRDAQRGGTDRGRPDLPALLSSCEEEAARSDPPPSVPIAL

QKNFRSFPRQYFTLRKMARRLLGDVTLDPSTAHPNLLLSRDLKSVRYTERRRPHLPPSAR

RFTAYPCVLASRAITSGRHYWEVEVGRKTHWALGVCGDSVRRDGEPTELPHGGFWRVRLW

NGDRYAATTAPFTPISPRVPPTRVGVFVDYEAGEVSFYNVTDRSHLYTFTETFTEKIWPL

FYPGIRAGRRNAAPLSICEPTDWE

>chTRIM39_nt

ATGGCGGCTTCGGACCCCCTGCAGACCCTCCGCATGGACACCAACTGCTCGCCCCCCCCCATGTCCTCGGACCCCCTGGAGACCCTCCGTGCGGAGGCGAGCTGCTCGGTGTGCCTGGACTTCCTGCAGGACCCGGTGCTGATCGAATGCGGTCACAACTTCTGCCGCCGTTGCATTAACCGGTGGTGGGCCGGCATGGAGGCTCCGTTCCCGTGCCCGGTGTGCCGCAAGAGCGCCCGGCGCCGCCGCCTCCGCCCCAACCGCCAGCTGGGCAATATGGTGGAGGCCGCCCGGAGGCTCGGAGTGGCCAAGAAGAGGGCGGGGGGTCCTTTGGGGGGTCCTTTGGGGGGTCCTTTGGGGGGTGGGGGGGGCGATGAGGAGGCGGTGGTGGTGGTGGAGGGCGATGAAGAGGAGGAGGAGGAGGAGAGGCGGTGCGCGGAGCACGGGAGGCCGCTGCGCAGCTTCTGCATGCAGGACATGCGGGCGCTGTGCGCCGCCTGCGAGATGCGCTGCGCCGCCGGCAGACACCAACTGAGCGCCGTGCGACAGGCCGAAGCCGAGTGCCTGGAGCGGCTGCAGCGCTGCGTGGAGCCGTTGGAGCAGCGTCTGCTCGCACTGCGGCACTGCCAGGAGCAGGAGGACAGGAAACCCGCCGAGCTGAAGCGTTCGGCGTGCTCGCTGCGGCGCCGCATCGCGGCGGAGTTCGCGGAGCTGCGGCGCGCGTTGTCGGCCGAGGAGGCGGAGCTGCAGCGGCGCGTGGACGACGAGGAGGCCGACATCGCGCAGCGCCTCCGCCGCAACGCCGCTGCGCTGCGGCAGCGCCGGGCGCACCTCCTGCGGCTGCTCCGAGACGCACAGCGCGGCGGGACCGACCGCGGCCGGCCGGACCTCCCGGCGCTGCTGTCCAGCTGTGAGGAGGAGGCAGCGCGGTCGGACCCCCCCCCCTCCGTCCCCATCGCCCTCCAGAAGAACTTCCGCAGTTTCCCCCGGCAGTACTTCACGCTGCGCAAGATGGCGCGACGCCTGCTGGGTGACGTCACTCTGGACCCCTCCACCGCCCACCCCAACCTCCTCCTCTCCCGGGACCTCAAAAGCGTCCGTTACACGGAGCGCCGCCGCCCCCACCTCCCCCCCAGCGCCCGCCGTTTCACGGCCTACCCGTGCGTGTTGGCCTCGCGGGCCATCACTTCCGGCCGCCATTACTGGGAGGTGGAAGTAGGCCGCAAAACCCATTGGGCTTTGGGGGTCTGCGGGGACTCCGTCCGTCGCGACGGGGAACCCACCGAACTGCCCCACGGCGGCTTCTGGAGGGTCCGCCTATGGAACGGAGACCGCTACGCCGCCACCACCGCCCCCTTCACCCCCATCAGCCCCCGCGTGCCCCCCACCCGCGTCGGGGTCTTCGTGGATTACGAGGCGGGGGAGGTCTCCTTCTACAACGTCACCGACCGCTCCCACCTCTACACCTTCACCGAGACCTTCACCGAGAAAATATGGCCGCTCTTCTACCCCGGGATCCGCGCCGGGCGCCGCAACGCCGCCCCGCTCAGCATCTGCGAGCCCACCGACTGGGAGTGA

>chFSD1_XP_025000152.1

MRGVGDSAVSQPDPMAQPDPIEHPDPMAHPDPIEHPDSVAYPDPMAQPDPMAHPDLMAQPDPIEHPNPMA

QPNPMQQPNPMAYPDPMAHPDPTAHPDPTAQPNPMQQPNSMAHPDPMAHPDPMAYPDPMAQPDPIEHPNP

MAQPDAMAYPESTAHPDPMAHPDHMAHPDPIEHPNPMAQPDAMAHPEPTAQPNPMAHPDHMAHPDPIEHP

NPMAYPDPMAQPDPIEHPNPMAQPNPMQQPDPMAYPDPMAQPNRMAHPDPIEHPDPMAHSDPMAHPDPMA

YPDPMAYPDPMAHSDPMALPAPTAQPAPHGSAAHSVRTELTPLCRPAVSPPLGHTVTAEQRSPRRPHGDS

HEGWGAAGGPSPPRPPTSCSLPRPPPPPFPRGGSDVGRSGARRQRCRSAPPGGLCPAVGGGLGPLGEARG

LRGTTHPSTPRARRTAIRRIPARPAAALHFPACPAPDHRRRDLSRHAPRQRDLSRHAPRPHYTSQHPPRP

SARQWGASQRAPRADYTSRRAPRAGRSGDAAAPLLLVPAGPGGGGGGPEALRKIITTLAVKNEEIQNFIY

SLKQMLQNVEDNSARVQEDLEGEFQSLYALLDELKDGMLMKIKQDRASRTYELQAQLAACAKALESSEEL

LEAANHTLATANNHDFAQAAKQIKDSVTMAPAFRLSLKAKVSDNMSHLMVDFGQERRLLQGLAFLPVPST

PEIDLAESLVADNCVTLRWRMPDEDSKIDHYVLEYRRTNFEGPPRAKEEQPWMVVEGIKGTEYTLAGLKF

DMKYMNFRVRACNKAVAGEFSEAVTLETRAFMFRLDASTCHQNLRVEDFSVEWDATGGKVQDVKAREKDG

KGRTASPANSPARAVQSPKRMSLGRAGRDRFTAESYTVLGDTPIDGAEHYWEVRYDRDSKAFAAGVAYRS

LGRFDPLGKTSASWCLHLNNWLQLSFSAKHANKAKALDGPVPECIGVYCNFHEGFLSFYNARTKQLLHTF

KARFAQPVLPAFMVWCGSFQVSSGLQVPSAVKCLQKRNSAASSSTASLP

>chFSD2_XP_046755109.1

MSSRPVSAREYESSDQFPGHSASPPVPNSSETEGLIFYHMDLYGSNERFDIFPEEPSGQGSRFLGNTRRE

SALSSEKCQNSHEAGYDMEKEVAELAKMYGLDEDREKELELLGGHLETVGSKWLPAHTQKSGLQDSVYNR

SKSSSPVKDQSKSTKHQELPDEVSQDEDQHKARTENEADSHTWFREGLSSSGVSDERSSQAVSEEEEMAD

VFCCTCKIPIRAFDKLFGEHKDHEVAQLSSAVESEKEEIHKNMCKLEEQIAQVENFASHLEEIFITVEEN

FGRQEQNFEVHYNDAVQVLAQKYEEQLEALGEEKKQKLEALYEQLVSCGEHLDTCKELMDTTHKLYQEND

KVTFMKEAVTMVDRLEKFLKKEVNLKLSTHPDFEERAIDFSEVEQLMNSINTIPAPCAPVINPQAPNAAT

GTSLRVCWGLFSDDTVECYQLCYKPVSNERHSDEQAEHMLKVKETYCTISNLLPNTQYEFWVSASNASGI

SPPSERAVYVTAPSPPIIKSKKIRSCENAALVCWESRDVNPVDSYTVELSKLTDEENGDIITESIVGIPN

CEALIHLQPAQSYHICVKALNLGGSSERSEPVLIHTTGTYFCLNEDTAHPLLAILDDGFTIACDELENPE

CDLPVYDNSFTRCIGILGSLIPFPGKHYWEVEAEEDTEYRIGVAFENTPRHGYLGANNSSWCMRHIITPS

RHKYEFLHSGMTPDIRITVPPTKIGILLDYESYRLSFYNADIAQHLYTFNSHFQHYVHPCFALETPGTLK

VHTGITVPPWTALL

>chFSD1L_XP_004949409.1

MEEQRETLQRIISTLASKNDEIHNFIDMLNQTIQNVQINSSNVISELDDEFDALYSVLDEMRGSMASTIQ

QEQARKIQALQNQLSNCSNALESSEELLELAAQSLDIKDPVKFSEAARQIKDRVTMASAFRISLKPEVSD

SMTHLMVDFTRERHALQTVKFLPVPKAPIIDLAACLVIDNCITVSWRMPEEDSRVDHFVLEYRKTNFDGL

ARVKDEQSWELIDYIKATEYTLSGLKFDTKYINVRVRACNKAVAGEYSDPVTLETKAFVFDLDNTSSHVN

LKVEDSYVEWEPTGGRGQEKVKGKENKSSGQNTVLKSSKRSGASPKRTSIGGKAPVKGSRDRFAGESYTV

LGNNAIESGQHYWEVRAQKDCKSYSVGVAYKTLGKYDQLGKTNISWCIHVNNWLQNTLAAKHNNKTKTLD

MPVPDRLGVYCNFDGGQLTFYNAISKVPLYTFRMKFTQPLLPAFMIRCGGLAVSTGLQVPSAVKTLQKNE

NGLSGSTGSLNNIAQ

>chBSPRY_XP_415546.4

MARRGAEPRALPERAAELRNRLVDRCERLQLQSAAIAKHVAEVLPARSQGVLSAANSARELVIQRLIFVG

KVCENEEQRLLEEVHAEEERAQHSILTQRAHWTEALQKLSALRTYLVDTITNLDDQDLVNAEQEIFERTE

VAEGILEPRESAKLNFNQQRVQSPLLHRLWASAVLCCVAGSQEISIDEKTVSPYLSLSEDKKTLTFSPKK

AKLDLDGPERFDHWPNALATAAFHTGLHAWKVSVEKSCAYKLGVCYGSLPRKGPGNEVRLGFNAASWVFS

RYDKEFRFLHAGQPQPVELLRAPAEIGVLVDFAGGEVLFYDPDSCTILFSHREAFAAPLYPVFAVAHQSI

SLSQ

>chNHLRC1_XP_040519561.1

MASVLRAPGSVPHRRSRPAGGSRRPAEARPVAAGMAEAADEAELGLLECRVCFERYGPGGQRRPRNLPCG

HVLCRGCVEALGGPERRRLECPFCRRPCGLCETSDCLPLLQLLELLGPGGRSPSALGRAAGGAAPPRLQL

SLGGWGSLLNPTGVAACRRSGRLAVAHDGKKRIHVFGPSGSCLQRFGERGDAGNDIKYPLGVAVTADGHV

VVTDGGDRSVKAFDFEGRGTLAVREGFCLPWGLGATPECDVMVTDAEAGALYRLTADFGSGKLKKCQLVR

SKLTNPRGVAVCPSSGAVVVIEHLKTEGPSSCSTRMKIFSADMTLIGQMDSFGLNLVFPSKIYTTAVAFD

KEGCIVVTDVCSQAVICLGKPEEFPIFNPLVSHGLSYPVGLTYTANNSLVVLDSGDHSVKIYSSA

>chRNF207_XP_417536.2

MAGGIFSPLGSCAELEQGAWHPLVCLLCHGPFQQPCLLDCYHAFCASCLRGRAASGRLRCPLCGHPSVVR

GGTGLPPVDRLLQFLVDSSAEGSEDAQCANCDRLCAEADLDTMCFCNTCSQPLCAPCREETHRAKVFARH

EIVSLSKRTKAIHKKCPLHEEPYIMFSTEKKSMLCINCFRDMQGESRAHCIDIETAYMQGCEKLDQAVLA

VKELQTSTREAIVLLKAMIEEVRNSASEEESAINALFSGMQEQLSDRKKALLKAVQSQHEEKEKAFKEQL

AHLASLLPTLQVHLVICSAFLSSANKAEFLDLGYQLMERLQRIVKLPHRLRPAQTSKINTEYRAEFARCL

EPLLVLTPRRSVVGSAGGIGPGITGANMIPGSQCSKTLMVPGCPPAGDKMSSSSIVRKPTLHRYISTKVL

LAEGRETPFAEHCRNYENTYRMLQTEIQGLKDQVQELHRDLTKHHSLIRTEIMSEILQKSLQMDVQIAAH

YSSVEMMRSVFEEVWEETYQRVANEQEIYEAQLHDLLQLRQENSCLTTITKQIAPYVRSIAKVKERLEPR

LQEPREPKDEHTQILLRIDDSSEAAQRDSSPGGTDSREQALGSRGGCTPALTPGDPPPKKEHRGSAQRSG

TLSADREEPPGSS

>chTRIM1_XP_004940642.3

METLESELTCPICLELFEDPLLLPCAHSLCFSCAHRILLSGCSSGESIEPLSAFQCPTCRYVISLNHRGL

EGLKRNVTLQNIIDRFQKASLSGPNSPSESRRDRPYRPNPSMSVGGERIACQFCEQDPPRDAVKTCITCE

VSYCDRCLRATHPNKKPFTSHRLVEPVPDAHFRGLTCLEHENEKVNMYCVADDQLICALCKLVGRHRDHQ

VASLSDRFEKLKQTLETNLTNLVKRNSELENQMAKLIQICQQVEVNTAMHEAKLMEECDELMEIIRQRKQ

VIAVKIKETKVMKLRKLAQQVANCRQCLERSTVLINQAEHILKENDHARFLQTARNVAERVAMATASSQV

LIPDINFNDAFENFALDFSREKKLLEGLDYLTAPNPPSVREELCTASHDTITVHWISEDEFSVSSYELQY

TIFTGQANFISLYNSMDSWMIVPNIKQNHYTVHGLQSGTRYIFLVKAINQAGSRNSEPARLKTNSQPFKL

DPKMAHKKLKISNDGLQMEKDESSLKKSHTPERFSGTGCYGAAGNVFIDSGCHYWEVVVGSSTWYAIGVA

YKSAPKNEWIGKNSSSWVFSRCNNNFVVRHNNKEMLVEVHPQMKRLGVLLDYDNNALSFYDPANSLHLHT

FEVSFILPVCPTFTIWNKSLMILSGLPAPDFIDYPEQQECNCRPQESPYVSGMKACH

>chTRIM210_NP_001376669.2

MGSPGSAQRKPVCPMGHPVLGGTASSSQSPPSQLTTTALPLTWASPCCVVCLADAMAEPQVLEGLEEELT

CSVCLDIYRNPVSLGCGHSFCEECIQGVLRSQHCPQGLFSCPLCSALEAPPTKLQPNIQLRSIVQKFLDT

SEQSAALEEELRRQVECEEKGESSYFHDEEILCDFCLENPQPAVKTCMNCETSLCQAHLGKHSTKAFLTS

HVLVEPCDARVLAERRCSQHGKLLECYCVTDSVCVCMLCCATSSHRSHEIISVEEAFSQAQNDFPKTLRT

LKKHKAAVNKSLENLLTQQEKIKALERLHRNHLENSFNAICEHLERRKTKILEAISDIEGKQLSQIQPWI

KEHKEMKDAVSSDVRELEALRDQKDPVLFIKGLAAIQARKREQVPNKDGVELAELLSIMDGSIKDTIQEF

FQPYIQLILDTKPSVESSPTHEHLTFKSCQAHGLHVDDKVLSVVELPSSLPVPHKEQFWVYSTRRFWNGQ

HFWEVITRNSLCWKVGVIDNSFQCYLEMSQRCLRVFLDKRQIKVQSLNTAIGMVRVELDCERNTVSFCDV

SGKDYPGSSWSFVPIATVPIPASYPVYAVFSISHGSLSLP

>chTRIM2_ NP_001244243.1

MASEGSNIPSPVVRQIDKQFLICSICLDRYKNPKVLPCLHTFCERCLQNYIPAHSLTLSCPVCRQTSILP

EKGVSALQNNFFITNLMDVLQRTPENSIEESSILETVTAVAAGKPLSCPNHDGNVMEFYCRSCETAMCRE

CTEGEHAEHPTVPLKDVVEQHKASLQVQLDSVNKRLPEIDSALHFISEIIHQLTNQKASIVDDIHSTFDE

LQKTLNVRKSVLLMELEVNYGLKHKVLQTQLDTLLEGQESIKSCSNFTAQALNHGTETEVLLVKKQMSDK

LNELAEQDFPLQPRENDQLDFIVETEGLKKSIHNLGTILTTNAVASETVATGEGLRQTVIGQPMSVTITT

KDKDGELCKSGNAYLTAELSTPDGSVADGEILDNKNGTYEFLYTVQKEGDFTLSLRLYDQHIKGSPFKLK

VVRSADVSPTTEGVKRRVKSPGSGHVKQKAVKRPASMYSTGKRKENPIEDDLIFRVGTKGRNKGEFTNLQ

GVAASTNGKILIADSNNQCVQIFSNDGHFKSRFGIRGRSPGQLQRPTGVAVHPSGDIIIADYDNKWVSIF

SSDGKFKAKIGSGKLMGPKGISVDRNGHIIVVDNKACCVFIFQPNGKIVTRFGSRGNGDKQFAGTLDGPH

FAAVNSNNEIIVTDFHNHSVKVFNQEGEFILKFGSNGEGNGQFNAPTGVAVDSNGNIIVADWGNSRIQVF

DGSGSFLSYINTSADPLYGPQGLALTSDGHVVVADSGNHCFKVYRYLQ

>chTRIM3_XP_004939021.1

MAKREANASPVVRQIDKQFLVCSICLDRYRNPKVLPCLHTFCERCLQNYIPPQSLTLSCPVCRQTSILPE

RGVAALQNNFFITNLMEVLQRDPDSRGPHPGPGLDPVSAVTGQPLSCPNHEGKVMEFYCESCETAMCREC

TEGEHREHVTVPLRDVVEQHKAALQQQLDAIRGRLPQLAAAIALVSEISQQLVERKNEAVSEIGSTFEEL

ERALQQRKGLLVRDLEAICGTKQKVLQAQLEVLRQGQENILSSCAFTEQALHHGTAPEVLLVRKQMSERL

SALASQEFPEHPHENDQLDYVVETDGVRKSILNLGVLITTSATAHKTVATGEGLRHAVVGQPASLSVTTK

DKDGELVRSGSACLRFVVTGPDGGAAESEVQDNKNGTYELLYTPRAEGDFQLSIALYGQPIRGSPFRVRA

VKASDVPPSPDDVKRRVKSPSSGHIRQKAVRRPSSMYSSGKKKENPIEDELIFRVGSRGREKGEFTNLQG

ISTSSTGRIVVADSNNQCVQVFSNEGQFRLRFGVRGRSPGQLQRPTGVTVDMNGDIIIADYDNRWVSIFS

PEGKFKTKIGAGRLLGPKGVAVDRNGHIIVVDNKACCVFIFQSNGKLVTKFGSRGTAERQFAGTLDGPHF

VAVNNKNEIVVTDFHNHSVKVYNADGEFLFKFGSHGEGNGQFNAPTGVAVDANGNIIVADWGNSRIQVFD

SAGSFLSYINTAADPLYGPQGLALTSDGHVVVADSGNHCFKAYRYLQ

>chTRIM7_NP_001385147.1

MQRAGRYGRTPLSYTSAPRWGAETRAAFGGAKSRDVSSLPFSSSRALCPKAEVFITSLRKPDTAVGSFLE

RERPWGRVPSIHALPPAPVKSVLARRREAMAAVFLPGNLQDEATCSVCLEFFKDPVSIECGHNFCRACIV

KSWKDLEMDFPCPQCREVFQQKSLRPNRQLANMSEIISQFALRGAKGAEEDGLCVKHREALKLYCKDDRR

SICVVCDRSREHRPHAVVPVDEASEEYKEKIQGRLDFLKKERQELLEFKVNDDKKTQELLKTIETERQKL

LVEFEGLRQFLHDQEHILLGQLDKMEKSIAKRQNENITDLSKEITLLNKLIAELEEKIQQPVLEFLKDVM

SIISRSDEVKCQKPVPVCTDMKMHVCNFSLKTVVLEKVLKRFKENLRDELGRGEKEDLTLDPDSANHLLI

LSADLKSVRMGCRKQELPDNPKRFDTNSRVLATTGFKSGRHYWEVEVGASDGWAFGVAKESVRRKGLTQF

SPEEGIWAVQQNGGRYWAVTSPQRTPLCLGQKLSKVRVYLDYEGEEVSFYNAENMQHIFTFNVAFREKVF

PLFSVCSTVTYIKLCS

>chTRIM201_NP_001092824.2

MEAPPAPPAVPPAPCSAARSLQDELTCPVCLEYFTDPVLVAECGHNFCRACVTQCWEDSARRLCCPQCRE

PVPQRLFRPNRSLGNIVHIVRQLGLPPGPADPPPGPPAPAPPLPVAAPPGPPGPPRCPRHGEPLRLFCVQ

DRRAVCVVCHLSREHRTHTVLPAEEAAHAAEEVPQEHLSSLRKGREEAKAERERQSEDLLKQTEVERQKI

VAECKELRGFLEEKEQLLLSRLEELDRDIVKRRDESVSRLSEEIAQLDKLLGEHGGENGPGSTAVTTAGS

SFESWMFCKPEPGFAELEKKLKSFSQKSAVLKEVLLEFKENLRFELENDTGDLSLDPDTANPYLVLSEDK

RSVRLRSAPQELPANPKRFDYSFCVLAAEGFVAGRHYWEVEVGDGESWVLGAARESVRRKEKIDFAPEEG

IWAVGLNWKGKNWDQYQAFTSPETPLSLCERPRKIGVYLDYEGGWVAFYNADNMAPIFTFTAAFTEKIFP

FFWLFYVGSSLSLCN

>chTRIM8_NP_001026404.2

MAENWKNCFEEELICPICLHVFVEPVQLPCKHNFCRGCIGEAWAKETGLVRCPECNQAYNQKPNLEKNLK

LTNIVEKFNSLNLEKPPSVLHCVFCRRGPPLPAQKICLRCEAPCCQSHVQTHLQQPSTARGHLLVEADDV

RAWSCPQHNAYRLYHCEAEQVAVCQFCCYYSGAHQGHSVCDVEIRRNEIRKMLMKQQDRLEEREQDIEEQ

LYKLESDKRLVEEKVSQLKDEVRLQYEKMHQILDEDLRKTMEILDKAQAKFCNENAAQVLHLNERMQEAK

KLLSSVQVMFDKTEDINFMKNTKSVKILMDRTQNCTGGSLPPPKIGHLNSKLFLNEIAKKEKQLRKLLEG

PLSTPVPFLQSIPMYPCTVSNSGAEKRKHSTAFPEGSFLEPSSGTVASQYMSQGASAGEGQSAKAMVPCS

STQHIVGFPSGPQPVHSGSVFNPSHYPNTTSSQQSVLSQYGGRKILVCSVDNCYCSSVSNHSGHQPYPRS

GHFPWTVSSQEYSHPLPPAPAVPQSLPGLAVRDWIDASQQHSRQDFYRVYGQPSAKHYVTS

>chTRIM9_XP_015132237.1

MEEMEEELKCPVCGSFYREPIILPCSHNLCQACARNILVQTPDSESPQSRRASGSAVSDYDYLDLDKMSL

YSEADSGYGSYGGFASAPTTPCQKSPNGVRVFPPAAPPPPAALAPPPPPPRNACLTCPQCHRSLVLDERG

LRGFPRNRLLEGVIDRYQQGRAAALRCQLCEKAPKEAAVMCEQCDVFYCEPCRLRCHPPRGPLAKHRLVP

PAQGRVSRRLSPRKISTCTDHELENHSMYCVQCKSPVCYQCLEEGKHSSHEVKALGAMWKLHKSQLSQAL

NGLSDRAKEAKEFLVQLRNMVQQIQENSVEFEACLVAQCDALIDALNRRKAQLLSRVNKEHEHKLKVVRD

QISHCTVKLRQTTGLMEYCLEVIKENDPSGFLQISDALIRRVHLTEDQWGKGTLTPRMTTDFDLNLDNGP

LLQSIHQLDFVQMKVSSPVPAPPILQLEECCTHNNSATLSWKQPPLSTVQVEGYILELDDGNGGQFREVY

VGKETMCTVDGLHFNSTYSARVKAFNKTGVSPYSKTLVLQTSEVAWFSFDPASAHADIIFSNDNLTVTCN

SYDDRVVLGKTGFSKGLHYWELSIDRYDNHPDPAFGVARIDVLKDAMLGKDDKAWAMYVDNNRSWFMHNN

SHTNRTEGGITKGATVGVLLDLTRRTLTFSINEDQQGPVAFENLEGLFFPAVSLNRNVQVTLHTGLPVPE

FYASRSAMQ

>chTRIM207_XP_015150487.1

MMASGKRNADLEEWDAKIRKLTEDFEGSDTELEECDTEDGDLAAEIDNLTAELEERDRKIRKLTSDLGDV

DTKLKERDRKITKLSAEIRRLTALLGDRDSKLRKLTAELAKCDATIRLFTVELGERHTKIGELTAEVGDY

NRKLRKHAAELEERDLKIREHEAEIRRLTELLEDRDSDVREQDILIRKLSEELEELRGREVEESDLEREE

HDARIDELSTEVEKRERRIDELTAELEHYKAKARRCFEEHRRGEEKLVNVTLDPETAHPRLVLSEDQKSV

RWEYSLQESPDGPERFDADPCVLGCETFTSGRHCWVVDLTEGQYCAVGVSRESLPRKGAVSFNPDEGIWA

VQQWGFKNRALTSPPTPLNLPRVPKKIRISLDYEWGEVAFFDVENQMPIFTFPLTSFGGERLRPWFWVEL

GSLSLPR

>chTRIM208_XP_040504620.1

MASAASRAKEKGEGGSHHPIPEHQMGNGCSSSVGRGGGTWQERGRLGDTEVISHRNHLRKGNFHTKLHLE

HLAEKLKLLGLDGDRGEKEKLCSWRKRTFPSNGPRDDGGPTGAPIKESVQEDVEQIHSDLENLKKQKEEL

LELKRSGERRCQDLLTRTEAERQKILSEFRQLRRFLKEKEMVLVARLGELDRAVLRRQEEEEAKVEGDIS

LLGILICEMEEKLKQPTRGFLQDVRSTLSRWERGRTRRMTENFADVERRLCVISQQHKILRETLGRFQDL

FLSELEKEEGASVGEEGKAFVTLDPTTATAGLVLSRDRRGVRWMDMGHNVSPCPQRFDVSCCVLGCRGFT

SGRHFWDVEVMGGATWALGVARSSVPRKGWLTFHPDYGIWAMGCCRNSFRAFTSPPSTLPISGDPNRIRV

VLDYGGGQVAFYVASQKNPIFAFQNASFCGESIFPFFWVGRGSHLRLCP

>chTRIM13_XP_015131811.2

MTLVLRDMMELLEEDLTCPICCSLFDDPRVLPCSHNFCRKCLEGILDGNVRNVLWRPSPFKCPTCRKETP

VTGVNSLQVNYSLKGIVEKYNKIKVTPKMPVCKVHSGQPLNIFCRTDMQLICGVCATRGDHTKHVFCSIE

EAYSQEKRAFETLFQGFETWRCGDALSRLDTLETSKRKALQMLTKDSDKVKEFFDKLQHTLEQKRNEILS

DFETMKLAVMQAYDPEINKLNSILQEQRMAFNIAEAFKDVSEPIVFLQQMQEFREKIRVLRETPLPCSNV

DITPTMKSFDTSQWNGIKLVDVDKLSLPQENNGLKLKIPSVFSRRFIVASLICLLILAITRMSFVESVVD

NFQCWKSQFFTISLSYLADTVEIADHAVFYWEQMTDGASLLSEKCKNYTLVVLDNVAQFVCKYKLL

>chTRIM14_NP_001026602.2

MAQGQPWVAAGPRGCAVHSGRPLELFCGDCGRCVCALCPALGPHRGHDARLLLQAARDKQELLTVYLRNL

EVRKEQQAGNRRSVEQAADDLKAHAATSKQQLLDRVTQLQLLLQEEERLARNVIDEKTQQALEAHGQQVQ

SCQEELAALEAFSRRIREIQQCSDPIQFLEKCTEIEKEMQDSRRQPEQWHPIPLSFEHILNHYSCLMKFL

QSILQKPLEVRLKEDVFGSLNATSKREPGTMLKTMSPVDRMLFLKHARSPTWEYDSLHPRLKLSDDRLVV

SCNWRRIFYPCGPQRFDKLWQVLSRDAFLSGSHYWEVDLLHAGAGWWIGAAYPSIGRKGDSESCRLGWNR

ASWCIKKFDLEYWAFHKGERIPLLIEDDPDRIGVFLDYEAGILSFYNVTDGMTHLHTFRCKFTEPVYPAL

RLWEGSIGIWKLT

>chTRIM18_NP_001384590.1

METLESELTCPICLELFEDPLLLPCAHSLCFNCAHRILVSHCASNEPVESITAFQCPTCRYVITLSQRGL

EGLKRNVTLQNIIDRFQKASVSGPNSPSETRRERAFDSNSMSSCEKVLCQFCDQDPAQEAVKTCVTCEVS

YCEECLKATHPNKKPFTGHRLIEPIPDSHIRGLMCLEHEDEKVNMYCVTDDQLICALCKLVGRHRDHQVA

ALSERYDKLKQNLESNLTNLIKRNTELETLLAKLIQTCQHVEVNASRQETKLMEECDQLIEIIQQRRQII

GTKIKEGKVVRLRKLAQQIANCKQCIERSTSLISQAEQSLKENDHARFLQTAKNITERVSMATASSQVLI

PEINLNDTFDTFALDFTREKKLLECLDYLTAPNPPTIREELCTASYDTITVHWTSDDEFSVVSYELQYTI

FTGQANVVSLCNSADSWMIVPNIKQNHYTVHGLQSGTKYIFIVKAINQAGSRSSEPGKLKTNSQPFKLDP

KSAHRKLKVSHDNLTVERDETSSKKSHTPERFTSQGSYGVAGNVFIDSGRHYWEVVISGSTWYAIGISYK

SAPKHEWIGKNSASWVLCRCNNTWVVRHNSKEIPIEPAPHLRRVGILLDYDNGSLAFYDALNSLHLYTFD

ITFGQPVCPTFTVWNKCLTIITGLPIPDHLDSSEQLA

>chTRIM19.1_XP_015134517.2

MPGSPEAPRAPGPQEDGSAAPKEPGAAPGPSQCPAPSGPPGEGDFQFVLCEGCRQESPNLKLLTCLHTLC

LGCLRENKPVGQCPVCQAPIPQPDGIPDVDNVLFSSLQTRLSIYRRISRGGLSCSRCRQDAAAVWCSECE

EFLCPGCFEDHQWFFKKRSHEARKVEELRAESAHRFLEGTKRSCNLFCSSPGHTEQGHVTSIFCRKCEKP

LCCSCALLDAQHSSFYCDIRTEIQRRQDELAVLGQELARRRGGFEASHAALQEKAARLEAEGRGMRELVR

QHVKQLVELIRREEEKLLELVEKRQERGRRELEGELRRVETVLRRMEAGERLVEKMGLYATEQEVMDMQP

FVKDALEELRRQRPAADVELVLHEDFAECRARLQELAERIEGLQGGAPRPVPVVEVALENNLEEEPPQHS

SQDVLPTFTISLAKMGVSQAATRPKRCQPHVERSSQVSPKLLKLERDGGEPSSSQWDGRGGPSTSAQSHN

GRIPPSKASRSRTRDAEDSSIIISSEDSEEDMVTSDFTTC

>chTRIM19.2_XP_040562550.1

MAGQEPAVPPLEDDFQFMLCEGCRQESPDLKLLTCLHTLCLGCLRENKPVGQCPVCQAPIPQPDGIPNVD

NVLFSSLQTRLSIYRRIVSGAELLCDNCGSSSEYWCPECKEFLCTKCFETHQRYVKRENHEAKRVQDIRV

GSPQEFLEGTRRTSSSSCPIPTHQNQTLSIYCKQCCRAVCCICALLDAAHTGQHCDISTEAQRRQDELAV

LGQELARRRGGFEASHAALQEKAARLEAEGRGMRELVRQHVEQLVELIRREEEKLLELVEKRQERGRREL

EGELRRVEAVLRRMEAGERLVEKMGLYATEQEVMDMQPFVKDVLEELRRQRPAADVELVLHEDFAECRAR

LQELAERIEGLQEAAPAPATTENSHQVRFAFCSHRKPSQIPGLPTTSPSTPVSSVHCLHLCPLPQVPLTS

TPAKRKKDEGTTTMPFPPKVIKVEKDDDEWSTPVVPQEPNCLPQPQISFSPPTMTASREGSSNERHDSDS

DLLNLGSAEEDSMDEELLDPSLLDDMLEDGTSRDNSHLPICLKNTLDVGQGSVIFFDVEIWKNEVIQMSV

VDKEKMLSVIIQPMMCLPTSTAITNSYEMGLRDLLSHLSTIRRPILAAFDLWSLPFPTLLKSLMAIGKKE

EFFSMVYGFLDVLPLIKEKIPMKESCKLKNLASNLLWRDLRSSSTLESAKATKDLCNVLEIDLKSKPRQL

LSLANLESFMSLQPLLDKRLLSRPSAQTLASHGIGLPELHACFACDPIRGLPKMCAVVNAHRHPSEKKVR

HLSKVKAYFQQQPLAGSSHALVHDELLEAMKTKQDC

>chTRIM23_XP_424752.3

MAALVVNKAGAVPDSSRGASNSRGPTGAAVKVLECGVCEDVFSLQGDKVPRLLLCGHTVCHDCLTRLPLH

GRAVRCPFDRQVTELGDSGVWGLKKNFALLELLERLQNGPPGQCGTAEEAIGLSGESIIRCDEDEAHVAS

VYCTVCATHLCADCSQLTHSTKTLAKHKRVPLADKPHEKTMCSQHQVHAIEFVCLEEDCQASPLMCCVCK

EYGKHQGHKHSVLEPEANQIRASILDMAHCIRTFTEEISDYSRKLVGIVQHIEGGEQIVEDGVGMAHTEH

VPGTAENARSCVRAYFSDLHETLCRQEEMALSVVDAHVREKLIWLRQQQEDMTILLSQVSTACLHCEKTL

QQDDCRVVLAKQEITRLLETLQKQQQQFTELADHVQLDASIPVTFTKDNRVHIGPKMEIRVVTLGLDGAG

KTTILFKLKQDEFMQPIPTIGFNVETVEYKNLKFTIWDVGGKHKLRPLWKHYYLNTQAVVFVVDSSHRDR

VSEAHSELAKLLTEKELRDALLLIFANKQDVAGALSVEEITELLSLHKLCCGRNWYIQGCDARSGTGLFE

GLDWLSRQLVAAGVLDVA

>chTRIM24_XP_015143032.2

MEEATATVAATAAAAGGGPCPVDRTGAAALSGENEAESRQGPAERGGEAAPLNLLDTCGVCGQPIQSRQP

KLLPCLHSVCQRCLPQPDRYLMLPPAAPTKEPPPPPASPGPSPLHCTPVGVIRCPICGQECAERHIIDNF

FVKDTTEVPSSTVEKSNQVCTSCEDNAEAHGFCVECVEWLCKTCIRAHQRVKFTKDHTVRQKEEVSPEAV

GVTSQRPVFCPFHKKEQLKLYCETCDKLTCRDCQLLEHKEHRYQFIEEAFQNQKVIIETLITKLMEKTKY

IKCTGKQIQNRILEMNQNQKQVEQDIKVAIFTLMVEINKKGKALLHQLETLAKEHRMKLMQQQLEVAGLS

KQLEHAMNFSKWAVSSGSSTALLYSKRLITYRLRYLLRARCDASPVTNNTIQFHCDPSFWAQNIFNLGSL

VIEDKETPPHMPKNPVTETNLQPPGSVPSNQLSKFPMQINLAQLRLQHMQQQVMAQRQQAQRRGGPVGLP

NPRMPGTVQQPPASHQAPPRLIHFQNHSPKSSGAAPPAQQMRFPQNQNLPRHAIKLNPLQMAFLAQQAIK

QWQVGNGQSSTAITTASSITSTPSSPTVTSAAGCDGKTYGPPVIDLSSPVGSSYNLPSLPDIDCSGNITL

DTVARKDDGTAEQNHPKPPSNRTVQSPNSSVPSPGISGGVSVTNIHPPVRSPSASSVGSRESSSSSSRPP

GADSTHKVPVVMLEPIRIKQESSTPNENFDFPIVIVKQEAEEESRPRNTSFSRSILTSLLLDGNHSSNSD

EAVLRTDAPDSTDDQPRILQENTTSAKMGWLGPSHTGEGRKEDDPNEDWCAVCQNGGELLCCEKCPKVFH

LSCHVPSLMSFPSGEWICTFCRDLSKPEVEYDCDKPTHNPEKRKLEDTVGLAPIDRRKCERLLLYLYCHE

MSLAFQDPVPPTVPDYYKIIKKPMDLSTIKKRLQVTNSFYTKPEDFVADFRLIFQNCAEFNEPDSEVADA

GMKLEAYFEELLKSLYPGRKFPVQSNCQSEKDNPELSDDSDDDIVQPRKKRLKGEDRQLLK

>chTRIM25_NP_001305387.1

MATLTKAQSEPNLLALEEDLTCSICLSIFDAPVTVPCGHNFCAYCLEQTWASQVRDFSCPQCRTTFPDRP

QLHKNTVLCRVVEQLQGCGDGKEDDEHEEEEQEAAAPIYCDSCLQAAATQTCLTCMASFCPEHLRPHYDS

PAFRHHQLCPPVRDLQQRKCPQHNKLFEFFCSQHSCCICSLCLLSHKLCHTSPLQQAKDNAESALKKRLA

ELHNQSERSVQAMNSVKTIQRQAAETAARKRDLLRAEFLEIKALIEEKENQTLKVFMEEEKRVCNKFDYV

YKILGNKKNEIQSLRDQIEMALTEGDDVLFLKRAAALQRTSIKEAFVPVIEMDNNMIHAAYQSAINLKDV

VKLAVNVSVDKRTDAKPPPGKTKPPSVPSPNKPVVKKKLAADSRTHKDKIPHPTKTTLLEETDTQDKRNP

VKLAPSMGAPSMGAPSAANAAKVKELISSFLKKPRAELLQYAANVTLDFNTAHSKVALSERYTKMSVSDT

QLNYNHHPQRFTDCPQVLGFQCFKRGIHYWEVEMQQNNFCGIGICYGSMERQGPDSRLGRNSSSWCIEWF

NSKISAWHNDVEKCLPNTKATKIGVLLHCDGGFVLFLTVEEKLNLIYKFKAQFTEAVYPAFWLFSSGTVL

SLW

>chTRIM211_NP_001376668.2

MAKAKEEVRASSSLEDELTCSICLSLYKNPVSLCCGHSFCKQCVQKVLSNQQQAKASYSCPLCRVDLGPI

LELQNNFHLCSIVETYLANKGKKDKGFATKKEEAVPCDFCLDKPQPAVKMCLSCEASLCQAHLDKHSARA

SQKDHVLVEVGMGGPIEERRCQEHGKAFEFFCQNESLFICALCSIAGCHNGHKILTLREAHSMKLDDLSH

TVDRLLRYRSDVSIALEELQQNKNQIETNTKTVTSLVQKKFEEIKTEINKKEKKILSDIQSIEKLQLAEC

DKLRKEMEEQRDELVQHLKSLQKIREQPNTFCLFREFKLVQKSIPSHKFRIRKKDVQMVLLDEAAISSFQ

TMMGDCLLQLDMYLQGVQRQIINQFKWDSEDPAAGSNSNNTFKKSQQKPWPNFY

>chTRIM205_NP_001025842.2

MATQSPLDSLQSEASCSICLGYFQDPVSIPCGHNFCRECIARCWEGLEGNFPCPQCRKTALHKNFRPSRE

LANIASIARQLSLHSSGWCKQHREALKLFCTEDQEPICAQCDASQAHRSHAVLPAHQAAQEYKEEIQARL

QASKEEREKYLESRRTGGRTGLYLEKTKKEGKRIVCEFEQLQRFLKEKERFLLVQLADLDRAVTKVQEDA

VTKVMEEMSHLDTLIWEMEGKFQQPDSKFLLDIRRLLNSCEMMKFSPPTEISPTLERRLEDFLQKNVLVR

LTLQKCQDSLMFQLQEPANVTMDPTTAHPNLHLSEDQKQARGQLVPQDFPENPERFSFEPCVLGCQGFTS

GRHFWEVEVGQGGVWALGVARASMKRKGPMSFSPKEGVWALEAYHSLTSPRANLRLNALPRRIRVSLDYE

GGRVAFFSSDDDTPILVYTRALFNGEKVLPLFKIGLGARLQEITQNPPSEEQSTSGQLMSHLDWVGFRSP

LKICP

>chTRIM203_NP_001092829.1

MAASNPTPLLPSEASCPICLEYFRDPVSIHCGHNFCRQCITRCWEWSTGGFCCPQCKETAEERVLCPNRE

LARVLEIARRLSLQAAHRDAAGQEGCEKHREPLSIYCKDDEAFICVICRESRLHRAHAMLPVQDAVQEYK

EQIQSHLQALKEDRDKLLGFREAEMRRNWEYLEKTSAERQKILGGFEGLRLFLEEQEHHLLAQLENMERD

VEKTQEENVTILTKEISHLDTIIQEMEEKCQQPASKFLQDIRSTLSRLGKENFQQPTLLLPDLESNLSHF

REKNNALEEILKNFKEILMFELPEKMSVTLDPSTAHPQLTVSEDGRSVRWEDTQRDAASEEFGTDPFVLG

HEGITSGRCCWEVEVTPKGSWAVGVAKESLKRREESGMSSEIELWSMGFCEGQFWALSSFERMTLPQIQV

PRRVRVTLDYERGQVAFFDADKRALIFIFPSASFKGESIHPWFLVWGEGSQITLCP

>chTRIM28_NP_001305930.2

MSAPAEEAAEAVGPAEKRPDLVDLLERCGSCRVLLQAEREPRLLPCLHSVCRQCLRTSPGPTADSGPGGQ

VVDCPICKHQCPLQDVVENYFLKDKGAKMAGDNLASTQCCTSCEDNAPATSFCVECSEPLCDTCVEAHQR

VKYTKDHTVRAAANTSVKEVDHTVYCSVHKQEPLMIFCDTCDTLTCRDCQLNAHKDHQYQFLEDAVKNQR

KMLATLVKRLGDKHASLQHSTKEVCSLIRQVSDVQKQVQVDVKTAILCIMRELNKRGRVLVSDAQKVTEG

QQEKLERQHWAMTKLQRHQEHVMRFTSWALESENSTALLLSKKLIHFQLQRALKMIVDPVKPQGDMKFQW

DPNAWTKSAESFGTIVSDNGLLPPALSPQPPAASPSSGPSQGSLKATVVSKGQCAPSPLLQSPRAAQPED

KEGQETPGTPQAGEDGVETPNTPQGPLGTEGPDLSPPCLEPQVSKTSKDANVELAGNASPDRAATGAKRR

RHANPPDQEVEEKFVPKLLVKRSQPSGGPTLRKVPRVSLERLDLDLSGEAQPPVFRVFPGTSAEEFSLIV

IERGAQPQPAPPPPPAIKEEQKELSIGDAEDTKPVVLPPKPPSGVVTGPEIPPAPTVAPTGTHRNVSCCR

VCCQAGAVVMCDLCERCYHLDCHLPVLHVVPSHDWRCLLCQDLPPQTEGLTNGGPKEGQLPTLCPTDQQK

CERVLLELLCHEPCRPLHRLSSSTESTDAIDLTLIRAKLQGKLSPGYTSPEDFTADIGRMIQQFNRLTED

KADVQSILGVQRFFQERLSAAFRDRNFSTLLDPTVPMEGAEGAAAAPPAPLPAPPVAP

>chTRIM29_XP_015153673.1

METGSAARTNGTAGKPEDVKSPSAPKKDEEVKKTTNPGGGEKEPIKGTGGTSLETGQIKSSLFSGSDWKR

PIIQFVESSDEKRSTYFSMDSADSKKMPFASGQIGDMRRTPLSFADKGDLRKSLFSLDSKKSFLPNEGEG

RKPLFSSGQIGDMKKPSLPLVETGDLRRPTFNKVPDRAAGSRPRVKLEDVLCDSCIDNKQKAVKSCLVCQ

ASFCELHLKPHLEGAAFRDHQLLDPIRDFEARKCPVHGKTMELFCQTDQMCICYLCMFQEHKNHSTVTVE

IEKAGKEAELSLQKEQLQLKIIEVEDEVDKWQKERDRIKNYTTNEKATVDQHFKELIRDLERQRDEVKAA

LDQREKIASENVKEIVDELEERAKLLREDKENREQIHQISDSVLFLQEFGALMRNYVPPPSLPTYSVLLE

GESMSPSMGLLRDDLLNVCMRHVEKICKADLGRNFIERNHMENGDHRYMMNNYEWNQPDNLKRFSMFLSP

KASFNPRSWEFSSFQATEETLGNGTKLPFQFSSVGQNPPGDFSKQSDGSLFTKTAYPSIVRHQSAKVTPQ

TWKSSKQSVLSHYRPFYVNKGNGATSNEAP

>chTRIM32_XP_040505171.1

MASAPQLNSDALREVLECPICMESFTEEHLRPKLLHCGHTICKQCLEKLLATSINGIRCPFCSKITRIKT

LAQLTDNLTVLKIIDTTGLGEVVGLLMCKVCGRRLPRHFCKSCGLVLCEPCKETSHMPQGHNVIAIKEAA

EERRREFGTRLARLRELMDELQKRKASLEGVSSDLQSRYRAVLQDYSKEERKIQEELARSRKFFTTSLSE

VEKVNNQVMEEQAYLLNLAEVQILSRCDYFLAKIKQGDIALLEEAADEEEPELTSSLPGELILHEVELLK

VSHVGPLQIGQVVKKPRTVNLEESLMETAASSSASFREPDLVQEEASCTPNTSPAKPRMPEAAASIQQCH

FIKKMGSKGSLPGMFNLPVSLYVTLQGEVLVADRGNFRIQVFTRKGFLKEIRRSPSGIDSFVLSFLGADL

PNLTPLSVTMNCHGLIGVTDSYDNSVKVYTMDGHCVACHRSQLSKPWGIAALPSGQFVVTDVEGGKLWCF

TVDRGVGVVKYTCLCSAVRPKFVTCDAEGTIYFTQGLGLNLENRQYEHHLEGGFSIGSVGPDGQLGRQIS

HFFSENEDFRCIAGMCVDARGDLIVADSSRKEILHFPKGGGYNILIREGLTCPVGIAITPKGQLLVLDCW

DHCIKIYSYHLRRYSTP

>chTRIM33_NP_001366055.1

MAENKGGGGGGEGAAEAGPGGGGGLEPTAASPSGAAPPAPSAAPLEERESPGAAAAAAAERGLGEAEAEA

GPGVVPGPGPSPVPPLTPAAPGPFSLLDTCAVCAQSLQSRREAEPKLLPCLHSFCRRCLPEPERQLSVPV

PGGANGDVQQVGVIRCPICRQECRQIDLVDNYFVKDTSETPSSSDEKSEQVCTSCEDNASAVGFCVECGE

WLCKTCIEAHQRVKFTKDHMIRKKEDVSSEAVGASGQRPVFCPVHKQEQLKLFCETCDRLTCRDCQLLEH

KEHRYQFLEEAFQNQKGAIENLLAKLLEKKNYVNFAATQVQNRIKEVNETNKRVEQEIKVAIFTLINEIN

KKGKSLLQHLENVTKERQMKLIQQQNDITGLSRQVKHVMNFTNWAIASGSSTALLYSKRLITFQLRHILK

ARCDPVPAANGAIRFHCDPTFWAKNVVNLGNLVIENKPAPSYTPNVVVGQAPPGTNHINKAPGQINLAQL

RLQHMQQQVYAQKHQQLQQMRMGQPSGSVPRQTNPQVLQQQPPRLISMQTMQRGNMNCGAFQAHQMRMAQ

NAARIPGIPRHNGPQYSMMQPHLQRQHSNPGHAGPFPVVSVHNNTINPTSPTTATMASANRGPTSPSVTA

IELIPSVTNPENLPSLPDIPPIQLEDAGSSSLDNLLSRYITGSHLPPQPTSTMNPSPGPSALSPGSSGLS

NSHTPVRPPSTSSTGSRGSCGSSGRTAEKTSVNFKSDQVKVKQEPGTEEEICSFSGAVKQEKTEDGRRSA

CMLSSPESSLTPPLSTNLHLESELEALGSLENHVKTEPGDLSESCKQSGHGLLNGKSPMRSLMHRSARTG

GEGTNKDDDPNEDWCAVCQNGGDLLCCEKCPKVFHLTCHVPTLLSFPSGDWICTFCRDLSKPEVEYDCDN

LQHSKKGKTAQGLSPVDQRKCERLLLYLYCHELSIEFQEPVPASIPNYYKIIKKPMDLSTVKKKLQKKHS

QHYQTPEDFVADVRLIFKNCERFNEADSEVAQAGKAVALYFEDKLTEIYPDRTFQPLPEFEQEEDDGEIT

EDSDEDFIQPRRKRLKSDERPVHIK

>chTRIM35_XP_004935921.1

MLPAAARGAHRPARRRARCKRCHPSASSPPSAMENEANSPSSSLAALLSPSTAPFKEELLCPICYEPFRE

AVTLCCGHNFCKGCISRSWEHRQHTCPVCKETSSFDDLRINHTLNNLVEMILREEGRRLGRPAALCPLHH

EEANLFCLDDKELACFACQGSKQHEGHKMRPVQETAMDFRACEGPEGVSPPQAKLKNMETSLQDKVKDFG

AVHRSYESISKHNKVEAKRLEEQIKREFEKLHEFLRDEEKALLSQLQEEKQHKHSLIESKMKQLAENSQA

VRSEARQLQADLEEDDYTFLKTHKNRKRRIACTAEEPEAVPLGMLIDSAKYLGSLQYNVWKKMLNIITVV

PFSFNPNSAAGWLSVSEDLTSVTNGSYRLLVENPERFTSAPCILGSRGFSSGFHTWEVDLGGITNWRVGV

AQLRGGIHWTFHHDARSGFWYIYRLPGMNSKMCQASNMARTVVALGELVRIRVELDCDEGELSFYNADCK

THIFTFHEKFGGTVFPYFYVGGTPVGATPEALRICPLRVRIHEDVPV

>chTRIM36_XP_040512100.1

MEGDGLEPEVATKSIERELICPACNELFTHPLILPCQHNLCHKCVKDILFTVEDSFADAGSESSNQSSPR

FRISSASMDKIDRISRSGRKRNSLTPRTTMFPCPGCLHDIDLGERGVNGLFRNFTLETIVERHRQAARAA

IAIMCDFCKPPPQESTKSCMDCSASYCNECFKVHHPWGTVKAQHEYVGPTTNIRLKVLMCPEHEMERVNM

YCEICRRPVCHLCKLGGSHANHRVTMMSTAYKTLKEKLSKDIEYLISKESQVKAHISQLGLLLKETESNG

ERAKEEASQSFEKLINILEEKKSAALRAIEVSKNLRLDKLRTQAEEYQGLLENSGLVGYAQEVLKETDQS

CFVQTAKQLHVRIQKATESLKSFRPAAESSFEDFVVDTAKQEVILDDLSFYSNGLEIPEINEEQCRMYNK

AVISWESSGKTDSADMYVLQYRKRNREEESVMWQEAEVYSKSKVISDLDDDSSYAFRVRGYKGSICSSWS

KEVILRTPPAPVFSFLFDDKCGYNSERLLLNPERTTVESRAGFPLLLRAERMQFGCYTSLNYIIGDTGIA

KGKHFWAFRVEAYSYLVKVGVVSSTKIQKFFHNTHDVTSPRSEQDSGHDSGSEDACLDSSQPFTLVTLGM

KKFFIPKAAADTRAAADPKDPASRILPLPSCMGICLDCDNGKVGFYDASHMKCLYECEVECSGIMYPTFA

LMGGAAIHLMEAIPAKYLEYQDDL

>chTRIM37_NP_001006224.2

MDEQSVESIAEVFRCFICMEKLRDARLCPHCSKLCCFSCIRRWLTEQRAQCPHCRAPLQLRELVNCRWAE

EVTQQLDTLQLCNLTKHEENEKDKCENHHEKLSVFCWTCKKCICHQCALWGGMHGGHTFKPLAEIYEQHV

TKVNEEVAKLRRRLMELISLVQEVERNVEAVRSAKDERVREIRNAVEMMIARLDTQLKNKLITLMGQKTS

LTQETELLESLLQEVEHQLRSCSKSELISKSSEILMMFQQVHRKPMASFVTTPVPPDFTSELVPAYDSTT

FVLENFSTLRQRADPVYSPPLQVSGLCWRLKVYPDGNGVVRGYYLSVFLELSAGLPETSKYEYRVEMVHQ

STNDPTKNIIREFASDFEVGECWGYNRFFRLDLLANEGYLNRQNDTVILRFQVRSPTFFQKCRDQHWYIA

QLEAAQTSYIQQINNLKERLAIELSRTQKSRGISPPDTHLSPQNDDGPETRSKKSGPSTEALLESVAAPG

LVRDNKDEDEEKIQHEDFNHELSDGDLDVDLAGEDEVNHLDGSSSSASSTATSNTEENDIDEETMSGEND

VEYSNNMELEEGDLMEDAAAAAAPGASGTSHGYTSASGRPSRRGGSALGSAASSSLLDIDPLILIHLLDL

KDRNGMENLWGLQPRPPASILQSRASSYSLKDRDLRRHQAMWRVPPDLKMLKRLKTQMAEVRSKMSDVKN

QLSEVRSSNAGSCDGQPNFFSIEQGALAACGTDGCSKLQEIGMELLTKSSVTSCYIRNSASKKRDSPKPI

RSGAAGSLSLRRAMDCGDNNLRLKGDGQTCEGGAGSSKSSSRHHCPRPLASSNASEALPKPEERPCEGSD

SDVGVSGLNGLAAVEKTRKAGALGSNPKGYRTEGTQSGGLEDSSETGELQGVLPEGASAGPEEGGSCQKH

VLHLLPGMSSDSDIECDTENEEQEDATSTSEGFNHAFSVQSSSEASERCSAFPGGDQVGPDDLSFVNGED

TTR

>chTRIM202_NP_001006196.3

MDEDNPAESFQDEASCSLCLGFFQDPVSIHCGHNFCRACITRCWEEQEETFSCPRCKATATQRNLRPNRE

LAKIIEIAKRLSLRAPSAGERLCERHREVLKLFCEEDQVPICLVCRESHAHRLHAAVPIEEAVEEQKEKI

QAHVQILKEKKEKLQGLKEAEEGKSLEFLEKVQQERQKVVLDIKELQQFVEQQERLLLGRLEKLDQEIVR

RKEENLAKLLEEISSVSEQIRELEEKCQQPPCEFLQDSRNILSRLENEKSQQAPEILPGAEENPASPPQK

NTALKETLMQFRESLTLDPDTAHPRLVLSEDHRCVRWEEARHPVPDNPKRFDSSRCVLGCQGFNAGRHYW

EVEVGDGEAWAVGVAKESVERKGRISVKPEVGIWAVGQCGAQYQALTSPTSPITLHSAPKVIGIYLDYEA

GRVAFFDANNEVPIFAYPPTSFGGERVLPLLCLGRGCRFTLSP

>chTRIM204_NP_001299812.1

MALFSRHVYLAMGLNTRTVGQKWLEMFPRELRAKALEDLKNEIEKRQKSTAKHSEEISPLDTKAQGEKSQ

QSDVKSPITRCGKVTFQLPAGASPGPEERLCHSSWRNGALKETLKRLQASITLDPDTAHPDLILSEDRKS

VKRGEGQQDLPDNPERFAYWPFVLGHQSFSAGRHCWEVEVGDEGDWAIGVARESIPRKGQLSLCPKGGIW

GVEKWGGQVRALTTHKVTLLALRWVPRRVSVHLDYAGGTVAFFDADEGGLIFVFSHASFSGERVHPWLWV

VGVRSKLRLCP

>chTRIM41_NP_001025843.1

MEAVGDDGASSGRLNPVETLQEEAICAICLDYFVEPVSIGCGHNFCRVCIAQLWGGGEAEVEESGGAAAL

EEEEEELEEEEEDELGEEELDVEQEEEEEDGGGEEEEDDMWSEEEEDGELWEDPVEEELWDGVVQGELYF

GDDDYDEDVMEEDVEEEEEEEDEAQSPPPPVLPARPRRLQTFTCPQCRKTFFQRNFRPNLQLANMVQIIR

QLHPHPQRLAPPAGPSASGGPGGNPGILVATGGRGCPNLCEKHQEPLKLFCEVDEQAICVVCRESRSHKH

HSVVPLEEVVQDYKYKLQSHLEPLKKKLDAVLKQKSNEQEKITELREKMKLEIKEFESDFELLHQFLIGE

HVLLLHQLEERYESLLARQSSNISQLEEQSAALSRLITEAEDKSKQDGLQLLKDIKGTFIRCENIKFQEP

EMVLVDVGKKYRNYFLQDVVMRKMEKAFSKVPQADITLDPDTAHPRLSLSLDRRSVKLGERRQELPNNPK

RFDSDYCVLGSQGFTTGRHYWEVEVGGKKGWAVGAARETARRKEKTMGPHQKREIWCVGTNGKKYQALTA

MEQMALSPSERPRRFGVYLDYERGQLCFYNAESMTHIHTFNASFHERIFPFFRILAKGTRIKICT

>chTRIM212_XP_001232970.5

MEGRSPQGRPTGATLRPRLPLPRLGVIIQGHTQCSCQAAAPTMALSEALERLDEEAICSICLEYMSEPVS

VDCGHNFCRSCIGKHCQEKGLWNDTPFSCPQCRAPCRRSSLRPNRQLANIVESIRQLGLRGSVGTATETG

AGTLLCPLHNERLKLFCEVDEQPICVVCRESQQHRSHTVYPIEEAAQGYKVKLQKCLEQLSKEVEDMKKR

ESEAKMKTQECKEKVKRKRETIVCEFGKLHQLLADEEKLLLQKLEEEETQILVMISENLARVTEQKCSLV

ELILEIKEKMQQPVEVLLKDRRCILNRCEMMKFQAPKPVSVNLKEDYSIPERCMGMRHMLRKFKVDVTLD

PETANLELILSEDRKSVRRGSRKLFLLLFDSPKRFNCAPVVLGLQAFFSGRSYWEVQVGDKPEWGLGLCK

EAAGRRGNIVFSPQNGYWVLRLQNGGYEALTCPVSCLTLRVRPRCIGIFLDYEAGEISFYNVSDRSHLYT

FTDKFSGKLRPFFYLGSLMGGKNAEPLVISWMRDTQGTSCVVL

>chTRIM42_XP_422632.5

MDEDGCTFSACPCFSNCCYLTCHRRKEKCCLCWRFLFTSEQNCSCFPCPYEEDKPYQCCHCSCSEHANCW

WCCCSCSNDPDCKCCCCGGENSACQYYASKCGRNSICEPQQSTAPETVQSKDVTSRLRTRNNACISIPER

KGSNQAFRDQLACPLCKQLFLQPFMLPCNHCICEKCILKSKTKAEATGNYYIIICPVCNKAHCLPYTNKI

QLRKNYLKAKLAKKYMRRHGILKWRFDHSVRPVYCETCRERRRVATKRCRTCGINMCSECLHLYHTESGA

QDHIFTNTCQEDNEQWACLLHCNSHLSEYCLDDHKLICGFCKNSLHNDHETIPLAAACSRQAASLSNTIV

KFKQARQGVDNDLMEVILLKNNFQASKDLQRKEIRNGFLKLHTVLHEQEKEMMELLENTELKKQKDISEY

VNYTFSQLSYMDGLIQYAEEALKEESQVVFLQSAHCLVKEIEDAIPSIFQPSPLIREDPLRKLQFKFDEL

FAILQGFSPSLCETKQLESKAEKYPCSFNPEIMVPRHVSSVHEAKQSTVLQSASLNSLFELGMPDESTLG

KTNSTPPHHSTESNKMCEFWDAACETSRKEKKCQIVNFPSSEPMEKESFPVPGPVVIYQTVVYPRSAKIY

WTCPVEDVDFFEVEFYEVVGIGPDNTVQTQLDGKLSKILQQNLEIHNLDSNTEYLFKVRAVNKSGQGEWS

ESCKIITSGEHKIIQDRWRTQ

>chTRIM45_XP_040516325.1

MAASRCPVCAEPCVEPRLLPCLHSLCAPCLRRLGTLGEPGRAAAALSVLCPLCDAEVALPPGGLGQLVPD

YTALGRGGAAGTAGCDLCADGAAARRCQTCGAHLCLFCCQAHRRQKKTASHVVMELESTKDCSQAGKPFF

CPSHPSEELGLFCEQCDQPVCQDCVAERHRQHPYDFTSNAVHRHGDSLRELLRSTQQRMSALEDVLGQTD

GVGSAIRARAEAVATEICLFARGYVKAIEEHRDRLLKQLEDLKVQKENLLHLQKAQLQQLLLDMRTGVEF

TERLLTSGSDLGILVTKGVVASRLAKLNSAAYSTHPSVDDSIQFSPQERAGQCHGYEVFGAVICKAVDPA

KCTLQGEGLRSARQNELTGFTLLCSDTTGERMGRGGEAVVVTITHKDKKDCAVKPTVCDNGDGTYHISYS

PEEPGLYAVCVYVKGQHVQGSPFIVMVKSQFRKHQGMFHCCTFCSSGGQKAARCACGGTMPGGYQGCGHG

HKGHPGCPHWSCCGQVKMSSECLSGLPSDRSQRSLLRTVEL

>chTRIM47_XP_040542204.1

MEGAPGRAPPVPSAATAAALRLALAVPGLPDGPFGCPICLDLLKDPVTVPCGHNFCQGCLGKLRGRTGPP

DGAAGGAARCPLCQEPFPAALRLSKNRALCEVLPLLGAAGAGTSPDGARTPPAIPSPQSGPGSPPGSAGF

SGTGPSGAESSPSEMAPGAEEEPGGEAELEEGAAAVLCDVCPEGDRAAAARSCLVCLASFCGAHLEPHRR

SPAFRSHRLVAPLRRLEEGLCPRHLQPLDGFCRAEQTCVCVRCRANEHRGHDVVSLEKERELKEAQRAKF

LSSVDNELEELAVTIAQTRKMVELIKGVASKEKERVEKLFAEASELLATFQKEVAAFIEDSERSMLGETE

ADLRWKEERRAKLAQGKQNLENIPSTDTIYFLQEFQALKIAMEDNLSPAPSFQNELNFTKCSQAVCAVKD

ALSAARKSQWDCLQGKGVDGMSFPKTEEAMTKSQFPDKSNNPACLESRDYFLKFAFIIDLDSDTADKFIQ

LFGTKGAKRVLCPIPYPESPTRFINCEQVLGVNLMNRGNYYWEVELIDGWVSIGVITEVFNPQDSYNHSR

LGRNTSSCCLQWNGQNYAAWFGGLESVIQQPFFHTIGVFLEYSEKALTFYGVKDSKMTCLKQLKASSFAK

AQSDPFQHKINQQFASLFSFKLKPAFFLESVDAHLQIGPLKKDCVSVLKRR

>chTRIM50_XP_015151390.2

MARKMSIDNLEDQLLCPICLEVFKEPLMLQCGHSYCKSCVLSLSGELDEQLLCPVCRKSVDCSASPPNVT

LARIIEALQSRGETEPTPESCPTHDNPLSLFCEADREVICGLCGTIGNHKQHKITPISTAYCRMKEELSV

LLTDVHRCKRNLDELFSKLINNKTRIANEADVFKWVIRKEFEELHRYIDEEKATFLESVEGKAAQLITSI

ESQVKQTSDTLQRLKEMQSILETLGNESQLDFIRKYSSYQFRSELPSLHPGDTIFSPVSFKPCFHQDDIK

MTVWKRLHRHVLPAPEMLKLDPVTAHPLLELFKGDTVVQCGLYQRRDSNPKRFDSSNCILTCKGFSCGQH

YWEVIVGTRNHWRVGIIKGTVSRKGKLSKSPENGVWLIGLKEGKVYEAFSTPRATLPLTARPQRIGIYLH

YEKGELTFYNADSPDELSPIYTFQAEFQGQLYPIVDLCWPERGPFSPPIILPAPAAGRHSQGPPAVEEPT

KL

>chTRIM54_XP_015140486.2

MNFAVGLKPLLAEARSMESLEKQLICPICLEMFTKPVVILPCQHNLCRKCANDVFQASNPLWQSRGSGAG

PSGGRFRCPSCRHEVVLDRHGVYGLQRNLLVENIIDIYKQESARPLHAKAEQQLMCEEHEDERINIYCLR

CEAPTCSLCKVFGAHKDCEVAPLPAVYQRQKSELSDGIAMLVAGNDRIQAIITQMEEICRTIEENGRRQK

QHVGLRFDSLCSILEERKKELLQSIAREQEAKVQRVRGLIRQYGDHLEASSKLVETAIQAMEEPQMAVYL

QHSKELLKKITDMSKVSMSSRPEPGYESMDHFSINVDHVAEMLRTIDFQPEALGEDDGDGPTDSSEGVGD

EESLEAPEAAEDVGPRQKPLSSPHGQH

>chTRIM55_NP_001186333.1

MSTSLGYKSFSKEQQTMDNLEKQLICPICLEMFTKPVVILPCQHNLCRKCASDIFQASNPYLPTRGGTTV

ASGGRFRCPSCRHEVVLDRHGVYGLQRNLLVENIIDIYKQESTRPERKCDLPMCEEHEDERINIYCLNCE

IPTCSLCKVFGAHKDCQVAPLTNVYQQQKSELSDGIAVLVGSNDRVQGIVTQLEETCKTVEECSRRQKEQ

LCEKFDYLYSVLEERKNEMTQIITRTQEEKLEHVRSLMKKYADHLEAVSKLVESGIQFMEEPEMAVFLQN

AKTLLQKITEASKGFQMEKIEDGYENMNQFTVNLSREEKIIREIDFDREEEAEEEEEETVEGEDLDEVHT

ESSGEEGEEEEKEEEEGAERAPQPPQQDPEQQNAVGEPPADPVPVPLPAAPAGQAASEPSPAGQAEAAAG

SSEQSTEPTRHIFSFSWLNSLTE

>chTRIM59_NP_001026491.1

MHQFEEELTCSICYSLFEDPRVLPCSHTFCRSCLEGVIQLSSNFSIWRPLRVPLKCPNCRSIVEIPASGT

ESLPINFALKAIIEKYRQEDHSDVATCSEHYRQPLNVYCLLDKKLVCGHCLTIGKHNGHPIDDLHSAYLK

EKESSGKILEQLTDKHWSDVCLLIEKLKEQKAQCESIVQDDKKVVVQYFKKLSETLEHKKQVLLAALDEI

NRQILEEYEPHIEKLKKIREEQLELMSLNTSIQKEESPLVFLEKVDNVHQRIKALKEKELPDVKPVEVYP

RVGHLLKDVWSKTEIGQINKILTPKIKLVPKRKLHSKNSEKERGKPEELLQAANPLSVTFIFTVIIAIAV

LSFHKPISSVVIESIPTHISDFFGFLYQDFCTCMQNTVDVVCHKLNSLAEFLGGIVPF

>chTRIM215_XP_015140854.2

MAAGPAEQGWRQRGEPGPAEMEPRPRSLVCSSQMEASLTCAVCLSLFEEPVTLPLCSHNFCRGCVLECLA

SAEAARLQQQQQRGQGQVRGARRGSGPGQGPDGDDAAGARVSCPLCRKLCPLPRGGAAALPVNTTLAEVV

RLYRSNAARAGGSCQKHPGRLLQLYCRMCRQAGCGQCVSEEHQGVFHSINLIDTVYQEEQLNFFSSLKEM

RIINEKLMNEISSPPNDTDMVLNNEAEIIALAFEEIFKTLEMKKQQLTEDLENQRRKKEKEFQIWKKMKE

THKKTIENLLNDCEKLVHECDPQHFLEVACGLNTRMKTQLDLMNIASSYKKPPEYTQKKMDIKSVVNEIL

ALKLVPVNVDINTDPPSGGNENSTENIIKQWQDQKNTPNSFLPVAGQEEALTDSGRICTRLMSVSEMPVF

QNWSHEELRYKYYMEHQKFNRLKTQTLPASKKYEFVAAEALKSKSSGIPLVSSTTKPNNVDRVKAEALSR

EGVIDKTSFPGTSNHCTPSTSTKLSETNGDLSLAHERSSEEATTPALPENSKDQEIKENLPMQSSAVTVS

NETNANSSVSLGLKPAASVAVTVSNSNLDFSASGACSNSLPRFIKDAATCSLKDNKYAFPKFYLGKCDRE

DKADNQCESKFGKCSSVAKTRVSDASTACNSESAASQKPVYSFSYGSSEREYLAASEVSDSFKTLSLYSF

FKQSEKTSDLNVLSHMGKNTSSLKKPVDGTLKPSVSWEQNNNASESITTVLCGTSETTTAAGVNVISESS

LLPSSCVFSFKNNSSQLLPNEIAGEDIVKNTSGSLTSSSVLLSRNGTDKTEKEKMKSLGKTPLNLGNSAY

PMCAEPASRLRHRKCEGSSLCTNSSENAGSTDILANINSSCTQPLCSAVLPVNNGNASSTQLTTTKQEIK

VKHQENKITAESNYSCPRREEEFESVPFQNTACSAPEARNDLPSVDSVLAVNEAREMSSDSDSDTEKLSQ

TSVSSDTSNASEYFSVTEDKMSARRKSET

>chTRIM62_XP_015152721.2

MACSLKDELLCSICLSIYQDPVSFGCEHYFCRRCITEHWVRQEPQGARDCPECRRTFTEPTLAPSLKLAN

IVERYSAFPLDAILGAQRSPFPCKDHEKVKLFCLTDRTVVCFFCDEPAVHEQHQVTNVDDAFEELQRELK

EQLQGLQESERGHTEALHLLKRQLADTKSSAKSLRATIGEAFERLHRLLRERQKAMLEELEADTARTLTD

IEQKIQRYSQQLRKVQEGSQILQERLAEADKHAFLAGVASLSERLKGKIHETNLTYEDFPTSKYMGPLQY

TIWKSLFQDIHPVPAALTLDPGTAHQRLILSDDCTIVAYGNLHPQPLQDSPRRFDVEVSVLGTQAFGGGV

HYWEVVVSEKTQWMIGLAHEAVTRKGSIQIQPSRGFYCIVMHDGNQYSACTEPWTRLNVKSKLEKVGVFL

DYDKGLLIFYNADDMSWLYTFREKFPGKLCSYFSPGQSHANGKNVQPLRINTVRI

>chTRIM63_XP_015153240.2

MDKSCARGSGCRTGVALRRSSSAVPMDFQPSILRDGSPMESLEKQLLCPICLEMFSKPVVILPCQHNLCR

KCANDIFQAANPYWQSRGSSIISGGRFRCPTCRHEVLLDRHGVYGLQRNLLVENIIDIYKQEFSSRPLKK

GEHPMCKEHEDERINIYCVTCEVPTCSMCKVFGAHKDCEVAPLQSIFQGQKSELNNCISMLVAGNDRIQT

IISQLEDSCRSTEENSEAAKQELCARFDAFSALLEEKKTELLGRITREQEDKTSFVQGLIHKYKEQLEKS

SRLVETAIQAMEETGGAAFLMNAKQLIKTIVEASKGGRLEKIEQGYENMDAFTVSLEHLADAVRALDFEP

DEEDEFYEEVEEDTGDTEPERVVAAPQ

>chTRIM65_XP_415625.3

MASPVLHKLEDKLLCSICLELFRVPVTLPCGHNFCERCINDHRSKQEQTADGAERGYKCPQCRSSCERQL

ELKKNVTLSEVVEVARASRVWAESCEVAHVGLCPQHGRPLELYCEDEQRCICCVCSVRQCQRHRRALFGD

ERSRKQALLERSLEETQREAERMERALRELEERTQSIKDSSERLQAVILSKFTHLEEALAKFQEQMVGKV

KQEELVALGHIEKDRNALKDHLEALGQHRDRAQYLLDCSDHQVFLEEFPQLLTPRSREVPPLVEFNAAVV

VEPMNEILAATSRLLLEELPASLSPSAPDPIAPGPVQPEETAVKVVSTPAPMCQLRAELLKDHRNLTFDP

DTANKYLELSWGQRRAKHGPTAAVGWQERGRCFEPWQVLCAQSYGQGCHYWEVTISSHSVVLGVTYGGLP

RHKVRGCKFNIGLDGGSWGLQVREDSYLAWHKGQEEKIQLRLYTKLGVCLDCDHGLLSFYGLGEGVQHIH

SFHSVFTEPLFPVFWLCEGRTVTLCHRDCGGPEGELGL

>chTRIM66_XP_040529288.1

MSHFVTLRVFDVKPQPTAMEPLDVLRKCLVCKWDLCRRDPRMLPCLHSFCKDCLPELIQGYSYISTGREI

VYEGILSCLACKQTCFARDVVENCFLKDFPTANSAMAKSCSMCKEKRPAHSLCTSCNKWLCSTCTEEHRH

GKETGDRFLSVSLKGCTATEEEASELPLLCPVHTQEPLKLFCETCDILTCSSCLLKDHKEHRFRHLDEAL

QNQRAILENVVTKVEEKKSGIQVSAKQIEDRLLEVKHLYKRVENQIKMAKMVLINEINKRANILLEQLEK

ITNERRQKLEQQLQGVVVLNAQVEHVQNFVNWAVCSKNSIPFLFSKELIVFQIQRLLETNPDTDIAPPLK

IRFTWDPSYWSKQLSNFGGFTMEGGHTSHSDVLLCGNVQGLQTPLYHGHCSPVSQLEPTNSQPHQFPPAS

PCPIPVCCSHCLSVPHPNKAQPSHQNISPHQHFRQPPELHQQHFPLQYSMQQRDKEQRGVSQPLKPSQSE

LEQQSRSESENGSGRMGKHLPSQLQQTVPLGYAVVSHEAQQVHTSHPQPFRSQTALQTSTVQVQLGHLQK

IKSNYVQQPPQQQLSPASPPPSQYENTHKQVMQQSLDIMHHQFELEEMKKDLELLLQAQGQSSLQLNQAK

PPQHVQQTIVGQINYIVRQPAPIQQQIEDEAQVCENSTELDAQKPAVPLDRSKISSMSQSLDEEVSVSSH

SPGTTSQQSTSHPVRKRSASLGIVGFSNELEMELPSRLSRSADPQVQGIATVALGPSPNTRCDCDSPPEP

VPSYSLALGRAELSPGVSTTFTPGDVHQGSAKCKLENEDFNTVDHHPENSLATAGQDVVNELSLSMQNIL

EEPINLSVKKSQRCTSPSELLSNNSLLPVNARMRPLRNEEDSNNCEKEHFEMVMKGNQNVSACLGELKIP

YVRLERLKICASESGELPVFKLQPQESGQEGNFLLIIEHGTQSSRMAIKINKGGSPEGLKSEEENSDDRK

FLITQAAGQIKSPPADAPSVDQKLTNGTSITKKSPVTQEVNTIENEDFCAVCLNGGELLCCDHCPKVFHL

SCHVPALLSFPVGEWVCTLCRNPMKPEVEYDCENTRYAHSYNAQYGLDDYDQKKCEKLVLSLFCSSMSLP

FHEPVSPLARHYYQIIKRPMDLSIIRKKLQKKDKFHYSAPEELVTDVRLMFWNCAKFNYPDSEVAEAGRC

LDVFFEGKLKEIYPDRHFPSAQQDDSDCEELESQNSKMPPQDFHWPSHEQECAQPKKRRRHTGSDKTKRM

TFQTANSLPQV

>chTRIM67_XP_025004963.1

MEEELKCPVCGSLFREPIILPCSHNVCLPCARTIAVQTPENEQHLPALLQPRGSAPPAAAAAAPGTGLGP

GPAASLDSECAAGGGVDHADKLSLHSETDSGYGSYTPSLKSPNGVRVLPLVPAPPGAAAAQRGAGPAGSS

LTCPQCHRSASLDQRGLRGFQRNRLLEAIVQRYQQGRAAAAAAKCQLCDRSPPEPAAVLCEQCDVLYCAA

CQLRCHPARGPFAKHRLVPPPGAPGPPGGGDGGGKGAGGGRKLPTCAEHDLENYSMYCVSCRSPVCYQCL

EDGRHGKHDVKALGAVWKQHKAQLSQALNGVSDKAKEAKEFLVQLKNLLQQIQENGLDYEACLVAQCDAL

VDALTRQKAKLLTKVTKEREHKLKVVWDQINHCTLKLRQSTGLMEYCLEVIKENDPSGFLQISDALIKRV

QVSQEQWVKGALEPKVSAEFDLTLDSEPLLQAIHQLDFIQMKLPPVPLLQLEKCCTRNNSVTLAWRMPPL

SHNPVEGYILELDDGDGGQFREVYVGKETLCTIDGLHFNSTYNARVKAFNSSGVGPYSKTVILQTSDVAW

FTFDPSSAHRDIVLSNDNQTATCSSYDDRVVLGTAAFSKGVHYWELHVDRYDNHPDPAFGIARINVVKDM

MLGKDDKAWAMYVDNNRSWFMHCNSHTNRTEGGVCKGATVGVLLDLNKHNLTFYINGQQQGPPAFENVEG

VFMPALSLNRNVQVTLHTGLEVPQCVKQPKLPSN

>chTRIM71_NP_001032352.2

MASFPEADFQVCPLCKEMCGSPAPLSSNSSTSSSSSQTSGSSGGGGSSCGGAARRLHVLPCLHAFCRQCL

EAQRHPAAGDALKLRCPICDQKVVISEPSGMDALPSSNFLLSNLLDVVVVAAAADEQKNGRAAGSGPAAG

SGAGGGGANNRHHGRPPPHRAAAAASSPAAGSAAPSASSSSSSGGGGPAALLLRRPHSRQGEPRCSSCDE

GNAASSRCLDCQEHLCDNCVRAHQRVRLTKDHFIERFAAGPAAASAGPAAPLALSPPYPASPYNILSVFP

ERASYCQHHDDEVLHFYCDTCSVPICRECTMGRHVGHSFIYLQDALQDSRTLTIQLLADAQQGRQAIQLS

IEQAQAVAEQVEMKAKVVQSEVKAVTTRHKKALEERECELLWKVEKIRQVKAKSLYLQVEKLRQNLNKLD

NTISAVQQVLEEGRTIDILLARDRMLAQVQELKNVRGLLQPQEDDRIMFTPPDQALYMAIKSMGFVSSGA

FAPLTKATGEGLKRALQGKVASFTVIGYDHDGEPRLSGGDMISAVVMGPDGNLFGADVSDQQNGTYLVSY

RPQLEGEHLVSVMMCNQHIENSPFKVVVKSGRSYIGIGLPGLSFGSEGDSDGKLCRPWGVSVDKEGYIIV

ADRSNNRIQVFKPCGTFHHKFGTLGSRPGQFDRPAGVACDVSRRIVVADKDNHRIQIFTFEGQFILKFGE

KGTKNGQFNYPWDVAVNAEGKILVSDTRNHRVQLFGPDGVFLNKYGFEGALWKHFDSPRGVTFNHEGHLV

VTDFNNHRLLVIHADCQSARFLGSEGSGNGQFLRPQGVAVDQEGRIIVADSRNHRVQIFESNGSFLCKFG

AQGSGFGQMDRPSGIAVTPDGMIVVVDFGNNRILVF
